# Orthogonally Protected Diaminocyclopentenones as Synthons: Total Synthesis of (±)-Agelastatin A

**DOI:** 10.1021/acs.orglett.3c01513

**Published:** 2023-05-30

**Authors:** Rafael F. A. Gomes, João R. Vale, Juliana G. Pereira, Carlos A. M. Afonso

**Affiliations:** Research Institute for Medicines (iMed.ULisboa), Faculty of Pharmacy, Universidade de Lisboa, Avenida Professor Gama Pinto, 1649-003 Lisbon, Portugal

## Abstract

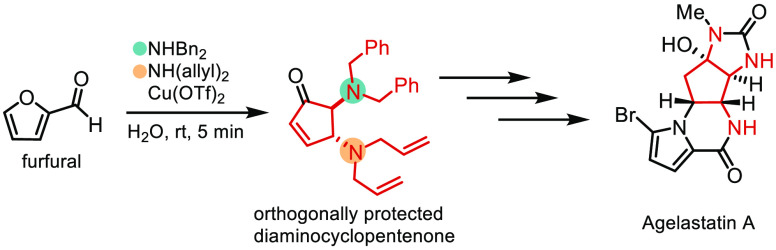

Natural products containing aminocyclopentanes are common secondary metabolites, often biologically active. This work aims at the preparation of a useful synthon for total synthesis containing orthogonally protected amines. To this end, furfural and two amines were employed to form mixed *trans*-4,5-diaminocyclopentenones promoted by Cu(OTf)_2_. The selected amines can be orthogonally deprotected, allowing selective modification of the amines on the cyclopentane core. Their utility was showcased for the total synthesis of highly complex (±)-Agelastatin A.

Nature provides synthetic organic chemists with several challenges in the form of complex natural products. These metabolites often exhibit remarkable biological activity such as antitumoral and antimicrobial, among others. A variety of these biologically active natural products share a common aminocyclopentane core. These have been isolated from various sources such as the plant *Alstonia macrophylla* (indole alkaloids),^1^ the bacteria *Streptomyces pactum* (pactamycin, pactamycate, and jogyamycin),^2^ and deep sea marine sponges of the genus *Agelas* (nemoechine A,^3^ agelamadins,^4^ and agelastatins^5^) as depicted in Figure 1A. Despite the pharmacological importance of these compounds, poor availability hinders drug development endeavors. For this reason, the scientific community has tackled this issue by performing the total synthesis of the active metabolites, hence bypassing the availability concerns. Moreover, the synthetic challenge of creating these complex natural products has inspired many groups to develop novel technologies transversal to other scientific communities.

**Figure 1:**
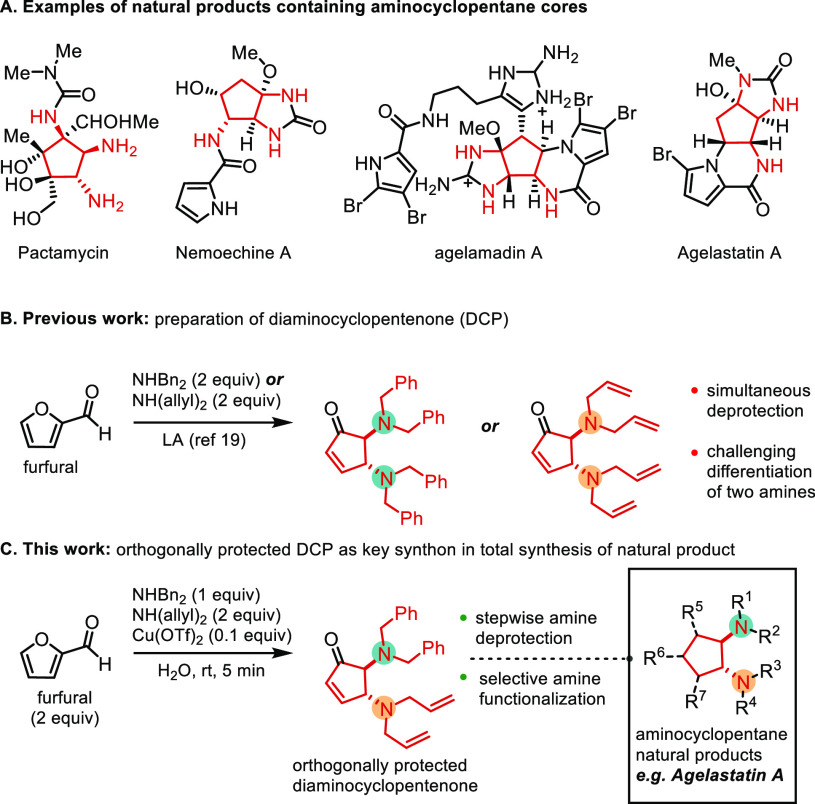
Natural products containing aminocyclopentane cores (A), the preparation of DCPs (B), and this work (C).

Due to the increased availability of synthetic Agelastatin A (AglA), the mechanism for its cytotoxicity was recently elucidated,^6^ highlighting the importance of total synthesis. The study by Liu and co-workers identified the eukaryotic ribosome as a cellular target of AglA using a systematic top-down approach, culminating in a cocrystallization of the alkaloid and the 80S eukaryotic ribosome from *S. cerevisiae*.

Several strategies have been employed for the formation of the cyclopentane core,^7^ such as bond insertion of an alkylidenecarbene,^8^ metathesis reaction,^9^ imidazolone-forming annulation reaction followed by a carbohydroxylative trapping of imidazolones,^10^ and polar 5-exo-trig cyclization,^11^ among others.

Concerning the introduction of the amine moieties, often aziridination^12^ is used to introduce both an amine and a carbon electrophilic center to undergo further amination. Additionally, other approaches involve conjugate additions,^13^ palladium-catalyzed asymmetric allylic alkylation (AAA),^13,14^ [3,3]-sigmatropic rearrangement of cyanates,^15^ and Mitsunobu reaction.^16^

In an attempt to reduce the number of steps toward the final products, Batey and co-workers designed an elegant approach in which a single operational step delivered a diamino carbocycle used in the total synthesis of AglA.^17^ To this end, the authors optimized the formation of *trans*-4,5-diaminocyclopentenones (DCPs) from biomass-derived furfural promoted by dysprosium trifluoromethanosulfonate under mild conditions,^18^ which have been further studied by several research groups (Figure 1B).^19^

One DCP was used as a diaminocyclopentane synthon through deprotection of the diallylamine group.^17^ Despite the concomitant reduction of reaction steps and the increase in yield provided by the one-step introduction of the two amines and formation of the carbocycle, a major challenge of this approach is the differentiation of the two primary amines formed upon deprotection of the DCP. The authors overcame this issue via a selective coupling of bromopyrrole lithium carboxylate and one of the primary amines.

Inspired by this approach, we envisioned that the preparation of orthogonally protected DCPs would allow sequential modification of the amines, broadening the toolbox of the DCP system as synthons for the preparation of other diaminocyclopentane natural products and AglA analogues, in particular with different amine substituents (Figure 1C).

The reversibility of cyclopentenone–Stenhouse salts, despite being uncommon on DCP, has been thoroughly studied in cyclopentenone–donor–aceptor Stenhouse adduct (DASA) systems developed by Alaniz and co-workers.^20^

Aligned with previous findings where DCP stock solutions appeared to be contaminated with furfural upon HPLC-UV analysis, we pursued the possibility of amine exchange in established cyclopentenones promoted by this equilibrium.

To this end, a mixture of DCP obtained from furfural and morpholine was stirred with 2 equiv of dibenzylamine in the presence of Cu(OTf)_2_. After 30 min a new product was identified as mixed CP **1a** (Figure 2). Further studies, including NOESY experiments, suggest the regioselective addition of morpholine in position 4 and dibenzylamine in position 5 (see SI). However, if the mixture was allowed to react longer to achieve full consumption of the initial DCP, a secondary product was obtained, consisting of the known 2,4-isomer cyclopentenone obtained by Aza-Michael addition of dibenzylamine followed by elimination of the amine to reform the enone system (Figure 2).

**Figure 2:**
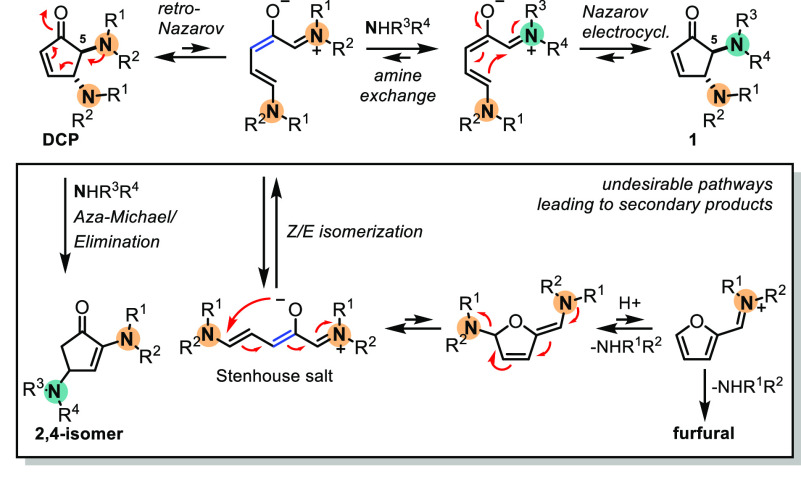
Proposed mechanism for the amine exchange in position 5 and for the secondary product formation.

This observation prompted us to react furfural under our previously reported conditions,^19b^ in the presence of stoichiometric amounts of dibenzylamine and morpholine. Upon isolation, the mixed CP **1a** was isolated as a minor product in 38% yield (Table S1, entry 1), with the majority of the mass balance being the bisdibenzylamine DCP.

The reaction was optimized by screening different solvents, reaction times, and amine equivalents. The optimal conditions were identified as 1 equiv of dibenzylamine and 2 equiv of morpholine and furfural under aqueous conditions for 5 min (for more detailed information see Table S1).

Attempts at developing an enantioselective methodology were fruitless, and a detailed description of our attempts is available in the SI.

With the optimized conditions in hand, the reaction was extended to other amines (Scheme 1). Used in conjunction with dibenzylamine, all three morpholine, piperidine, and diallylamine afforded the desired mixed product as a single regioisomer in good yields (**1a**–**1c**, 59–70% yield). Simultaneous use of *N*-methylaniline and morpholine gave as a major product the DCP containing the arylamine in position 5 (**1d**, 62% yield), although traces of product with the arylamine in position 4 were detected, which could not be properly purified for characterization. When the reaction was performed in the presence of tetrahydroquinoline (THQ) and morpholine, the major product exhibited THQ in position 5 (**1e**, 68% yield), similar to dibenzylamine. These results indicate a trend that benzyl and aryl amines tend to be incorporated on the carbocycle in position 5, whereas alkyl amines are incorporated in position 4.

**Scheme 1:**
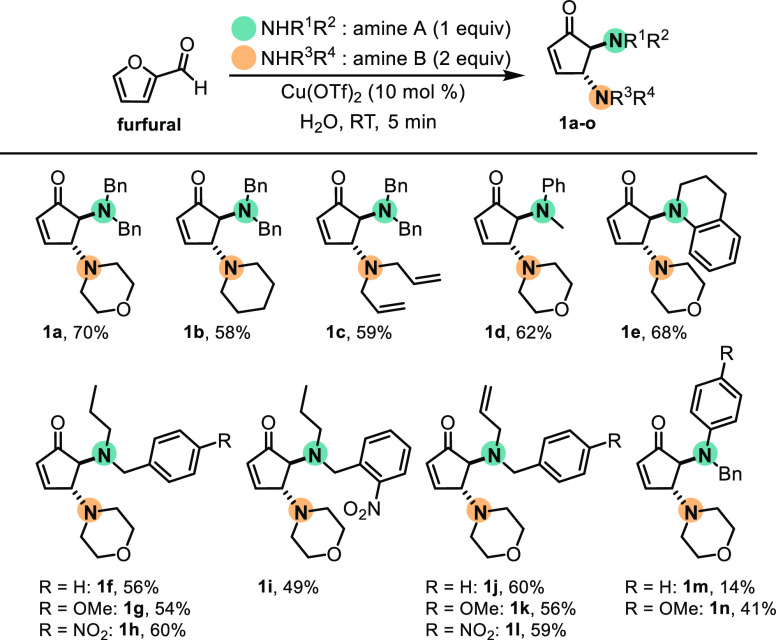
Amine Scope for the Formation of Mixed DCPs Directly from Furfural Reaction conditions: furfural (200 mg, 2.2 mmol), amine A (1 equiv, 1.1 mmol), amine B (2 equiv, 2.2 mmol), Cu(OTf)_2_ (10 mol %, 0.1 mmol), H_2_O (1 mL).

Moreover, a selection of amines encompassing protecting groups were prepared by reductive amination and reacted with furfural in the presence of morpholine. The selected examples were *N*-benzylpropan-1-amine, *N*-(4-methoxybenzyl)propan-1-amine, *N*-(4-nitrobenzyl)propan-1-amine, and *N*-(2-nitrobenzyl)propan-1-amine which can be deprotected under hydrogenation conditions, oxidative conditions,^21^ basic conditions,^22^ and UV irradiation^23^ correspondingly. The propyl-benzylamines afforded the desired products in moderate to good yields (**1f**–**1i**, 49–60% yield). Additionally, orthogonally protected *N*-allyl-benzylamines also reacted with furfural and morpholine, affording the desired products in good yields (**1j**–**1l**, 56–60% yield).

Finally, *N*-aryl-benzylamines were also employed, in which the desired mixed DCPs were obtained with electron-rich arylamines. However, the reaction did not withstand electron-withdrawing groups such as 4-chloro, 4-CF_3_, and 4-nitro anilines. Noteworthy, the reaction afforded the mixed DCP as a major product in the case of electron-rich aniline **1n**, the remaining mass being in the form of bisaniline DCP (28%) and bismorpholine DCP, whereas in **1m** the product was only formed in low yield (14%), with no formation of the bisaniline DCP. This suggests a high dependency on the electronics of the arylamines. Hindered alkyl amines containing an α-substituent were not tolerated under the reaction conditions (for a full scheme of unsuccessful amines, see Scheme S1). Due to the reversible nature of the reaction, we cannot determine if the cause is (1) harder nucleophilic addition to the furan ring or (2) difficult electrocyclization.

Having the amine scope established, we further functionalized the CP core by Michael addition of thiols. It has been previously reported by our group and others that bismorpholine DCP undergoes Michael addition with thiols followed by base-promoted elimination of the morpholine in position 4 to re-establish the enone.^19b,19f^ However, in the presence of thiophenol under basic conditions, bisdibenzyl DCP was not reactive, and the starting material was recovered after 24 h (result not shown). For this reason, there are no reports on the preparation of 4-thio-2-enaminones from DCP, except for the bismorpholine derivative. Aiming at broadening the scope of 4-thio-2-enaminones, the new mixed DCP decorated with a morpholine in position 4 underwent thio-Michael addition followed by base-promoted elimination to yield the desired enaminones with a broad scope of amines in position 2.

Selected DCPs **1a**,**d**,**e**,**f**,**k** underwent Michael addition with 4-methoxythiophenol, affording the corresponding adducts **2a**–**e** in good yields (65–70%) (Scheme 2). These studies suggest a higher dependency on the amine substituent in position 4, compared to the other substituent. In this way, mixed DCP allows the preparation of otherwise unattainable nonmorpholine thio-enaminones.

**Scheme 2:**
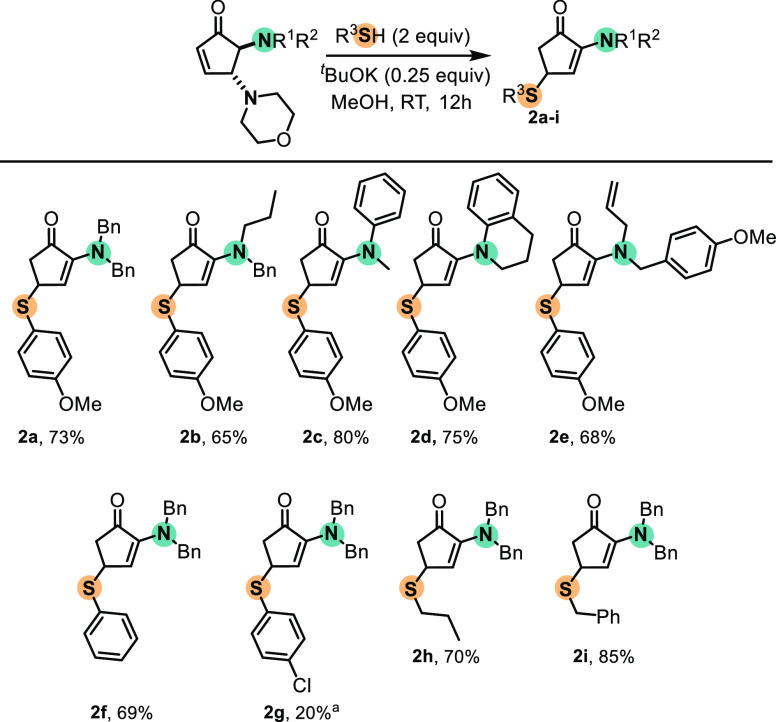
Scope for the Michael Addition to Mixed 4,5-Diamino CPs Reaction conditions: CP (0.3 mmol), thiol (1 equiv, 0.3 mmol), *t*BuOK (25 mol %, 0.075 mmol), MeOH (3 mL). Required 5 equiv of thiol.

Reaction of **1a** with different aryl thiols afforded the corresponding products **2f**–**i**. Electron-donating groups on the aryl thiol favored the addition, whereas electron-poor 4-chloro-thiophenol required an excess of thiol. Only 20% of the desired product **2g** was obtained. Reaction with alkyl thiols afforded **2h** and **2i** in good yields (85 and 70%, respectively).

Noteworthy, if the reaction is performed using NaOMe as the base, the addition of the alkoxide to the enone yields the corresponding 4-hydroxy-cyclopentenone in 54% yield (see Supporting Information).

To showcase the potential of the new orthogonally protected diaminocyclopentane core, DCP **1c** was used as a key intermediate for the total synthesis of AglA. The stepwise deprotection would allow bypassing the challenges of amine differentiation, previously encountered by Batey and co-workers.

Attempts at deallylation in the presence of the enone always led to decomposition, and the protection of the ketone was not feasible under common conditions. Despite the poor “redox economy”, the only viable approach to inhibit decomposition of the enone was to reduce the carbonyl and oxidize the allylic alcohol just before formation of the B ring (Scheme 3). Hence, DCP **1c** was reduced with DIBAL-H to the corresponding allylic alcohol with high diastereoselectivity (>20:1 dr). Other 1,2-reductive agents were attempted unsuccessfully such as CeCl_3_/NaBH_4_ (Luche reduction), l-Selectride, and LiAlH_4_. The crude reaction mixture was reacted with TBSCl and imidazole to yield the corresponding protected alcohol **4** in 92% yield over the two steps.

**Scheme 3:**
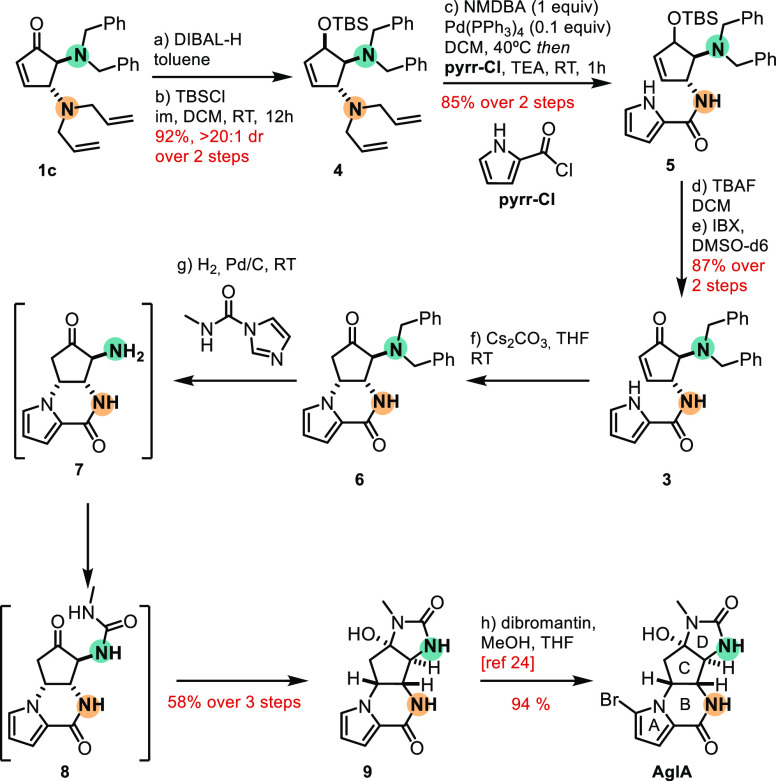
Total Synthesis of Natural Product AglA from DCP **1c**

With the enone masked as a protected allylic alcohol, the deallylation occurred in the presence of *N*,*N*-dimethylbarbituric acid (NMDBA) and Pd(PPh_3_)_4_. However, the corresponding primary amine was challenging to purify and was therefore trapped with pyrrole acyl chloride. The corresponding amide **5** was obtained in 85% yield over two steps, without the challenges of amine differentiation engaged by previously reported procedures due to the stepwise amine deprotection. Before deprotecting the dibenzylamine, the formation of ring B was addressed to remove the conflicting olefin. To this end, deprotection of the silyl ether with TBAF afforded the desired alcohol which was used without further purification. The alcohol was readily oxidized to enone **3** with IBX in almost quantitative yield. With **3** in hand, the synthetic plan toward the desired final product was straightforward since it had been previously studied by Davis and co-workers.^9,24^ Avoiding possible side products (e.g., elimination of the amide leading to the 2,4-enone isomer and dimerization of the α-amino ketone), the reaction was performed in one pot by first stirring enone **3** with Cs_2_CO_3_, followed by addition of the Pd/C catalyst and methylamine-carbamoylimidazole. The reaction was stirred under a hydrogen atmosphere, leading to deprotection of the dibenzylamine and formation of the urea **9**. Spontaneous formation of the D ring was observed, without detection or isolation of intermediate **8**.

The bromination of the pyrrole was performed following previously reported conditions^9,24^ originating (±)-Agelastatin A (AglA) in 94% yield, corresponding to an overall yield of 26% in 6 steps. The one-pot procedures in combination with the late dibenzylamine deprotection hindered the formation of side products and facilitated the chromatographic purifications.

In summary, the preparation of mixed DCP was successfully performed with two amines, in a regioselective manner. Benzyl and aryl amines are incorporated preferentially in position 5 in comparison with alkyl amines that are incorporated in position 4 of the cyclopentenone ring. This affords an orthogonally protected diamino-cyclopentane core that can be explored for further applications.^25^ The flexibility of this approach allowed the introduction of an amide in position 4 and urea in position 5. Showcasing the potential of orthogonally protected DCP, (±)-AglA, a natural product isolated from marine sponge *Agelas dendromorpha*, was prepared in 6 steps with an overall yield of 26%. Further application of this platform may lead to the development of elegant and efficient strategies for total synthesis of natural products containing diaminocyclopentane cores.

## Data Availability

The data underlying this study are available in the published article and its Supporting Information

## Supporting Information Available

General procedures, experimental details, characterization data, ^1^H and ^13^C NMR spectra, gCOSY, gHSQC, and gHMBC experiments, and HPLC chromatograms (PDF)

## ABBREVIATIONS

AglA: Agelastatin A
DCP: *trans*-4,5-diaminocyclopentenones
DASA: donor–aceptor Stenhouse adducts
THQ: tetrahydroquinoline
NDMBA: *N*,*N*-dimethylbarbituric acid
TLC: thin-layer chromatography
