# Supplementary material for: Orthogonally Protected Diaminocyclopentenones as Synthons: Total Synthesis of (±)-Agelastatin A
Source: Org Lett. 2023 May 30;25(22):4188–92. doi: 10.1021/acs.orglett.3c01513 (PMC10262273; doi:10.1021/acs.orglett.3c01513)
Supplement: Supplementary file 1 — ol3c01513_si_001.pdf [file ol3c01513_si_001.pdf]

Orthogonally protected diaminocyclopentenones as synthons: Total synthesis of (±)-Agelastatin A

Rafael F. A. Gomes,[a]* João R. Vale,[a] Juliana G. Pereira,[a] Carlos A. M. Afonso[a]*

[a]Research Institute for Medicines (iMed.ULisboa), Faculty of Farmacy, Universidade de Lisboa, Avenida Professor Gama Pinto, 1649-003 Lisbon, Portugal

Table of Contents

General information S2

General procedure for the reaction optimization S2

General procedure for the preparation of mixed trans-4,5-diamino-cyclopent-2-enones S2

General Procedure for the Preparation of 2-amino-4-thio cyclopentenones S10

Attempts at protecting the enone **1c** towards the total synthesis of Agelastatin A S15

Total Synthesis of (±)-Agelastatin A S17

Enantioselective attempts S24

NMR Characterization S31

General information:

All solvents were distilled prior to use. All reagents were used as received from commercial suppliers, unless otherwise stated. ^1H and ^{13}C NMR spectra were acquired on Bruker MX300 spectrometer. HPLC analysis was performed on a Thermo Scientific Dionex Ultimate 3000 apparatus with a LPG- 3400SD Pump, a UV MWD-3000(RS) detector and an autosampler ACC-3000, equipped with a 20 µL loop, using a reversed-phase EC 250/4 Nucleodur 100-5 C18ec column (250×4 mm, 5 µm) Thermo ScientificTM DionexTM. Shimadzu LC-20AT prominence liquid chromatograph with SPD-M20A diode-array detector was used alongside HPLC CHIRALPAK® IC 5 µm 4.6 mm x 250 mm chiral HPLC column for chiral analysis. Chromatograms were processed with Shimadzu LC solution software. Structural assignments were made with additional information from gCOSY, gHSQC, and gHMBC experiments

General procedure for the reaction optimization

To a solution of $Cu(OTf)_2$ (7.5 mg, 10 mol%) in water (0.2 mL) was added dibenzylamine (41 mg, 0.20 mmol, 1 equiv.), morpholine (1 or 2 equiv.), and furfural (20 mg, 0.2 mmol). The reaction was allowed to stir vigorously at room temperature for 5 minutes. Then the reaction mixture was diluted with water (2 mL) and extracted with MTBE (3 × 2 mL). The combined organic phases were dried with $MgSO_4$, the solvent was evaporated under reduced pressure and the crude mixture was purified by column chromatography to quantify the product yield.

Table S1. Reaction optimization towards mixed diaminocyclopentenones.[a]

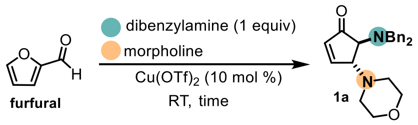

Entry	Time (min)	Solvent	Morpholine (equiv.)	1a (%)
1	5	H_2O	1	38
2	5	H_2O	2	46
3[b]	5	H_2O	2	72
4[b]	15	H_2O	2	52
5[b]	5	MeCN	2	0
6[b,c]	5	H_2O	2	47

General procedure for the preparation of mixed *trans*-4,5-diamino-cyclopent-2-enones

To a solution of $Cu(OTf)_2$ (80 mg, 10 mol%) in water (2 mL) was added amine A (1.04 mmol, 1 equiv.), amine B (2.08 mmol, 2 equiv.), and furfural (200 mg, 2.08 mmol). The reaction was allowed to stir vigorously at room temperature for 5 minutes. Then the reaction mixture was diluted with water (18 mL) and extracted with MTBE (3 × 20 mL). The combined organic phases were dried with $MgSO_4$, the solvent was evaporated under reduced pressure and the crude mixture was purified by column chromatography.

5-(dibenzylamino)-4-(morpholin-1-yl)cyclopent-2-en-1-one (1a)

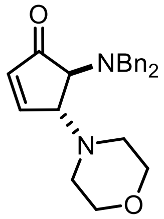

The title compound was prepared using dibenzylamine as amine A and morpholine as amine B according to general procedure. The crude mixture was purified by flash chromatography using hexane:ethyl acetate (9.5:0.5 to 8:2) affording 263 mg (70%) of pure product as a yellow oil.

^{1}H NMR (300 MHz, $CDCl_3$) δ 7.36 (dd, J = 6.3, 2.1 Hz, 1H), 7.32 – 7.25 (m, 4H), 7.19 (t, J = 7.2 Hz, 4H), 7.12 (d, J = 7.1 Hz, 2H), 6.08 (dd, J = 6.2, 1.8 Hz, 1H), 3.79 (d, J = 13.3 Hz, 2H), 3.56 (d, J = 13.2 Hz, 2H), 3.52 (dd, J = 3.4, 1.8 Hz, 1H), 3.43 (t, J = 4.5 Hz, 4H), 3.38 (d, J = 3.2 Hz, 1H), 2.24 – 2.14 (m, 4H).**^{13}C NMR (75 MHz, $CDCl_3$)** δ 208.0, 160.8, 139.1, 135.3, 129.3, 128.3, 127.3, 68.6, 67.0, 62.8, 55.2, 50.2. **HRMS (ESI-MS)** *m/z* calcd for compound $C_{23}H_{27}N_2O_2$ [M + H]$^+$ 363.2067, found 363.2074.

5-(dibenzylamino)-4-(piperidin-1-yl)cyclopent-2-en-1-one (1b)

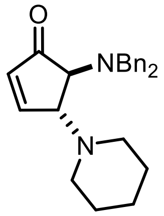

The title compound was prepared using dibenzylamine as amine A and piperidine as amine B according to general procedure. The crude mixture was purified by flash chromatography using hexane:ethyl acetate (9.5:0.5 to 8:2) affording 217 mg (58%) of pure product as a yellow oil.

^{1}H NMR (300 MHz, $CDCl_3$) δ 7.50 (dd, J = 6.2, 2.1 Hz, 1H), 7.48 – 7.20 (m, 10H), 6.19 (dd, J = 6.2, 1.9 Hz, 1H), 3.92 (d, J = 13.3 Hz, 2H), 3.76 (m, 1H), 3.78 – 3.64 (m, 2H), 3.60 (d, J = 3.0 Hz, 1H), 2.49 – 2.19 (m, 4H), 1.44 (q, J = 5.6 Hz, 4H), 1.36 – 1.17 (m, 2H). **^{13}C NMR (75 MHz, $CDCl_3$)** δ 208.8, 162.8, 139.5, 134.8, 129.6, 128.3, 127.3, 69.2, 61.8, 55.4, 50.8, 26.3, 24.3. **HRMS (ESI-MS)** *m/z* calcd for compound $C_{24}H_{29}N_2O$ [M + H]$^+$ 361.2274, found 361.2271.

5-(benzyl(phenyl)amino)-4-morpholinocyclopent-2-en-1-one (1c)

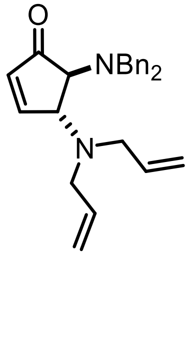

The titled compound was prepared using dibenzylamine as amine A and diallylamine as amine B according to general procedure. The crude mixture was purified by flash chromatography using hexane:ethyl acetate (9.5:0.5 to 8:2) affording 226 mg (59%) of pure product as a low melting point yellow oil.

^{1}H NMR (300 MHz, $CDCl_3$) δ 7.39 (dd, J = 6.2, 2.2 Hz, 1H), 7.37 – 7.32 (m, 4H), 7.29 – 7.14 (m, 6H), 6.10 (dd, J = 6.2, 2.0 Hz, 1H), 5.61 (m, 2H), 5.16 – 4.88 (m, 4H), 4.00 (m, 1H), 3.81 (d, J = 13.3 Hz, 2H), 3.61 (d, J = 13.3 Hz, 2H), 3.41 (d, J = 3.1 Hz, 1H), 2.83 (d, J = 6.2 Hz, 4H). **^{13}C NMR (75 MHz, $CDCl_3$)** δ 208.4, 163.2, 139.4, 136.5, 135.2, 129.5, 128.3, 127.3, 117.3, 64.6, 64.1, 55.3, 53.6. **HRMS (ESI-MS)** *m/z* calcd for compound $C_{25}H_{29}N_2O$ [M + H]$^+$ 373.2274, found 373.2265.

5-(methyl(phenyl)amino)-4-morpholinocyclopent-2-en-1-one (1d)

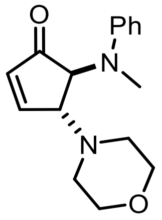

The titled compound was prepared using *N*-methylaniline as amine A and morpholine as amine B according to general procedure. The crude mixture was purified by flash chromatography using hexane:ethyl acetate (9.5:0.5 to 8:2) affording 175 mg (62%) of pure product as a yellow oil.

^{1}H NMR (300 MHz, $CDCl_3$) δ 8.10 (dd, J = 6.3, 2.0 Hz, 1H), 7.73 – 7.68 (m, 2H), 7.26 – 7.17 (m, 3H), 6.88 (dd, J = 6.3, 1.9 Hz, 1H), 4.88 (d, J = 3.4 Hz, 1H), 4.54 – 4.52 (m, 1H), 4.17 (t, J = 4.6 Hz, 4H), 3.30 (s, 3H), 3.09 (dt, J = 9.4, 4.6 Hz, 2H), 3.00 (dt, J = 11.3, 4.6 Hz, 2H). **^{13}C NMR (75 MHz, $CDCl_3$)** δ 204.2, 160.6, 148.9, 134.9, 129.3, 117.9, 113.4, 67.3, 67.1, 65.7, 50.1, 35.8. **HRMS (ESI-MS)** *m/z* calcd for compound $C_{16}H_{21}N_2O_2$ [M + H]$^+$ 273.1597, found 273.1589.

5-(3,4-dihydroquinolin-1(2H)-yl)-4-morpholinocyclopent-2-en-1-one (1e)

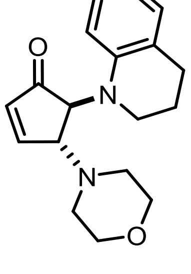

The titled compound was prepared using tetrahydroquinoline as amine A and morpholine as amine B according to general procedure. The crude mixture was purified by flash chromatography using hexane:ethyl acetate (9.5:0.5 to 8:2) affording 211 mg (68%) of pure product as a yellow oil.

^{1}H NMR (300 MHz, $CDCl_3$) δ 7.63 (dd, J = 6.3, 2.0 Hz, 1H), 6.97 (dd, J = 7.3, 1.4 Hz, 2H), 6.61 (td, J = 7.3, 1.1 Hz, 1H), 6.42 (dd, J = 6.3, 1.9 Hz, 1H), 6.33 (d, J = 8.0 Hz, 1H), 4.29 (s, 1H), 4.21 – 4.15 (m, 1H), 3.72 (t, J = 4.6 Hz, 4H), 3.22 (dd, J = 10.9, 5.5 Hz, 2H), 2.86 – 2.71 (m, 2H), 2.70 – 2.37

(m, 4H), 2.01 – 1.83 (m, 2H). **^{13}C NMR (75 MHz, CDCl$_3$)** δ 204.3, 160.1, 143.9, 135.1, 129.7, 127.1, 123.5, 117.0, 110.8, 67.2, 66.8, 50.2, 28.1, 22.4. **HRMS (ESI-MS)** *m/z* calcd For compound $C_{18}H_{23}N_2O_2$ [M + H]$^+$ 299.1754, found 299.1750.

5-(benzyl(propyl)amino)-4-morpholinocyclopent-2-en-1-one (1f)

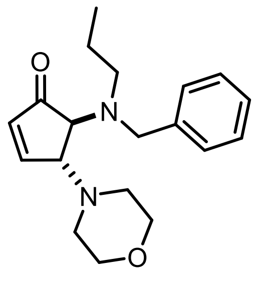

The titled compound was prepared using *N*-propyl-benzyl-amine as amine A and morpholine as amine B according to general procedure. The crude mixture was purified by flash chromatography using hexane:ethyl acetate (9.5:0.5 to 8:2) affording 183 mg (56%) of pure product as a yellow oil.

^{1}H NMR (300 MHz, CDCl$_3$) δ 7.43 (dd, J = 6.3, 1.9 Hz, 1H), 7.36 – 7.09 (m, 5H), 6.14 (dd, J = 6.2, 1.7 Hz, 1H), 3.86 (d, J = 13.6 Hz, 1H), 3.65 (d, J = 13.6 Hz, 1H), 3.57 (t, J = 4.7 Hz, 4H), 3.44 (d, J = 3.2 Hz, 1H), 2.71 (m, 1H), 2.51 – 2.27 (m, 4H), 2.27 (m, 1H), 1.46 (m, 2H), 0.81 (t, J = 7.3 Hz, 3H). **^{13}C NMR (75 MHz, CDCl$_3$)** δ 208.6, 160.9, 139.7, 135.5, 129.3, 128.2, 127.2, 68.9, 67.1, 63.9, 55.7, 53.7, 50.3, 21.8, 11.9. **HRMS (ESI-MS)** *m/z* calcd for compound $C_{19}H_{27}N_2O_2$ [M + H]$^+$ 315.2067, found 315.2067.

5-((4-methoxybenzyl)(propyl)amino)-4-morpholinocyclopent-2-en-1-one (1g)

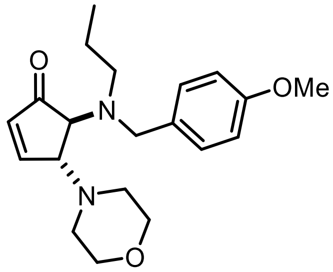

The titled compound was prepared using *N*-propyl-4(OMe)-benzyl-amine as amine A and morpholine as amine B according to general procedure. The crude mixture was purified by flash chromatography using hexane:ethyl acetate (9.5:0.5 to 8:2) affording 196 mg (54%) of pure product as a yellow oil.

^{1}H NMR (300 MHz, CDCl$_3$) δ 7.49 (dd, J = 6.3, 2.1 Hz, 1H), 7.36 – 7.16 (m, 2H), 6.84 (d, J = 8.7 Hz, 2H), 6.19 (dd, J = 6.2, 1.9 Hz, 1H), 3.86 (d, J = 13.4 Hz, 1H), 3.79 (s, 3H), 3.67 (d, J = 7.7 Hz, 1H), 3.64 (m, 4H), 3.50 (d, J = 3.1 Hz, 1H), 2.76 (ddd, J = 13.1, 8.3, 5.0 Hz, 1H), 2.63 – 2.31 (m, 4H), 2.38 (ddd, J = 12.9, 8.5, 7.1 Hz, 1H), 1.67 – 1.39 (m, 2H), 0.87 (t, J = 7.3 Hz, 3H). **^{13}C NMR (75 MHz, CDCl$_3$)** δ 208.6, 160.7, 158.9, 135.5, 131.8, 130.4, 113.7, 68.9, 67.2, 64.1, 55.4, 55.1, 53.7, 50.4, 21.9, 11.9. **HRMS (ESI-MS)** *m/z* calcd for compound $C_{20}H_{29}N_2O_3$ [M + H]$^+$ 345.2173, found 345.2170.

4-morpholino-5-((4-nitrobenzyl)(propyl)amino)cyclopent-2-en-1-one (1h)

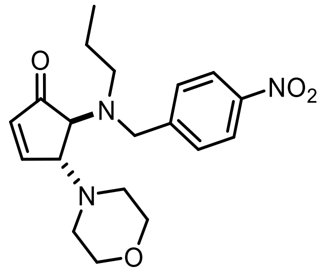

The titled compound was prepared using *N*-propyl-4(NO_2)-benzyl-amine as amine A and morpholine as amine B according to general procedure. The crude mixture was purified by flash chromatography using hexane:ethyl acetate (9.5:0.5 to 8:2) affording 224 mg (60%) of pure product as a yellow oil.

^{1}H NMR (300 MHz, $CDCl_3$) δ 8.11 (d, J = 8.7 Hz, 2H), 7.54 (d, J = 8.2 Hz, 3H), 6.20 (dd, J = 6.3, 1.8 Hz, 1H), 3.98 (d, J = 15.0 Hz, 1H), 3.83 – 3.58 (m, 6H), 3.45 (d, J = 3.2 Hz, 1H), 2.75 (ddd, J = 12.7, 8.6, 5.2 Hz, 1H), 2.54 (t, J = 4.6 Hz, 4H), 2.39 (ddd, J = 13.0, 8.8, 6.7 Hz, 1H), 1.62 – 1.35 (m, 2H), 0.82 (t, J = 7.3 Hz, 3H). **^{13}C NMR (75 MHz, $CDCl_3$)** δ 207.7, 161.3, 148.4, 147.5, 135.8, 129.7, 123.8, 69.0, 67.5, 64.9, 55.7, 54.5, 50.6, 22.1, 12.1. **HRMS (ESI-MS)** *m/z* calcd for compound $C_{19}H_{26}N_3O_4$ $[M + H]^+$ 360.1918, found 360.1915.

4-morpholino-5-((2-nitrobenzyl)(propyl)amino)cyclopent-2-en-1-one (1i)

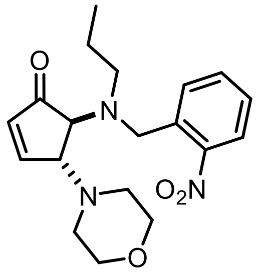

The titled compound was prepared using *N*-propyl-2(NO_2)-benzyl-amine as amine A and morpholine as amine B according to general procedure in a 0.50 mmol scale. The crude mixture was purified by flash chromatography using hexane:ethyl acetate (9.5:0.5 to 8:2) affording 92 mg (49%) of pure product as a yellow oil.

^{1}H NMR (300 MHz, $CDCl_3$) δ 7.81 (dd, J = 8.0, 1.4 Hz, 1H), 7.76 (d, J = 7.7 Hz, 1H), 7.55 (dt, J = 6.0, 2.3 Hz, 2H), 7.46 – 7.36 (m, 1H), 6.22 (d, J = 6.2 Hz, 1H), 4.20 (d, J = 14.7 Hz, 1H), 4.10 (d, J = 14.7 Hz, 1H), 3.82 – 3.68 (m, 1H), 3.89 – 3.48 (m, 4H), 3.39 (d, J = 3.1 Hz, 1H), 2.96 – 2.58 (m, 1H), 2.69 – 2.26 (m, 4H), 2.33 (m, 1H), 1.45 (m, 2H), 0.81 (t, J = 7.3 Hz, 3H). **^{13}C NMR (75 MHz, $CDCl_3$)** δ 207.6, 161.2, 150.1, 135.4, 134.6, 132.4, 131.9, 128.2, 124.5, 68.3, 67.1, 63.7, 54.1, 52.6, 50.0, 21.4, 11.7. **HRMS (ESI-MS)** *m/z* calcd for compound $C_{19}H_{26}N_3O_4$ $[M + H]^+$ 360.1918, found 360.1914.

5-(allyl(benzyl)amino)-4-morpholinocyclopent-2-en-1-one (1j)

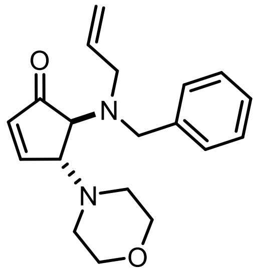

The titled compound was prepared using *N*-allyl-benzyl-amine as amine A and morpholine as amine B according to general procedure. The crude mixture was purified by flash chromatography using hexane:ethyl acetate (9.5:0.5 to 8:2) affording 195 mg (60%) of pure product as a yellow oil.

^{1}H NMR (300 MHz, CDCl$_3$) δ 7.51 (dd, J = 6.2, 2.1 Hz, 1H), 7.40 – 7.20 (m, 5H), 6.22 (dd, J = 6.2, 1.9 Hz, 1H), 6.02 – 5.74 (m, 1H), 5.41 – 4.87 (m, 2H), 3.95 – 3.78 (m, 1H), 3.67 (m, 6H), 3.46 (dd, J = 14.0, 7.9 Hz, 1H), 3.28 (d, J = 6.0, 1H), 3.19 (dd, J = 13.9, 5.0 Hz, 1H), 2.54 (t, J = 4.6 Hz, 4H). **^{13}C NMR (75 MHz, CDCl$_3$)** δ 208.2, 160.9, 136.5, 135.4, 129.1, 128.5, 128.3, 127.3, 68.8, 67.2, 63.3, 54.9, 53.3, 50.4. **HRMS (ESI-MS)** *m/z* calcd for compound $C_{19}H_{25}N_2O_2$ [M + H]$^+$ 313.1911, found 313.1903.

5-(allyl(4-methoxybenzyl)amino)-4-morpholinocyclopent-2-en-1-one (1k)

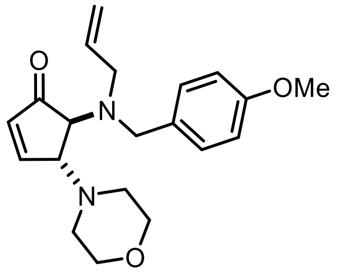

The titled compound was prepared using *N*-allyl-4(OMe)-benzyl-amine as amine A and morpholine as amine B according to general procedure. The crude mixture was purified by flash chromatography using hexane:ethyl acetate (9.5:0.5 to 8:2) affording 199 mg (56%) of pure product as a yellow oil.

^{1}H NMR (300 MHz, CDCl$_3$) δ 7.43 (dd, J = 6.2, 2.0 Hz, 1H), 7.20 (d, J = 8.6 Hz, 2H), 6.77 (d, J = 8.6 Hz, 2H), 6.13 (dd, J = 6.3, 1.8 Hz, 1H), 5.78 (dddd, J = 17.2, 10.1, 7.8, 5.1 Hz, 1H), 5.25 – 5.02 (m, 2H), 3.71 (s, 3H), 3.63 – 3.55 (m, 6H), 3.35 (dd, J = 13.9, 7.8 Hz, 1H), 3.11 (dd, J = 14.0, 5.1 Hz, 1H), 2.47 (t, J = 4.6 Hz, 4H). **^{13}C NMR (75 MHz, CDCl$_3$)** δ 208.1, 160.8, 158.8, 136.5, 135.4, 131.3, 130.2, 118.0, 113.7, 68. 7, 67.2, 63.3, 55.3, 54.7, 54.4, 50.3. **HRMS (ESI-MS)** *m/z* calcd for compound $C_{20}H_{27}N_2O_3$ [M + H]$^+$ 343.2016, found 343.2015.

5-(allyl(4-nitrobenzyl)amino)-4-morpholinocyclopent-2-en-1-one (1l)

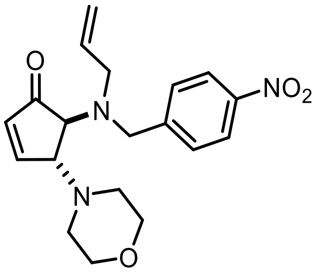

The titled compound was prepared using *N*-allyl-4(NO_2)-benzyl-amine as amine A and morpholine as amine B according to general procedure. The crude mixture was purified by flash chromatography using hexane:ethyl acetate (9.5:0.5 to 8:2) affording 219 mg (59%) of pure product as a yellow oil.

^{1}H NMR (300 MHz, $CDCl_3$) δ 8.11 (d, J = 8.7 Hz, 2H), 7.50 (d, J = 8.5 Hz, 3H), 6.18 (dd, J = 6.2, 1.8 Hz, 1H), 5.85 – 5.71 (m, 1H), 5.32 – 4.83 (m, 2H), 3.89 (d, J = 14.9 Hz, 1H), 3.69 (d, J = 11.3 Hz, 1H), 3.63 (m, 4H), 3.55 (d, J = 3.3 Hz, 1H), 3.40 (dd, J = 14.0, 7.6 Hz, 1H), 3.13 (dd, J = 14.0, 5.4, 1H), 2.53 (t, J = 4.6 Hz, 4H). **^{13}C NMR (75 MHz, $CDCl_3$)** δ 207.2, 160.9, 147.5, 147.3, 135.8, 135.5, 129.3, 123.7, 118.8, 68.7, 67.2, 64.0, 55.2, 54.7, 50.3. **HRMS (ESI-MS)** *m/z* calcd For compound $C_{19}H_{24}N_3O_4$ [M + H]$^+$ 358.1761, found 358.1749.

5-(benzyl(phenyl)amino)-4-morpholinocyclopent-2-en-1-one (1m)

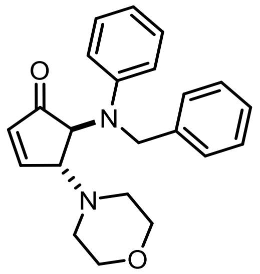

The titled compound was prepared using *N*-benzylaniline as amine A and morpholine as amine B according to general procedure. The crude mixture was purified by flash chromatography using hexane:ethyl acetate (9.5:0.5 to 8:2) affording 50 mg (14%) of pure product as a yellow oil.

^{1}H NMR (300 MHz, $CDCl_3$) δ 7.63 (dd, J = 6.4, 2.0 Hz, 1H), 7.50 – 7.39 (m, 2H), 7.32 (dd, J = 8.2, 6.4 Hz, 2H), 7.16 (dd, J = 8.8, 7.3 Hz, 2H), 6.89 – 6.69 (m, 1H), 6.66 (dd, J = 8.9, 1.0 Hz, 2H), 6.42 (dd, J = 6.3, 1.9 Hz, 1H), 4.65 (d, J = 17.0 Hz, 1H), 4.41 (d, J = 3.6 Hz, 1H), 4.32 (d, J = 17.0 Hz, 1H), 4.09 (m, 1H), 3.69 (m, 4H), 2.76 – 2.43 (m, 4H). **^{13}C NMR (75 MHz, $CDCl_3$)** δ 204.1, 160.1, 148.0, 138.6, 135.1, 129.3, 128.6, 127.2, 127.0, 118.4, 114.4, 67.5, 67.1, 65.6, 54.7, 49.9. **HRMS (ESI-MS)** *m/z* calcd for compound $C_{22}H_{25}N_2O_2$ [M + H]$^+$ 349.1910, found 349.1903.

5-(benzyl(4-methoxyphenyl)amino)-4-morpholinocyclopent-2-en-1-one (1n)

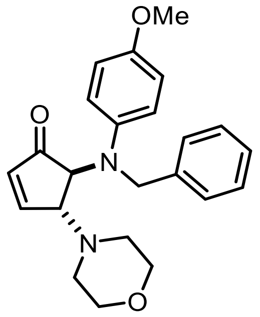

The titled compound was prepared using *N*-benzyl-4-methoxyaniline as amine A and morpholine as amine B according to general procedure. The crude mixture was purified by flash chromatography using hexane:ethyl acetate (9.5:0.5 to 8:2) affording 161 mg (41%) of pure product as a brown oil.

^{1}H NMR (300 MHz, $CDCl_3$) δ 7.58 (dd, J = 6.3, 2.0 Hz, 1H), 7.49 – 7.42 (m, 2H), 7.37 – 7.28 (m, 2H), 7.28 – 7.21 (m, 1H), 6.75 (s, 4H), 6.36 (dd, J = 6.3, 1.8 Hz, 1H), 4.58 (d, J = 16.2 Hz, 1H), 4.29 (m, 2H), 4.01 (m, 1H), 3.71 (s, 7H), 2.87 – 2.40 (m, 4H). **^{13}C NMR (75 MHz, $CDCl_3$)** δ 205.1, 160.4, 153.0, 142.3, 138.9, 134.8, 128.4, 127.4, 127.0, 117.7, 114.5, 67.6, 67.1, 66.1, 55.4, 55.1, 49.9. **HRMS (ESI-MS)** *m/z* calcd for compound $C_{23}H_{27}N_2O_3$ $[M + H]^+$ 379.2016, found 379.2009.

5-(benzyl(4-methoxyphenyl)amino)-4-morpholinocyclopent-2-en-1-one (1n')

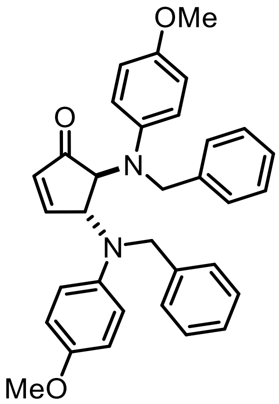

The titled compound was prepared using 1 equiv of furfural and 2 equiv of *N*-benzyl-4-methoxyaniline according to general procedure. The crude mixture was purified solely by extraction affording 520 mg (99%) of pure product as a yellow oil.

^{1}H NMR (300 MHz, $CDCl_3$) δ 7.47 (dd, J = 6.3, 2.0 Hz, 1H), 7.28 – 7.11 (m, 14H), 6.67 – 6.60 (m, 7H), 6.53 – 6.47 (m, 3H), 6.25 (dd, J = 6.3, 2.0 Hz, 1H), 5.04 (m, 1H), 4.41 (d, J = 15.9 Hz, 1H), 4.15 (m, 3H), 4.04 (d, J = 3.7 Hz, 1H), 3.64 (m, 6H). **^{13}C NMR (75 MHz, $CDCl_3$)** δ 204.1, 161.2, 153.6, 153.3, 141.9, 138.9, 138.8, 134.6, 128.6, 128.5, 127.7, 127.6, 127.08, 127.06, 118.7, 117.9, 115.0, 114.6, 114.5, 114.1, 70.1, 63.4, 56.2, 55.6, 52.7, 49.3.

General Procedure for the Preparation of 2-amino-4-thio cyclopentenones

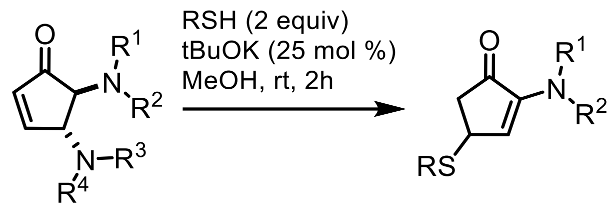

The mixed CP (0.2 mmol, 1 equiv) was dissolved in MeOH (2 mL) Then, was added 2 equivalents of the corresponding substituted thiol (0.4 mmol) and KO^{t}Bu (5.6 mg, 0.25 equiv, 0.05 mmol). The mixture was stirred at room temperature under nitrogen atmosphere for 2h. Afterwards, the crude was filtered through a short plug of celite and the filter cake was washed with DCM (6 mL). To the filtrate was added AcOH/NaOAc buffer solution at pH 5 (2 mL) and brine (4 mL). The organic layer was separated, and the aqueous layer was further extracted with DCM (2×6 mL), and the combined organic layers were dried with anhydrous $MgSO_4$, filtered and concentrated under reduced pressure. The crude mixture was further purified by column chromatography.

2-(dibenzylamino)-4-(p-methoxythio)cyclopent-2-en-1-one (2a)

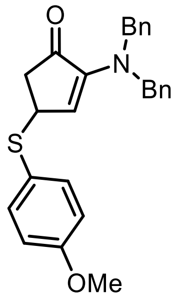

The titled compound was prepared according to general procedure. The crude mixture was purified by flash chromatography using hexane:ethyl acetate (9.5:0.5 to 8:2) affording 61 mg (73%) of pure product as a brown oil.

^{1}H NMR (300 MHz, CDCl$_3$) δ 7.38 – 7.04 (m, 12H), 6.79 (d, J = 8.8 Hz, 2H), 5.90 (d, J = 3.3 Hz, 1H), 4.42 (d, J = 4.2 Hz, 4H), 4.06 (ddd, J = 5.3, 3.2, 1.8 Hz, 1H), 3.81 (s, 3H), 2.86 (dd, J = 19.2, 6.3 Hz, 1H), 2.48 (dd, J = 19.1, 1.8 Hz, 1H). **^{13}C NMR (75 MHz, CDCl$_3$)** δ 202.3, 160.1, 148.0, 138.0, 136.9, 132.8, 128.6, 127.8, 127.2, 114.8, 114.6, 55.5, 53.3, 43.8, 43.5. **HRMS (ESI-MS)** *m/z* calcd for compound $C_{26}H_{26}NO_2S$ [M + H]$^+$ 416.1679, found 416.1670.

2-(benzyl(propyl)amino)-4-((4-methoxyphenyl)thio)cyclopent-2-en-1-one (2b)

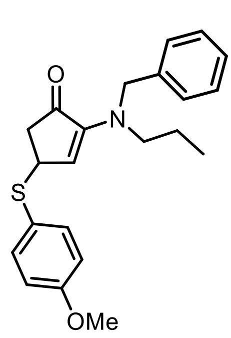

The titled compound was prepared according to general procedure. The crude mixture was purified by flash chromatography using hexane:ethyl acetate (9.5:0.5 to 8:2) affording 45 mg (65%) of pure product as a brown oil.

^{1}H NMR (300 MHz, CDCl$_3$) δ 7.33 – 7.24 (m, 5H), 7.17 – 7.14 (m, 2H). 6.81 (d, J = 8.8 Hz, 2H), 5.85 (d, J = 3.3 Hz, 1H), 4.47 (d, J = 15.8 Hz, 1H), 4.39 (d, J = 15.8 Hz, 1H), 4.11 – 4.08 (m, 1H), 3.80 (s, 3H), 3.20 – 3.15 (m, 2H), 2.82 (dd, J = 19.1, 6.2 Hz, 1H), 2.46 (dd, J = 19.1, 1.7 Hz, 1H), 1.46 (q, J = 7.8 Hz, 2H), 0.81 (t, J = 7.4 Hz, 3H). **^{13}C NMR (75 MHz, CDCl$_3$)** δ 202.2, 160.0, 147.9, 138.4, 136.6, 132.7, 128.4, 127.4, 126.9, 123.0, 114.5, 55.3, 53.5, 51.8, 43.5, 20.3, 11.3. **HRMS (ESI-MS)** *m/z* calcd for compound $C_{22}H_{26}NO_2S$ [M + H]$^+$ 368.1679, found 368.1674.

4-((4-methoxyphenyl)thio)-2-(methyl(phenyl)amino)cyclopent-2-en-1-one (2c)

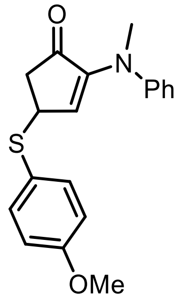

The titled compound was prepared according to general procedure. The crude mixture was purified by flash chromatography using hexane:ethyl acetate (9.5:0.5 to 8:2) affording 52 mg (80%) of pure product as a brown oil.

^{1}H NMR (300 MHz, CDCl$_3$) δ 7.41 (d, *J* = 8.9 Hz, 2H), 7.27 – 7.15 (m, 2H), 7.01 (ddt, *J* = 7.8, 6.9, 1.1 Hz, 1H), 6.87 (d, *J* = 8.7 Hz, 2H), 6.81 (dt, *J* = 7.9, 1.1 Hz, 2H), 6.49 (d, *J* = 3.2 Hz, 1H), 4.19 (ddd, *J* = 6.3, 3.2, 1.8 Hz, 1H), 3.81 (s, 3H), 3.16 (s, 3H), 2.87 (dd, *J* = 19.2, 6.4 Hz, 1H), 2.52 (dd, *J* = 19.2, 1.8 Hz, 1H). **^{13}C NMR (75 MHz, CDCl$_3$)** δ 201.3, 160.5, 149.2, 147.3, 137.0, 135.4, 129.0, 123.4, 122.6, 121.9, 114.8, 55.5, 44.0, 42.9, 40.3. **HRMS (ESI-MS)** *m/z* calcd for compound $C_{19}H_{20}NO_2S$ [M + H]$^+$ 326.1209, found 326.1205.

2-(3,4-dihydroquinolin-1(2H)-yl)-4-((4-methoxyphenyl)thio)cyclopent-2-en-1-one (2d)

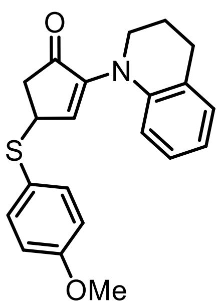

The titled compound was prepared according to general procedure. The crude mixture was purified by flash chromatography using hexane:ethyl acetate (9.5:0.5 to 8:2) affording 53 mg (75%) of pure product as a brown oil.

^{1}H NMR (300 MHz, $CDCl_3$) δ 7.40 (d, J = 8.7 Hz, 2H), 7.08 – 6.91 (m, 2H), 6.90 – 6.70 (m, 4H), 6.50 (dd, J = 8.2, 1.2 Hz, 1H), 4.20 (dq, J = 5.0, 1.6 Hz, 1H), 3.80 (s, 3H), 3.46 (m, 2H), 2.91 (dd, J = 19.2, 6.4 Hz, 1H), 2.75 (t, J = 6.5 Hz, 2H), 2.54 (dd, J = 19.2, 1.8 Hz, 1H), 1.86 (p, J = 6.2 Hz, 2H). **^{13}C NMR (75 MHz, $CDCl_3$)** δ 202.3, 160.4, 148.6, 141.8, 140.2, 137.2, 129.3, 127.1, 126.1, 122.1, 120.9, 119.3, 114.7, 55.47, 47.7, 43.9, 42.6, 27.2, 22.5. **HRMS (ESI-MS)** *m/z* calcd for compound $C_{21}H_{22}NO_2S$ $[M + H]^+$ 352.1366, found 352.1358.

2-(allyl(4-methoxybenzyl)amino)-4-((4-methoxyphenyl)thio)cyclopent-2-en-1-one (2e)

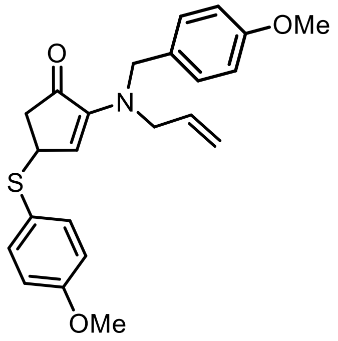

The titled compound was prepared according to general procedure. The crude mixture was purified by flash chromatography using hexane:ethyl acetate (9.5:0.5 to 8:2) affording 54 mg (68%) of pure product as a brown oil.

^{1}H NMR (300 MHz, $CDCl_3$) δ 7.23 (d, J = 8.8 Hz, 2H), 7.02 (d, J = 8.7 Hz, 2H), 6.77 (d, J = 6.5 Hz, 2H), 6.74 (d, J = 6.7 Hz, 2H), 5.87 (d, J = 3.3 Hz, 1H), 5.60 (m, 1H), 5.19 – 4.90 (m, 2H), 4.31 (d, J = 15.2 Hz, 1H), 4.22 (d, J = 15.2 Hz, 1H), 4.02 (ddd, J = 6.3, 3.3, 1.7 Hz, 1H), 3.73 (d, J = 2.0 Hz, 8H), 2.75 (dd, J = 19.1, 6.2 Hz, 1H), 2.39 (dd, J = 19.1, 1.7 Hz, 1H). **^{13}C NMR (75 MHz, $CDCl_3$)** δ 202.3, 160.2, 158.9, 148.1, 136.8, 133.8, 130.2, 129.1, 128.0, 123.0, 117.2, 114.6, 113.9, 55.45, 55.40, 52.3, 51.9, 43.9, 43.5. **HRMS (ESI-MS)** *m/z* calcd for compound $C_{23}H_{26}NO_3S$ $[M + H]^+$ 396.1628, found 396.1616.

2-(dibenzylamino)-4-(phenylthio)cyclopent-2-en-1-one (2f)

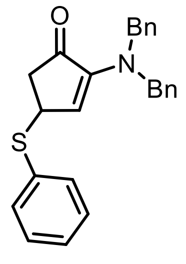

The titled compound was prepared according to general procedure. The crude mixture was purified by flash chromatography using hexane:ethyl acetate (9.5:0.5 to 8:2) affording 53 mg (69%) of pure product as a brown oil.

^{1}H NMR (300 MHz, CDCl$_3$) δ 7.41 – 7.23 (m, 11H), 7.20 – 6.99 (m, 4H), 5.92 (d, *J* = 3.2 Hz, 1H), 4.44 (s, 4H), 4.24 – 4.20 (m, 1H), 2.93 (dd, *J* = 19.2, 6.2 Hz, 1H), 2.54 (dd, *J* = 19.1, 1.7 Hz, 1H). **^{13}C NMR (75 MHz, CDCl$_3$)** δ 202.1, 148.2, 137.9, 133.3, 129.1, 128.6, 127.96, 127.86, 127.8, 127.3, 53.3, 43.9, 43.1. **HRMS (ESI-MS)** *m/z* calcd for compound $C_{25}H_{24}NOS$ [M + H]$^+$ 386.1573, found 386.1566.

2-(dibenzylamino)-4-(p-chlorothio)cyclopent-2-en-1-one (2g)

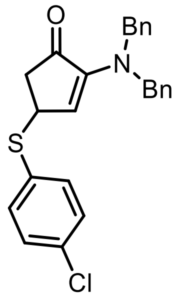

The titled compound was prepared according to general procedure using 5 equiv of *p*-chloro-thiophenol. The crude mixture was purified by flash chromatography using hexane:ethyl acetate (9.5:0.5 to 8:2) affording 17 mg (20%) of pure product as a brown oil.

^{1}H NMR (300 MHz, CDCl$_3$) δ 7.46 – 6.79 (m, 14H), 5.80 (d, *J* = 3.3 Hz, 1H), 4.65 – 4.22 (m, 4H), 4.11 (ddd, *J* = 6.3, 3.3, 1.7 Hz, 1H), 2.86 (dd, *J* = 19.1, 6.3 Hz, 1H), 2.41 (dd, *J* = 19.1, 1.7 Hz, 1H). **^{13}C NMR (75 MHz, CDCl$_3$)** δ 202.2, 148.3, 138.2, 133.6, 133.5, 129.2, 128.8, 128.1, 127.9, 127.4, 53.4, 44.0, 43.2 and **HRMS (ESI-MS)** *m/z* calcd for compound $C_{25}H_{23}ClNOS$ [M + H]$^+$ 420.1183, found 420.1191.

2-(dibenzylamino)-4-(propylthio)cyclopent-2-en-1-one (2h)

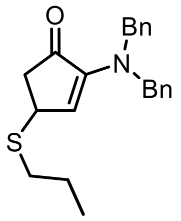

The titled compound was prepared according to general procedure. The crude mixture was purified by flash chromatography using hexane:ethyl acetate (9.5:0.5 to 8:2) affording 49.5 mg (70%) of pure product as a brown oil.

^{1}H NMR (300 MHz, CDCl$_3$) δ 7.42 – 7.08 (m, 10H), 5.90 (d, J = 3.3 Hz, 1H), 4.46 (d, J = 2.2 Hz, 4H), 3.85 (ddd, J = 6.3, 3.3, 1.8 Hz, 1H), 2.94 (dd, J = 19.2, 6.3 Hz, 1H), 2.50 (dd, J = 19.2, 1.8 Hz, 1H), 2.31 (q, J = 7.2 Hz, 2H), 1.70 – 1.36 (m, 2H), 0.93 (t, J = 7.3 Hz, 3H). **^{13}C NMR (75 MHz, CDCl$_3$)** δ 202.7, 147.9, 137.8, 129.9, 128.5, 127.7, 127.1, 53.3, 44.7, 39.3, 31.8, 23.1, 13.6. **HRMS (ESI-MS)** *m/z* calcd for compound $C_{22}H_{26}NOS$ [M + H]$^+$ 352.1730, found 352.1722.

4-(benzylthio)-2-(dibenzylamino)cyclopent-2-en-1-one (2i)

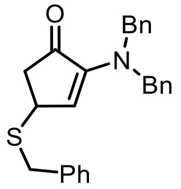

The titled compound was prepared according to general procedure. The crude mixture was purified by flash chromatography using hexane:ethyl acetate (9.5:0.5 to 8:2) affording 69 mg (85%) of pure product as a brown oil.

^{1}H NMR (300 MHz, CDCl$_3$) δ 7.65 – 6.98 (m, 15H), 5.90 (d, J = 3.2 Hz, 1H), 4.51 (s, 4H), 3.84 (m, 1H), 3.66 (m, 2H), 2.91 (dd, J = 19.2, 6.2 Hz, 1H), 2.51 (dd, J = 19.0, 1.8 Hz, 1H). **^{13}C NMR (75 MHz, CDCl$_3$)** δ 202.1, 147.6, 137.9, 137.5, 128.7, 128.5, 128.3, 128.2, 127.5, 126.9, 126.8, 52.9, 44.0, 39.0, 34.8. **HRMS (ESI-MS)** *m/z* calcd for compound $C_{26}H_{26}NOS$ [M + H]$^+$ 400.1730, found 400.1724.

2-(dibenzylamino)-4-methoxycyclopent-2-en-1-one (S1)

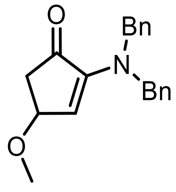

The titled compound was prepared according to general procedure without the use of any thiol and using 1 equiv of NaOMe as base. The crude mixture was purified by flash chromatography using hexane:ethyl acetate (9.5:0.5 to 8:2) affording 33 mg (54%) of pure product as a brown oil.

^{1}H NMR (300 MHz, CDCl$_3$) δ 7.56 – 7.00 (m, 10H), 5.95 (d, *J* = 3.1 Hz, 1H), 4.53 (d, *J* = 15.7 Hz, 2H), 4.46 (d, *J* = 15.6 Hz, 2H), 4.38 (ddd, *J* = 5.7, 3.1, 1.8 Hz, 1H), 3.28 (s, 3H), 2.76 (dd, *J* = 18.3, 5.7 Hz, 1H), 2.41 (dd, *J* = 18.4, 1.8 Hz, 1H).**^{13}C NMR (75 MHz, CDCl$_3$)** δ 201.4, 149.1, 137.9, 128.6, 127.8, 127.3, 125.2, 74.2, 56.0, 53.2, 42.9. **HRMS (ESI-MS)** *m/z* calcd for compound $C_{20}H_{22}NO_2$ [M + H]$^+$ 308.1645, found 308.1651.

3-(diallylamino)-2-(dibenzylamino)-4-(propylthio)cyclopentan-1-one (S2)

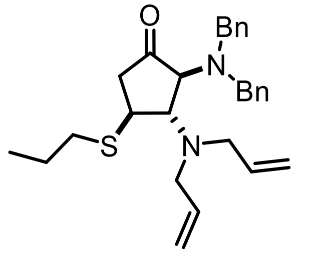

The titled compound was prepared according to general procedure in the absence of base. The crude mixture was evaporated affording 88 mg (98%) of pure product as a brown oil.

^{1}H NMR (300 MHz, $CDCl_3$) δ 7.52 – 7.34 (m, 4H), 7.33 – 7.05 (m, 6H), 6.03 – 5.59 (m, 2H), 5.39 – 4.88 (m, 4H), 3.80 (s, 4H), 3.35 – 3.50 (m, 2H), 3.08 (d, J = 6.3 Hz, 4H), 2.65 (dd, J = 18.9, 8.1, Hz, 1H), 2.50 (t, J = 7.3 Hz, 2H), 2.03 (dd, J = 18.8, 10.7 Hz, 1H), 1.68 – 1.27 (m, 2H), 0.91 (t, J = 7.3 Hz, 3H). **^{13}C NMR (75 MHz, $CDCl_3$)** δ 215.0, 139.5, 137.6, 129.3, 128.4, 127.2, 117.0, 66.6, 65.2, 54.6, 53.4, 45.5, 38.8, 33.7, 23.1, 13.6.

Attempts at protecting the enone 1c towards the total synthesis of Agelastatin A

Table S2. Protection of the enol as ketal

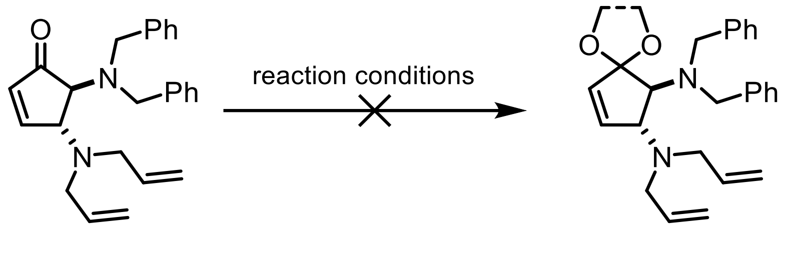

1c **S3**

Entry	Protection reagent	T (º C)	Catalyst	Solvent	**S3** (%)
1	Ethylenoglycol	RT	pTSOH	MeOH	ND
2	Ethylenoglycol	reflux	pTSOH	MeOH	ND
3	Ethylenoglycol	reflux	pTSOH	Toluene	ND
4	1,2-Bis(trimethylsiloxy)ethane	RT	TMSOTf	DCM	ND
5	1,2-Bis(trimethylsiloxy)ethane	RT	TMSOTf, 4 A MS	DCM	ND
6	$CH(OCH_3)_3$	RT	pTSOH	MeOH	ND
7	$CH(OCH_3)_3$	reflux	pTSOH	MeOH	ND
8	$CH(OCH_3)_3$	reflux	pTSOH	Toluene	ND

ND stands for not detected.

Table S3. Protection of the enol as imine/oxime

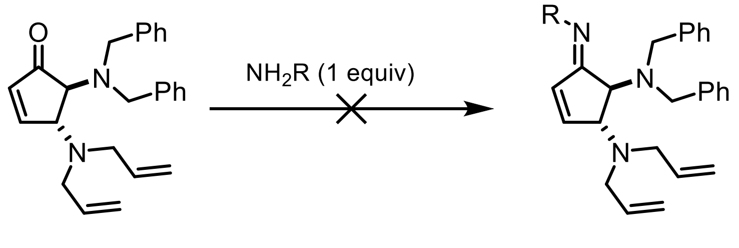

1c **S4**

Entry	Protection reagent	T (º C)	Catalyst	Solvent	**S4** (%)
	Aniline (1 equiv)	RT	none	toluene	ND
	Aniline (1 equiv)	reflux	none	toluene	ND
	$NH_2OH.HCl$ (2 equiv)	RT	NaOAc (2 equiv)	MeOH	Decomposition
	$NH_2OH.HCl$ (2 equiv)	RT	NEt_3	EtOH	Decomposition

ND stands for not detected.

Table S4. Enone 1,2 reduction

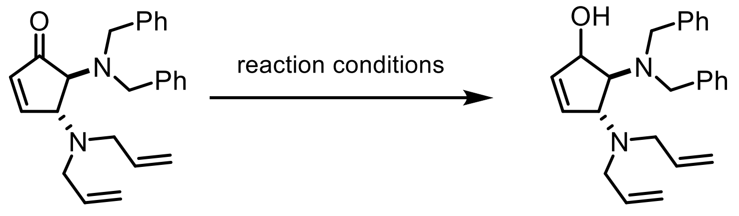

Entry	Reduction agent	T (º C)	Solvent	**S5** (%)
1	$CeCl_3 \cdot 7H_2O$, $NaBH_4$	0 to RT	MeOH	0
2	$CeCl_3 \cdot 7H_2O$, $NaBH_4$	-50 ºC	MeOH	16
3	$LiAlH_4$	0 to RT	THF	0
4	DIBAL-H	0 to RT	THF	40
5	DIBAL-H	-78 ºC	Toluene	95
6	L-Selectride	0 to RT	THF	0

Preparation of 4-(diallylamino)-5-(dibenzylamino)cyclopent-2-en-1-ol (S5)

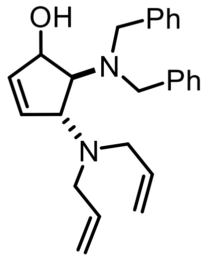

Mixed CP **1c** (2.5 g, 6.7 mmol, 1.0 eq) was dissolved in toluene (25 mL) and cooled to -78 °C. Then, 1.4 equivalent of DIBAL-H was added dropwise as a 1.0 M solution in hexanes (9.5 mL, 9.5 mmol, 1.4 equiv). The mixture was stirred for 1 hour. The reaction was quenched with water (20 mL) and diluted in a saturated aqueous Rochelle's salt (30 mL). Then the reaction mixture was extracted with ethyl acetate (3 × 10 mL). The organic layer was dried over Na_2SO_4 and concentrated under reduced pressure, affording 2.46 g (98%) of pure product as a clear oil.

^{1}H NMR (300 MHz, $CDCl_3$) δ 7.38 – 7.27 (m, 10H), 6.09 (m, 1H), 6.00 (m, 1H), 5.80 (m, 2H), 5.28 – 5.06 (m, 4H), 4.46 (d, *J* = 6.5 Hz, 1H), 4.29 (m, 1H), 4.00 (d, *J* = 13.9 Hz, 2H), 3.66 (d, *J* = 13.9 Hz, 2H), 3.28 (dd, *J* = 6.8, 4.2 Hz, 1H), 3.01 (d, *J* = 6.2 Hz, 4H). **^{13}C NMR (75 MHz, $CDCl_3$)** δ 139.2, 136.9, 136.3, 134.0, 128.8, 128.6, 127.3, 117.1, 73.8, 66.1, 62.6, 55.6, 53.9. **HRMS (ESI-MS)** *m/z* calcd for compound $C_{25}H_{31}N_2O$ [M + H]$^+$ 375.2431, found 375.2440.

Total Synthesis of (±)-Agelastatin A

Preparation of trans-5-(benzyl(phenyl)amino)-4-morpholinocyclopent-2-en-1-one (1c)

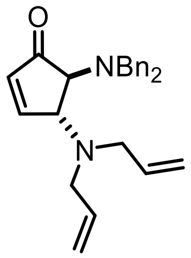

To a solution of $Cu(OTf)_2$ (80 mg, 10 mol%) in water (2 mL) was added dibenzylamine (0.2 mL, 1.04 mmol, 1 equiv.), diallylamine (0.17mL, 2.08 mmol, 2 equiv.), and furfural (0.250 mL, 2.08 mmol). The reaction was allowed to stir vigorously at room temperature for 5 minutes. Then the reaction mixture was diluted with water (18 mL) and extracted with MTBE (3 × 20 mL). The combined organic phases were dried with $MgSO_4$, the solvent was evaporated under reduced pressure and the crude mixture was purified by flash chromatography using hexane:ethyl acetate (9.5:0.5 to 8:2), affording 226 mg (59%) of pure product as a yellow oil.

Preparation of N^2,N^2-diallyl-N^1,N^1-dibenzyl-5-((tert-butyldimethylsilyl)oxy)cyclopent-3-ene-1,2-diamine (4)

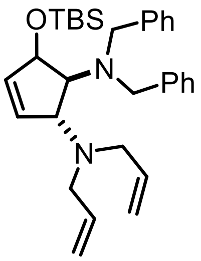

Mixed CP **1c** (2.5 g, 6.7 mmol, 1.0 eq) was dissolved in toluene (25 mL) and cooled to -78 °C. Then, 1.4 equivalent of DIBAL-H was added dropwise as a 1.0 M solution in hexanes (9.5 mL, 9.5 mmol, 1.4 equiv). The mixture was stirred for 1 hour. The reaction was quenched with water (20 mL) and diluted in a saturated aqueous Rochelle's salt (30 mL). Then the reaction mixture was extracted with ethyl acetate (3 × 30 mL). The organic layer was dried over Na_2SO_4 and concentrated under reduced pressure. The obtained oil was redissolved in CH_2Cl_2 (70 mL). To the mixture was added imidazole (913 mg, 13.4 mmol, 2.0 eq), followed by TBDMSCl (1.1 g, 7.4 mmol, 1.1 eq). The mixture was stirred for 12 hours. The reaction mixture was washed with brine (70 mL). The organic layer was dried over Na_2SO_4 and concentrated under reduced pressure. The crude was purified by flash chromatography using hexane:ethyl acetate (9.5:0.5) affording 3.02 g (92%) of pure product as a yellow oil.

^{1}H NMR (300 MHz, CDCl$_3$) δ 7.90 – 7.82 (m, 4H), 7.78 – 7.57 (m, 6H), 6.35 (M, 1H), 6.30 – 6.12 (m, 3H), 5.57 – 5.43 (m, 4H), 5.25 (m, 1H), 4.63 (m, 1H), 4.47 (d, *J* = 14.2 Hz, 2H), 4.36 (d, *J* = 14.4 Hz, 2H), 3.59 (m, 1H), 3.43 – 3.31 (m, 4H), 1.39 (s, 9H), 0.62 (s, 3H), 0.57 (s, 3H). **^{13}C NMR (75 MHz, CDCl$_3$)** δ 141.5, 137.1, 135.6, 134.6, 128. 8, 128.1, 126.7, 116.7, 77.4, 68.5, 60.7, 55.9, 53.6, 26.3, 18.2, -3.9, -4.2. **HRMS (ESI-MS)** *m/z* calcd for compound $C_{31}H_{45}N_2OSi$ [M + H]$^+$ 489.3296, found 489.3294.

Preparation of N-(4-((tert-butyldimethylsilyl)oxy)-5-(dibenzylamino)cyclopent-2-en-1-yl)-1H-pyrrole-2-carboxamide (5)

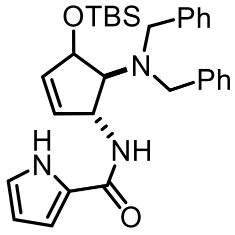

To a mixture of N,N-dimethylbarbituric acid (NMDBA) (640 mg, 4.1 mmol, 4.0 eq) and $Pd(PPh_3)_4$ (118 mg, 0.102 mmol, 10 mol%) in a schlenk tube was added a solution of **4** (500 mg, 1.02 mmol, 1.0 eq) in CH_2Cl_2 (10.0 mL). The reaction was allowed to stir at room temperature. After 1 hour a freshly prepared solution of pyrrole acyl chloride (132 mg, 1.02 mmol, 1 equiv) in CH_2Cl_2 (2 mL) was added, followed by triethylamine (575 µL, 4.09 mmol, 4 equiv). After 1 hour the reaction mixture was washed with H_2O (2 × 10 mL). The organic layer was dried over Na_2SO_4 and concentrated under reduced pressure. The crude mixture was purified by flash chromatography using hexane:ethyl acetate (8:2) affording 436 mg (85%) of pure product as a yellow oil.

^{1}H NMR (300 MHz, $CDCl_3$) δ 10.23 (s, 1H), 7.37 (m, 5H), 7.29 – 7.18 (m, 5H), 6.88 (m, 1H), 6.46 (m, 1H), 6.22 (m, 1H), 5.95 (dd, *J* = 6.2, 1.6 Hz, 1H), 5.87 (m, 1H), 5.71 (m, 1H), 5.56 – 5.48 (m, 1H), 4.78 (m, 1H), 4.03 (s, 4H), 2.97 (dd, *J* = 7.5, 5.6 Hz, 1H), 0.91 (s, 9H), 0.17 (s, 3H), 0.12 (s, 3H).**^{13}C NMR (75 MHz, $CDCl_3$)** δ 161.4, 140.9, 136.2, 134.3, 128.6, 128.4, 126.9, 126.1, 122.3, 109.8, 108.9, 74.9, 67.4, 55.9, 54.0, 26.1, 18.2, -3.9, -4.2. **HRMS (ESI-MS)** *m/z* calcd for compound $C_{30}H_{40}N_3O_2Si$ $[M + H]^+$ 502.2884, found 502.2888.

Preparation of N-5-(dibenzylamino)-4-oxocyclopent-2-en-1-yl)-1H-pyrrole-2-carboxamide (3)

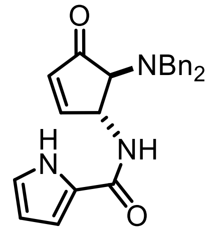

To a solution of **6** (50 mg, 0.99 mmol) in CH_2Cl_2 (1 mL) was added TBAF (78 mg, 0.29 mmol, 3 equiv), and the mixture was stirred for 12h. The crude mixture was concentrated under reduced pressure and re-dissolved in DMSO (1 mL). IBX (28 mg, 0.99 mmol, 1 equiv) was added and the reaction was allowed to stirred for 3 hours, after which was added methanol. The mixture was filtered and the crude was concentrated under reduced pressure and further purified by flash chromatography using hexane:ethyl acetate (8:2) affording 33 mg (87%) of pure product as a yellow solid.

^{1}H NMR (300 MHz, $CDCl_3$) δ 7.48 – 7.37 (m, 5H), 7.29 – 7.17 (m, 6H), 7.00 (m, 1H), 6.45 (m, 1H), 6.31 – 6.22 (m, 2H), 5.92 (m 1H), 5.45 (m, 1H), 3.89 (d, *J* = 13.5 Hz, 2H), 3.67 (d, *J* = 13.5 Hz, 2H), 3.42 (d, *J* = 3.9 Hz, 1H). Data in accordance with the literature.[1]

Preparation of N-5-(dibenzylamino)-4-oxocyclopent-2-en-1-yl)-1H-pyrrole-2-carboxamide (9)

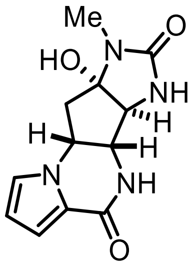

To a solution of **3** (20 mg, 0.51 mmol) in THF (0.5 mL) was added Cs_2CO_3 (17 mg, 0.51 mmol, 1 equiv) and the reaction was allowed to stir at room temperature under inert atmosphere. The reaction was monitored by TLC until full consumption of starting material. The reaction was diluted with AcOEt (3 mL) and washed with brine (4 mL). The organic layer was dried over Na_2SO_4 and concentrated under reduced pressure. The crude mixture was re-dissolved in THF (1 mL) and added to a reactor containing Pd/C (2 mg). *N*-methyl-1H-imidazole-1-carboxamide (13 mg, 1 mmol, 2 equiv) was added to the reaction mixture. The reactor was purged with nitrogen and filled with H_2 (8 bar). After 12h the reaction mixture was filtered through Celite and concentrated under reduced pressure. The crude was further purified by flash chromatography using CH_2Cl_2:MeOH (9:1) affording 8 mg (58 %) of pure product as a white solid.

^{1}H NMR (300 MHz, DMSO-*d6*) δ 7.80 (s, 1H), 7.06 (dd, *J* = 2.6, 1.6 Hz, 1H), 6.96 (d, *J* = 2.3 Hz, 1H), 6.64 (dd, *J* = 3.7, 1.6 Hz, 1H), 6.37 (s, 1H), 6.13 (dd, *J* = 3.8, 2.5 Hz, 1H), 4.51 – 4.58 (m, 1H), 3.83 (d, *J* = 5.3 Hz, 1H), 3.73 – 3.64 (m, 1H), 2.63 (s, 3H), 2.02 (dd, *J* = 12.8, 11.0 Hz, 1H). Data in accordance with the literature.[1]

Preparation of (±)-Agelastatin A

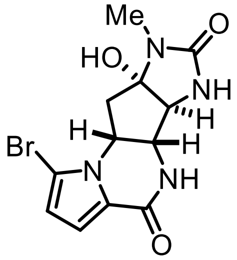

Debromoagelastatin A was brominated according to a reported procedure.[1] The reaction delivers agelastatin A contaminated with ~10% of its 13-Debromo-14-bromo-agelastatin A isomer, as observed in ^{1}H NMR (CD_3OD) of reaction crude and as previously reported by Molinsky.[2] The isomers are inseparable through standard normal phase silica column chromatography and preparative TLC. Pure (±)-Agelastatin A can be obtained through semi-preparative reversed-phase HPLC on a Dionex Ultimate 3000 system using a Phenomenex Luna® 10 µm C18(2) 100 Å, LC Column 250 x 10 mm, with a linear solvent gradient from 20–30% aqueous CH_3CN over 10 min, 5 mL min−1, UV detection at 250nm.

^{1}H NMR (300 MHz, $(CD_3)_2SO$) δ 7.99 (s, 1H), 7.09 (d, J = 2.4 Hz, 1H), 6.73 (d, J = 4.0 Hz, 1H), 6.53 s, , 1H), 6.34 (d, J = 4.0 Hz, 1H), 4.40 – 4.32 (m, 1H), 3.95 (d, J = 5.5 Hz, 1H), 3.76 (d, J = 2.3 H, 1H), 2.64 (s, 3H), 2.47 – 2.42 (m, 1H), 1.92 (t, J = 12.4 Hz, 1H). **^{13}C NMR (300 MHz, $(CD_3)_2SO$)** δ 158.6,

157.7, 123.7, 113.3, 111.9, 104.5, 93.4, 65.1, 60.3, 52.5, 38.9, 23.5. **HRMS (ESI-MS)** *m/z* calcd for compound $C_{12}H_{14}BrN_4O_3$ $[M + H]^+$ 341.0244, found 341.0243.

Table S4. Spectroscopic comparison between our synthetic Agelastatin A, reported isolated Agelastatin A and reported synthetic Agelastatin A.

This work (in $(CD_3)_2SO$)	***J. Chem. Soc., Chem. Commun.***, 1993, 1305-1306 (in $(CD_3)_2SO$) **(Isolation)**	**Chem. Commun.**, 2018, *54*, 9893-9896 (in CD_3OD) **(Preparation)**	***J. Chem. Soc., Chem. Commun.***, 1993, 1305-1306 (in CD_3OD) **(Isolation)**
	**^{1}H NMR**		
7.99 (s, 1H)	8.02 (s, 1H)	-	-
7.09 (d, J = 2.4 Hz, 1H)	7.11 (d, J = 2.1 Hz, 1H)	-	-
6.73 (d, J = 4.0 Hz, 1H)	6.74 (d, J = 4.2 Hz, 1H)	6.90 (d, 1H, J = 4.6 Hz)	6.92 (br d, 1H)
6.34 (d, J = 4.0 Hz, 1H)	6.34 (d, J = 4.2 Hz, 1H)	6.32 (d, 1H, J = 4.0 Hz)	6.33 (d, J = 4.2, 1H)
4.40 – 4.32 (m, 1H)	4.36 (m, J = 12.0, 6.0, 5.4 Hz, 1H)	4.59 (ddd, 1H, J = 12.0, 6.3, 5.7 Hz)	4.60 (m, 1H)
3.95 (d, J = 5.5 Hz, 1H)	3.96 (br d, J = 5.4 Hz, 1H)	4.08 (d, 1H, J = 5.2 Hz)	4.09 (br. D, 1H)
3.76 (d, J = 2.3 Hz, 1H)	3.77 (br d, J = 2.1 Hz, 1H)	3.87 (s, 1H)	3.89 (br s, 1H)
2.64 (s, 3H)	2.64 (s, 3H)	2.80 (s, 3H)	2.81 (s, 3H)
2.47 – 2.42 (m, 1H)	2.46 (br dd, J = 12.6, 6.0 Hz, 1H)	2.64 (dd, 1H, J = 13.2, 6.3 Hz)	2.65 (br dd, 1H)
1.92 (t, J = 12.4 Hz, 1H)	1.92 (br t, J = 12.6, 12 Hz, 1H)	2.09 (dd, 1H, J = 12.6, 12.6 Hz)	2.10 (br t, 1H)
	**^{13}C NMR**		
158.6	158.6	161.4	163.0
157.7	157.7	161.1	162.7
123.7	123.5	124.1	125.7
113.3	113.4	116.0	117.6
111.9	111.8	113.8	115.4
104.5	104.5	107.3	108.8
93.4	93.3	95.7	97.2
65.1	65.0	67.4	68.9
60.3	60.2	62.2	63.8
52.5	52.5	54.4	55.9
38.9	38.9	40.0	41.6
23.5	23.6	24.2	25.8

Reaction mixture and purified (±)-Agelastatin A were analysed in reversed-phase HPLC with a Nucleodur® 100-5 C18ec column with a linear solvent gradient from 20−40% aqueous CH_3CN over 20 min, 1 mL min−1, UV detection at 250nm.

LC-HRMS of pure (±)-Agelastatin A was realized using a Dionex Ultimate 3000 UHPLC+ system equipped with a Multiple-Wavelength detector, using an imChem Surf C18 TriF 100A 3 µm 100x2,1mm column, linear solvent gradient from 20−30% aqueous CH_3CN over 10 min, 0.2 mL min−1, UV detection at 250nm, connected to Thermo Scientific Q Exactive hybrid quadrupole-Orbitrap mass spectrometer (Thermo ScientificTM Q ExactiveTM Plus).

^{1}H NMR (300 MHz) in CD_3OD of reaction crude:

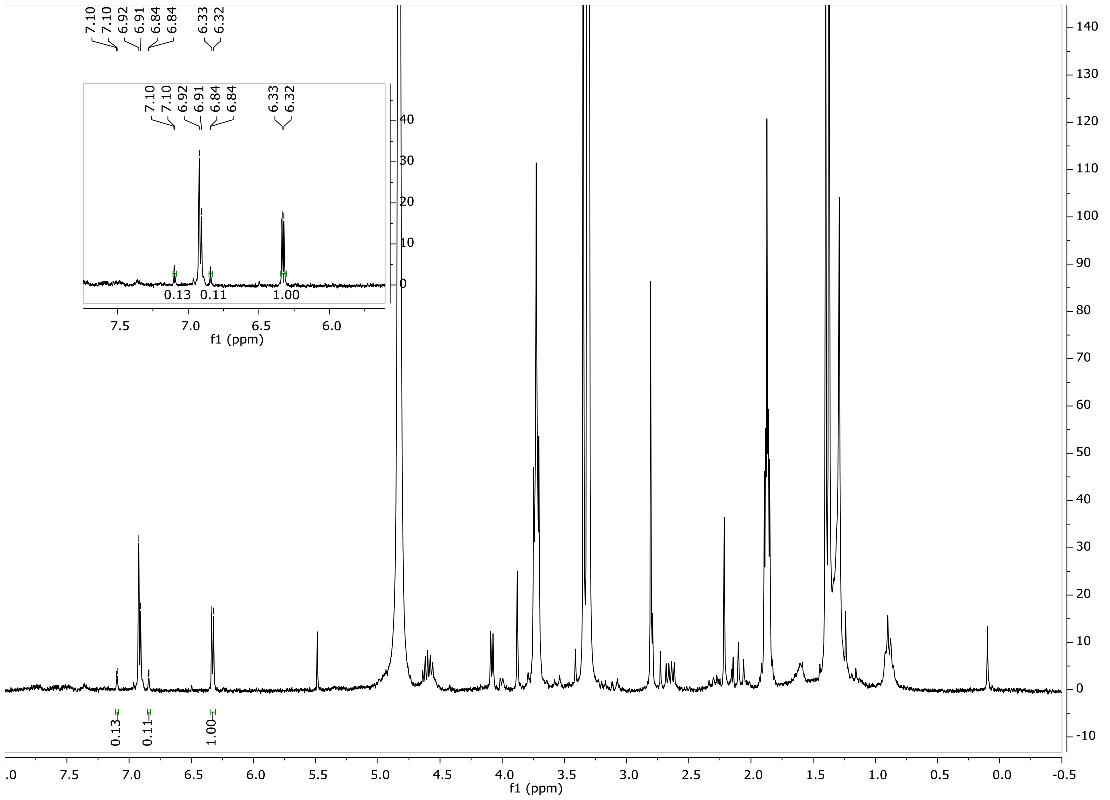

7.10
7.10
6.92
6.91
6.84
6.84
6.33
6.32
0.13
0.11
1.00
f1 (ppm)

HPLC chromatogram of reaction crude:

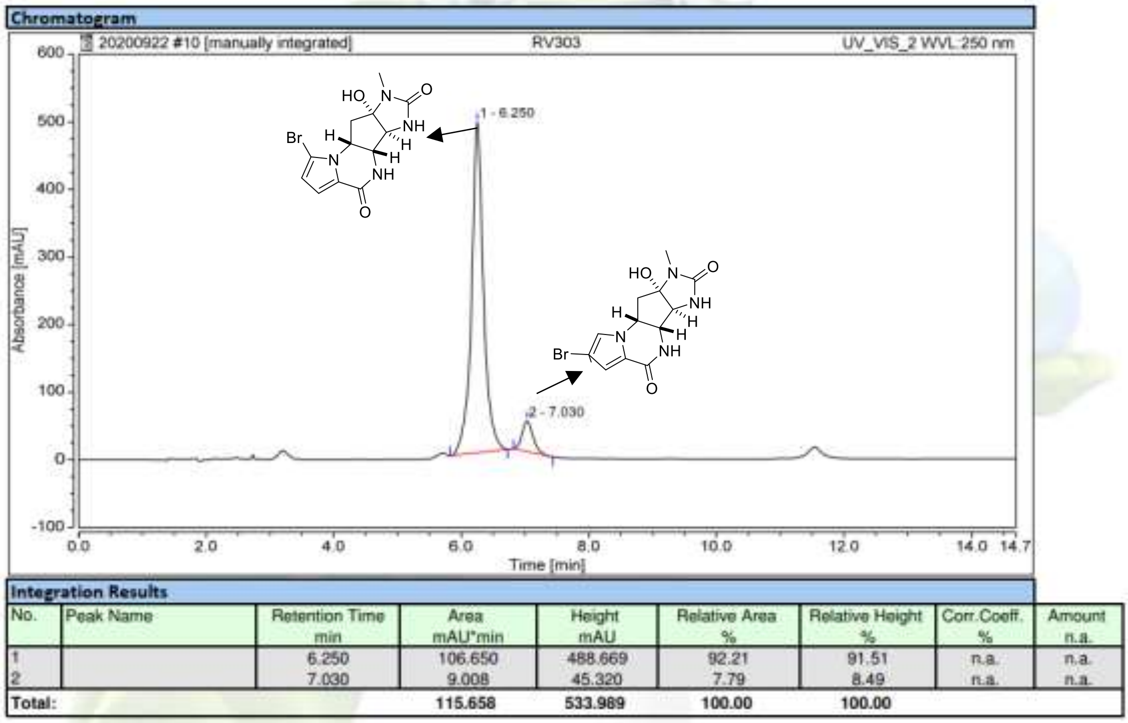

Integration Results								
No.	Peak Name	Retention Time min	Area mAU*min	Height mAU	Relative Area %	Relative Height %	Corr.Coeff. %	Amount n.a.
1		6.250	106.650	488.669	92.21	91.51	n.a.	n.a.
2		7.030	9.008	45.320	7.79	8.49	n.a.	n.a.
Total:			115.658	533.989	100.00	100.00		

HPLC chromatogram of (±)-Agelastatin A after purification through semi-preparative HPLC:

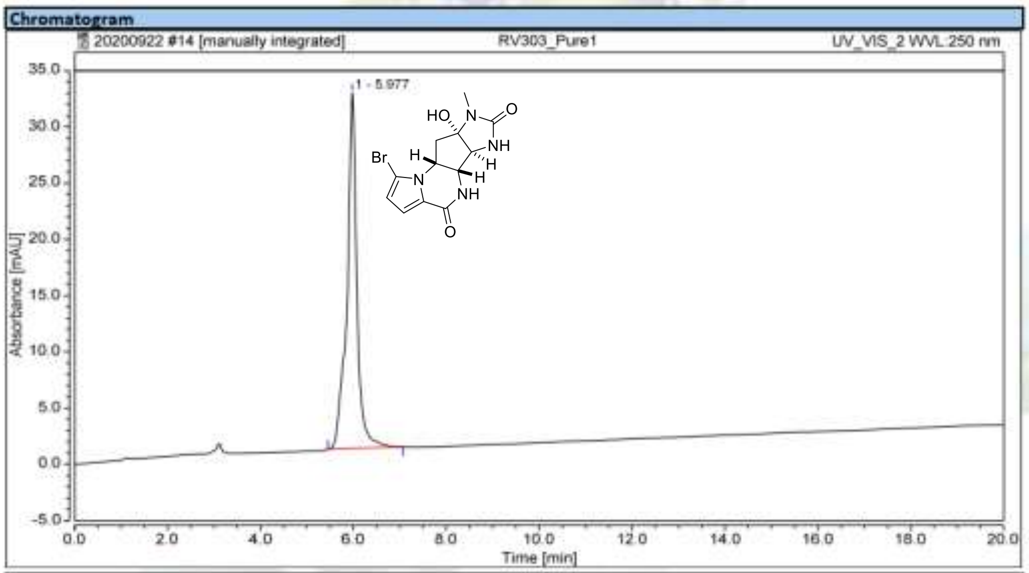

Integration Results								
No.	Peak Name	Retention Time min	Area mAU*min	Height mAU	Relative Area %	Relative Height %	Corr.Coeff. %	Amount n.a.
1		5.977	7.827	31.585	100.00	100.00	n.a.	n.a.
Total:			7.827	31.585	100.00	100.00		

LC-HRMS analysis of pure (±)-Agelastatin A:

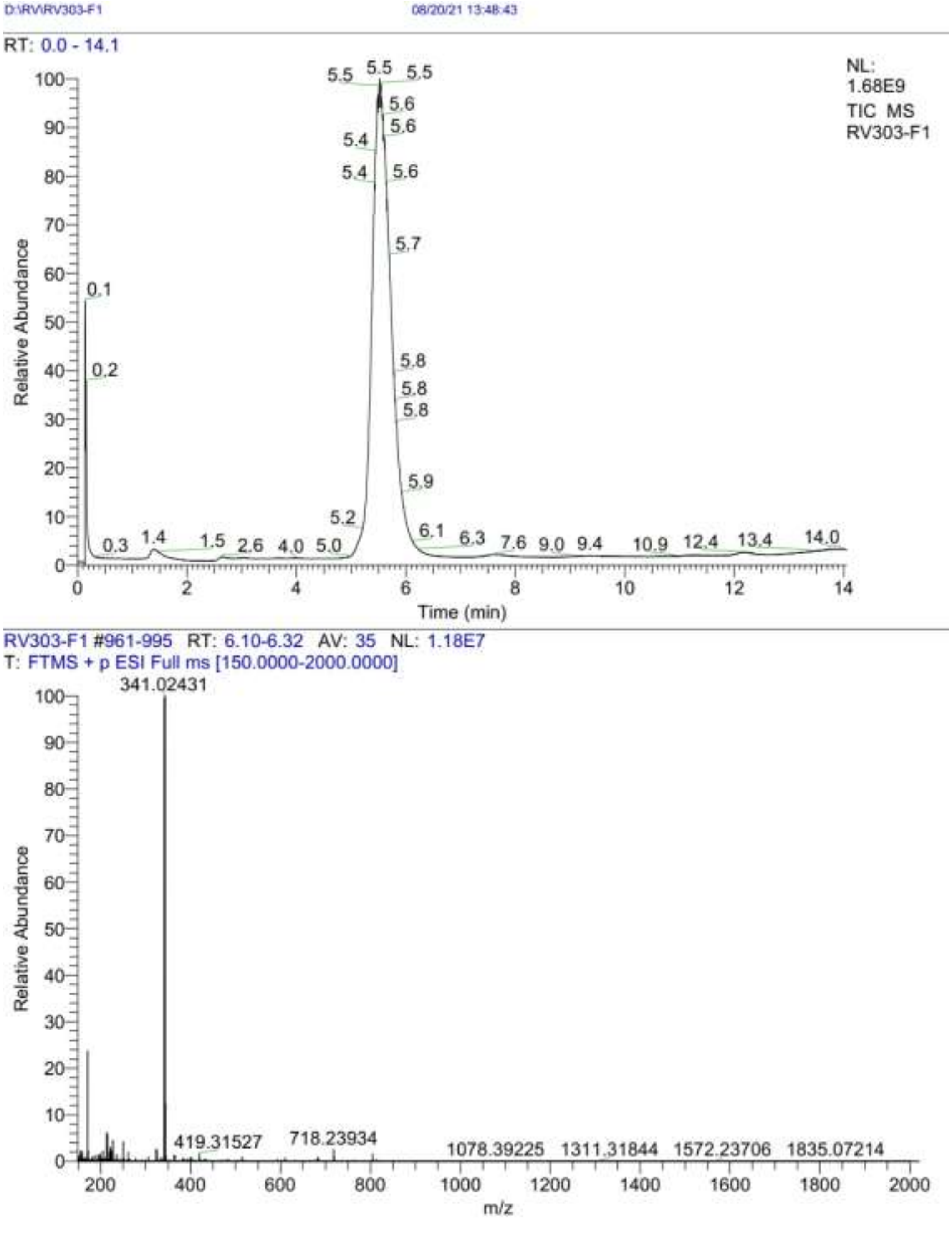

Enantioselective attempts:

Firstly enantiomeric attempts at obtaining the desired *trans*-4,5-diamino-cyclopent-2-enones focused on the use of chiral ligands as depicted in Table S5. Unfortunately no ee was observed using a variety of pybox and box ligands in different solvents. Additionally, the reactions were often incomplete. Also, due to the reversible nature of the CP systems, in several cases the chromatogram exhibits a furfural peak, despite being pure by ^{1}H-NMR.

Table S5. Attempts towards asymmetric variant using BOX-catalysis.[a]

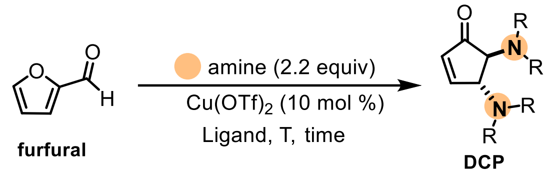

Entry	Time (min)	amine	Solvent	Ligand	e.e.
1	30	Morpholine (DCP 1)	MeCN	L1 (30 mol%)	0
2	5	Morpholine (DCP 1)	MeCN	L1 (30 mol%)	-[b]
3	5	Morpholine (DCP 1)	HFIP/DCM (1:4)	L1 (20 mol%)	0
4	30	Morpholine (DCP 1)	MeCN	L2 (20 mol%)	0
5	30	Morpholine (DCP 1)	HFIP	L5 (20 mol%)	0
6	20	Morpholine (DCP 1)	HFIP/DCM (1:4)	L5 (20 mol%)	0
7	5	Morpholine (DCP 1)	DCM	L5 (20 mol%)	0
8	30	Morpholine (DCP 1)	MeCN	L5 (20 mol%)	0
9	15	THQ (DCP 2)	DCM	L2 (30 mol%)	-[b]
10	15	THQ (DCP 2)	DCM	L2 (60 mol%)	-[b]
11	120	THQ (DCP 2)	DCM	L2 (30 mol%)	0
12	120 (-40 °C)	THQ (DCP 2)	DCM	L3 (30 mol%)	0
13	30	THQ (DCP 2)	MeCN	L2 (20 mol%)	0
14	60	THQ (DCP 2)	MeCN	L4 (20 mol%)	0
15	25	DBA (DCP 3)	ACN	L1 (30 mol%)	0[b]
16	25	DBA (DCP 3)	ACN	L4 (30 mol%)	0[b]

[a] furfural (20 mg, 0.2 mmol), amine (2 equiv.), Ligand (x equiv), anhydrous solvent (0.2 mL) The ligand was stirred with the metal for 18h prior to the reaction. [b]No product was observed.

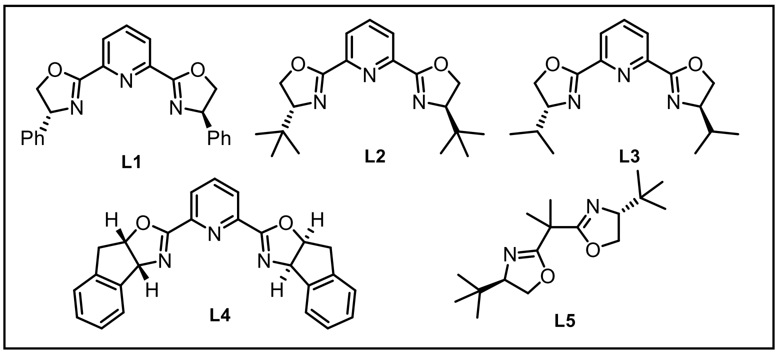

A second approach was the use of chiral acids to induce enantiomeric resolution by crystallization as depicted in Table S6. The selected acids were (+)-Tartaric acid, (-)-Mandelic acid and (+)-Camphorsulfonic acid. To this end, a solution of **DCP 1** (20 mg/mL) was prepared and a solution of the corresponding acid (0.5 equiv) was added dropwise and left to crystalize. The crystals were removed, redissolved in acetonitrile and the e.e. was evaluated by HPLC.

Table S6. Attempts at enantiomeric resolution by crystalization.

Entry	solvent	acid	e.e.
1	MTBE:EtOH	(+)-Tartaric acid	0
2	MTBE:EtOH	(-)-Mandelic acid	0
3	MTBE:EtOH	(+)-Camphorsulfonic acid	0
4	Acetone	(+)-Tartaric acid	0
5	Acetone	(-)-Mandelic acid	0
6	Acetone	(+)-Camphorsulfonic acid	0
7	EtOH	(+)-Tartaric acid	0
8	EtOH	(-)-Mandelic acid	0
9	EtOH	(+)-Camphorsulfonic acid	0
10	MTBE	(+)-Tartaric acid	0
11	MTBE	(-)-Mandelic acid	0
12	MTBE	(+)-Camphorsulfonic acid	0
13	EtOAc	(+)-Tartaric acid	0
14	EtOAc	(-)-Mandelic acid	0
15	EtOAc	(+)-Camphorsulfonic acid	0

Additionally the reaction was performed with chiral amines (Scheme S1), namely proline and the corresponding methyl ester, α-substituted morpholine (gratefully supplied by Jeffrey W. Bode, ETH) and a non-chiral α-substituted alkyl amine. However we observed no reaction with these amines, possibly due to steric hindrance.

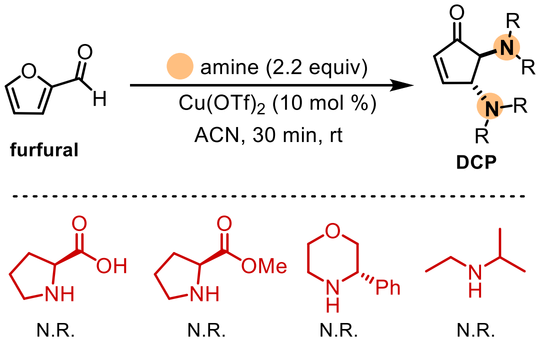

Scheme S1. Scope of chiral amines.

HPLC-DAD analysis of racemic **DCP 1**. Method: IC column, Isocratic eluent ACN:IPA (95:5), Flow 1mL/min:

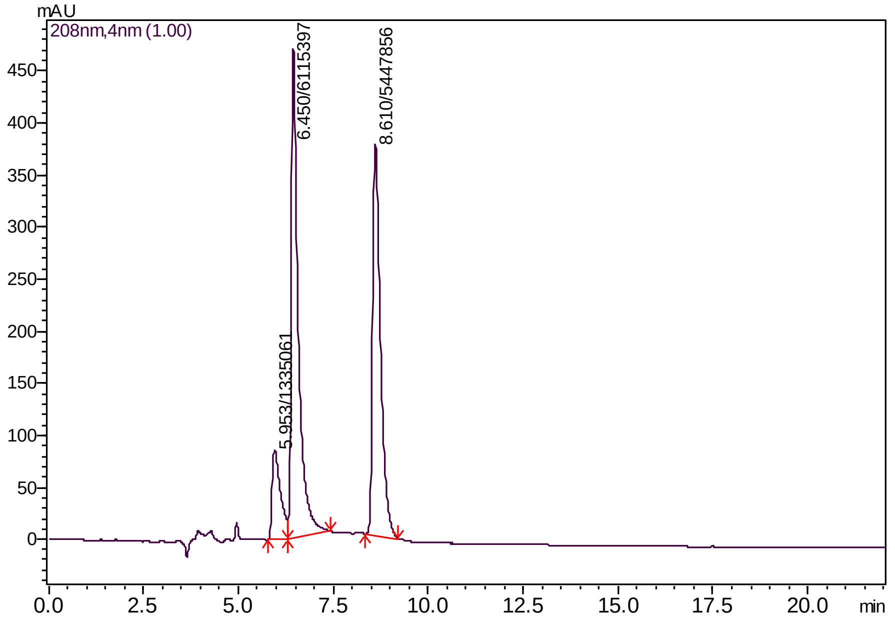

Selected HPLC-DAD analysis of crude mixture from attempts at enantioselective reaction towards DCP 1 (Entry 1, Table S1) Method: IC column, Isocratic eluent ACN:IPA (95:5), Flow 1mL/min:

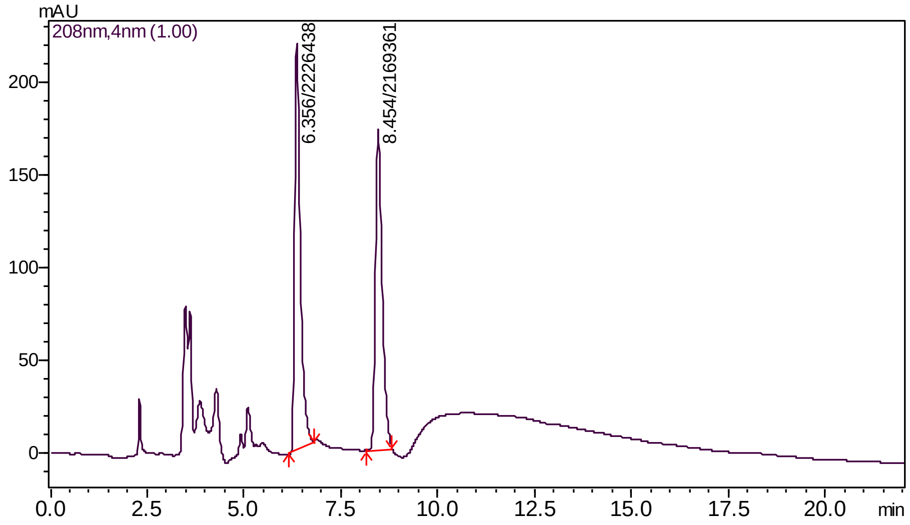

HPLC-DAD analysis of racemic **DCP 2**. Method: IC column, Isocratic eluent Hex:IPA (82:18), Flow 1mL/min:

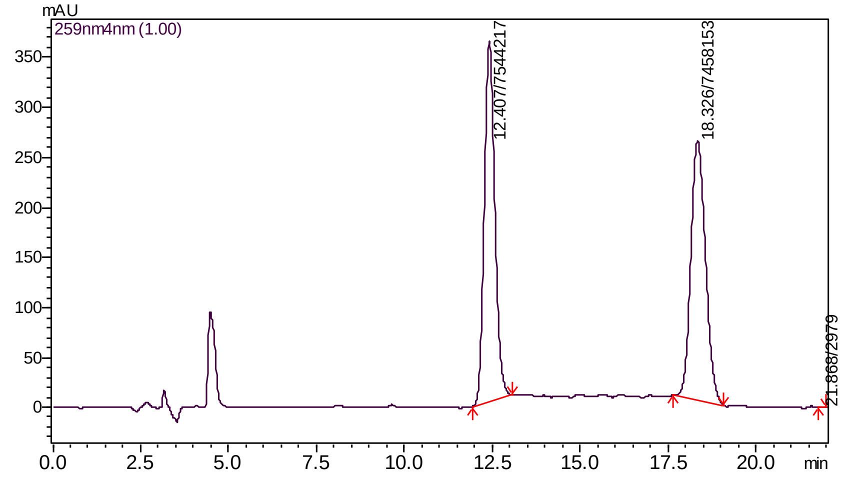

HPLC-DAD analysis of furfural.

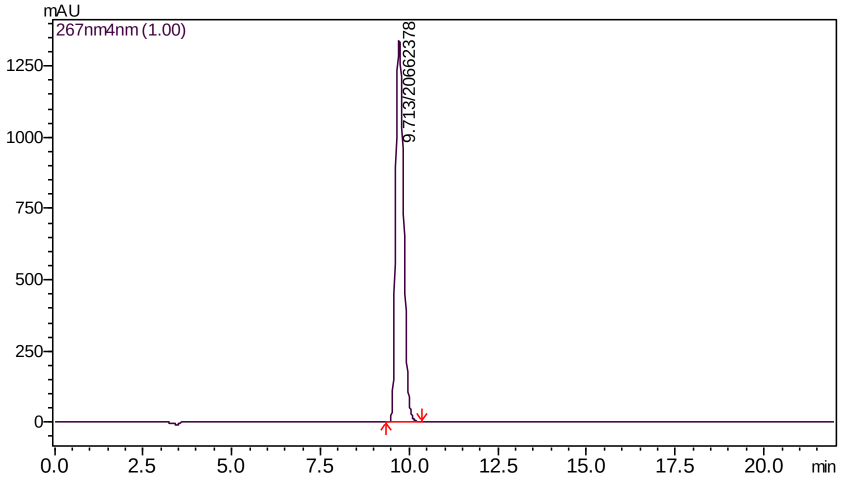

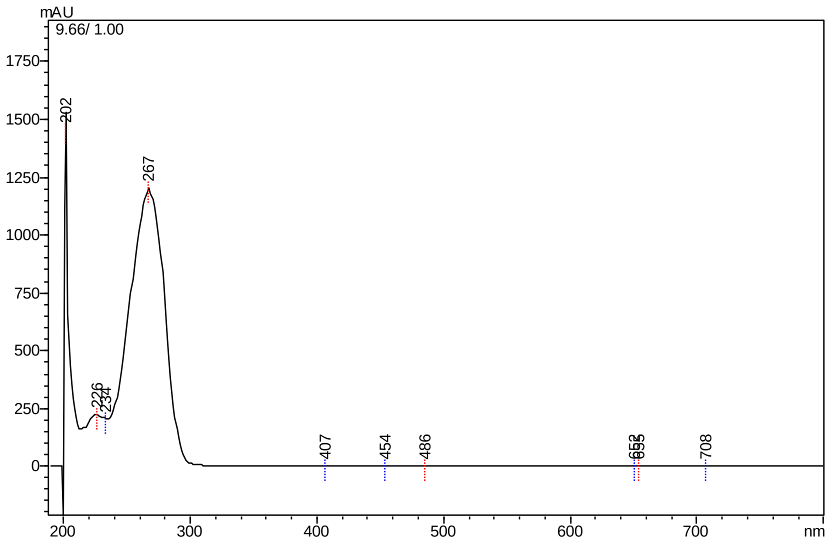

HPLC-DAD analysis of THQ.

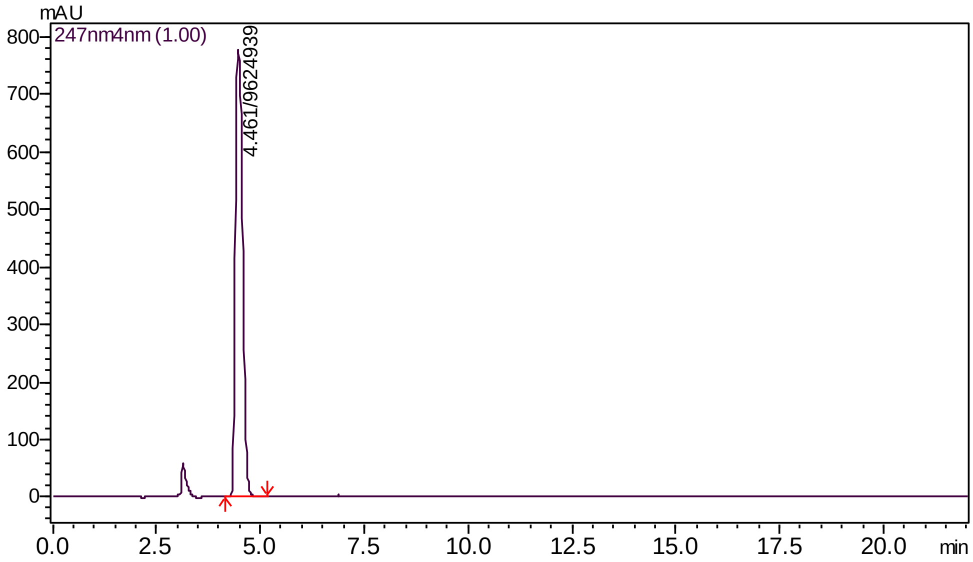

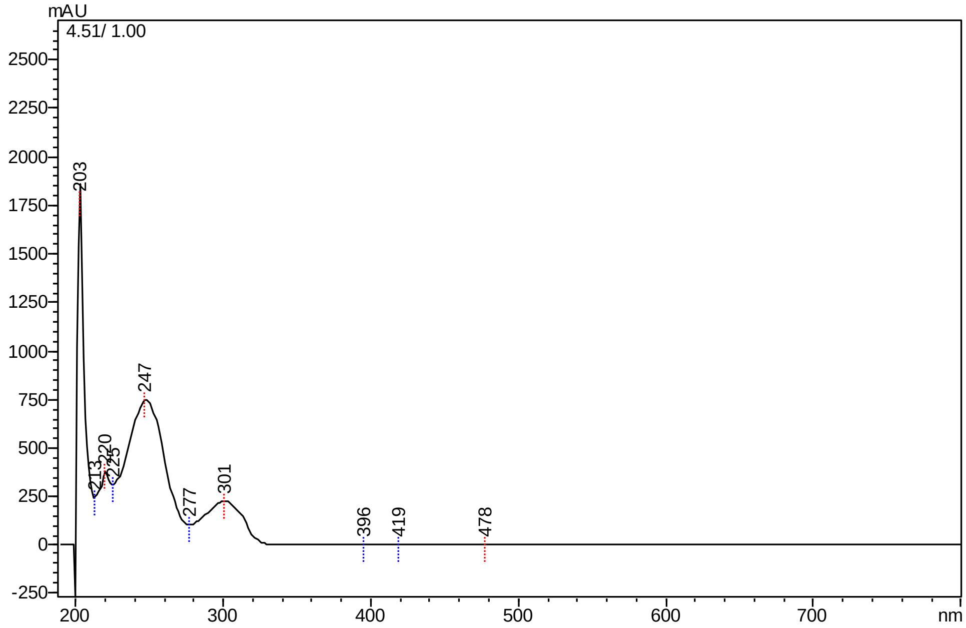

Selected HPLC-DAD analysis of crude mixture from attempts at enantioselective reaction towards DCP 2 (Entry 13, Table S1) Method: IC column, Isocratic eluent Hex:IPA (82:18), Flow 1mL/min:

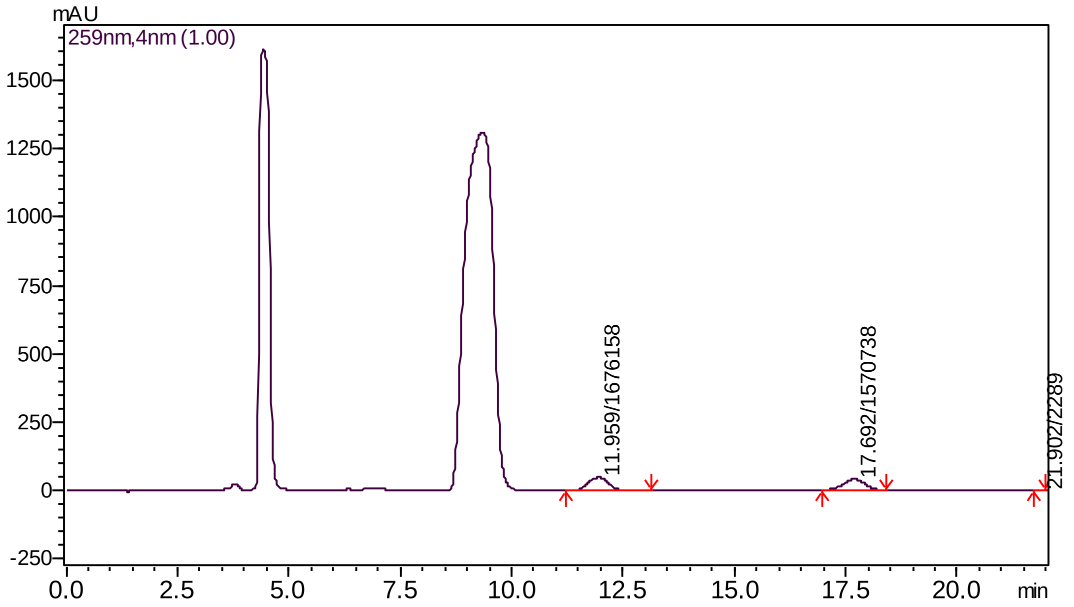

HPLC-DAD analysis of racemic **DCP 3**. Method: Lux-i-amylose 3 column, Isocratic eluent Hex:IPA (92:8), Flow 1mL/min:

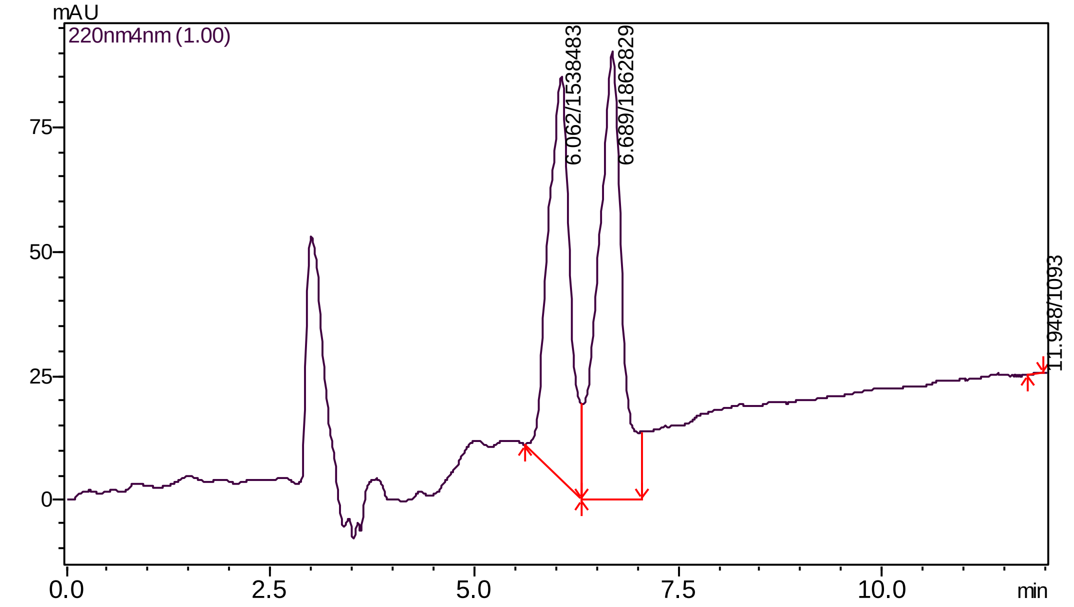

Selected HPLC-DAD analysis of crude mixture from attempts at enantioselective reaction towards DCP 2 (Entry 16, Table S1) Method: IC column, Isocratic eluent Hex:IPA (92: 8), Flow 1mL/min:

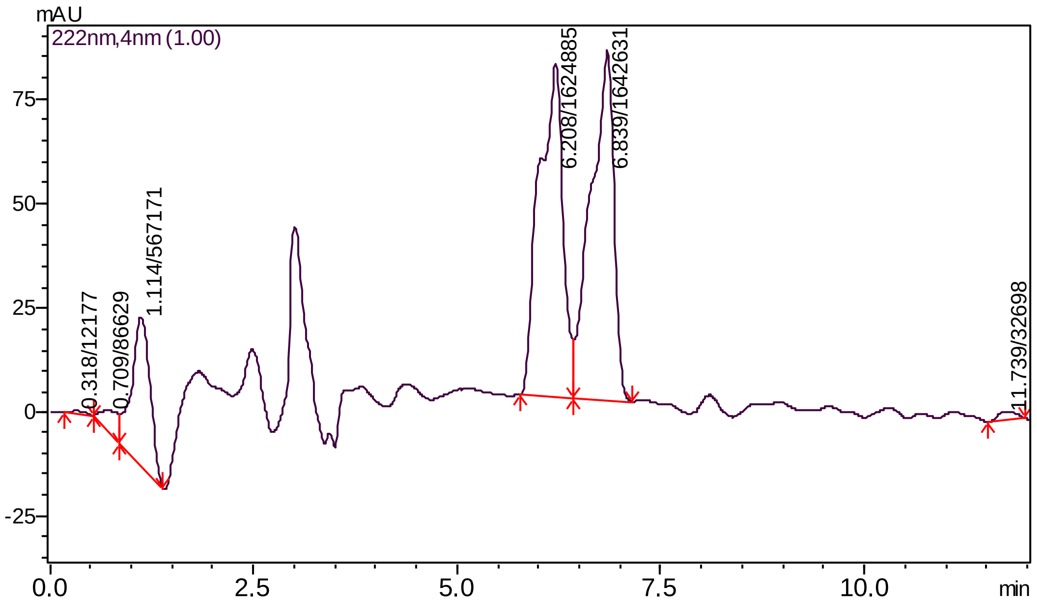

NMR Characterization:

^1H NMR (300 MHz, $CDCl_3$) and ^{13}C NMR (75 MHz, $CDCl_3$) of compound **1a**

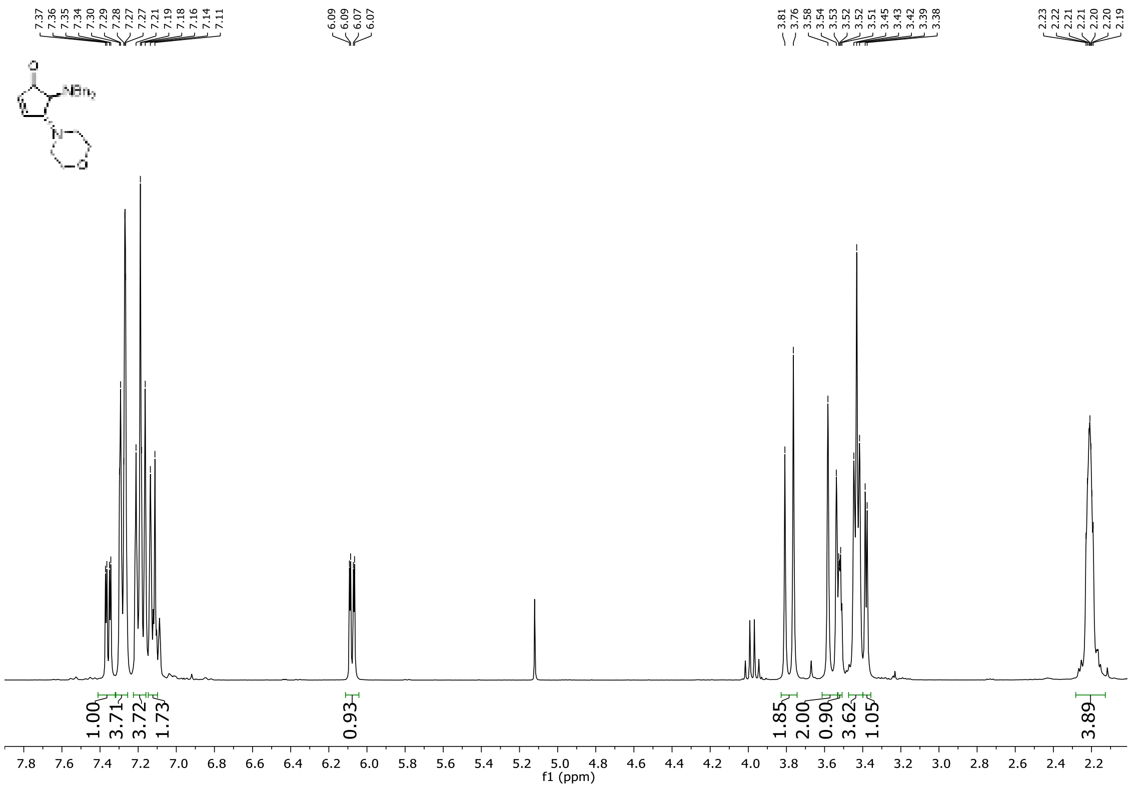

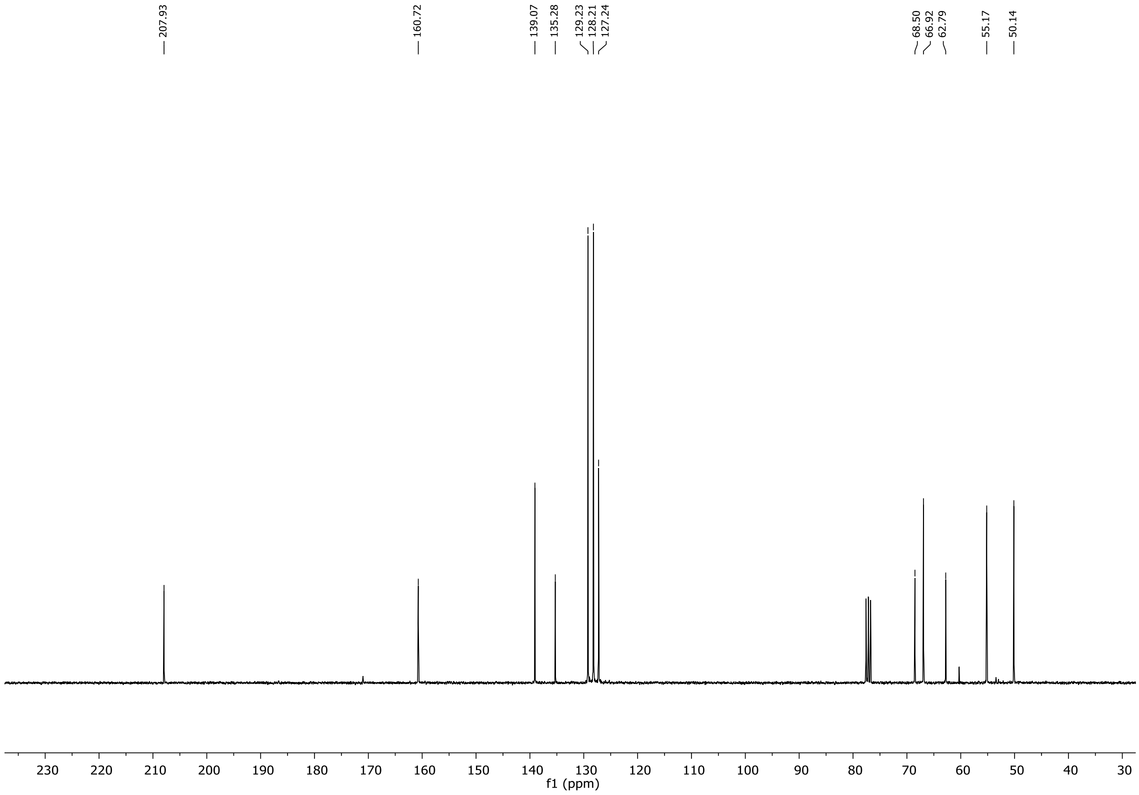

COSY and NOESY of compound **1a**

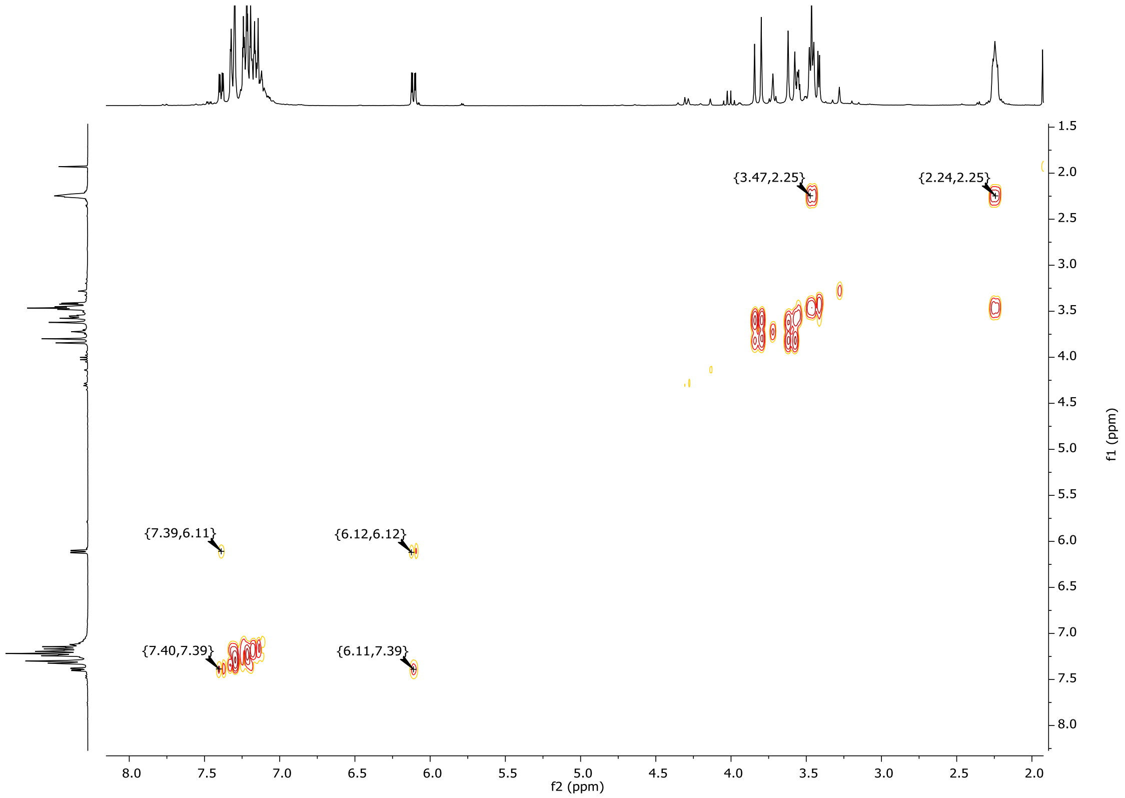

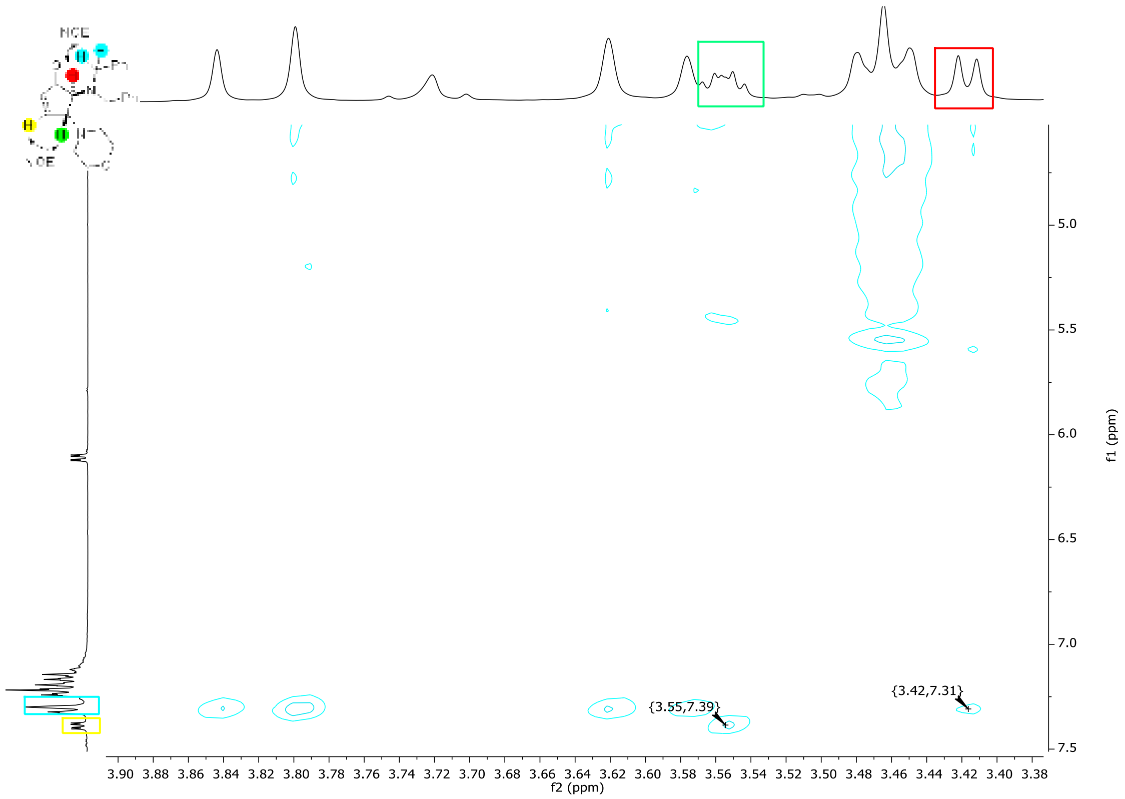

HSQC and HMBC of compound **1a**

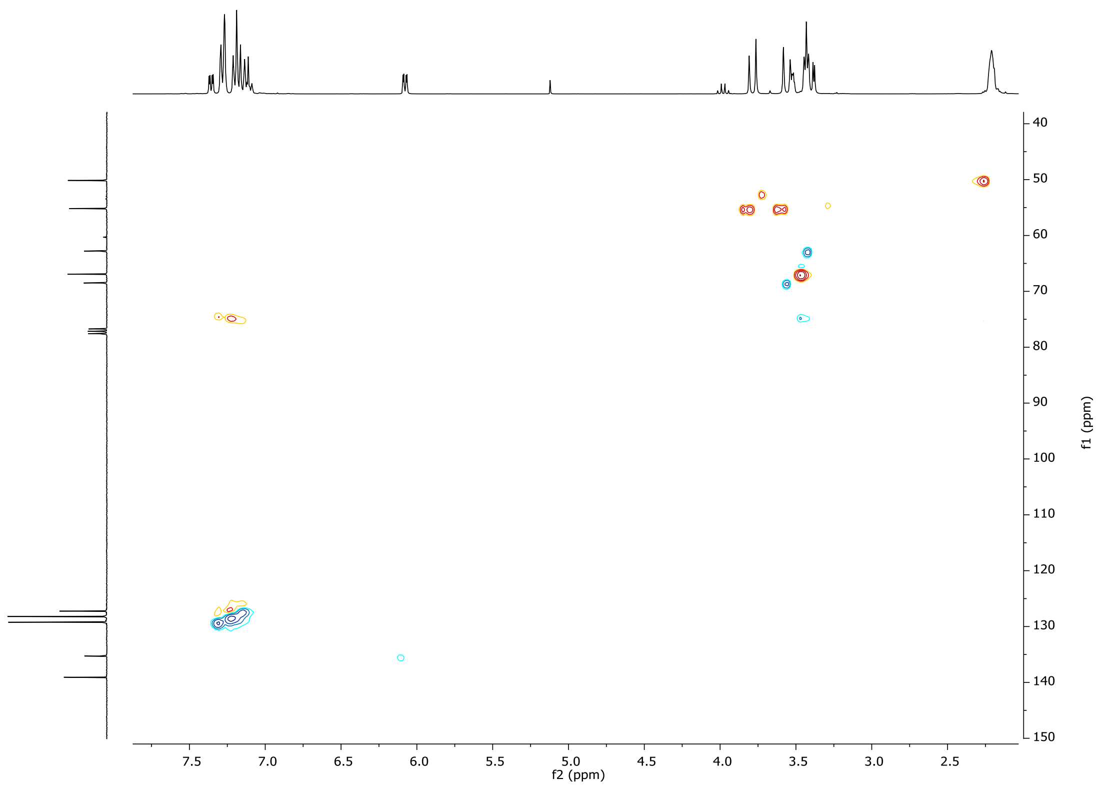

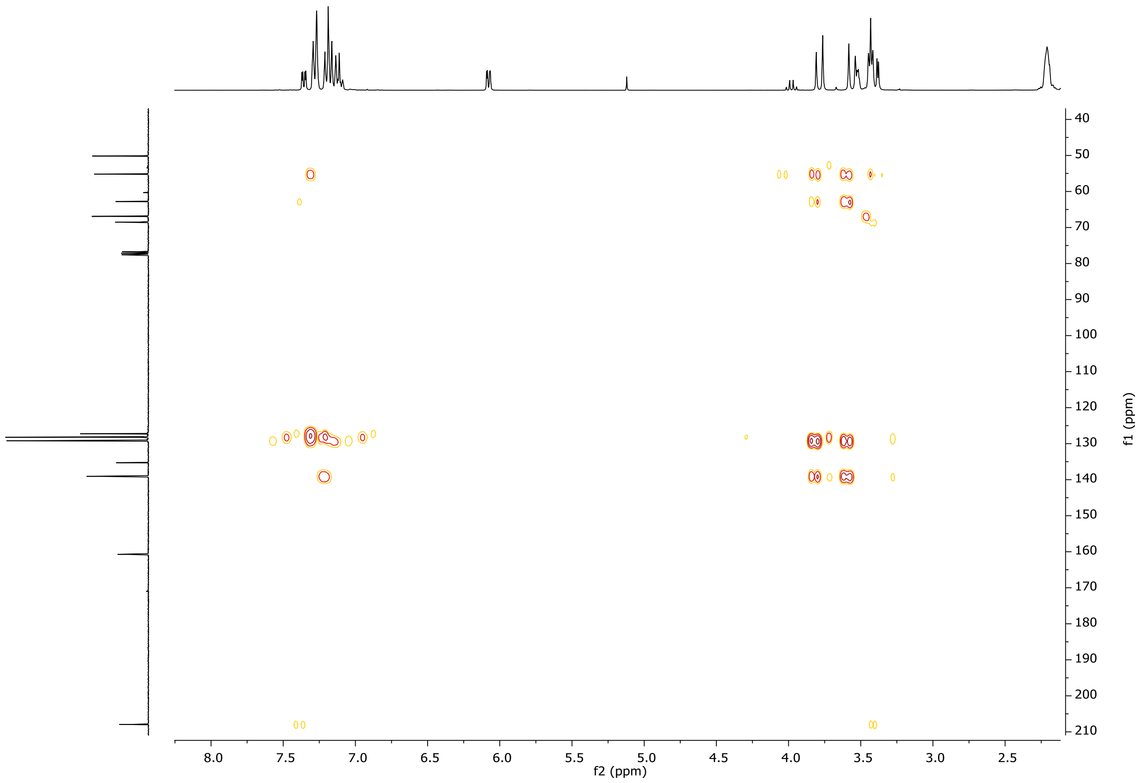

^{1}H NMR (300 MHz, $CDCl_3$) and ^{13}C NMR (75 MHz, $CDCl_3$) of compound **1b**

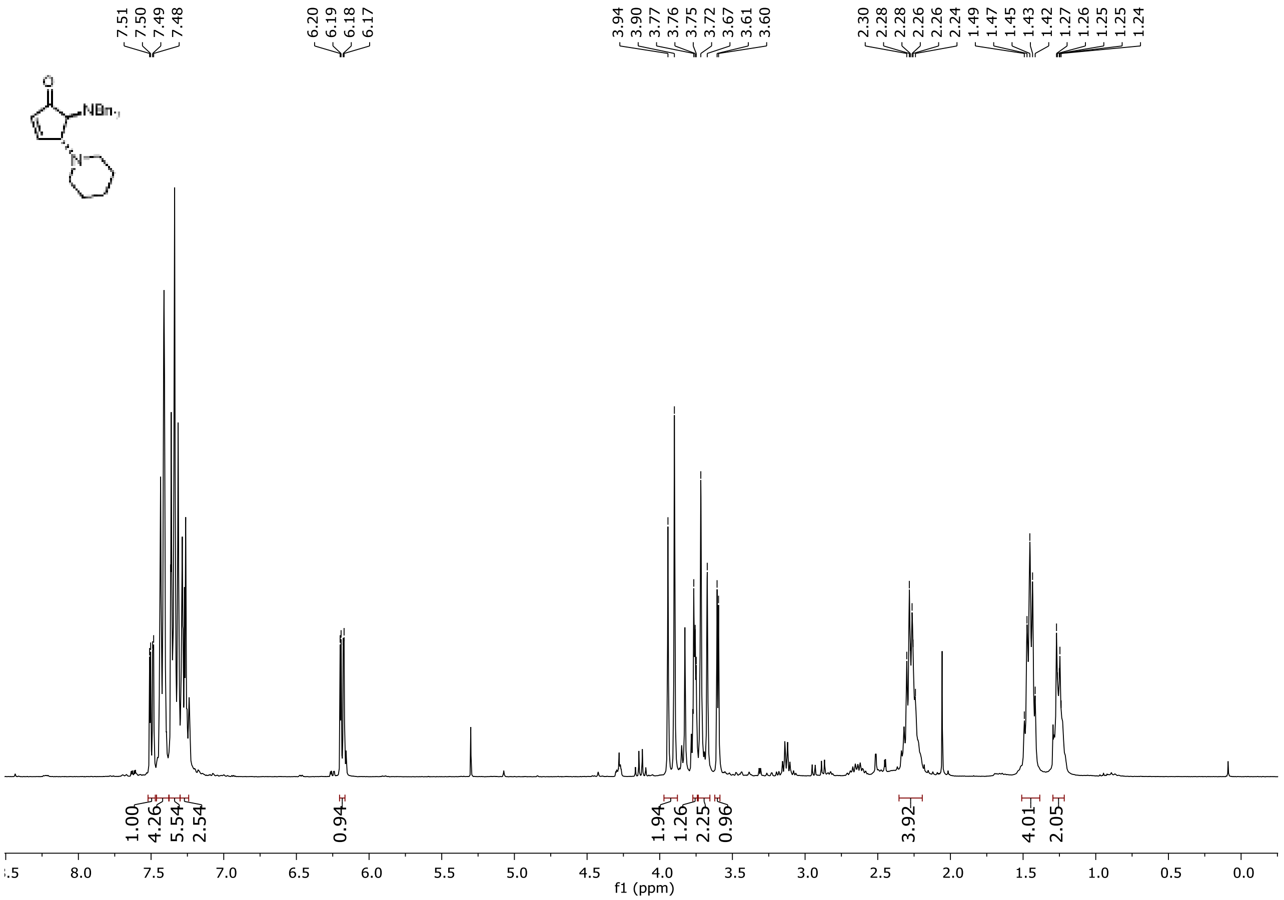

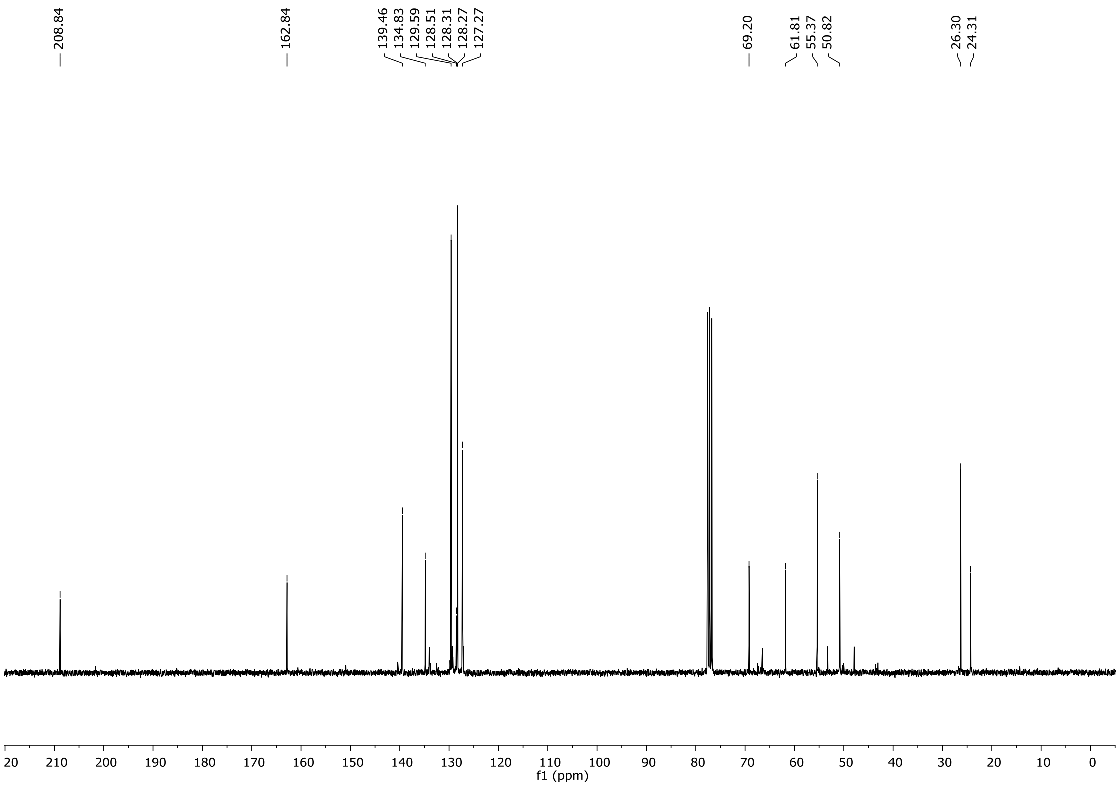

COSY and NOESY of compound **1b**

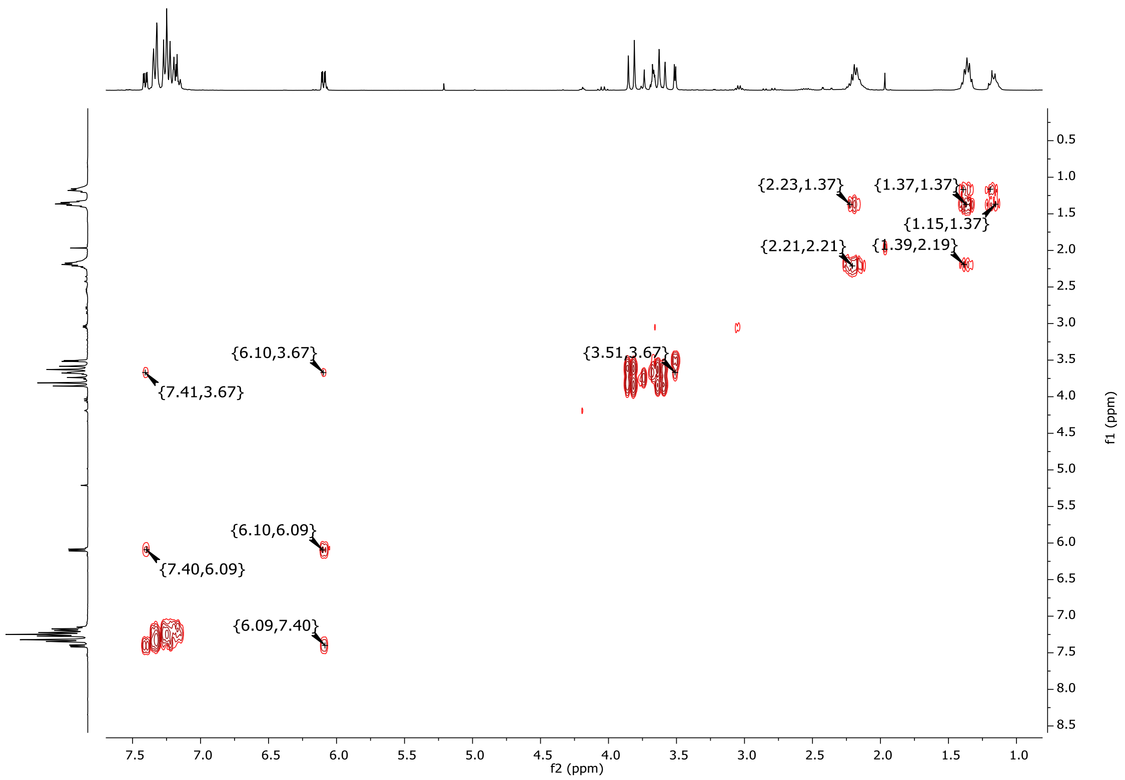

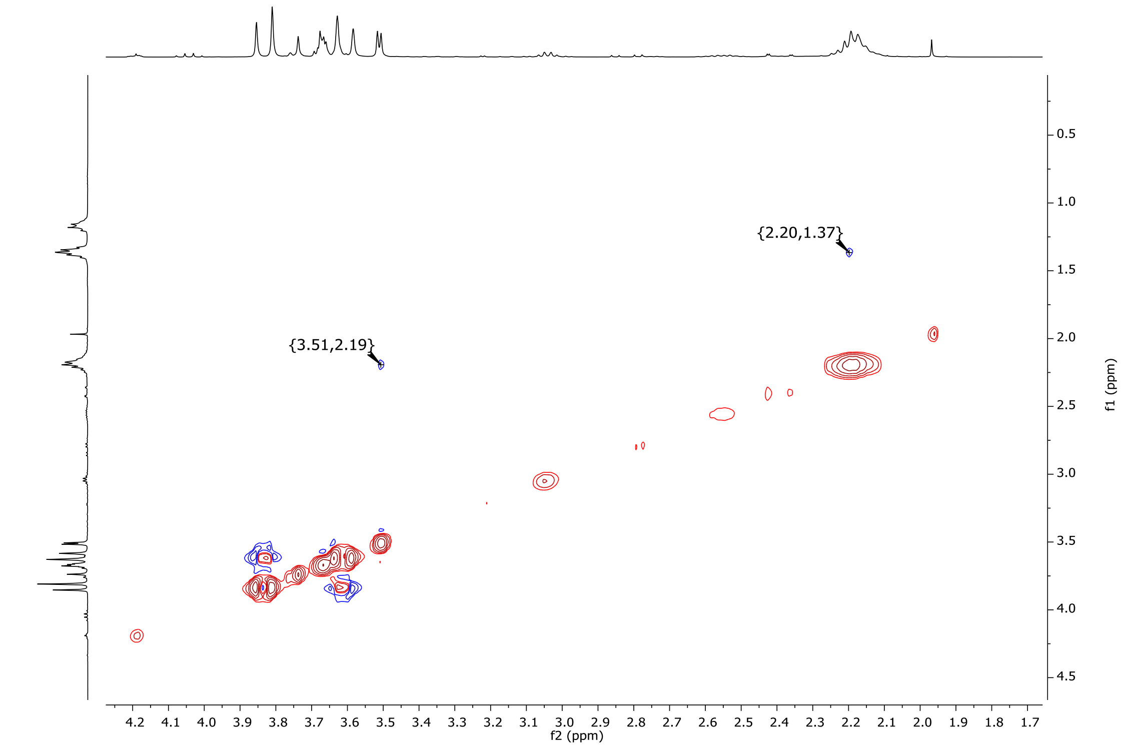

HSQC and HMBC of compound **1b**

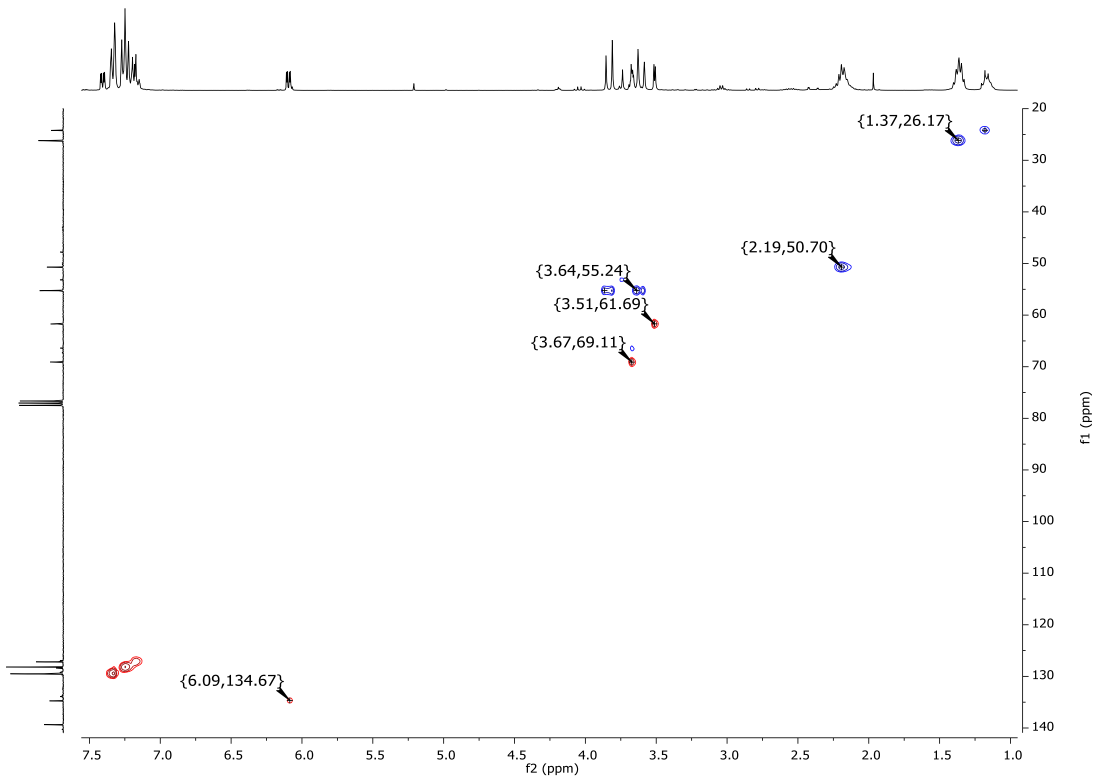

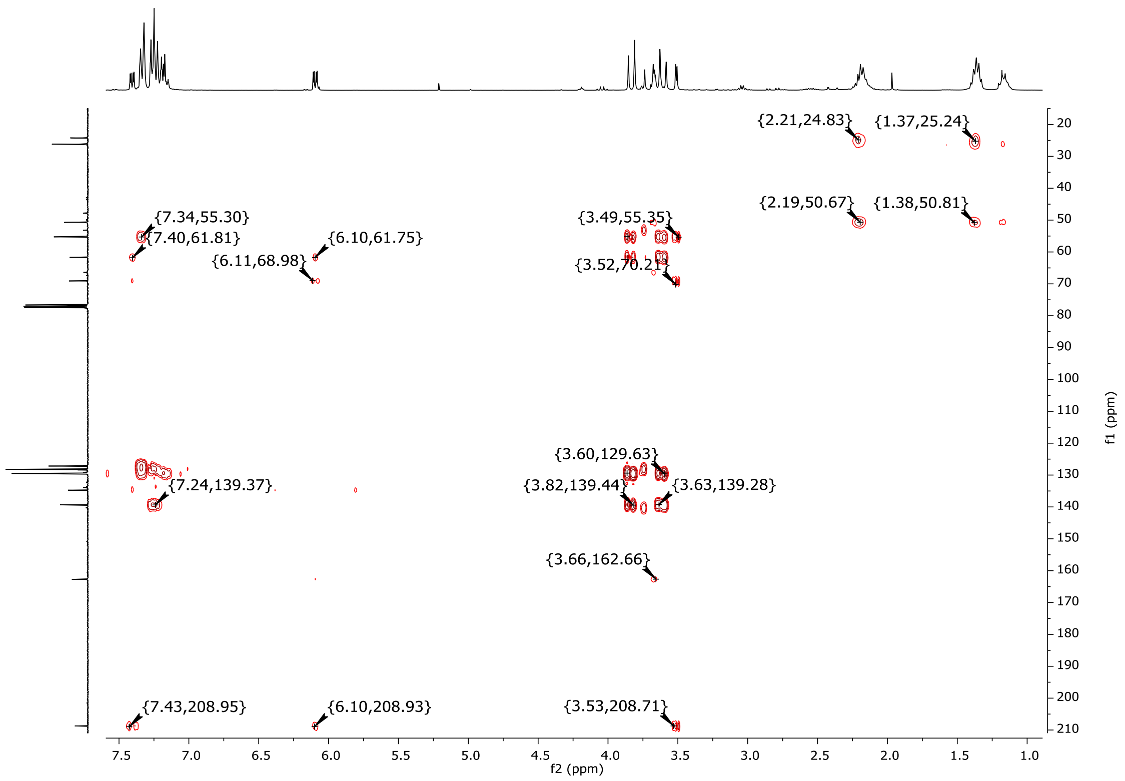

^{1}H NMR (300 MHz, $CDCl_3$) and ^{13}C NMR (75 MHz, $CDCl_3$) of compound **1c**

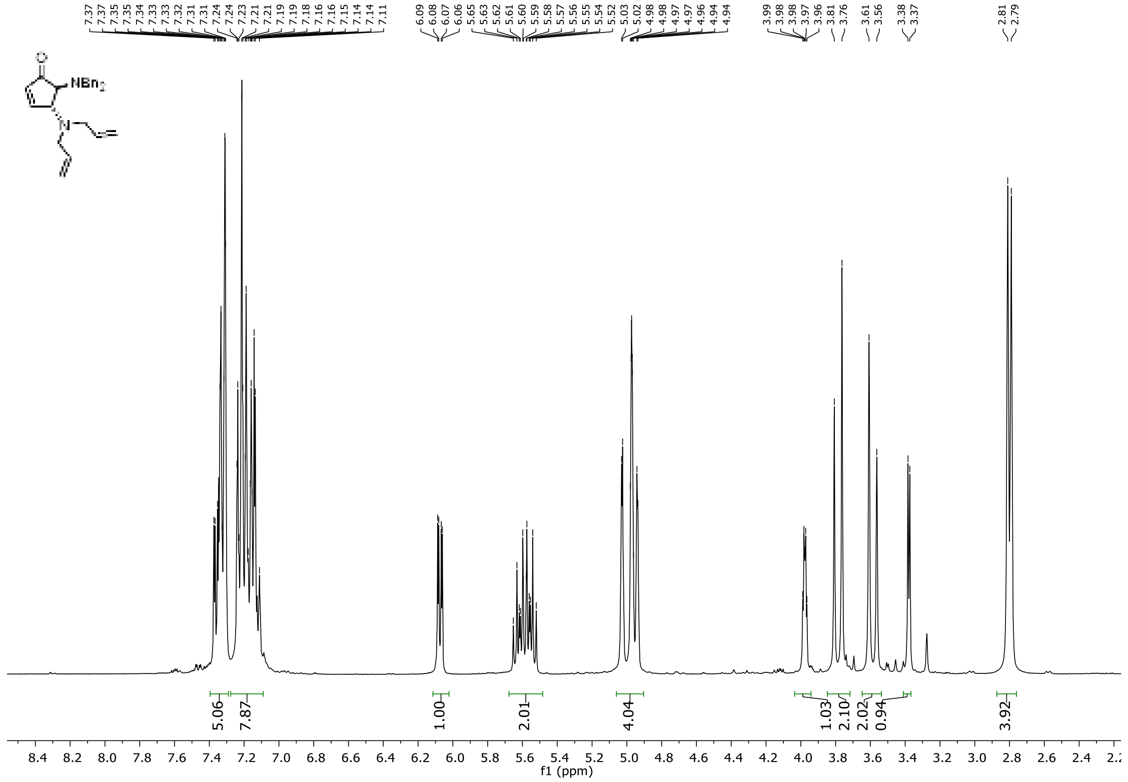

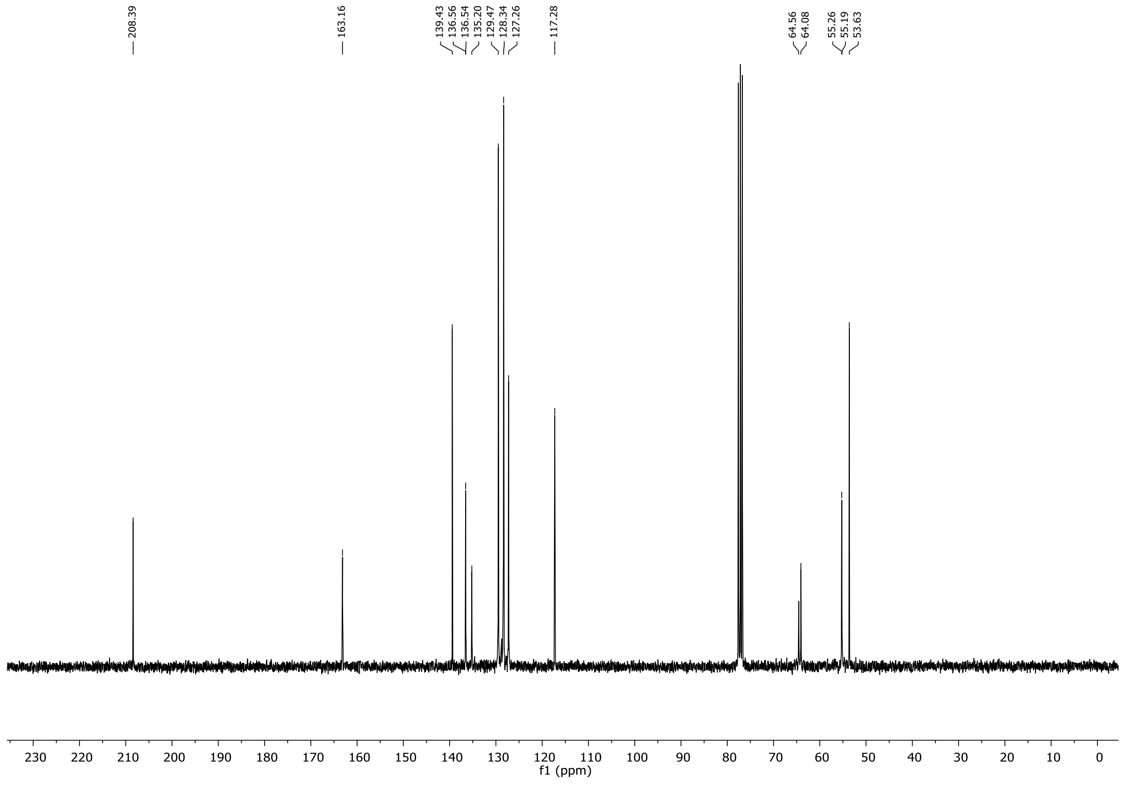

COSY and NOESY of compound **1c**

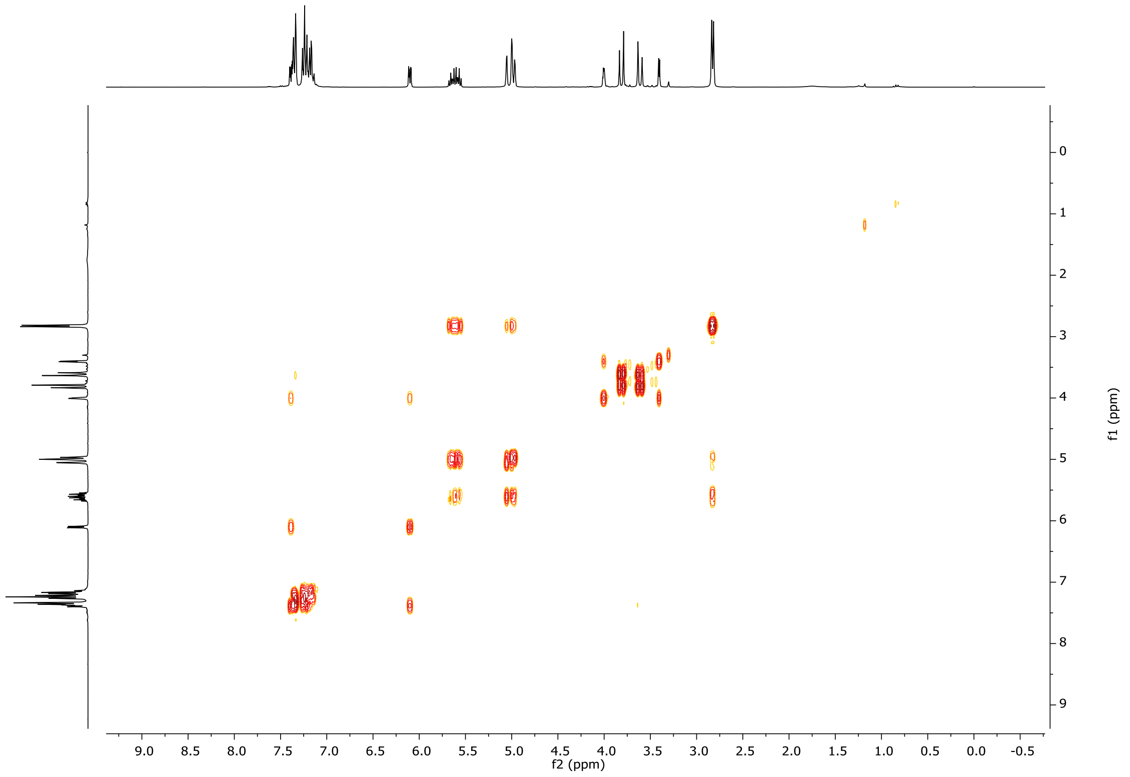

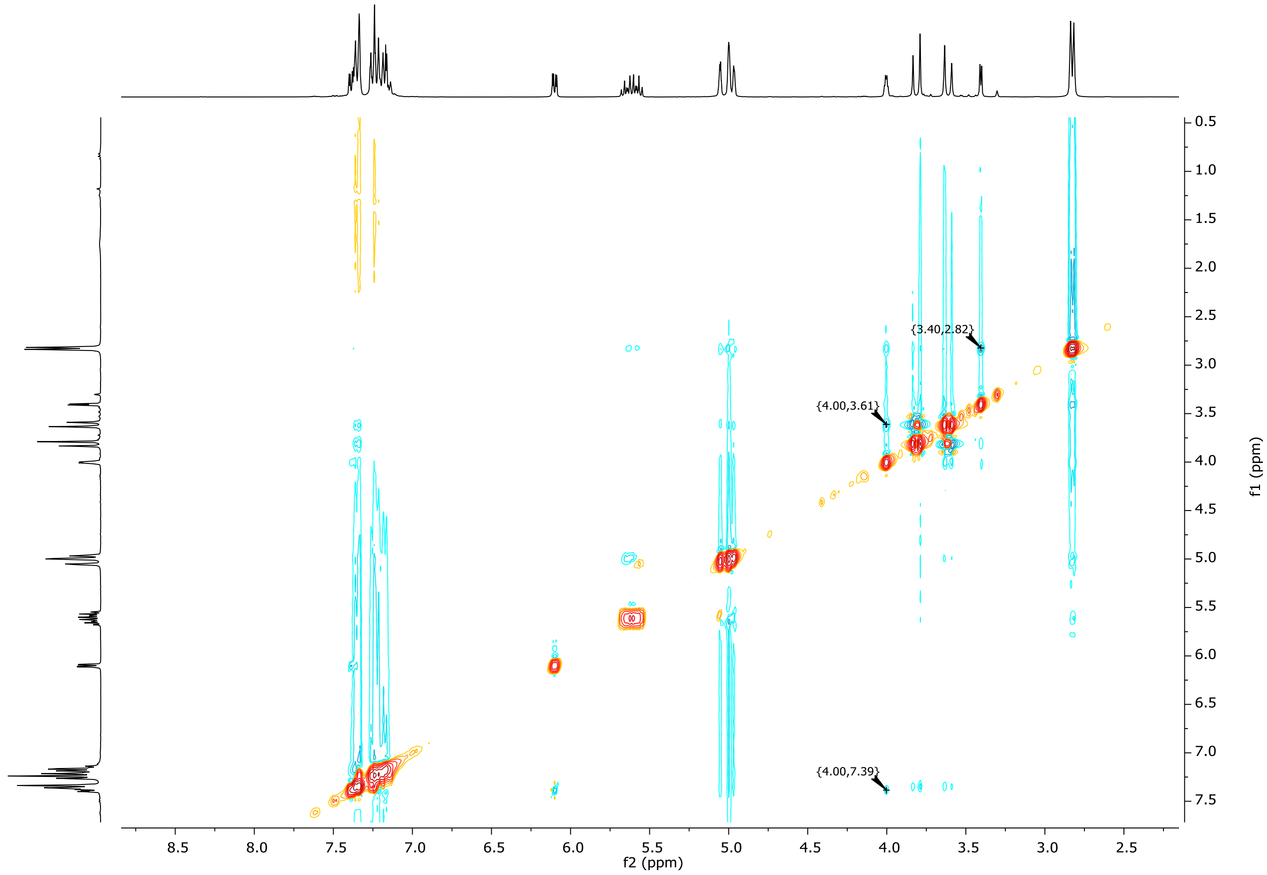

HSQC and HMBC of compound **1c**

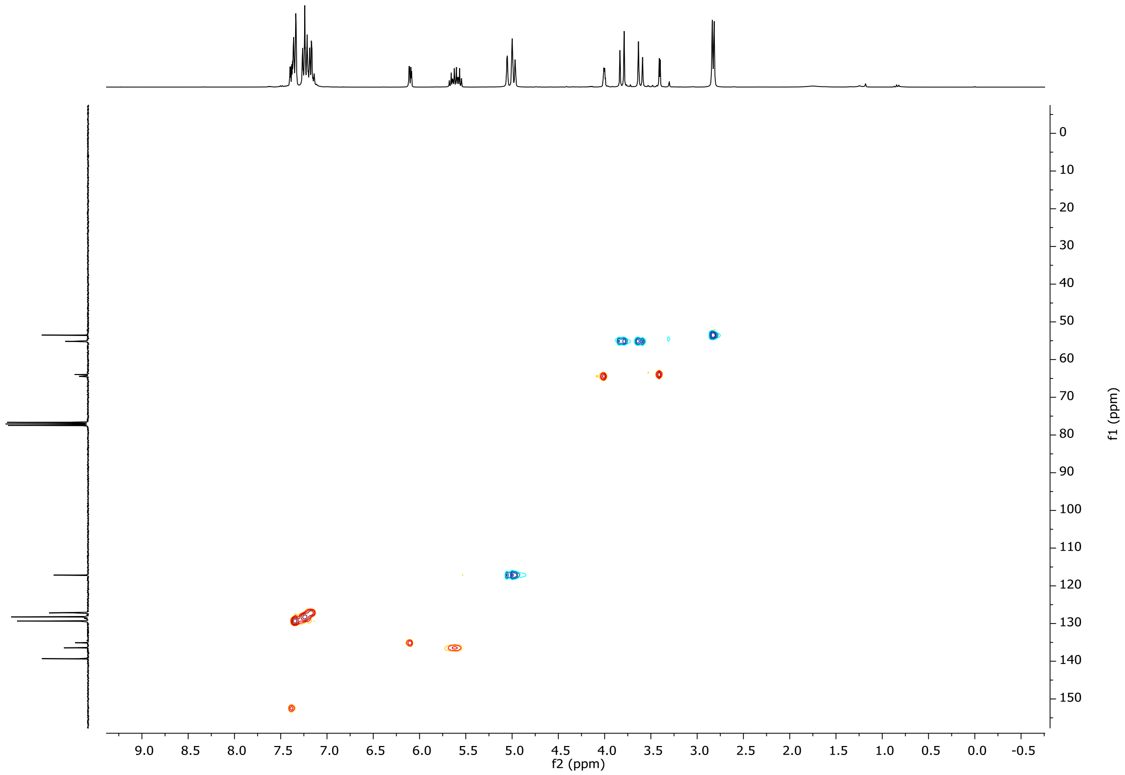

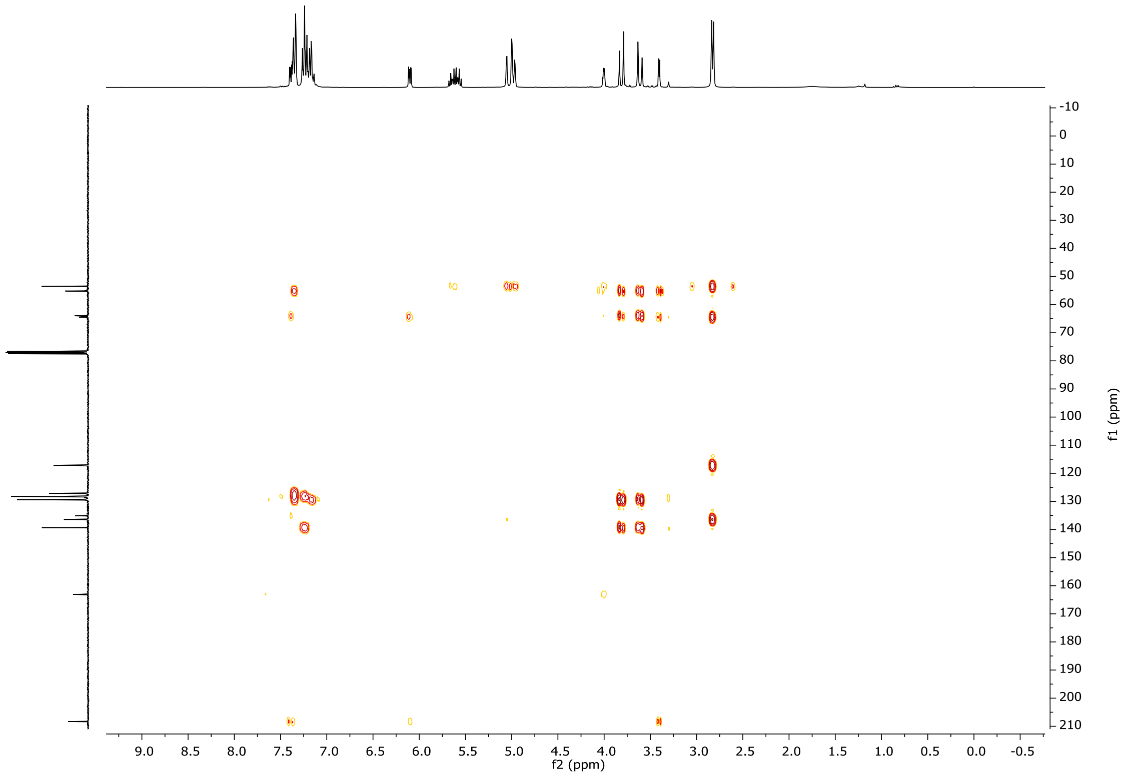

HSQC and HMBC of compound **1c**

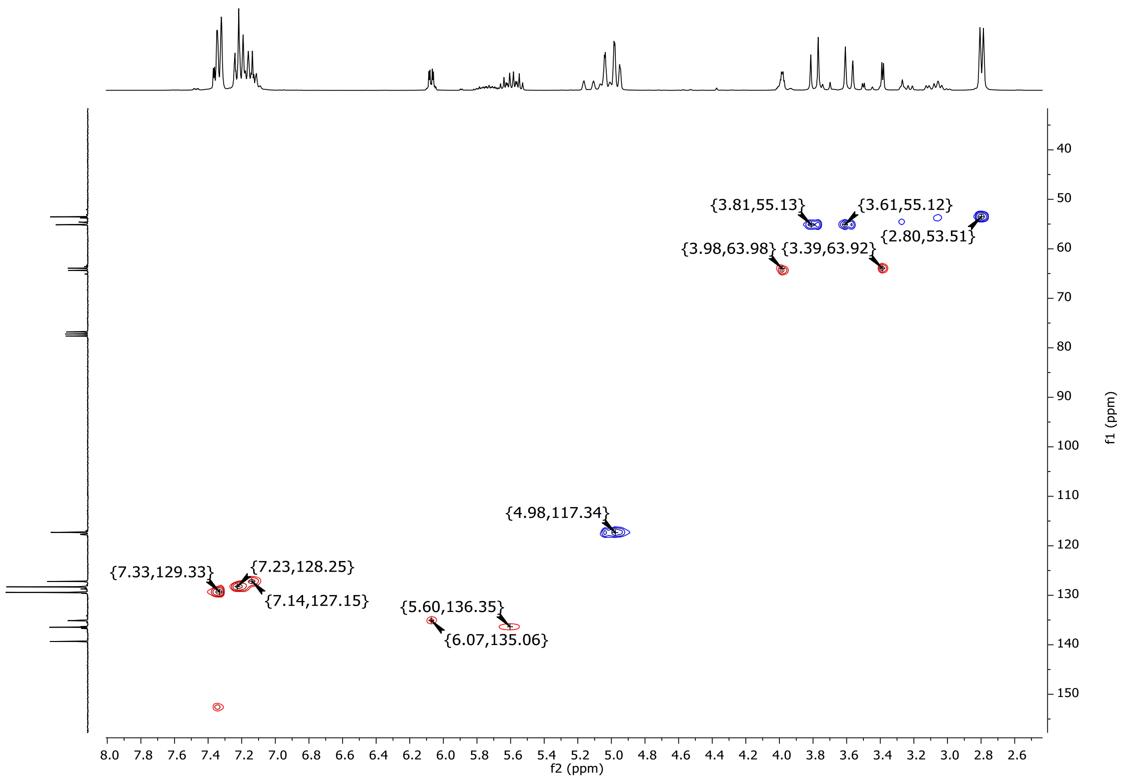

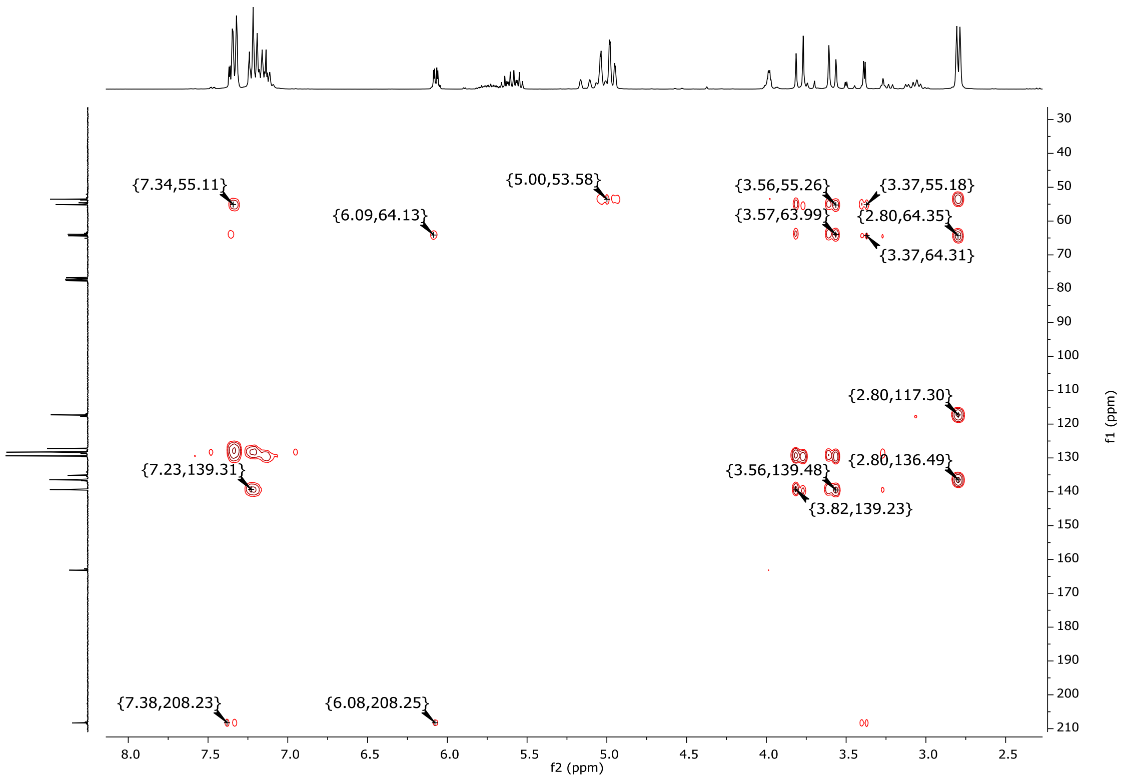

^{1}H NMR (300 MHz, $CDCl_3$) and ^{13}C NMR (75 MHz, $CDCl_3$) of compound **1d**

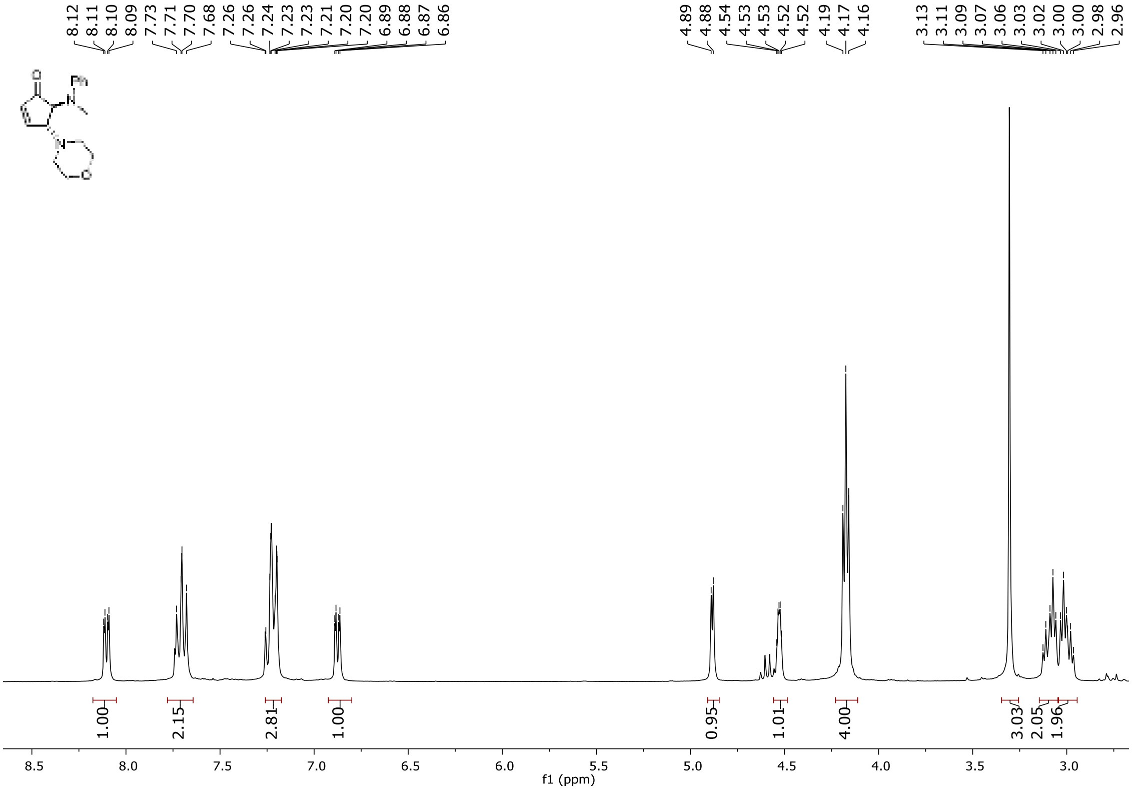

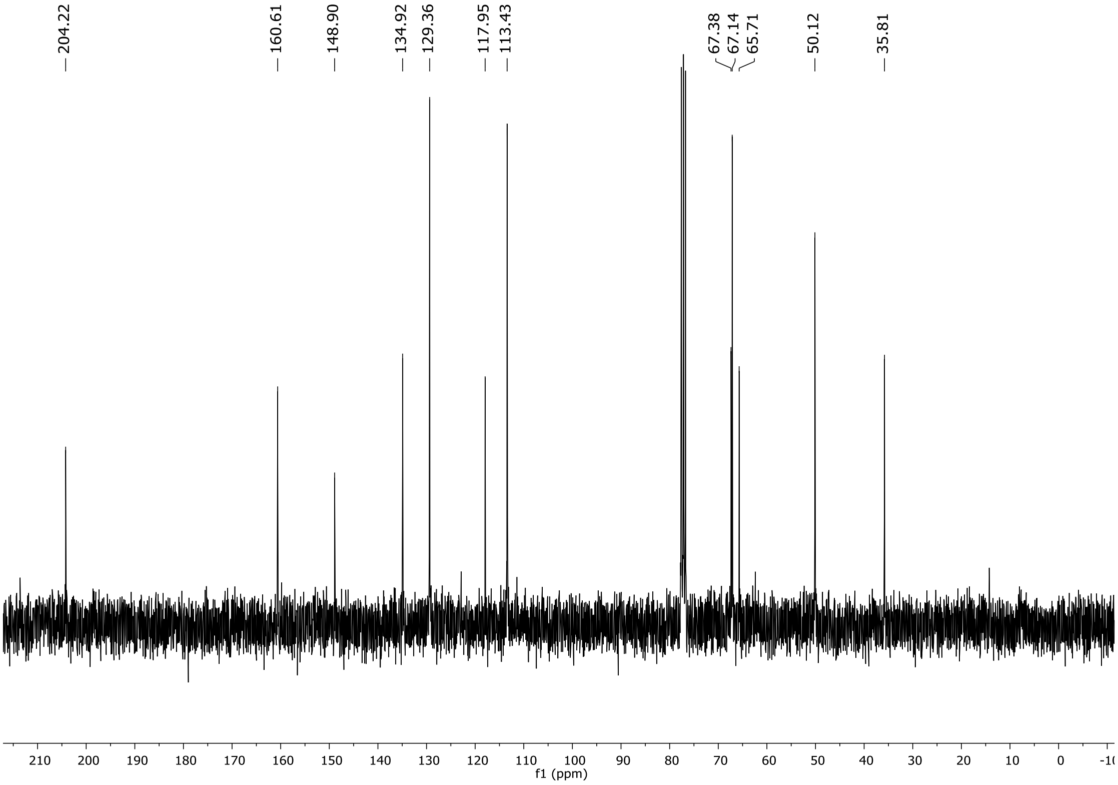

COSY and NOESY of compound **1d**

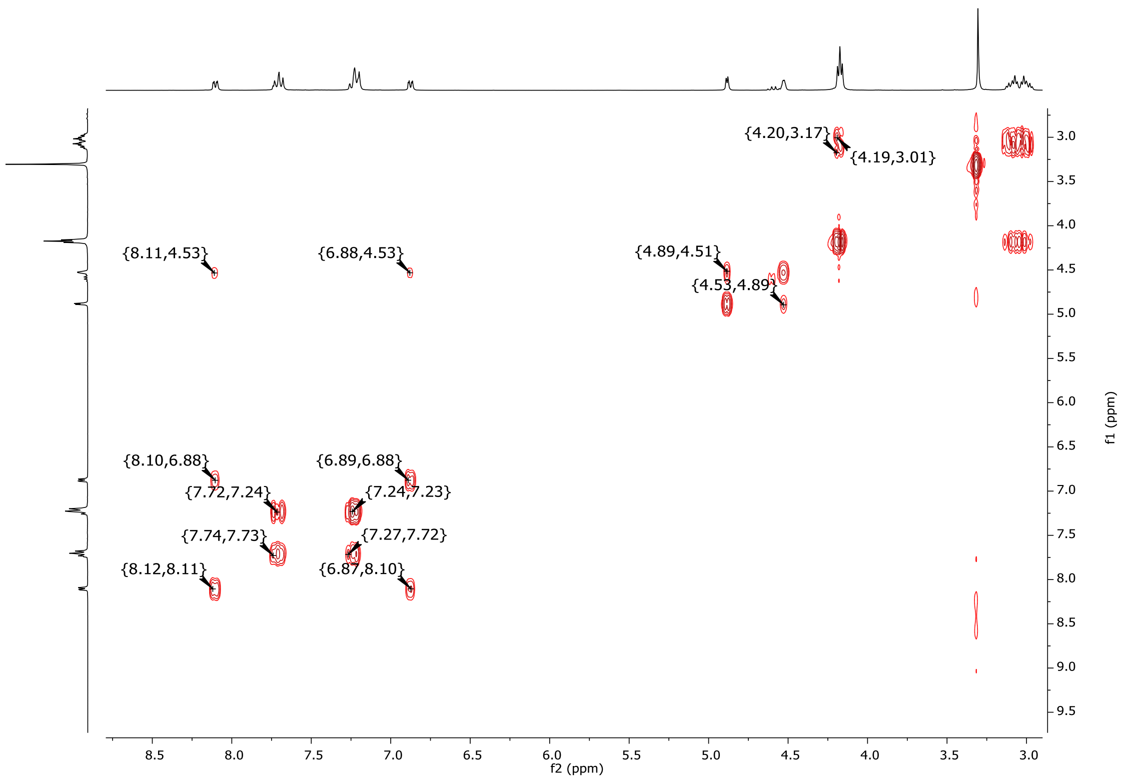

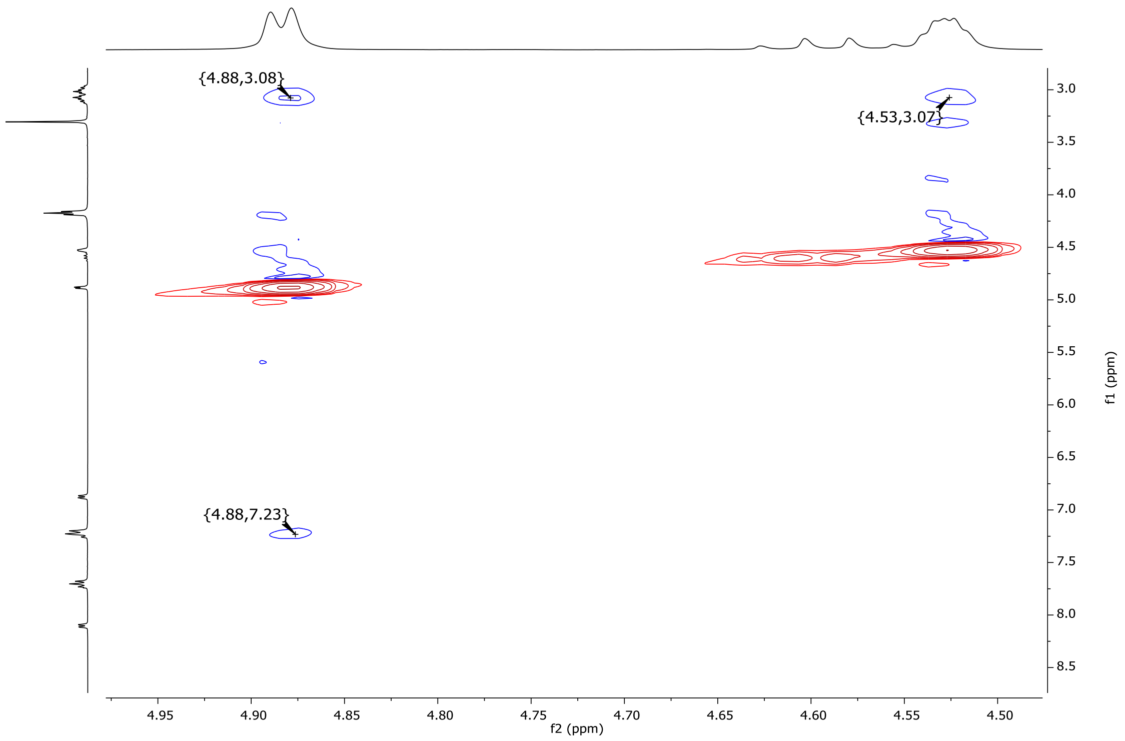

HSQC and HMBC of compound **1d**

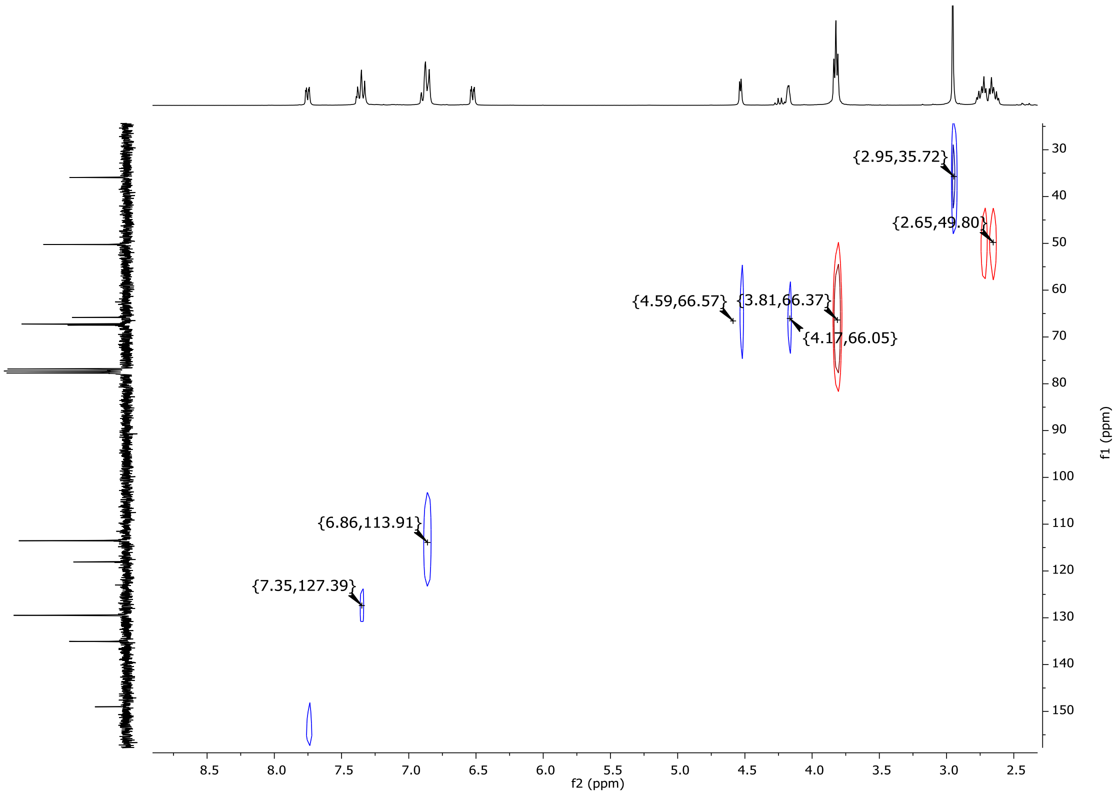

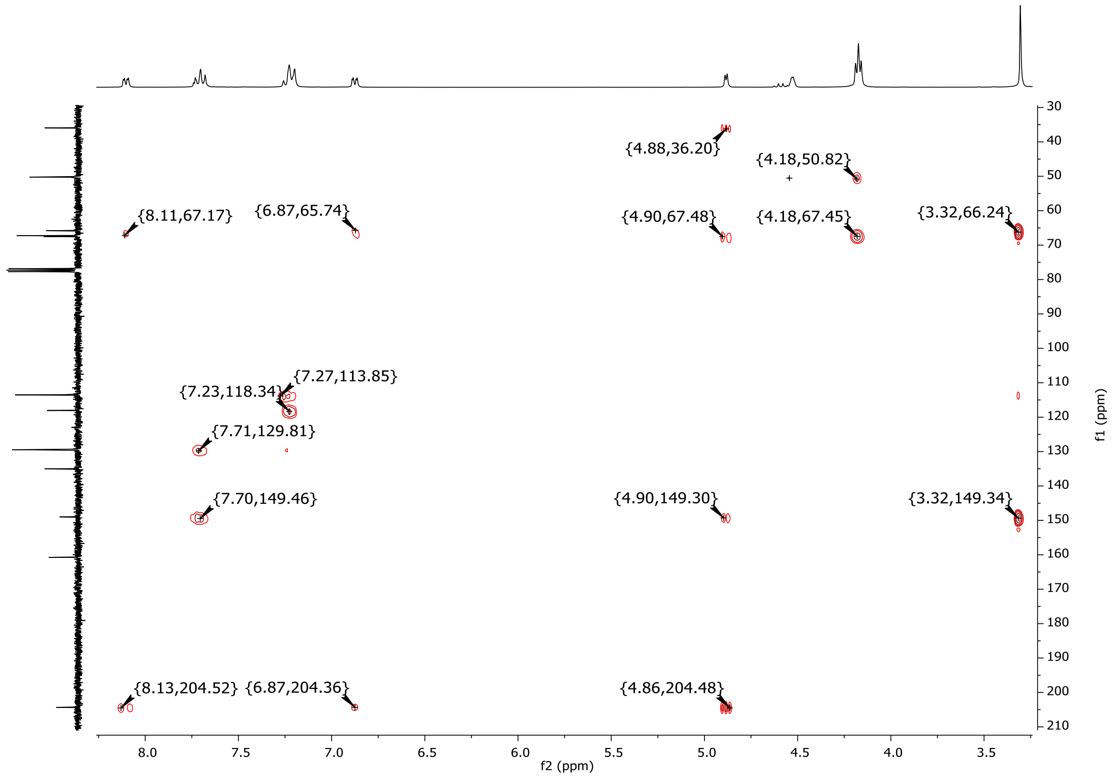

^{1}H NMR (300 MHz, $CDCl_3$) and ^{13}C NMR (75 MHz, $CDCl_3$) of compound **1e**

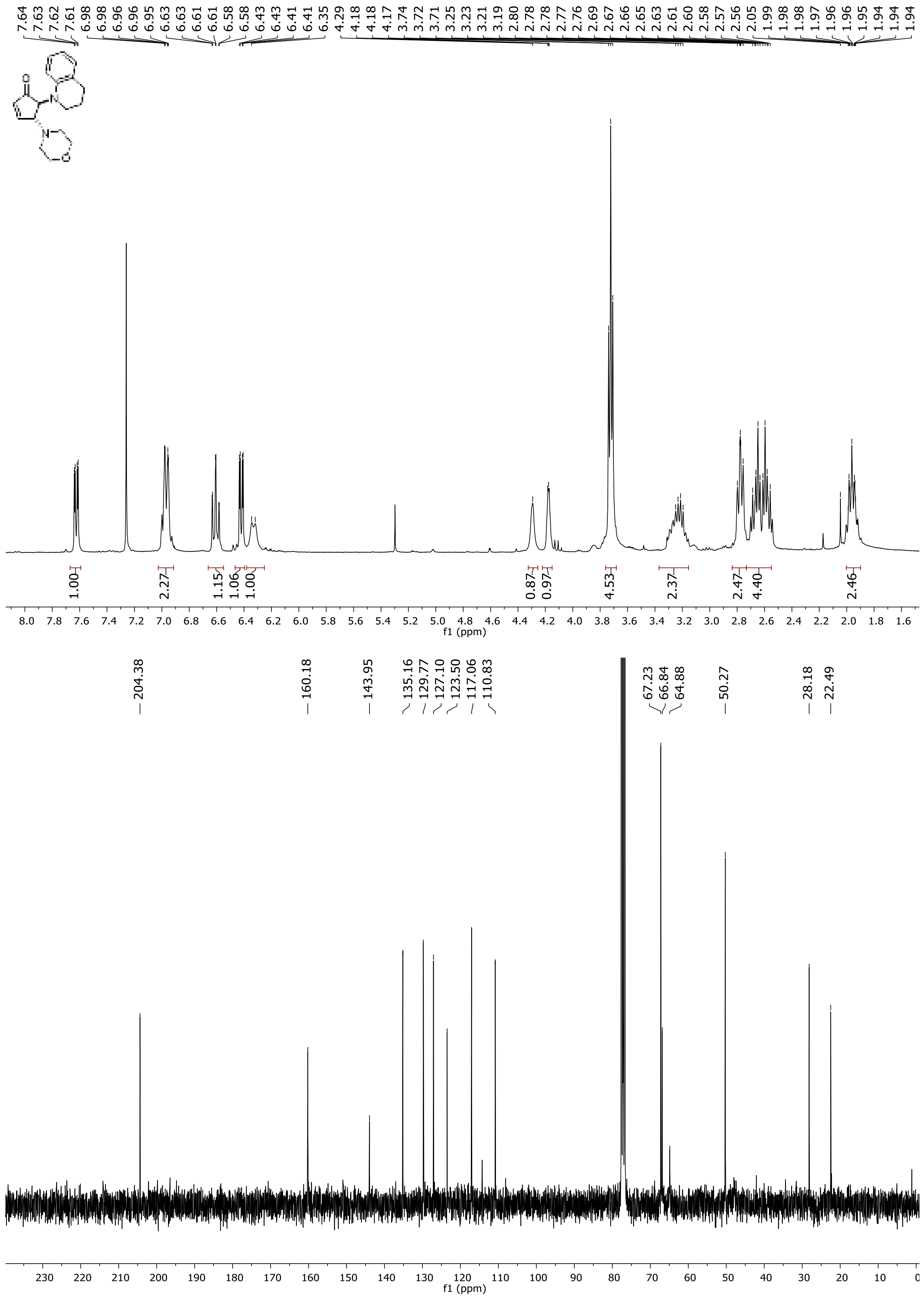

COSY and NOESY of compound **1e**

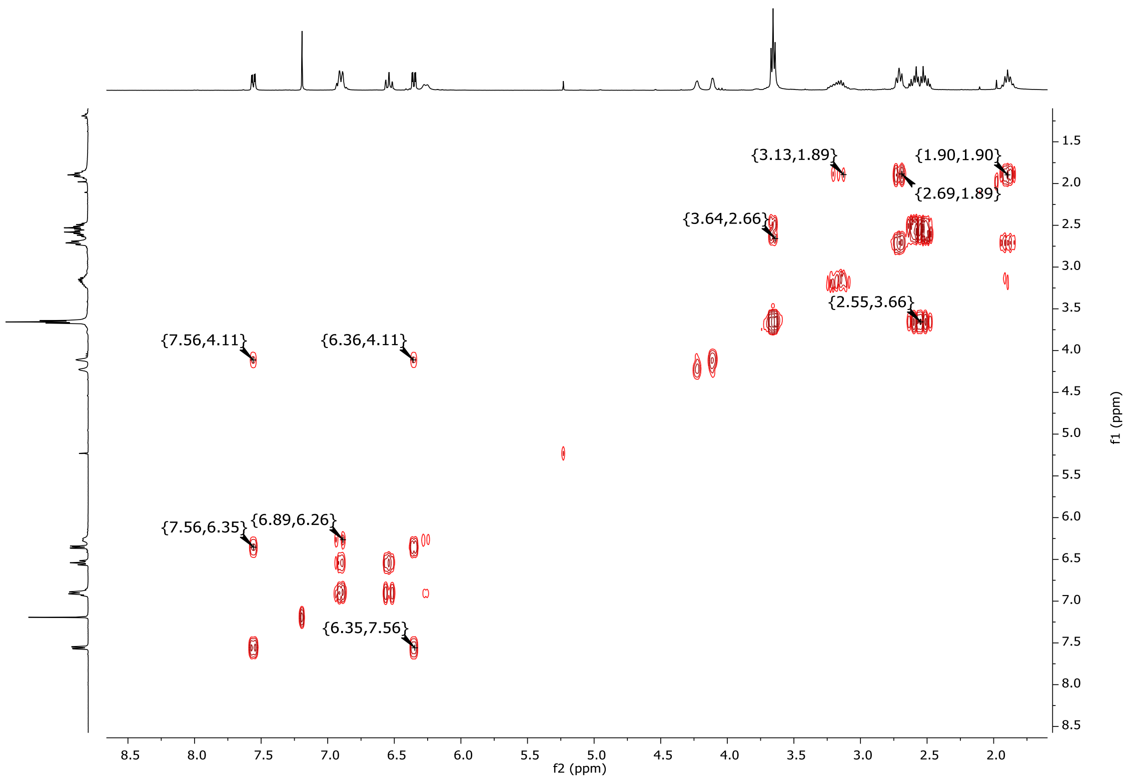

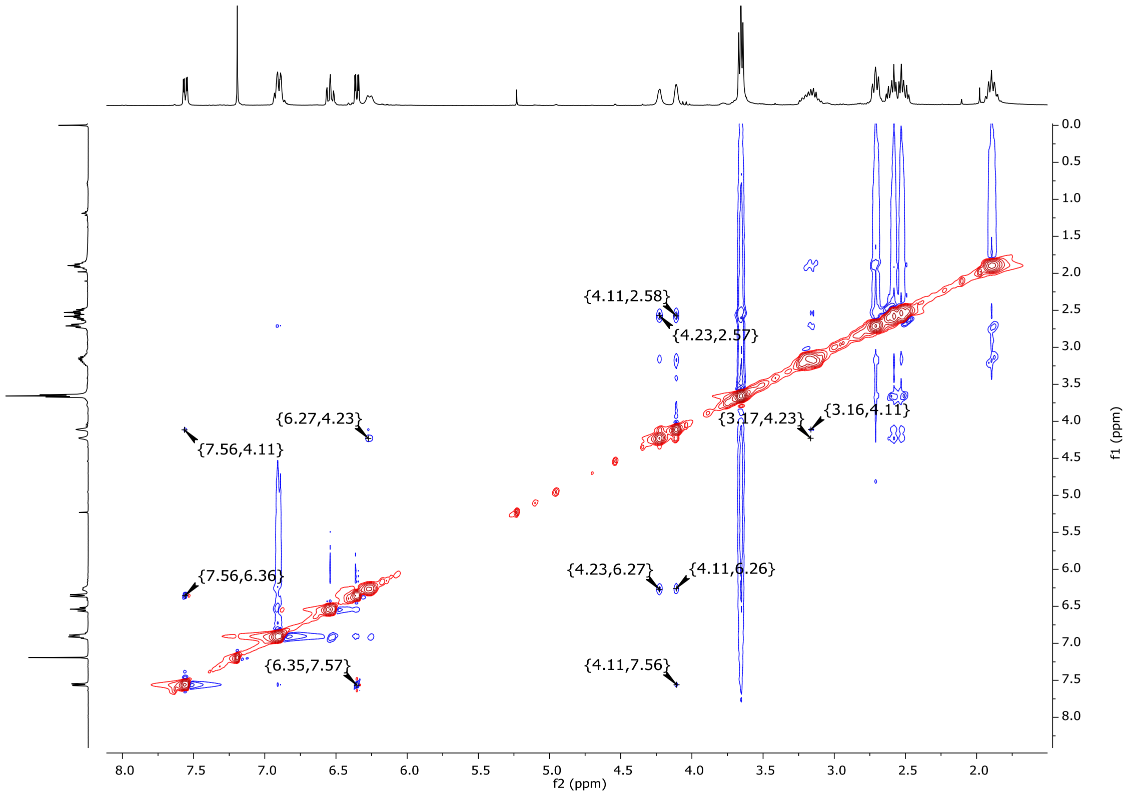

HSQC and HMBC of compound **1e**

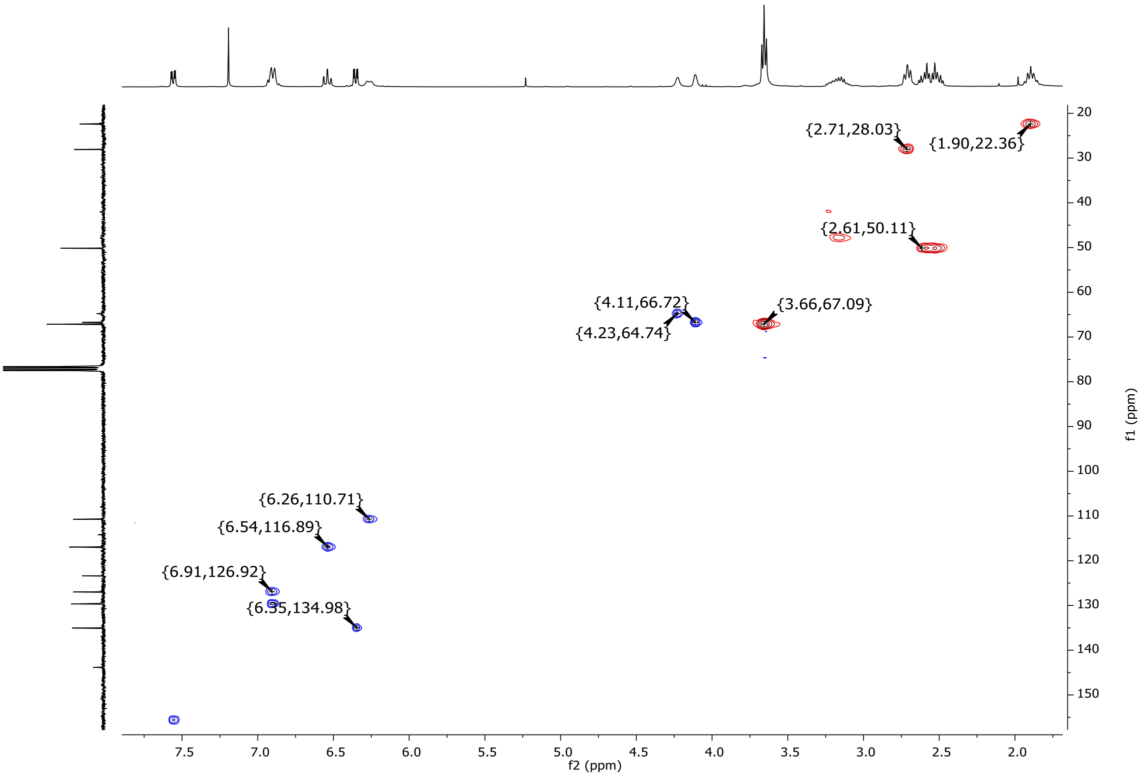

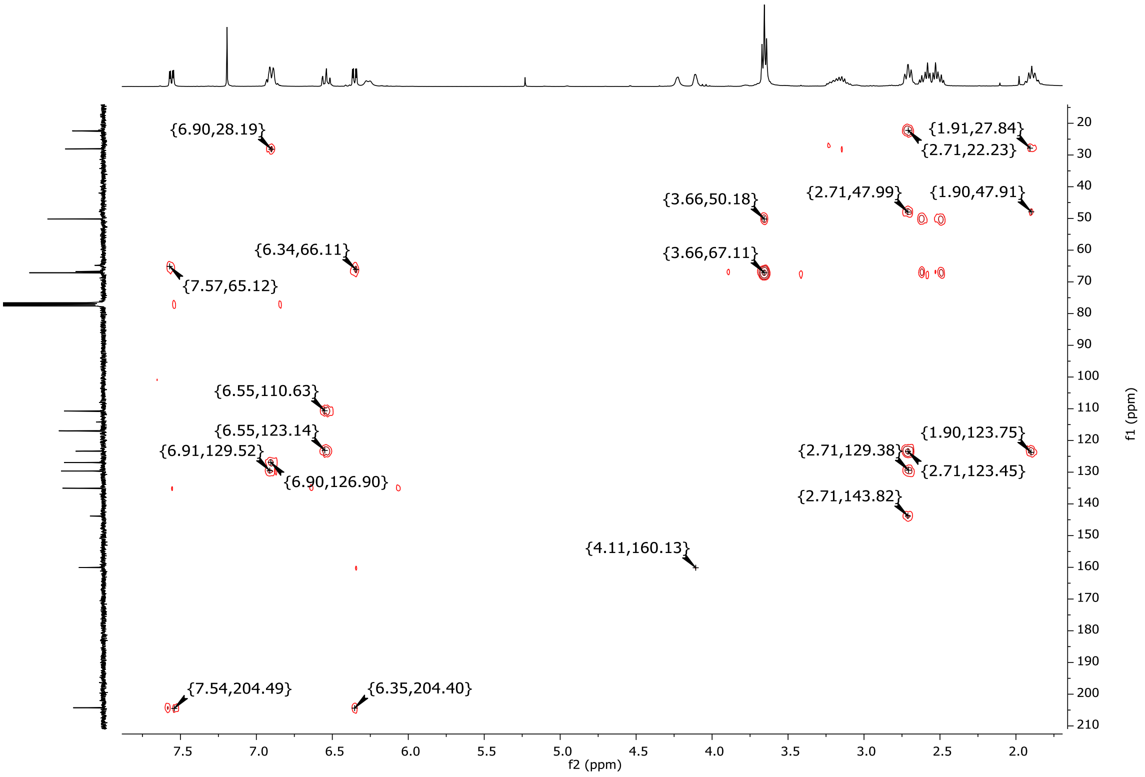

^{1}H NMR (300 MHz, $CDCl_3$) and ^{13}C NMR (75 MHz, $CDCl_3$) of compound **1f**

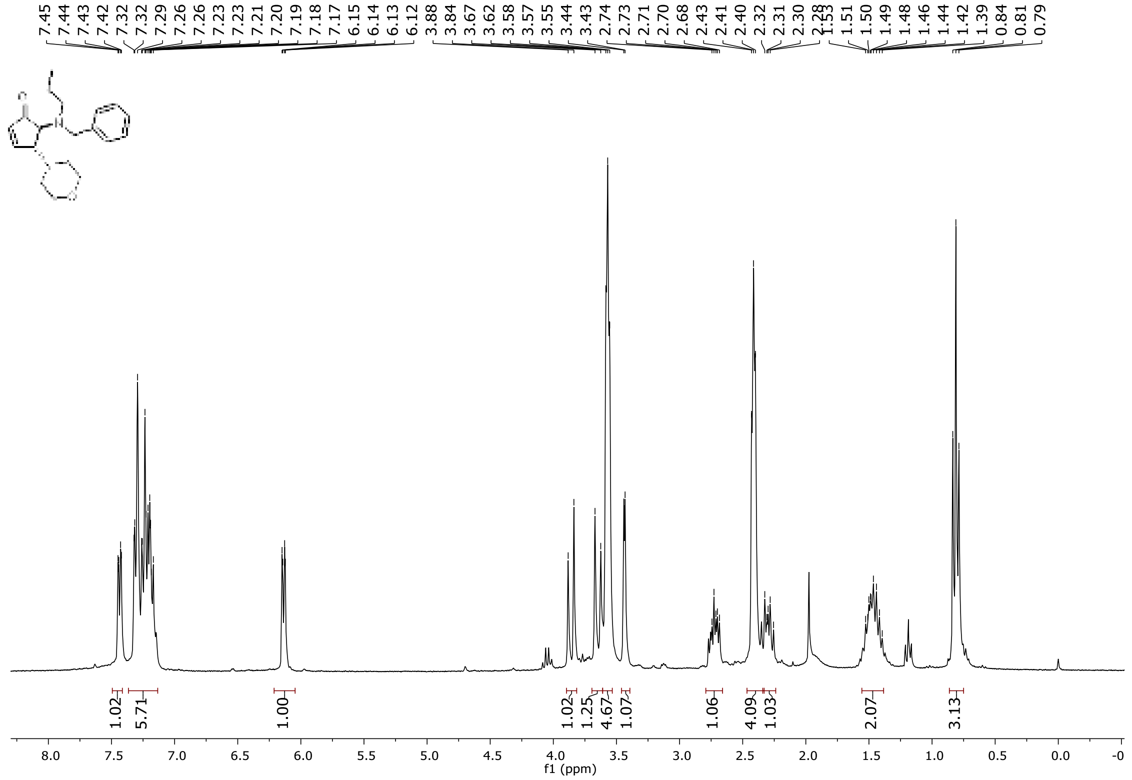

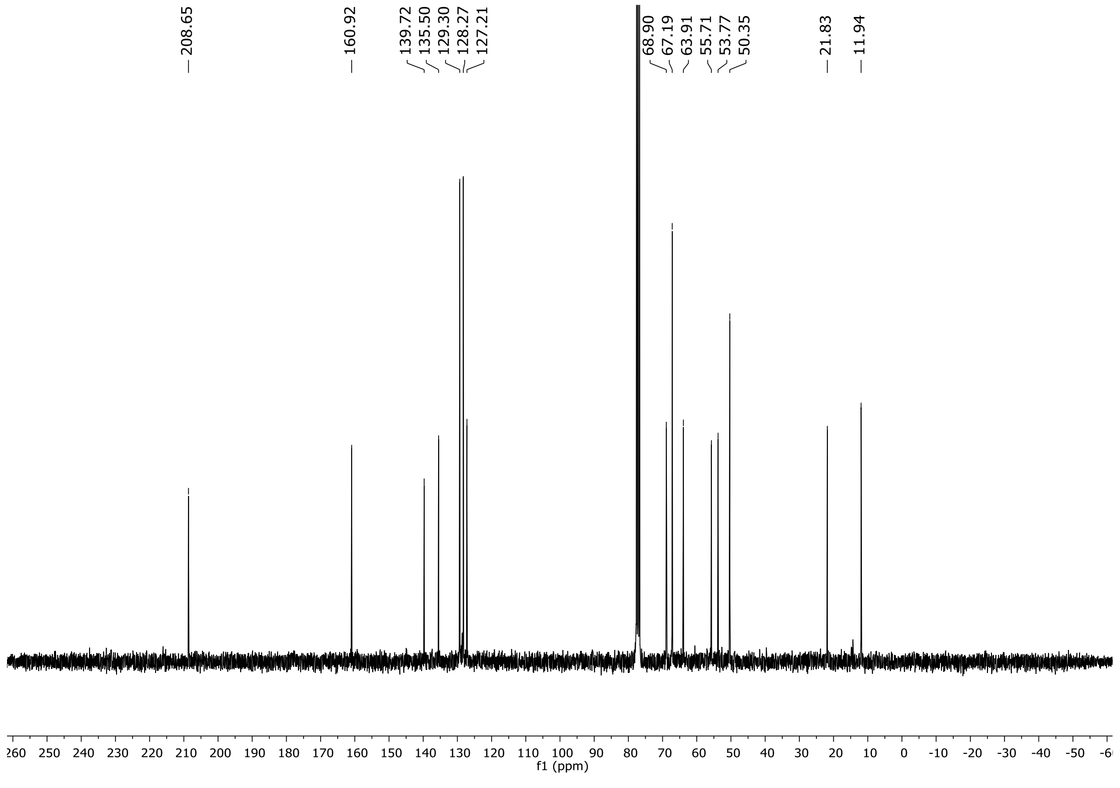

COSY and NOESY of compound **1f**

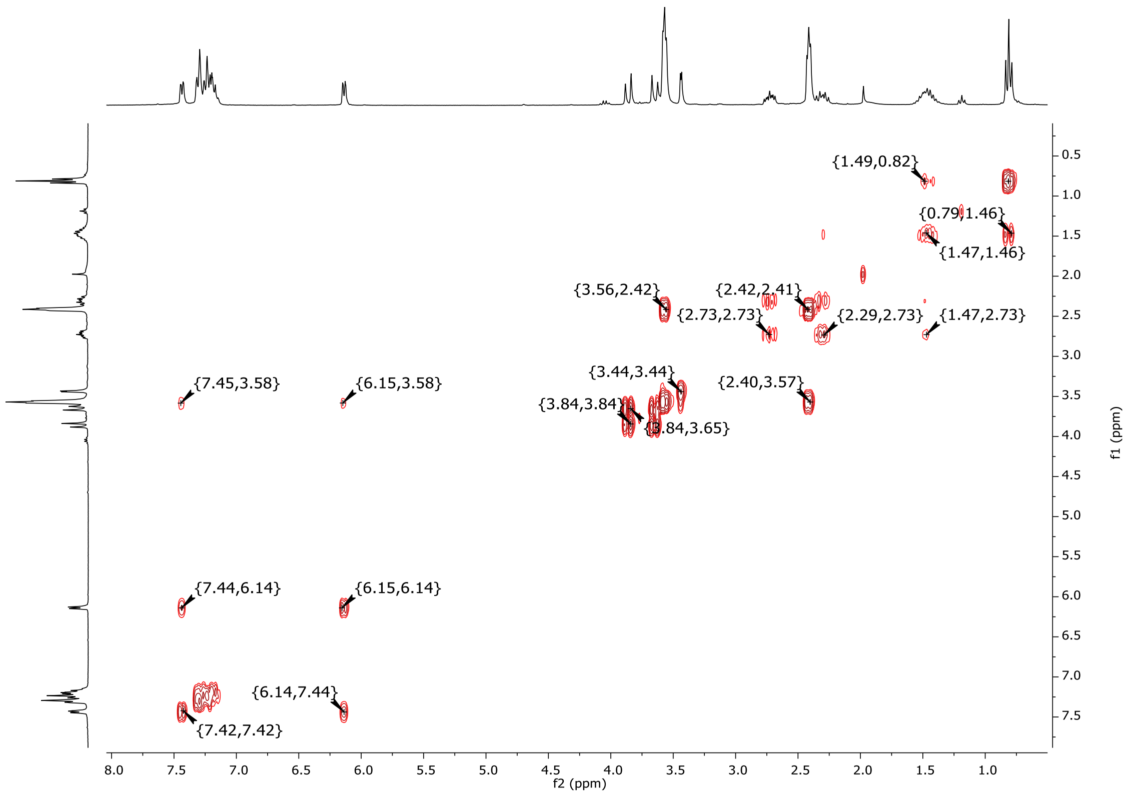

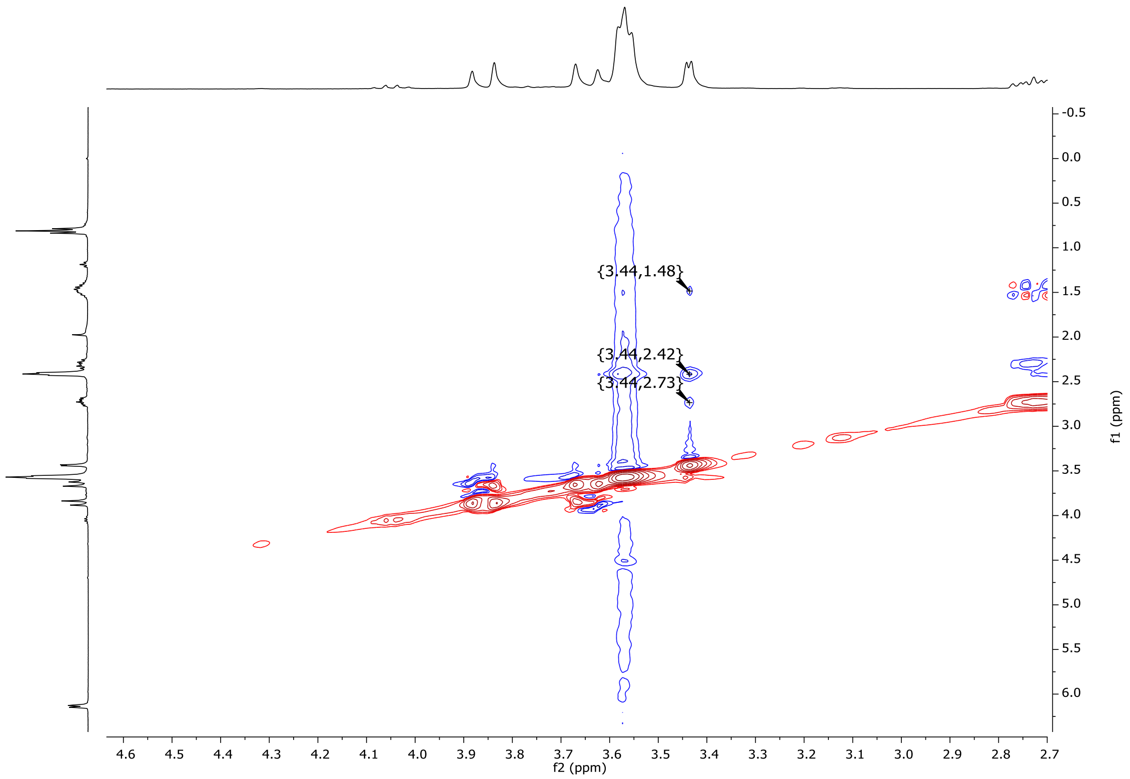

HSQC and HMBC of compound **1f**

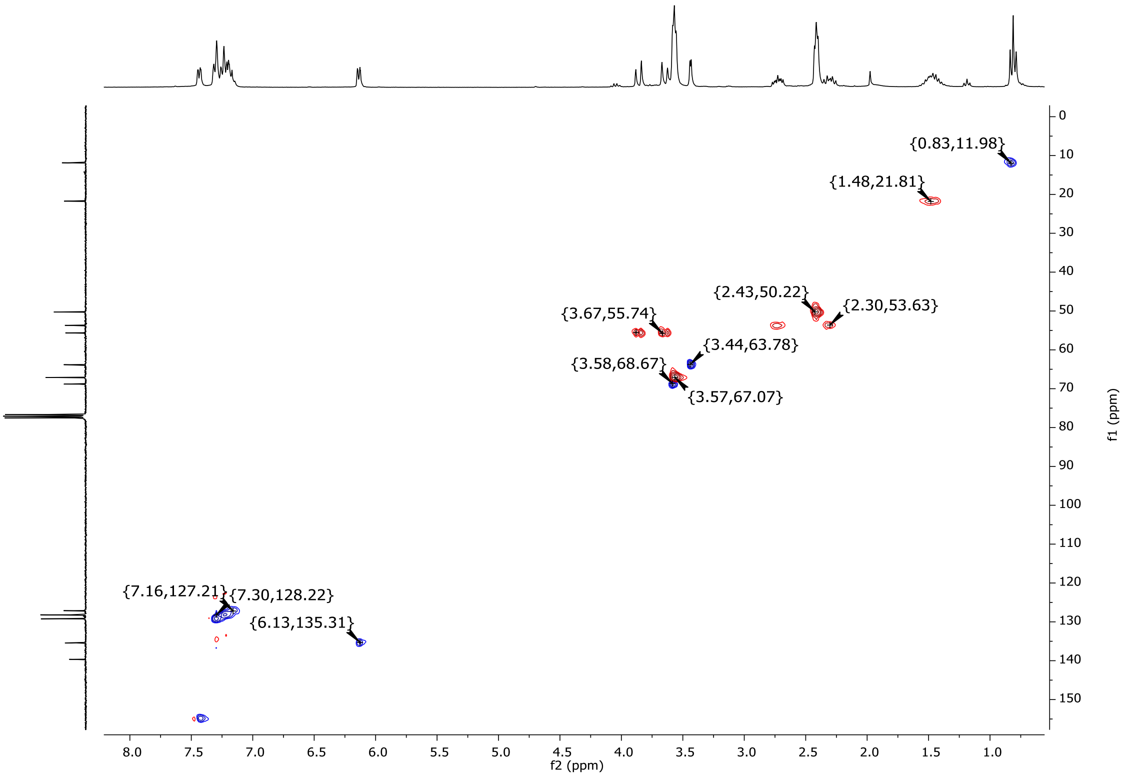

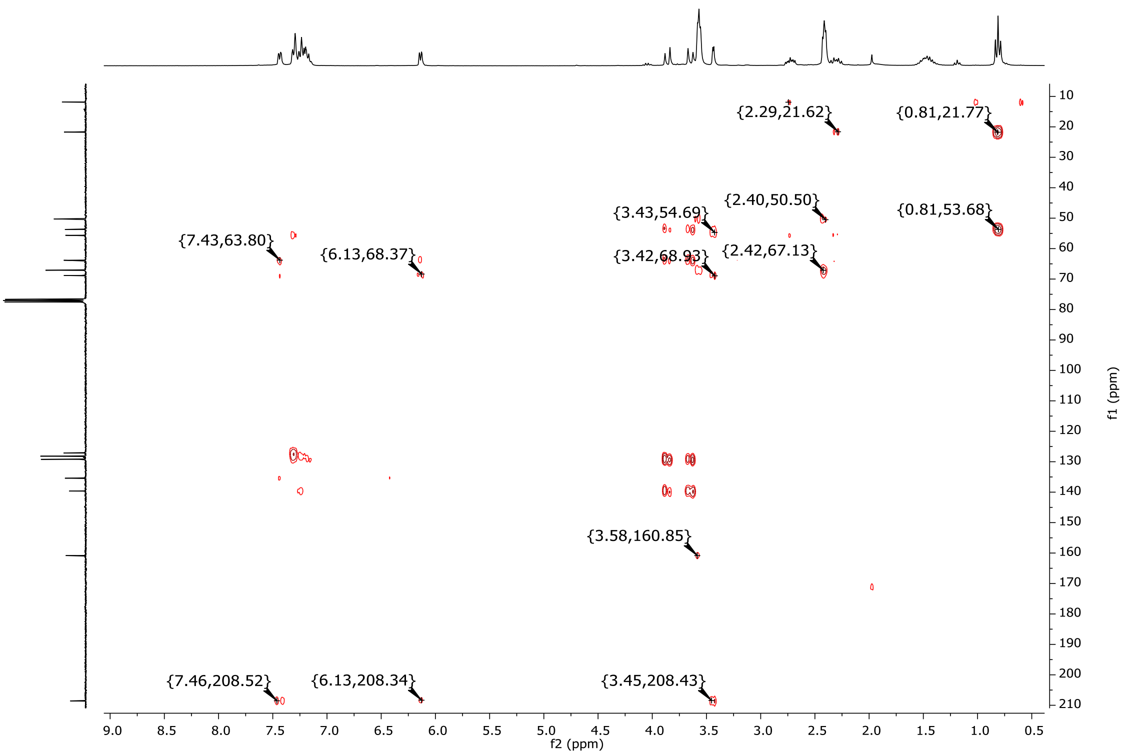

^{1}H NMR (300 MHz, $CDCl_3$) and ^{13}C NMR (75 MHz, $CDCl_3$) of compound **1g**

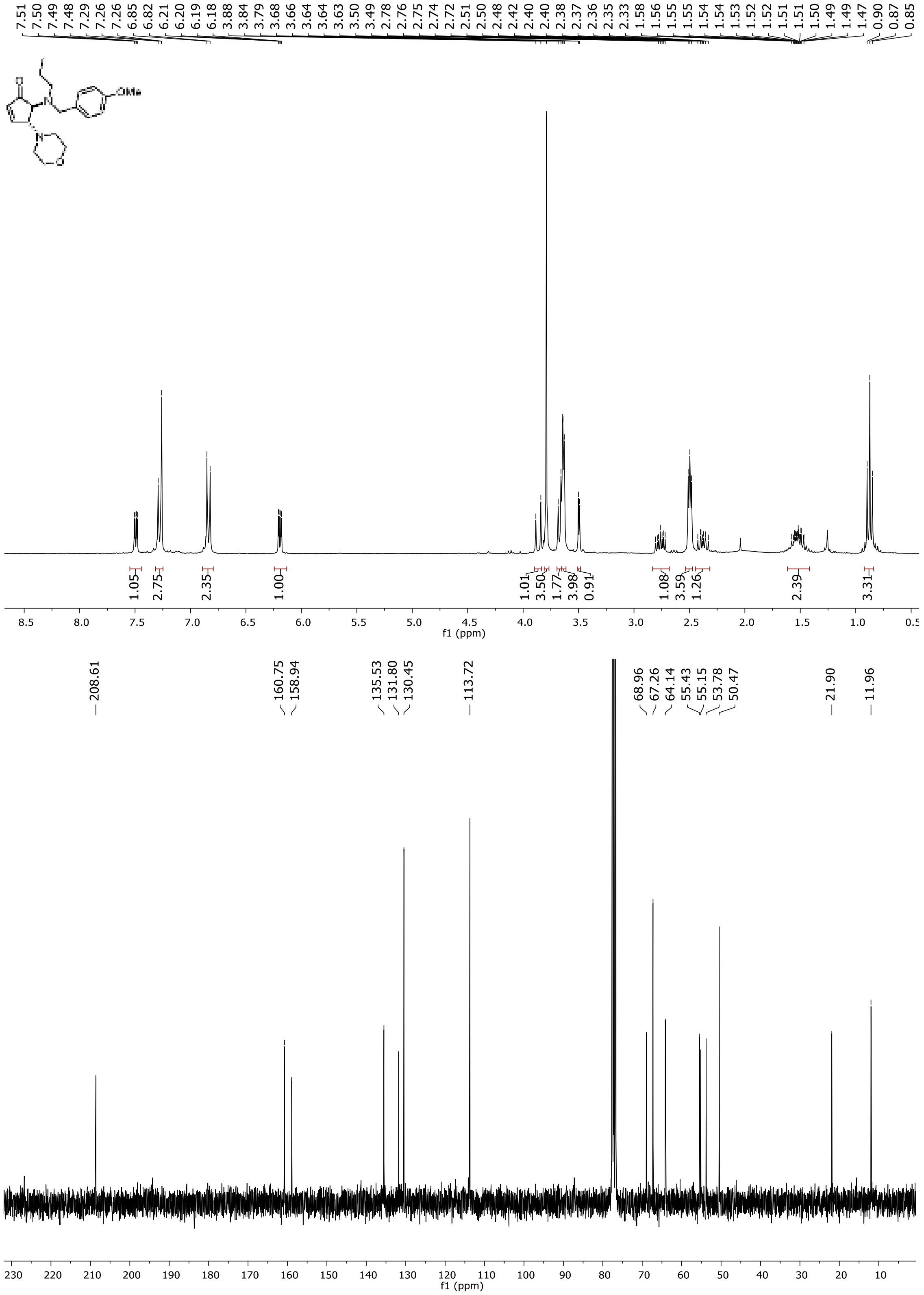

COSY and NOESY of compound **1g**

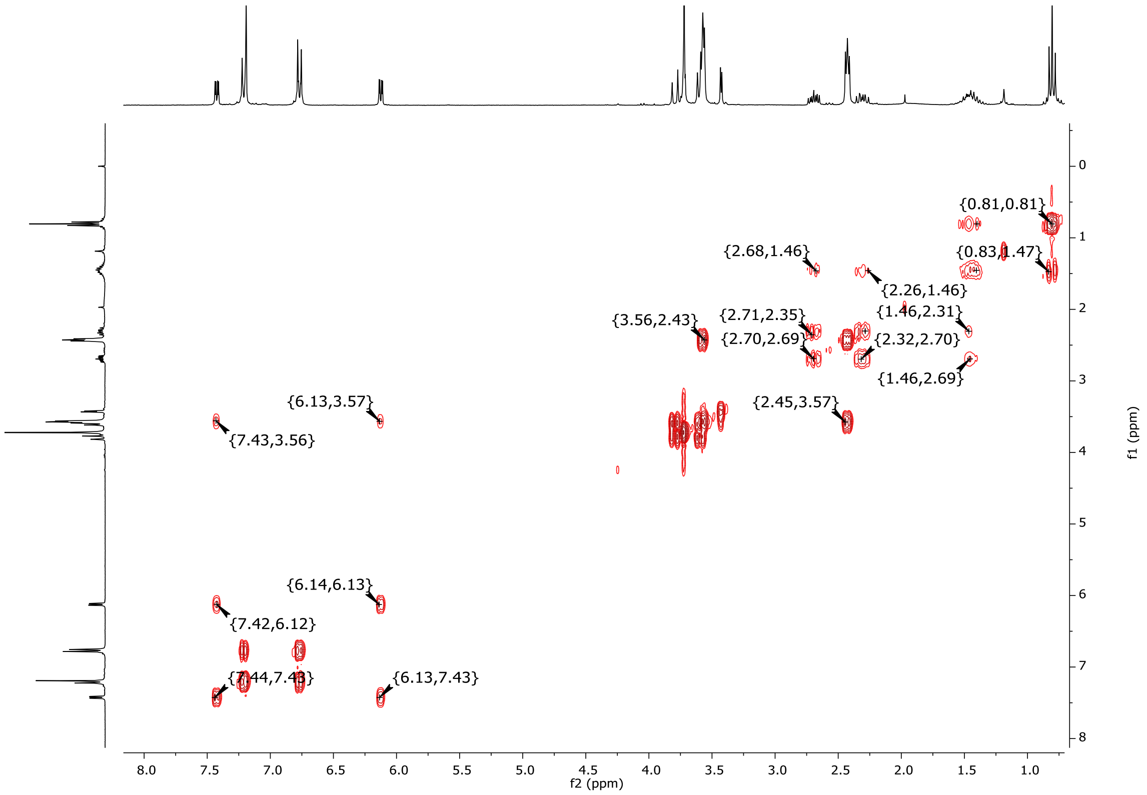

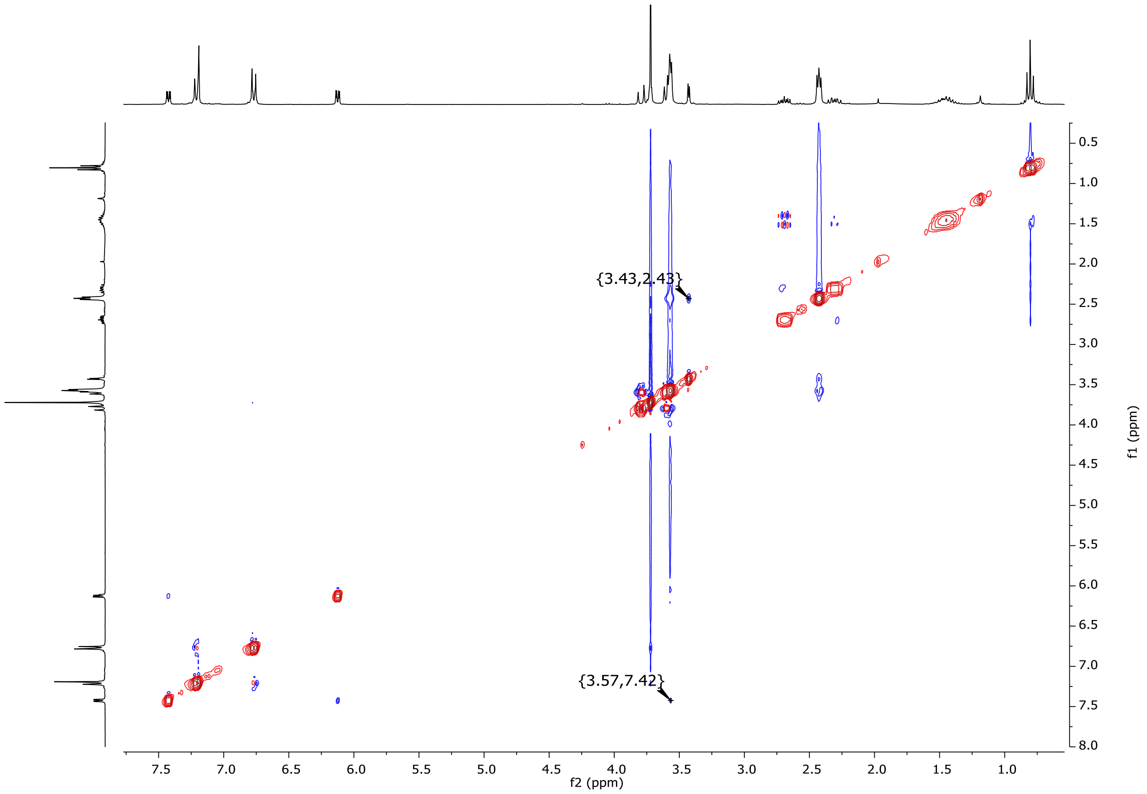

HSQC and HMBC of compound **1g**

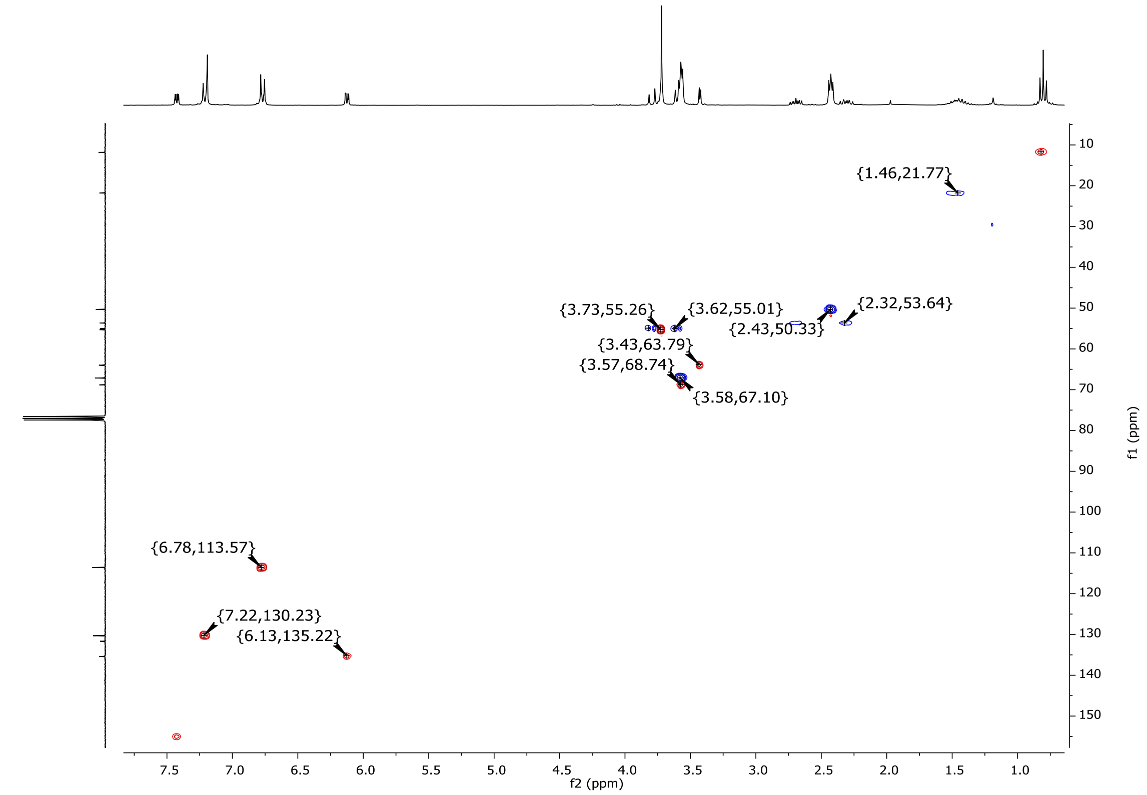

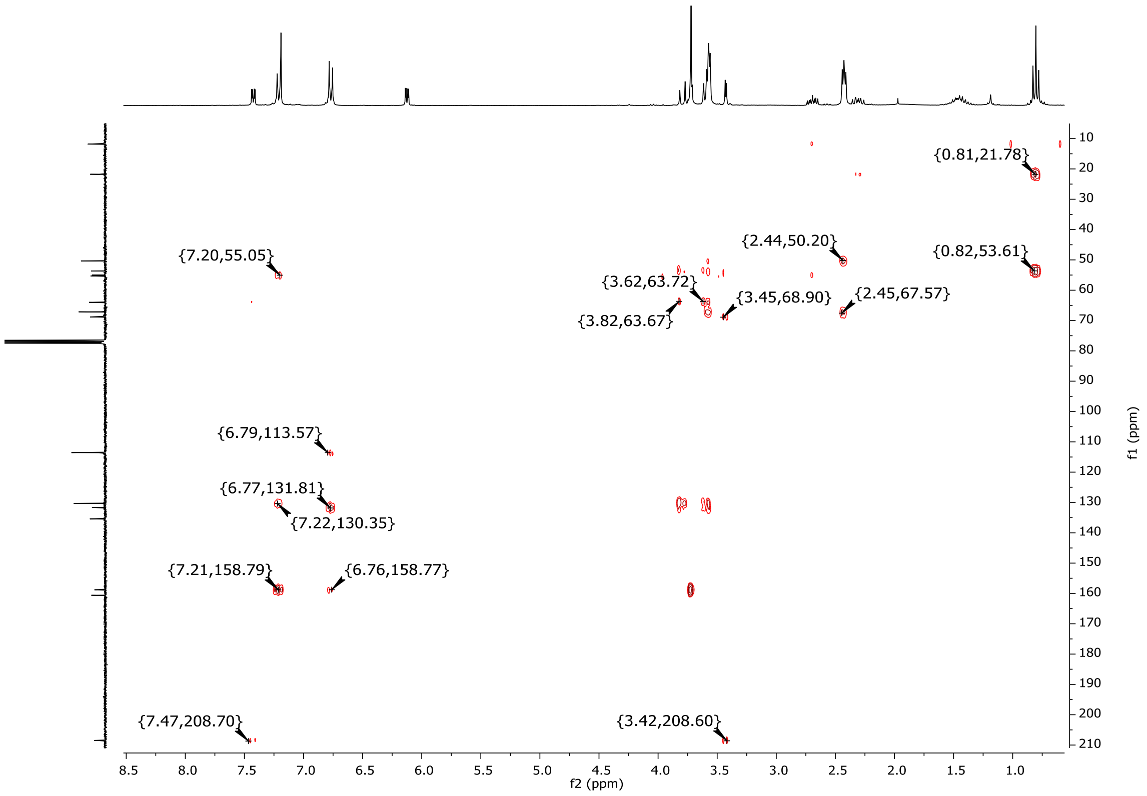

^{1}H NMR (300 MHz, $CDCl_3$) and ^{13}C NMR (75 MHz, $CDCl_3$) of compound **1h**

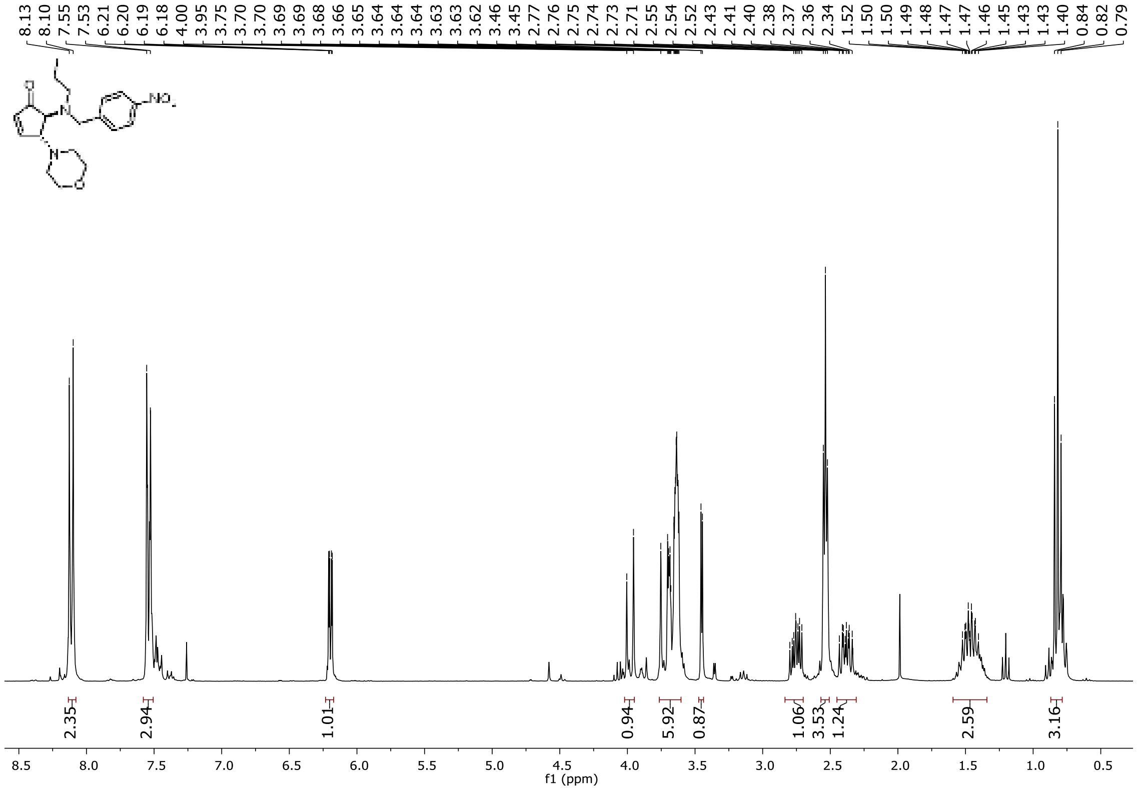

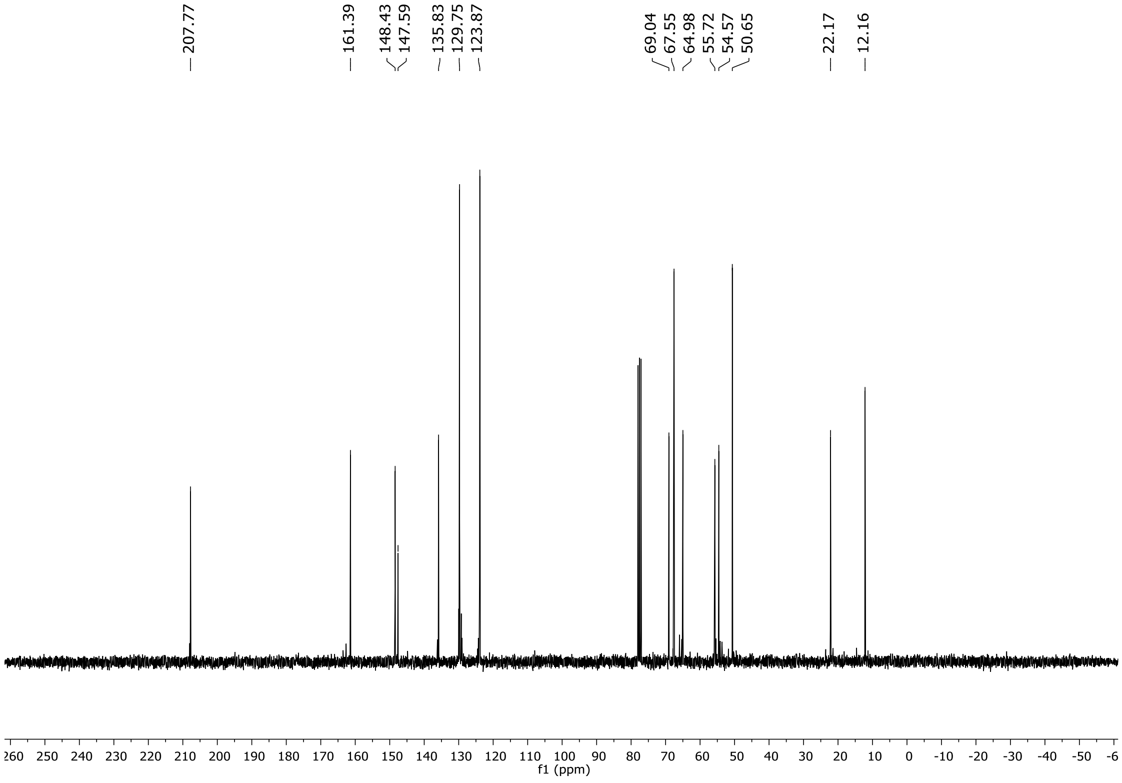

^{1}H NMR (300 MHz, $CDCl_3$) and ^{13}C NMR (75 MHz, $CDCl_3$) of compound **1i**

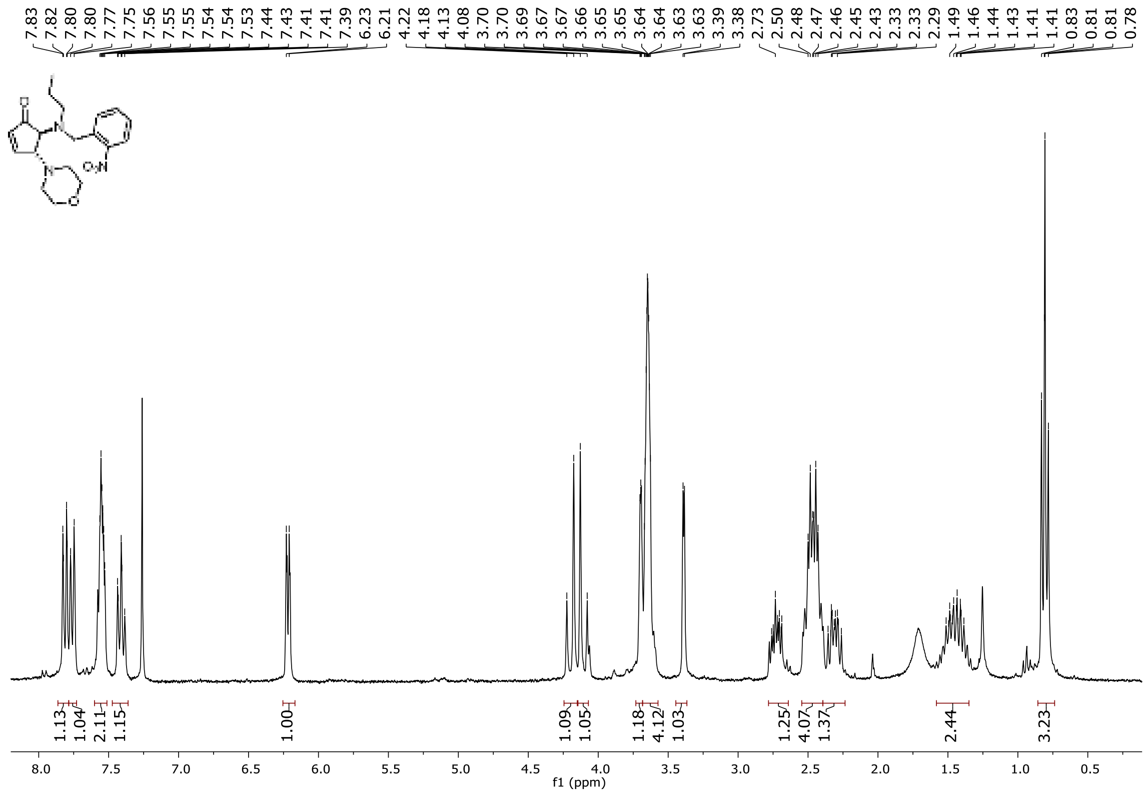

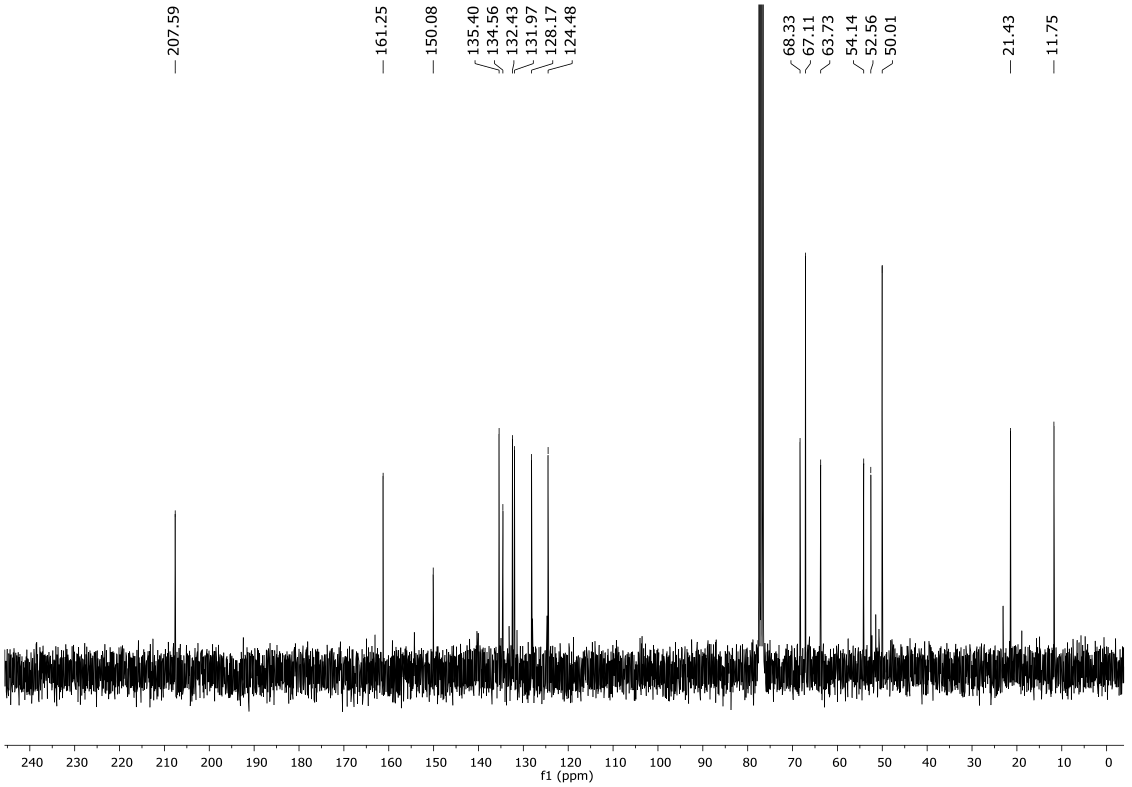

COSY and HSQC of compound **1i**

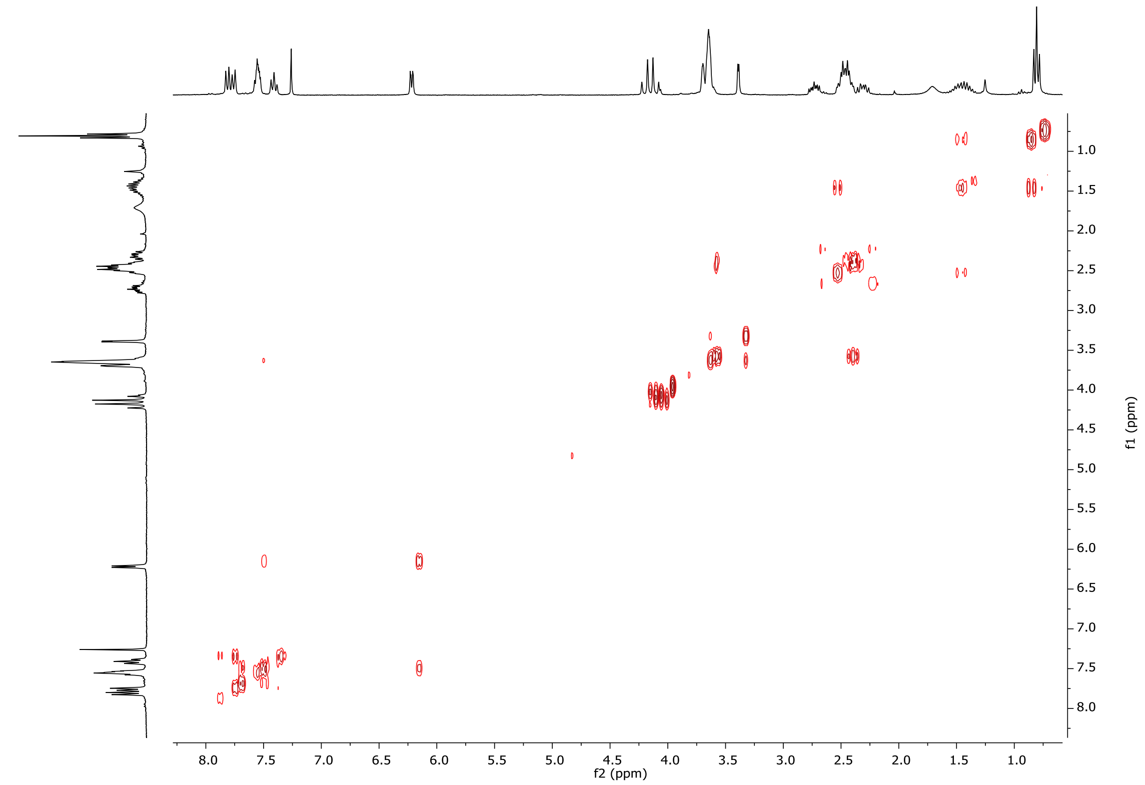

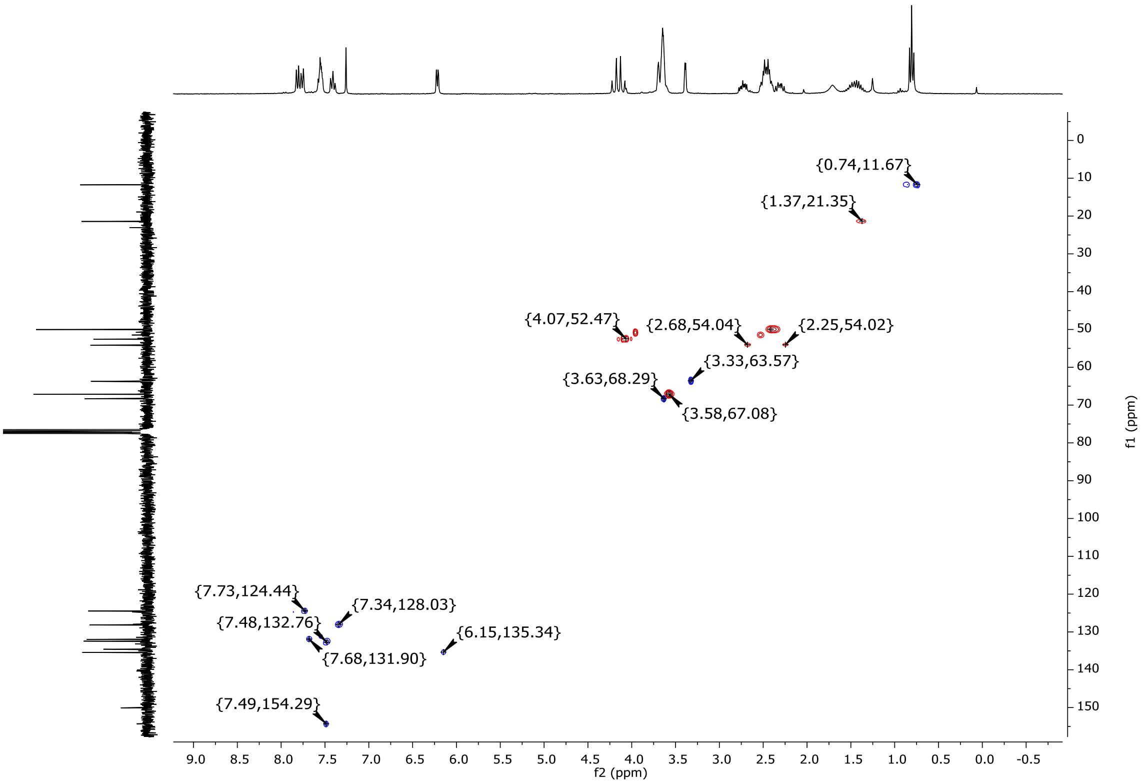

HMBC of compound **1i**

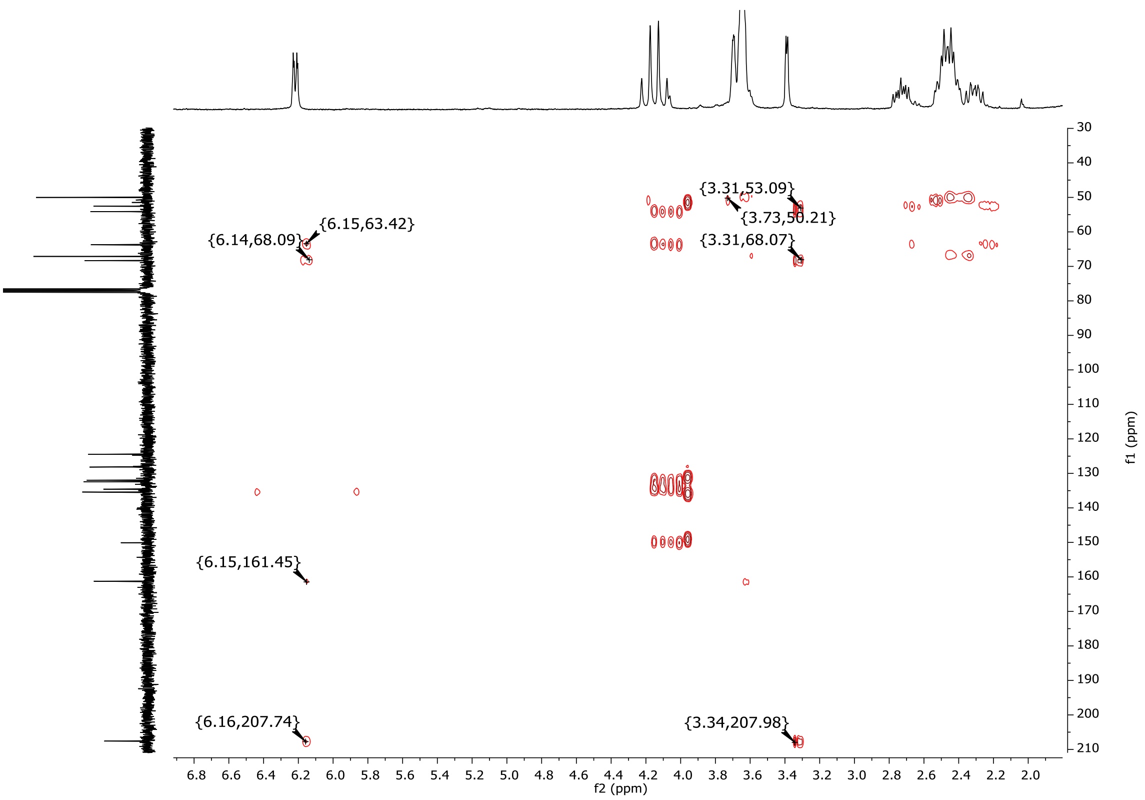

^{1}H NMR (300 MHz, $CDCl_3$) and ^{13}C NMR (75 MHz, $CDCl_3$) of compound **1j**

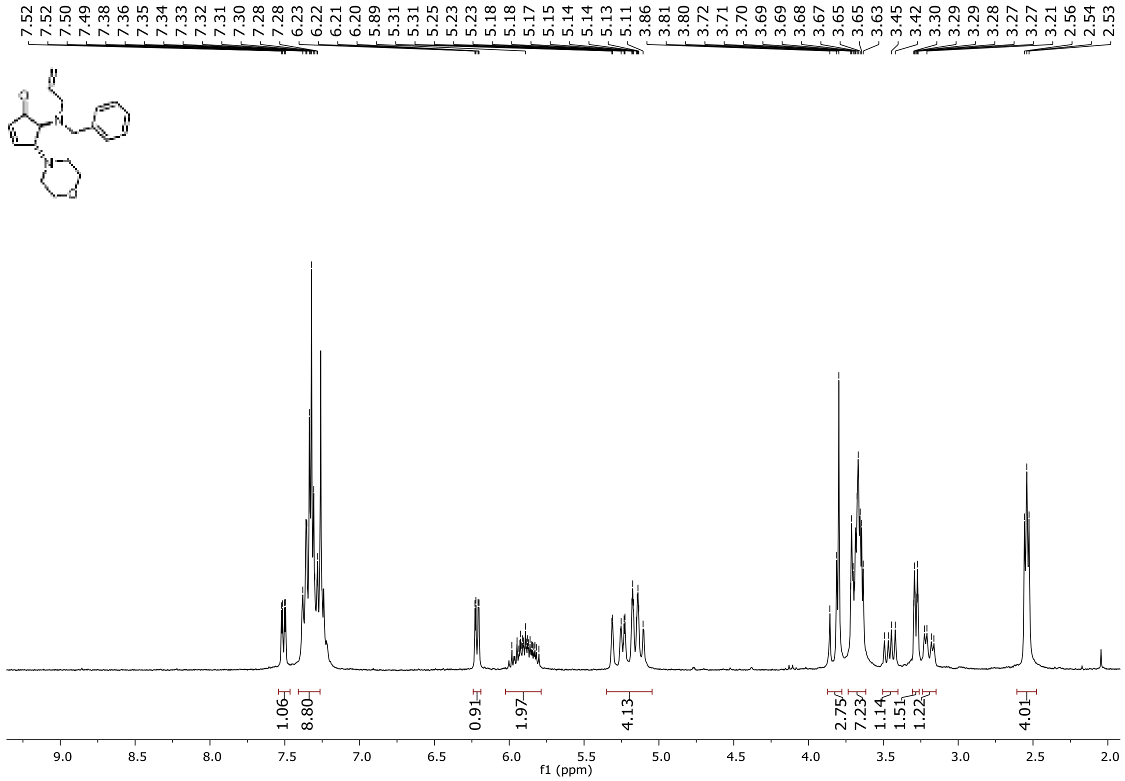

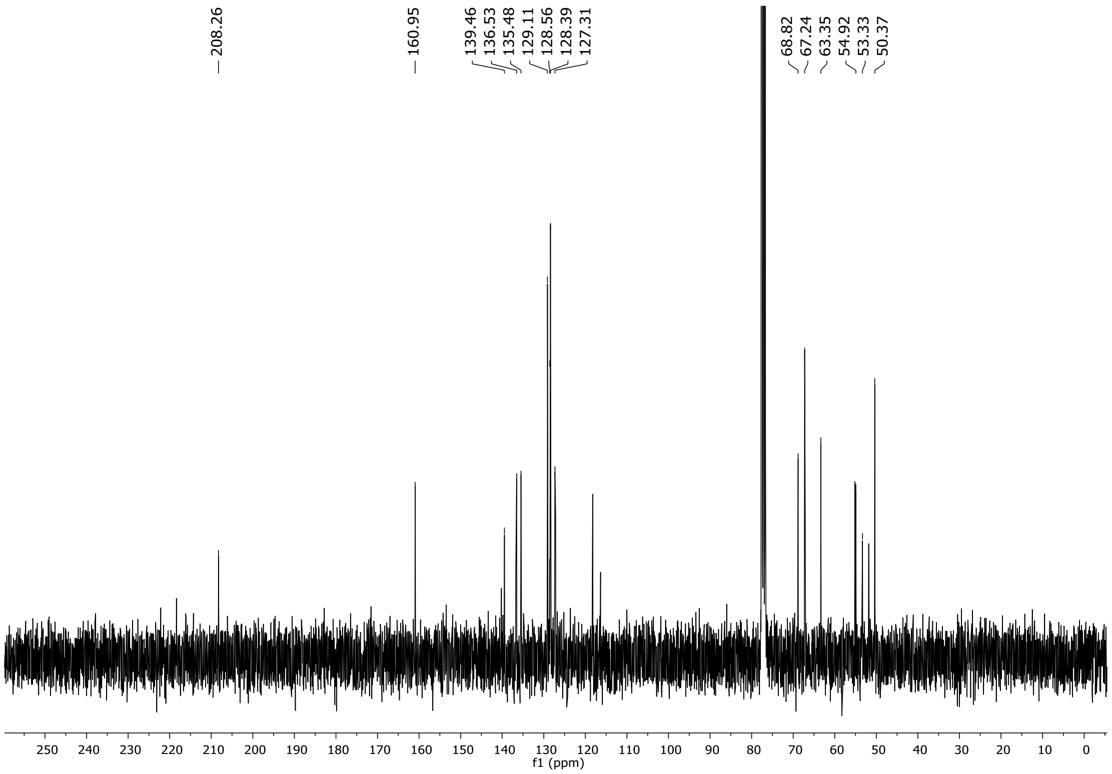

^{1}H NMR (300 MHz, $CDCl_3$) and ^{13}C NMR (75 MHz, $CDCl_3$) of compound **1k**

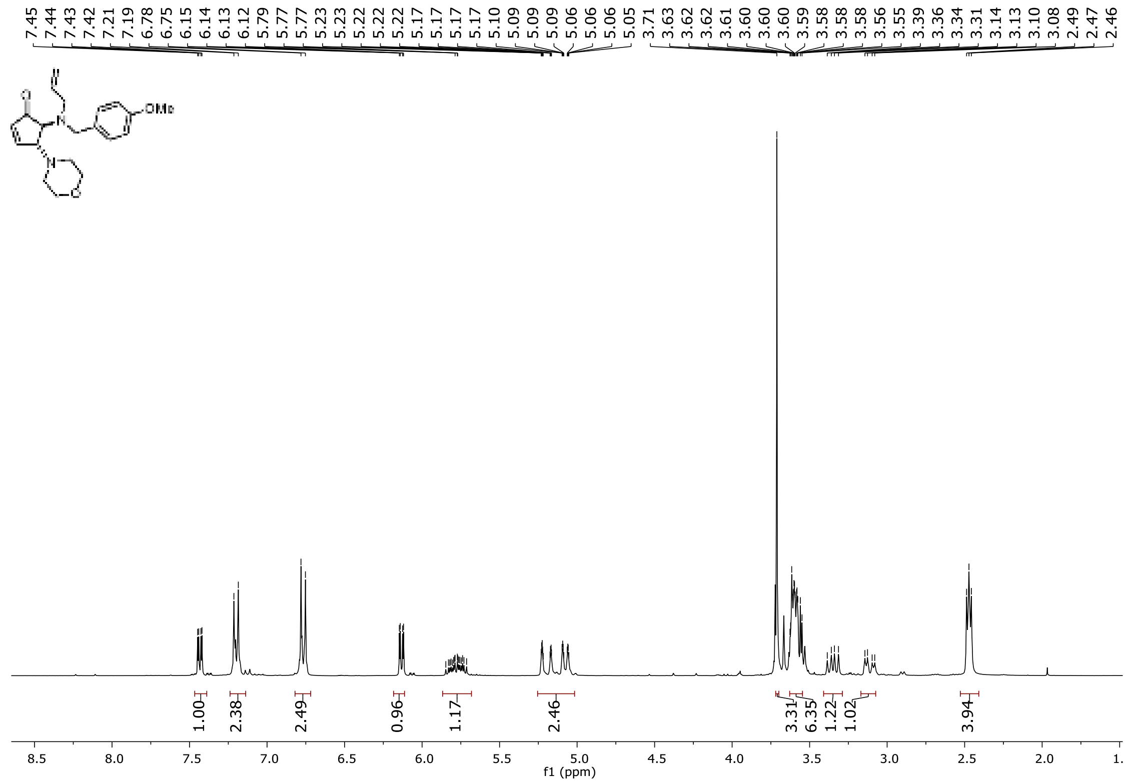

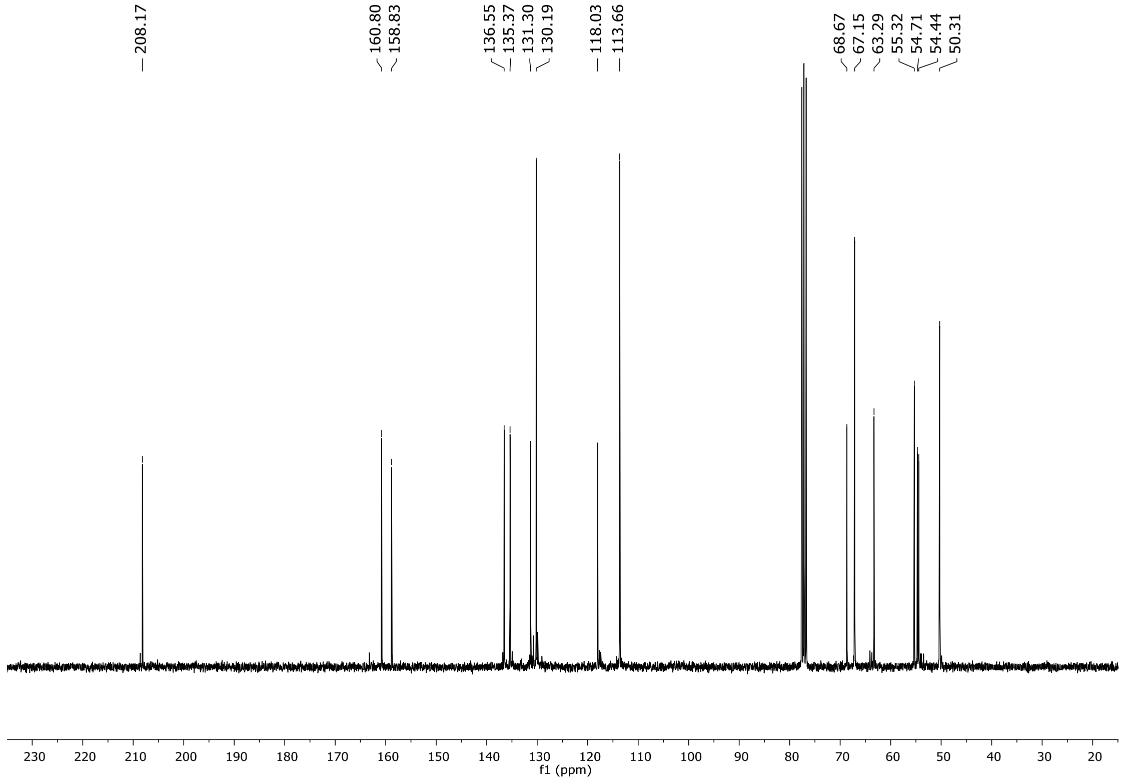

COSY of compound **1k**

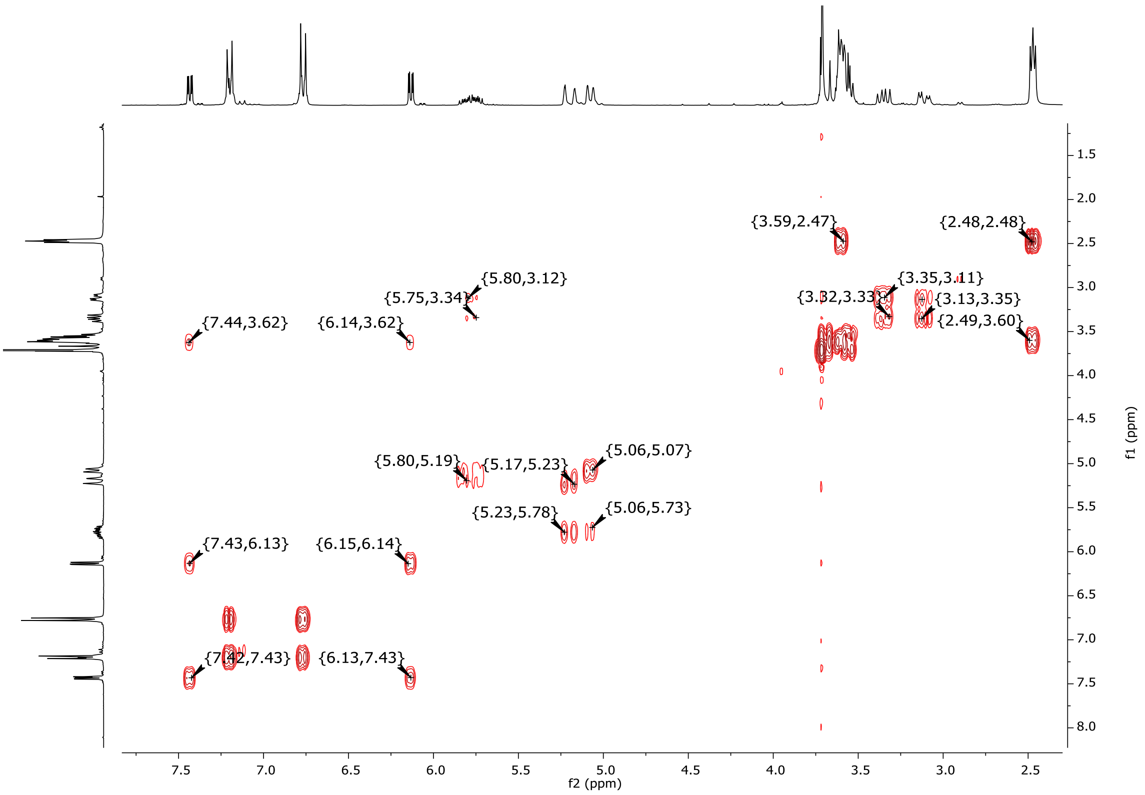

HSQC of compound **1k**

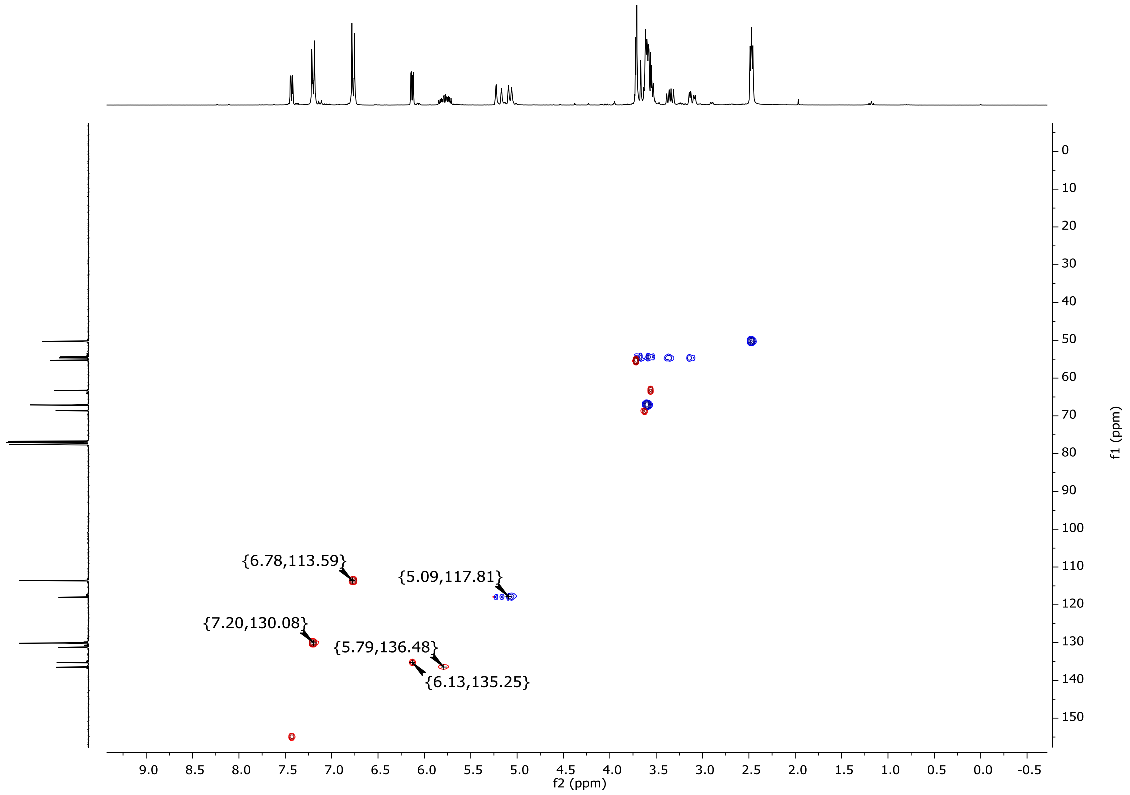

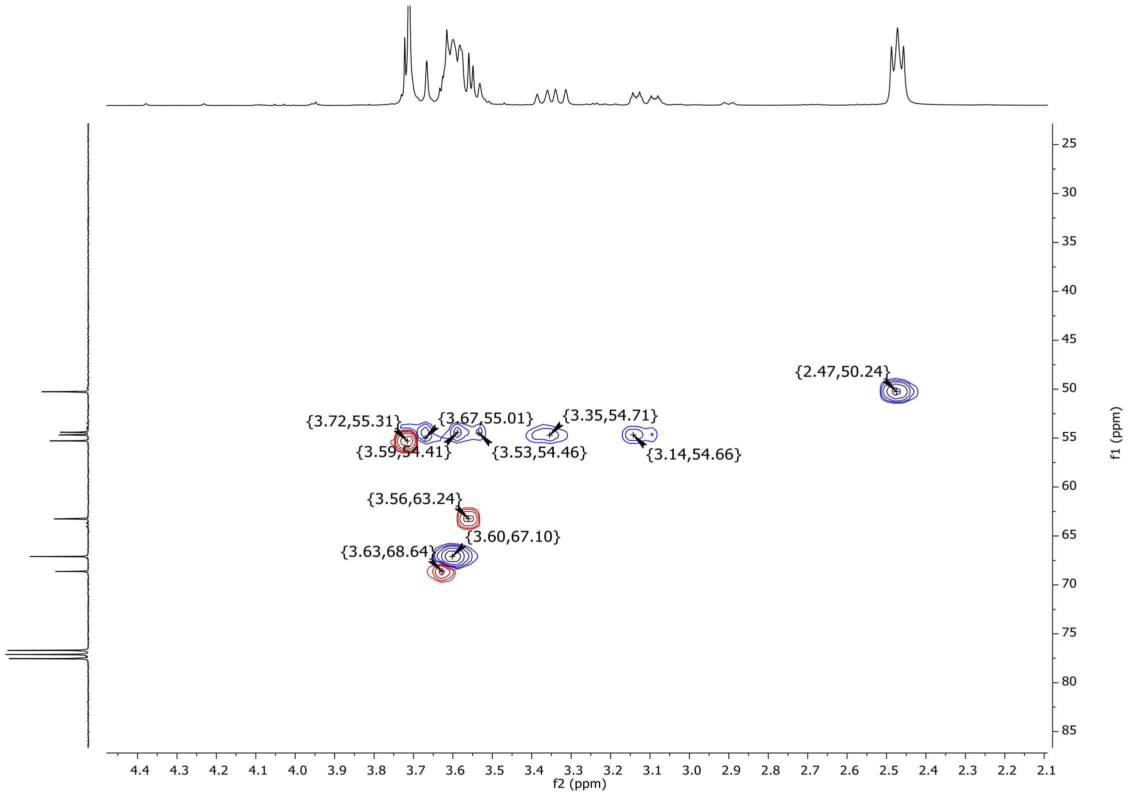

HMBC of compound **1k**

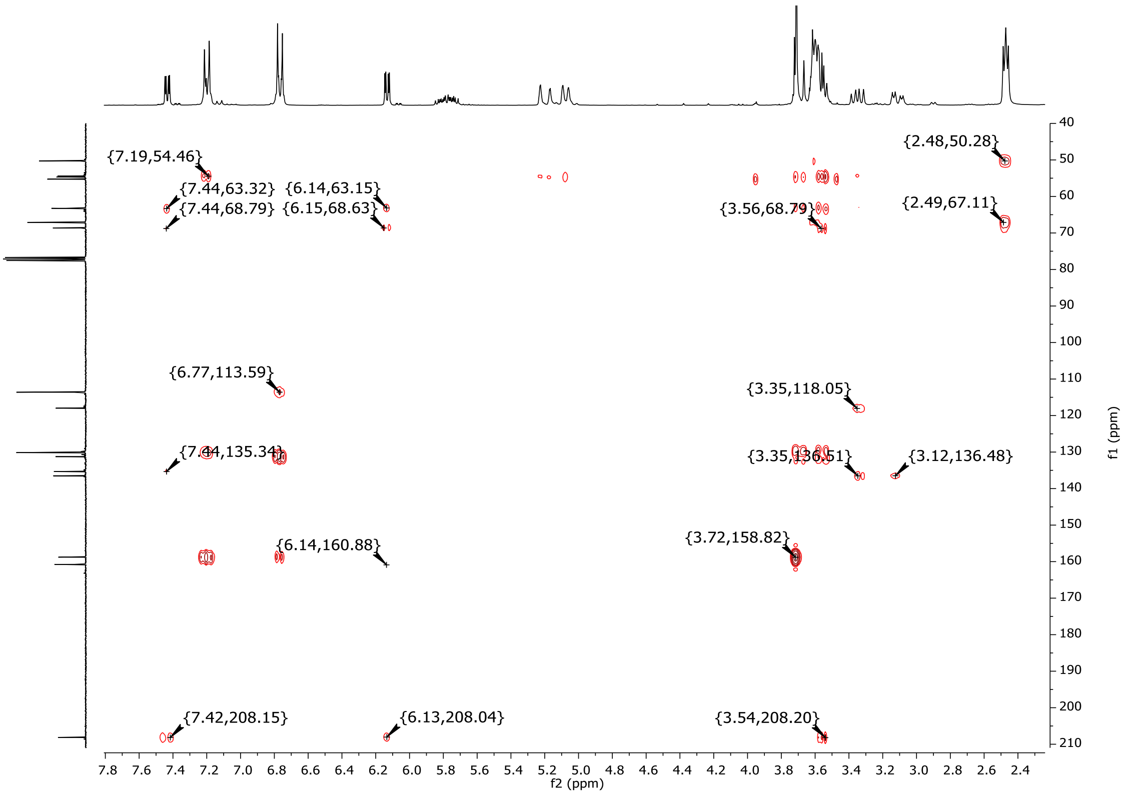

^{1}H NMR (300 MHz, $CDCl_3$) and ^{13}C NMR (75 MHz, $CDCl_3$) of compound **1l**

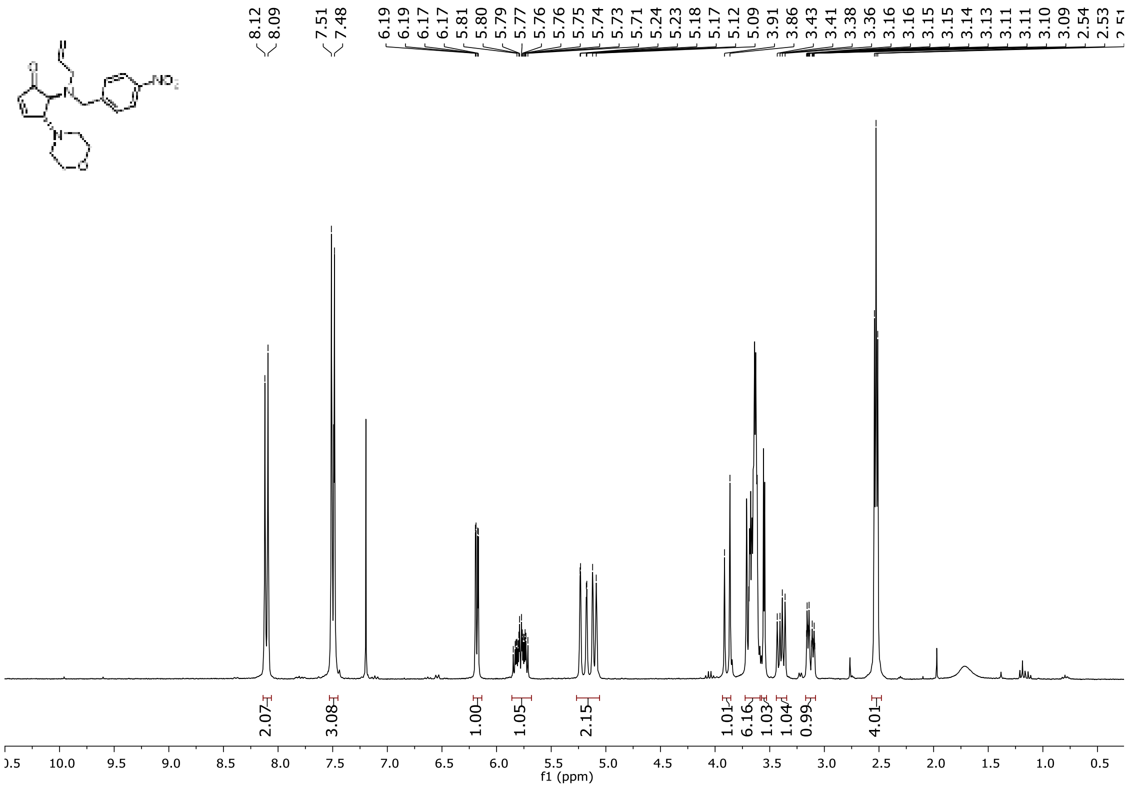

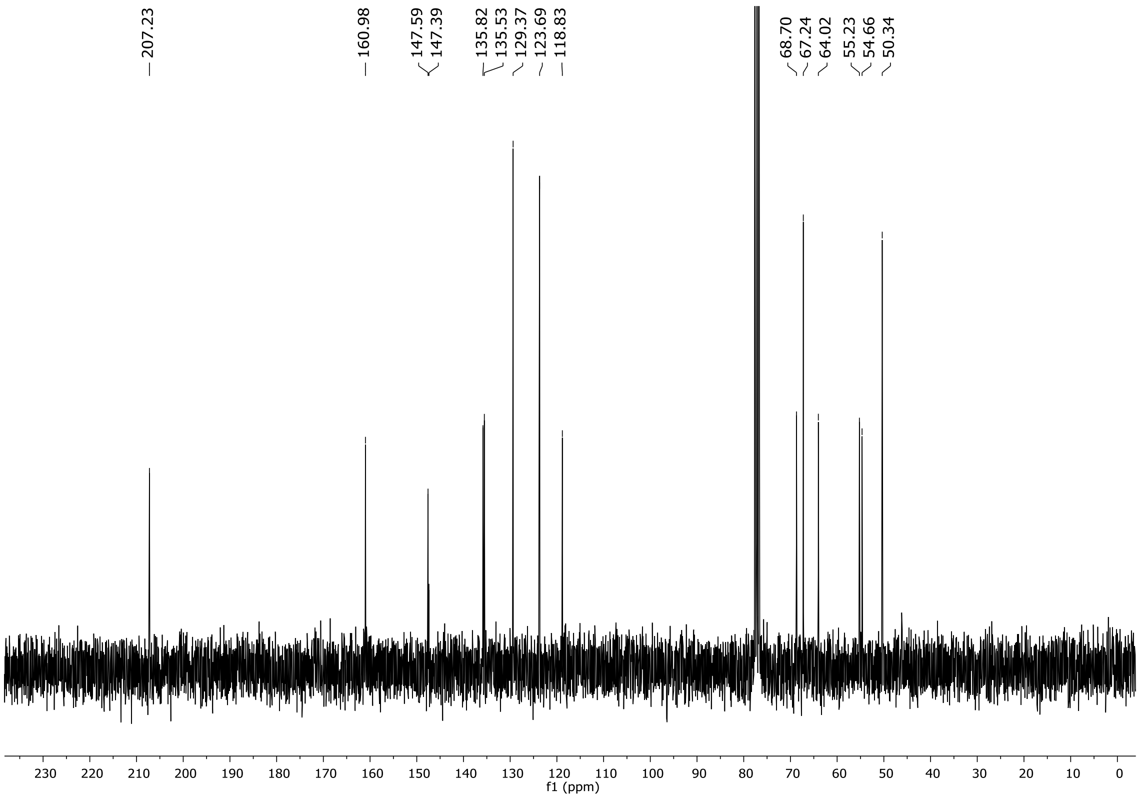

HSQC and HMBC of compound **1l**

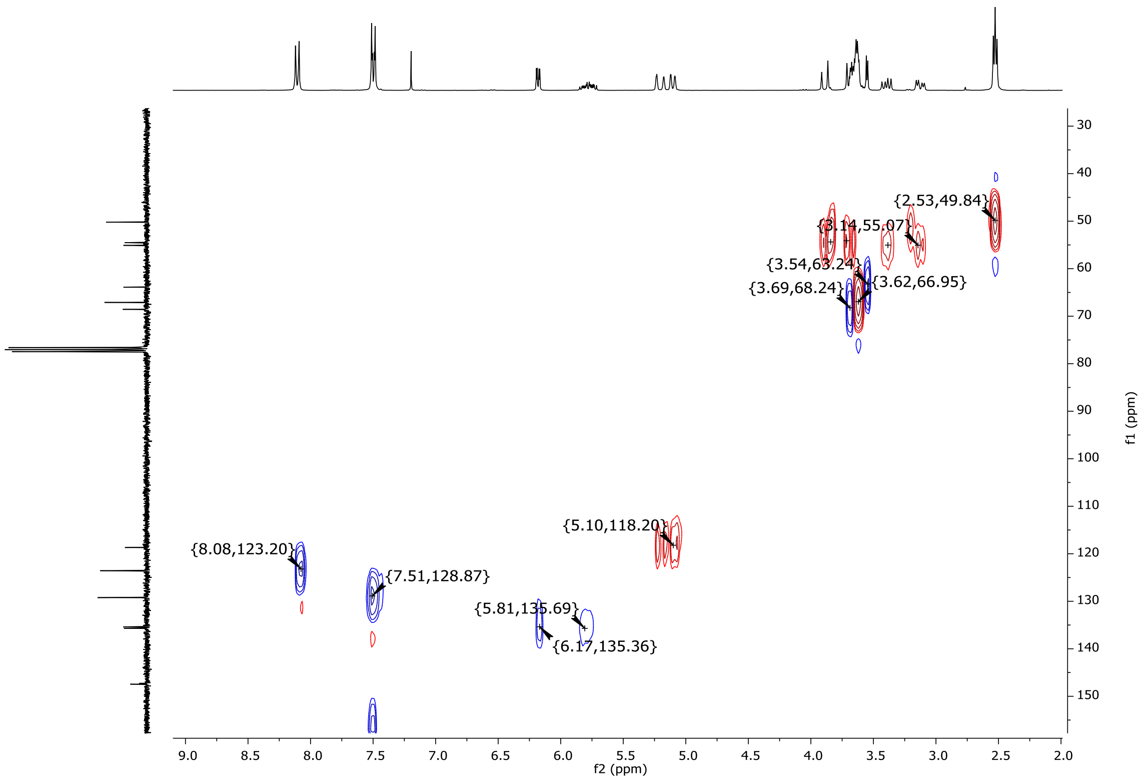

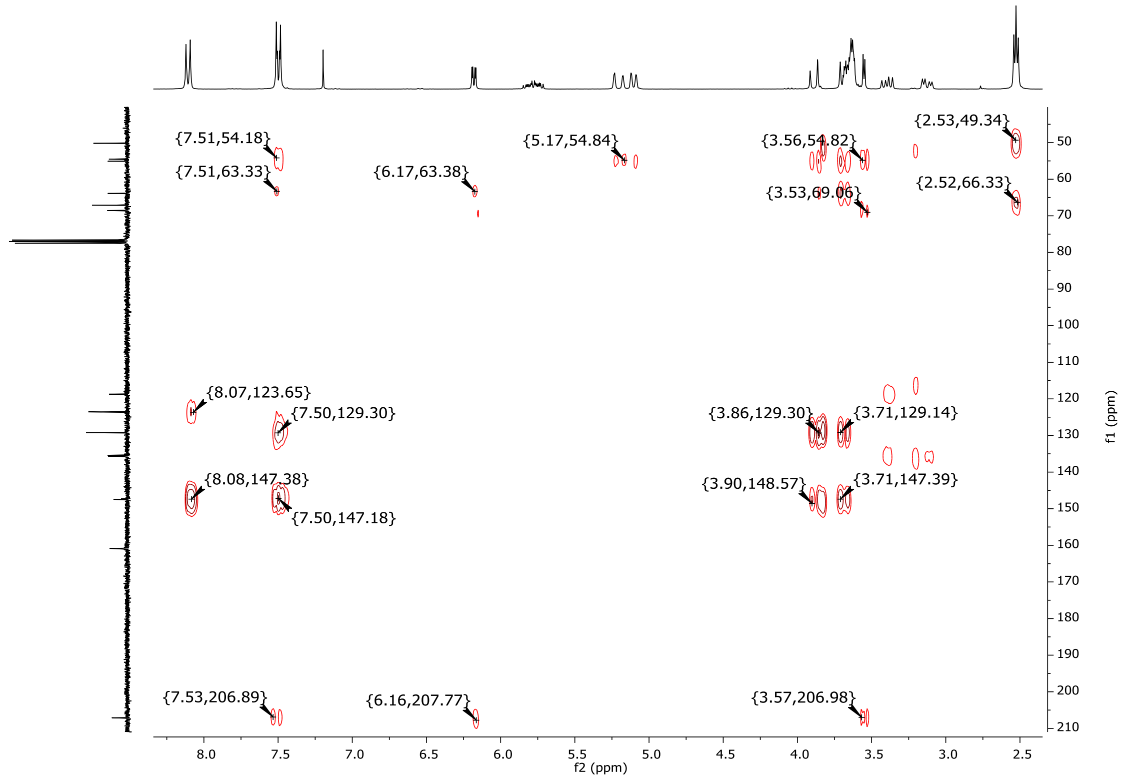

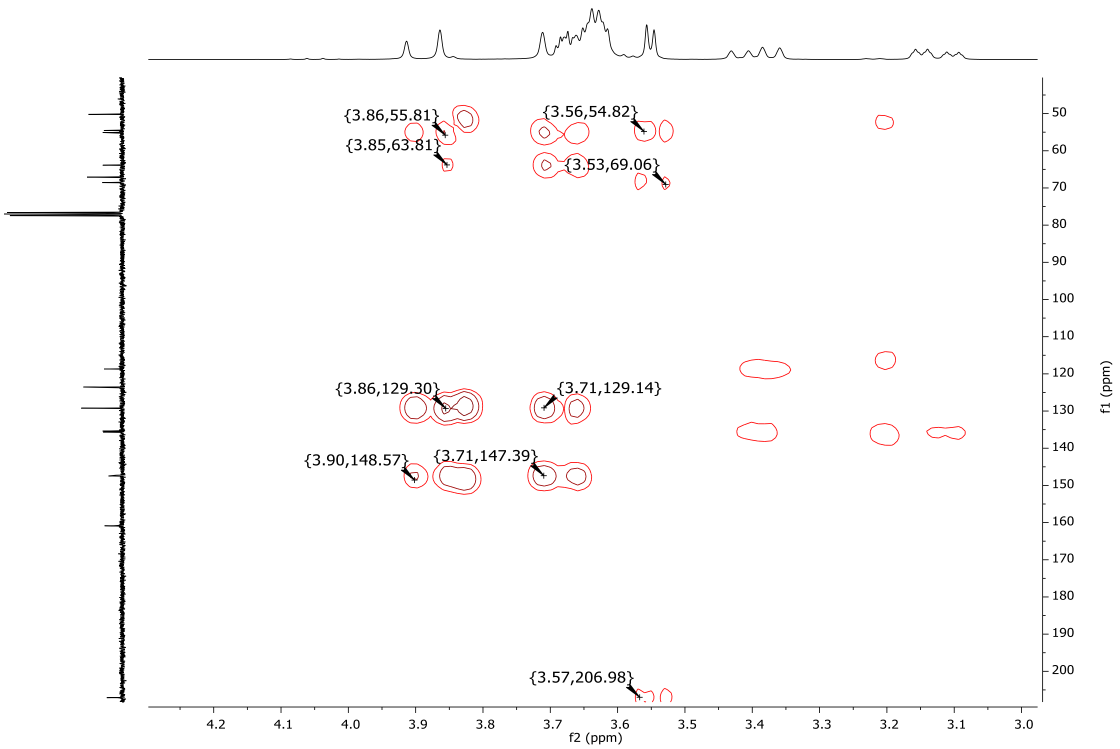
{3.86,55.81}
{3.85,63.81}
{3.56,54.82}
{3.53,69.06}
{3.86,129.30}
{3.71,129.14}
{3.90,148.57}
{3.71,147.39}
{3.57,206.98}
f2 (ppm)
f1 (ppm)

^1H NMR (300 MHz, $CDCl_3$) and ^{13}C NMR (75 MHz, $CDCl_3$) of compound **1m**

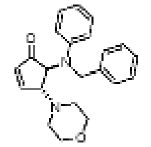

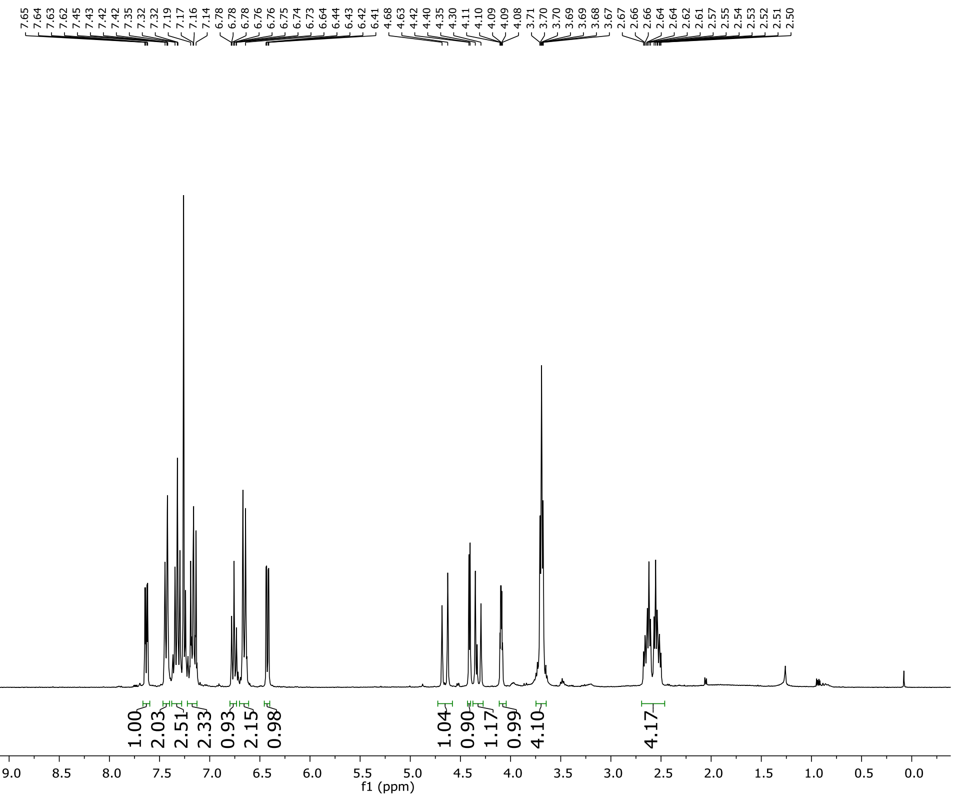

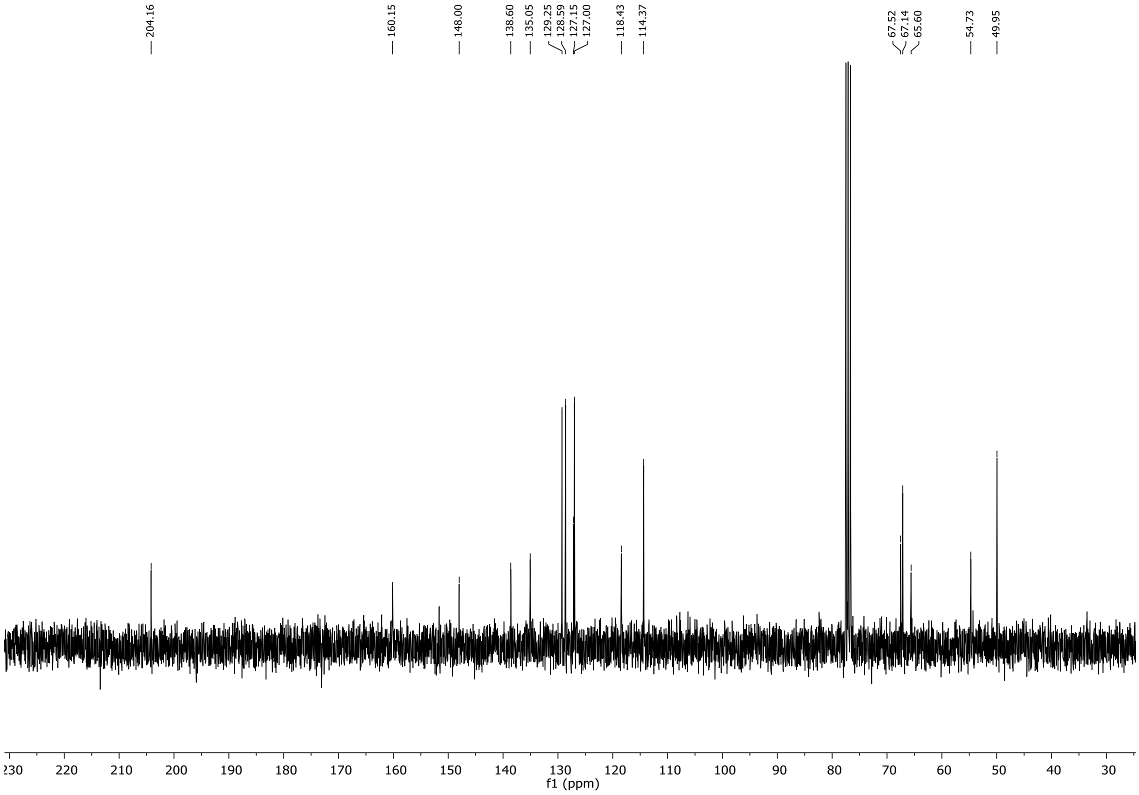

^{1}H NMR (300 MHz, $CDCl_3$) and ^{13}C NMR (75 MHz, $CDCl_3$) of compound **1n**

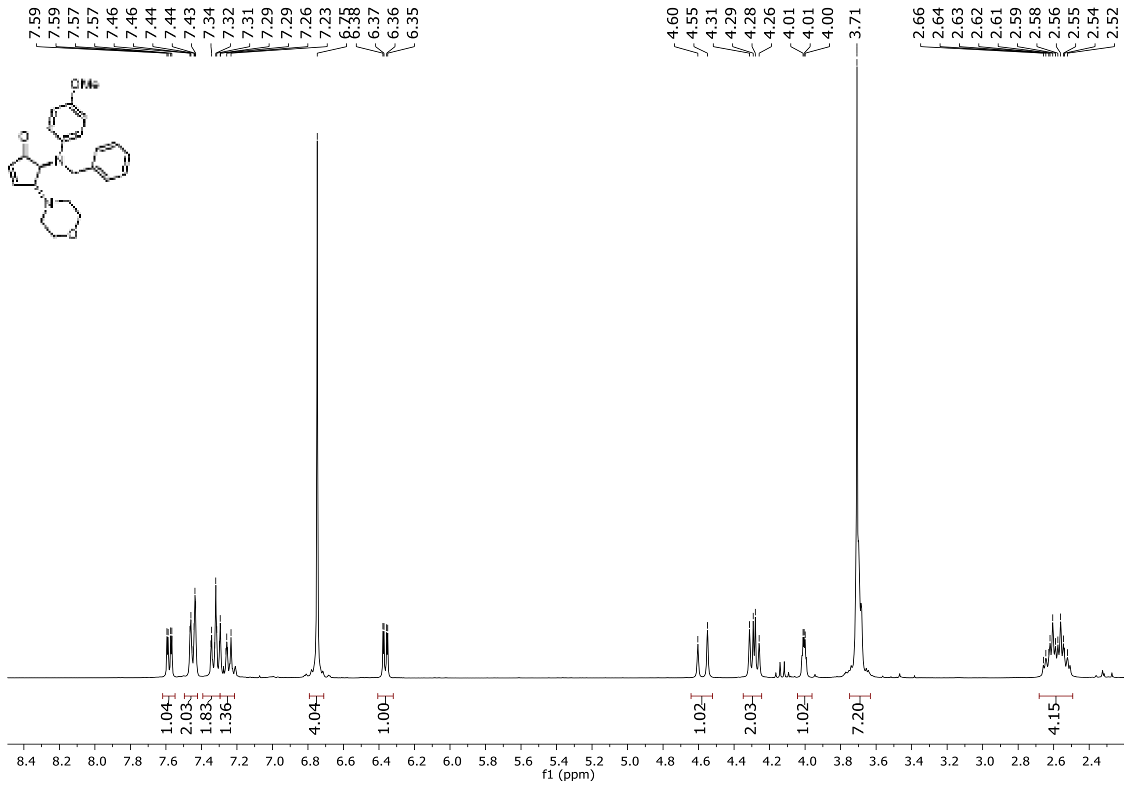

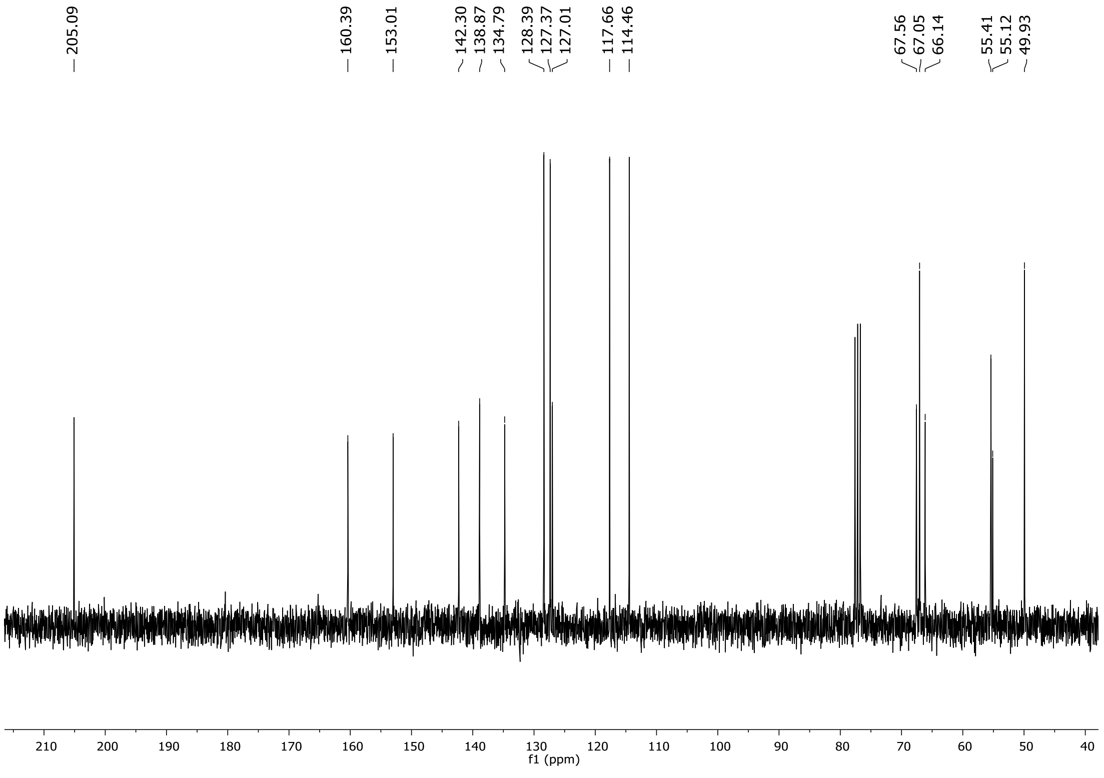

COSY and NOESY of compound **1n**

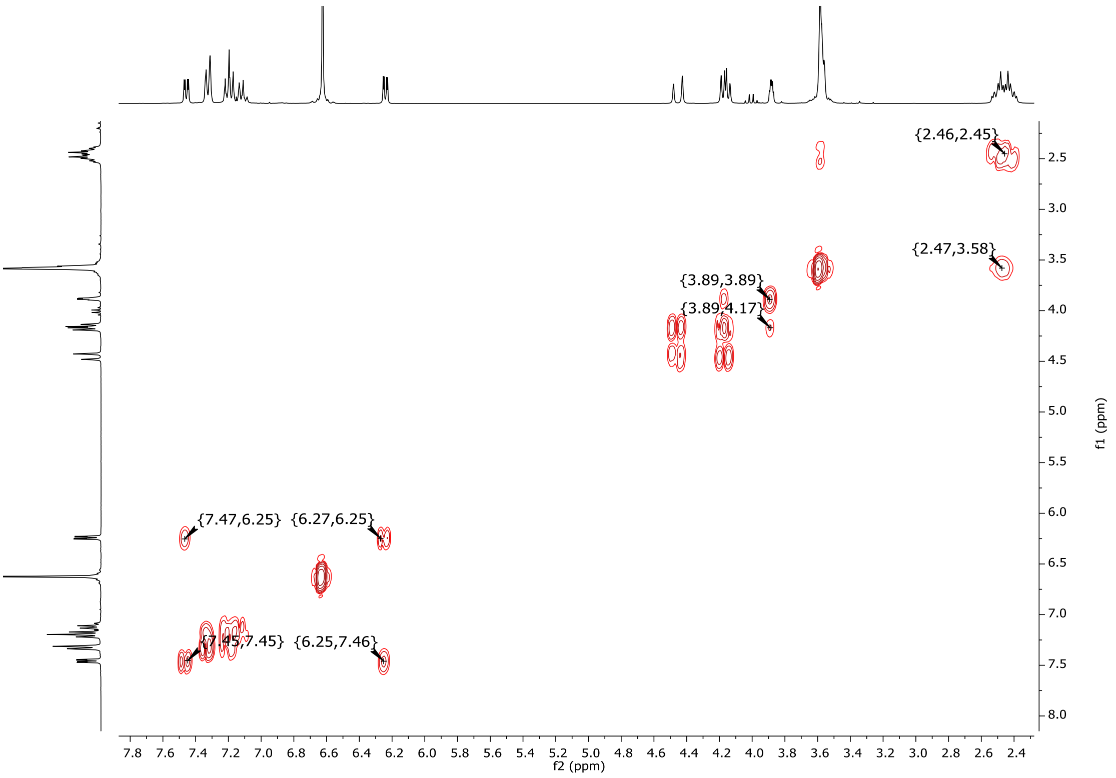

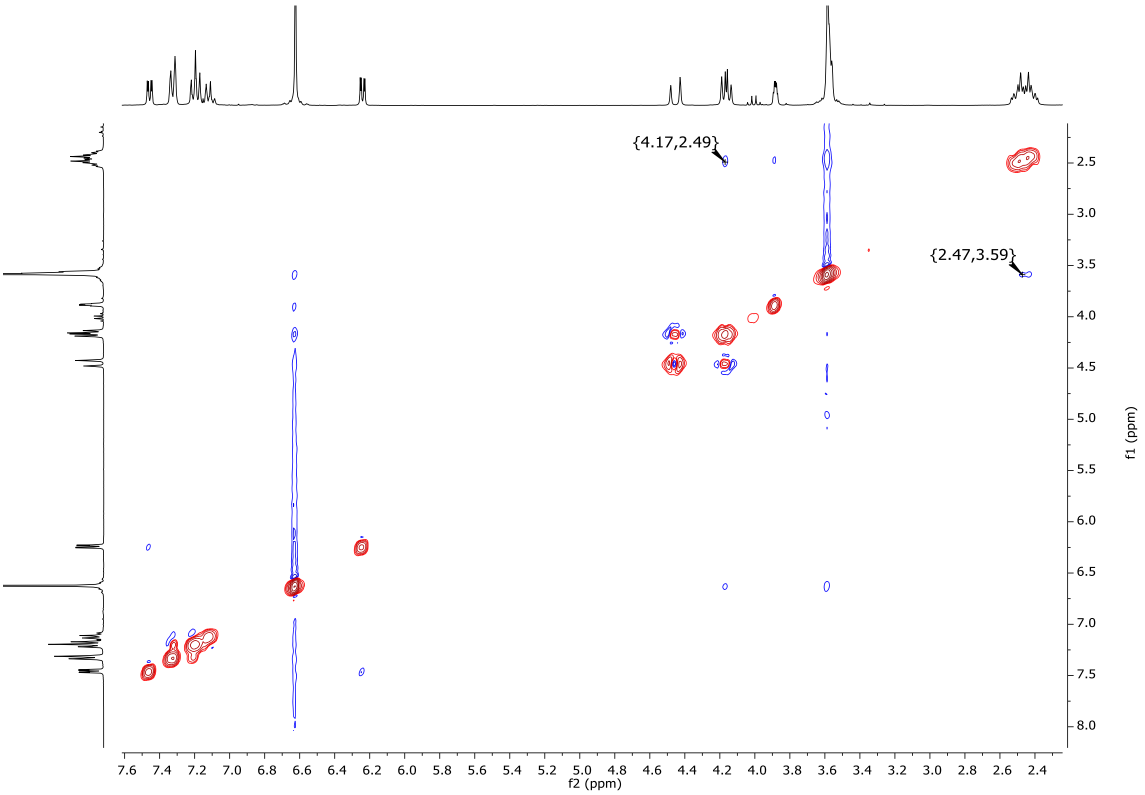

HSQC and HMBC of compound **1n**

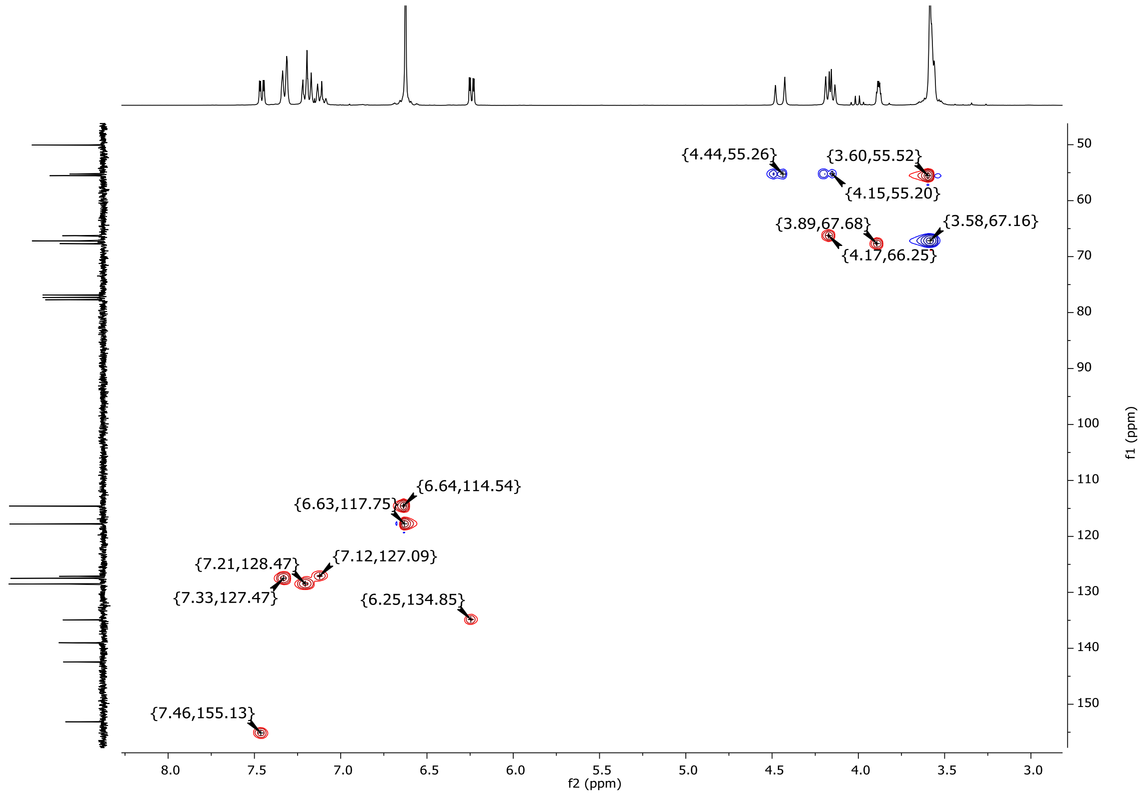

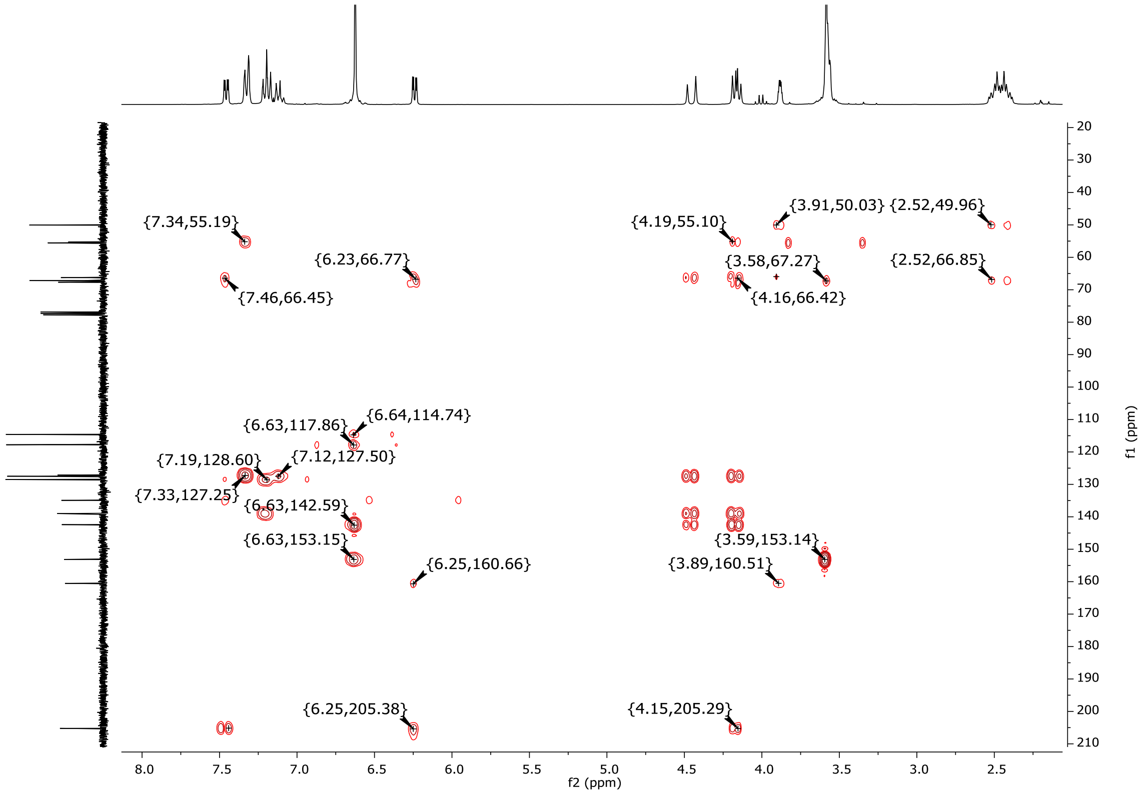

^1H NMR (300 MHz, $CDCl_3$) and ^{13}C NMR (75 MHz, $CDCl_3$) of compound **1n’**

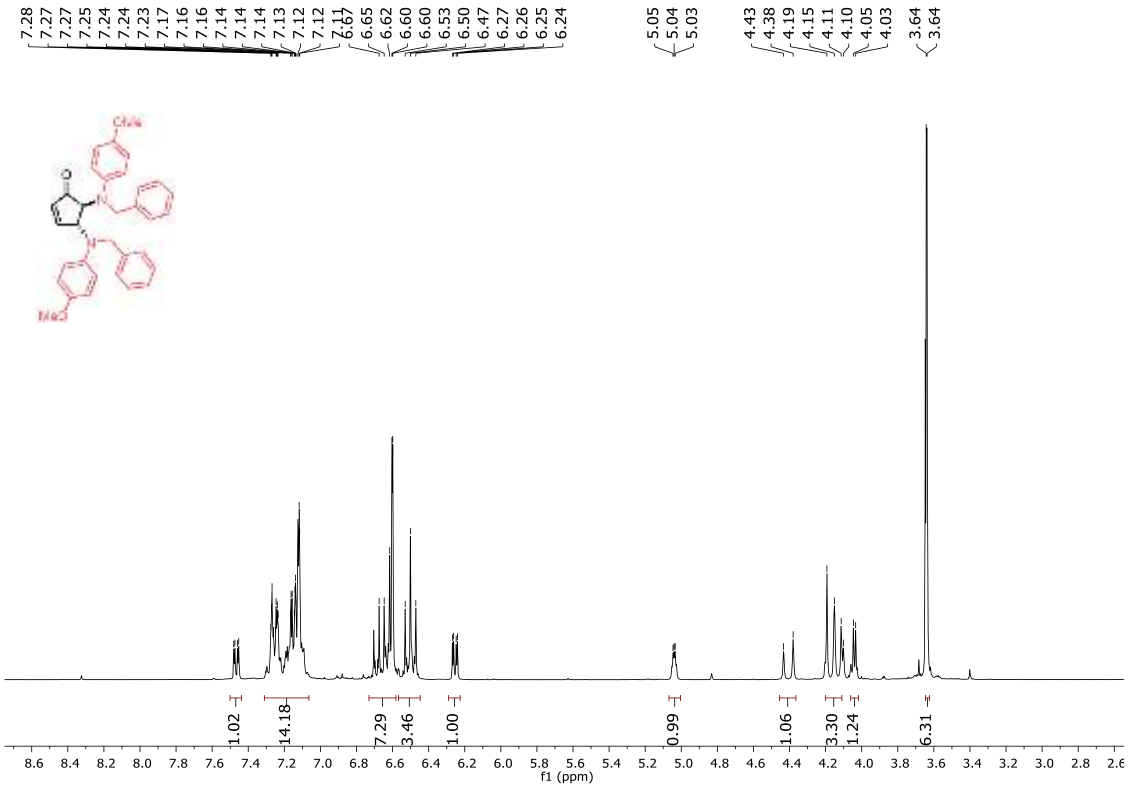

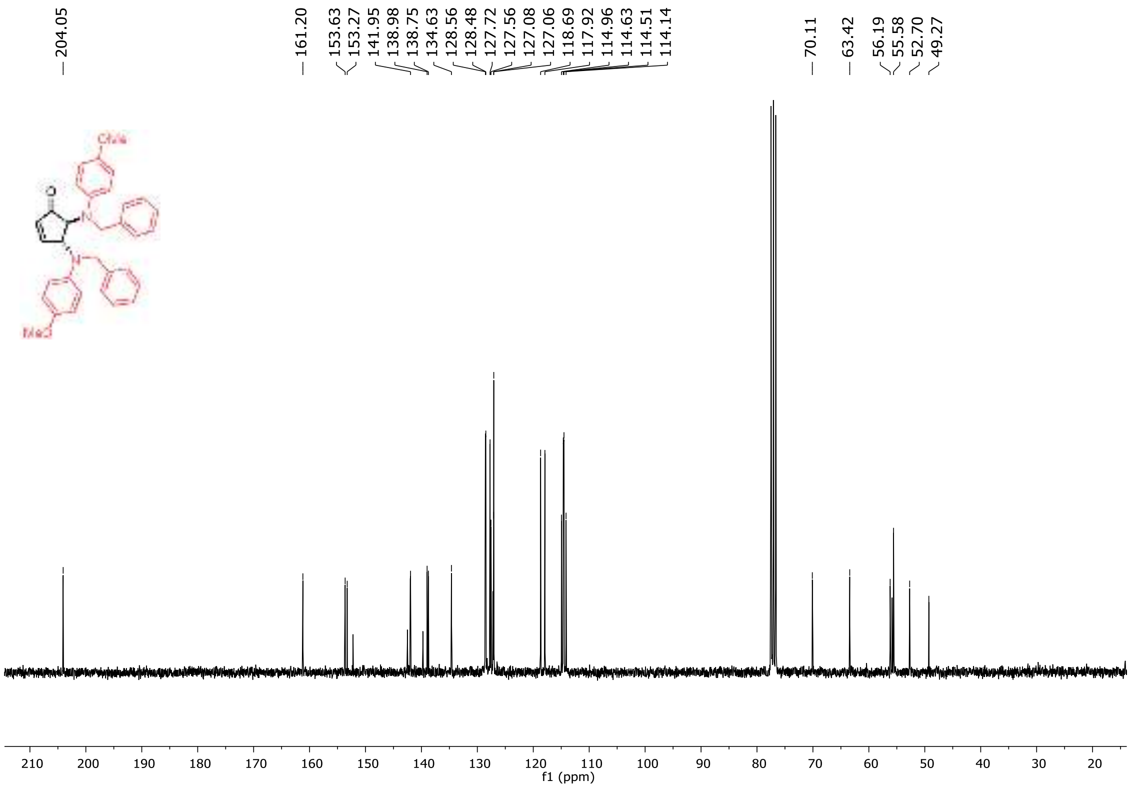

^{1}H NMR (300 MHz, $CDCl_3$) profile for a reaction to prepare **1n**.

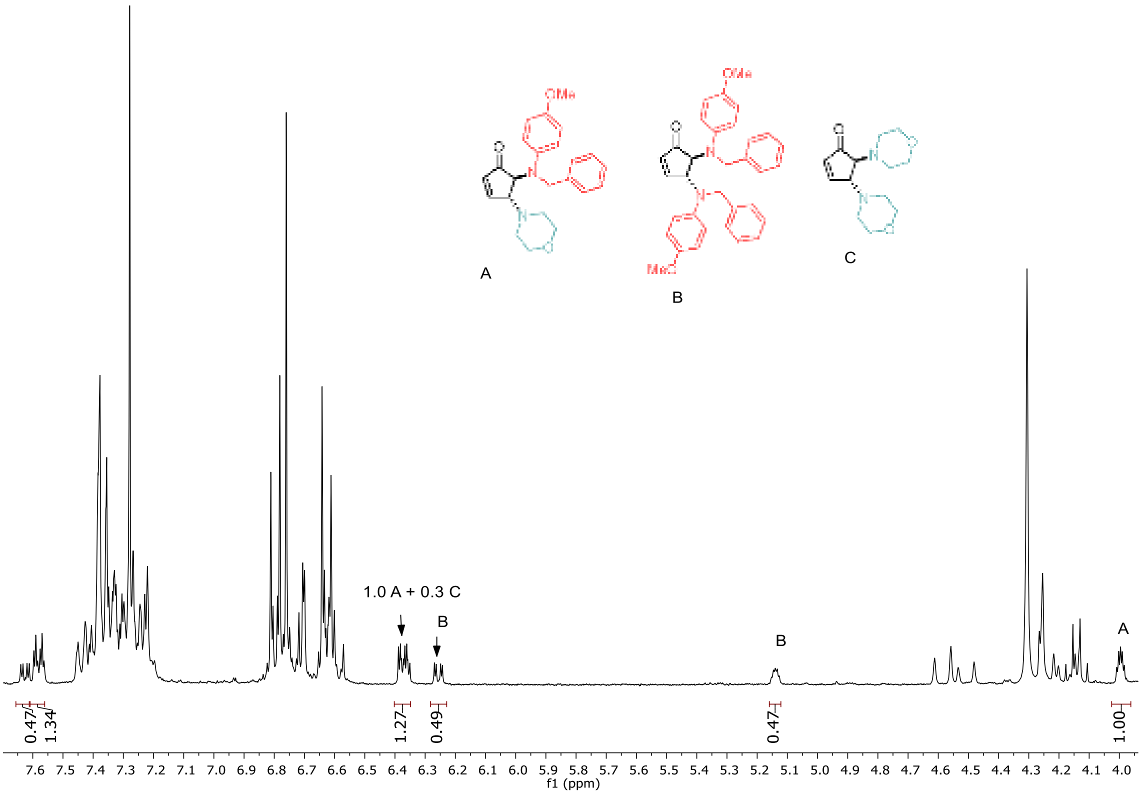

^1H NMR (300 MHz, $CDCl_3$) and ^{13}C NMR (75 MHz, $CDCl_3$) of compound **2a**

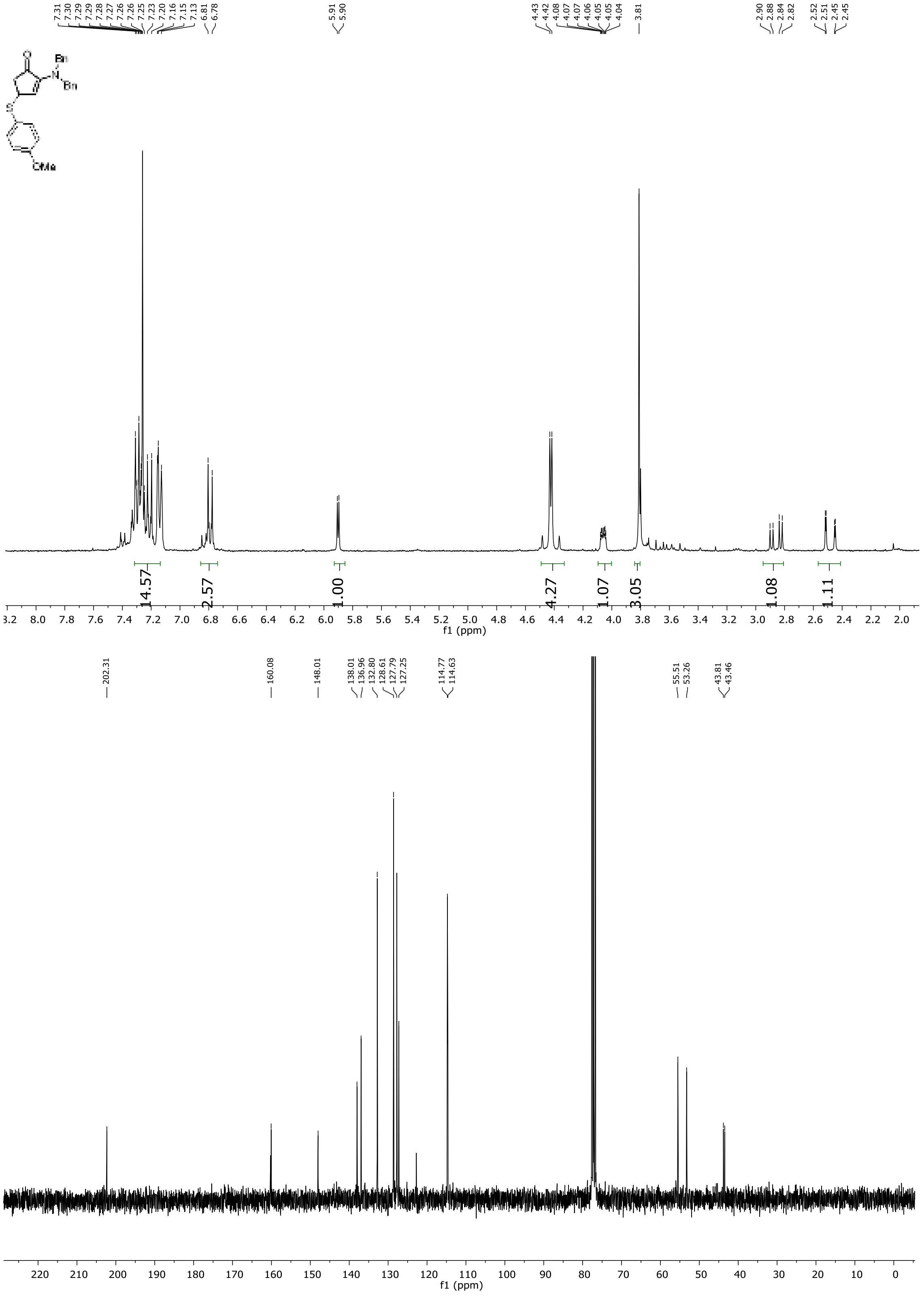

^{1}H NMR (300 MHz, $CDCl_3$) and ^{13}C NMR (75 MHz, $CDCl_3$) of compound **2b**

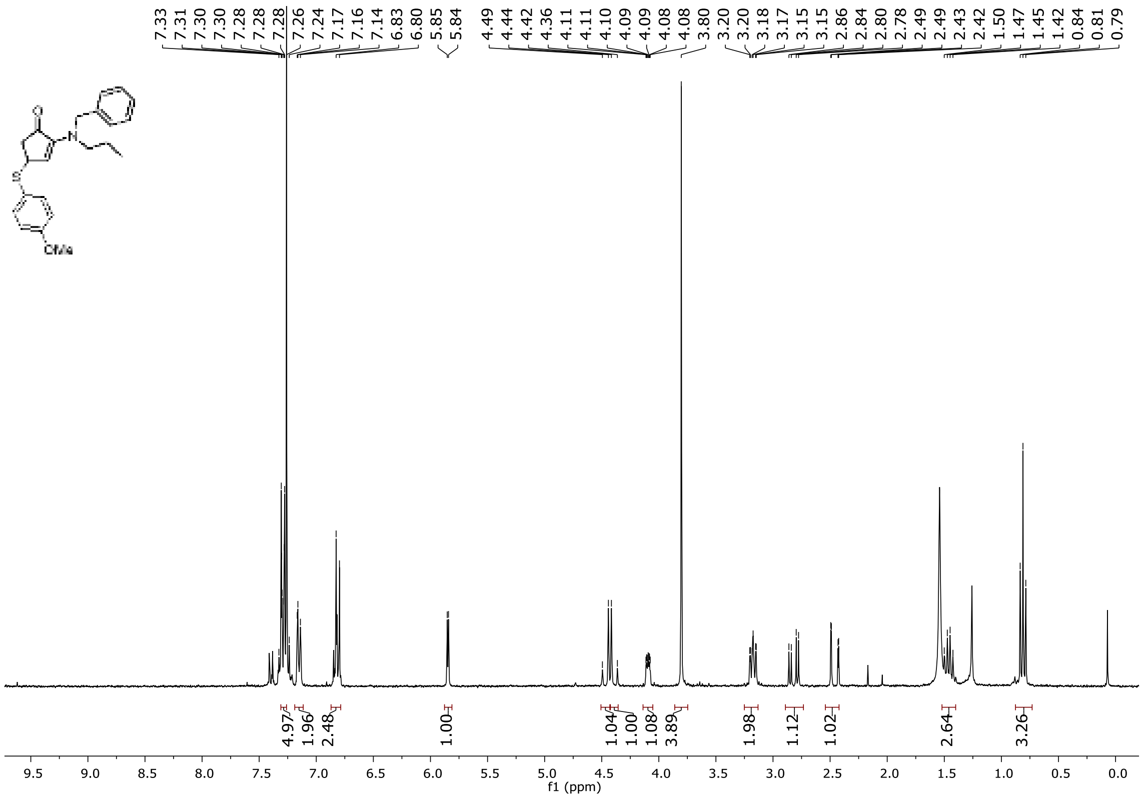

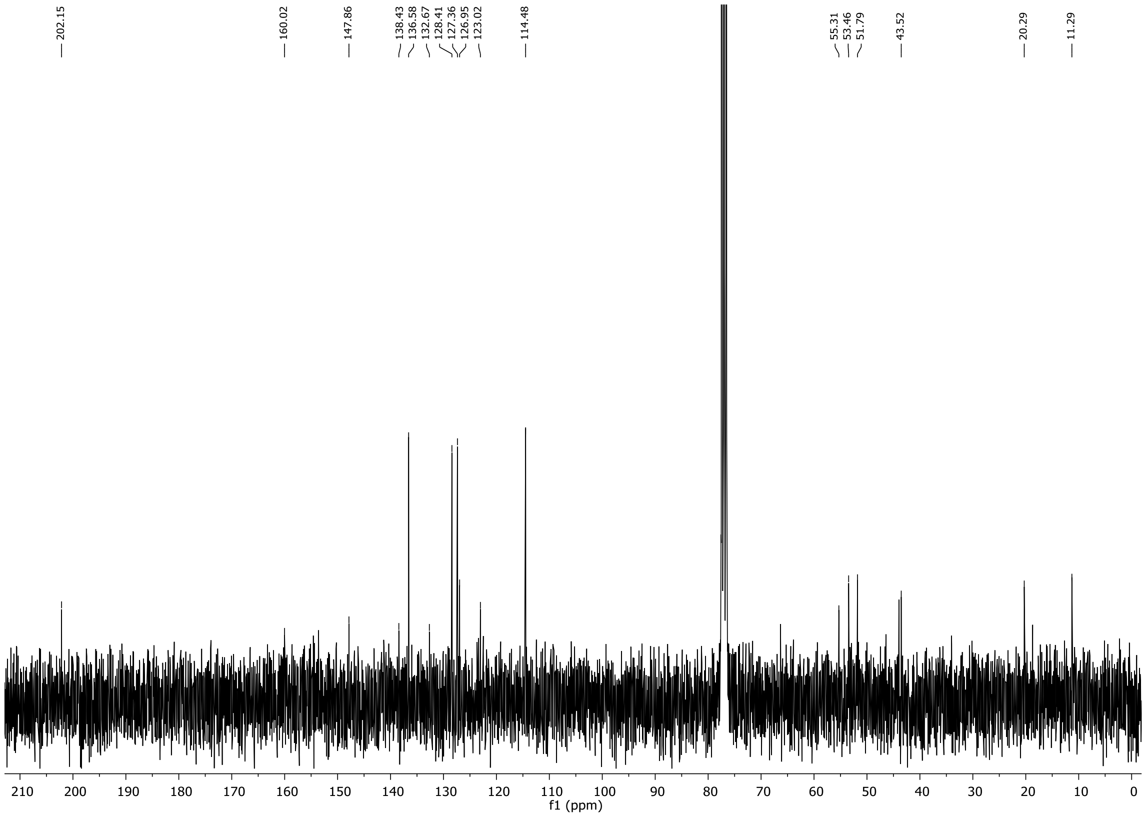

^{1}H NMR (300 MHz, $CDCl_3$) and ^{13}C NMR (75 MHz, $CDCl_3$) of compound **2c**

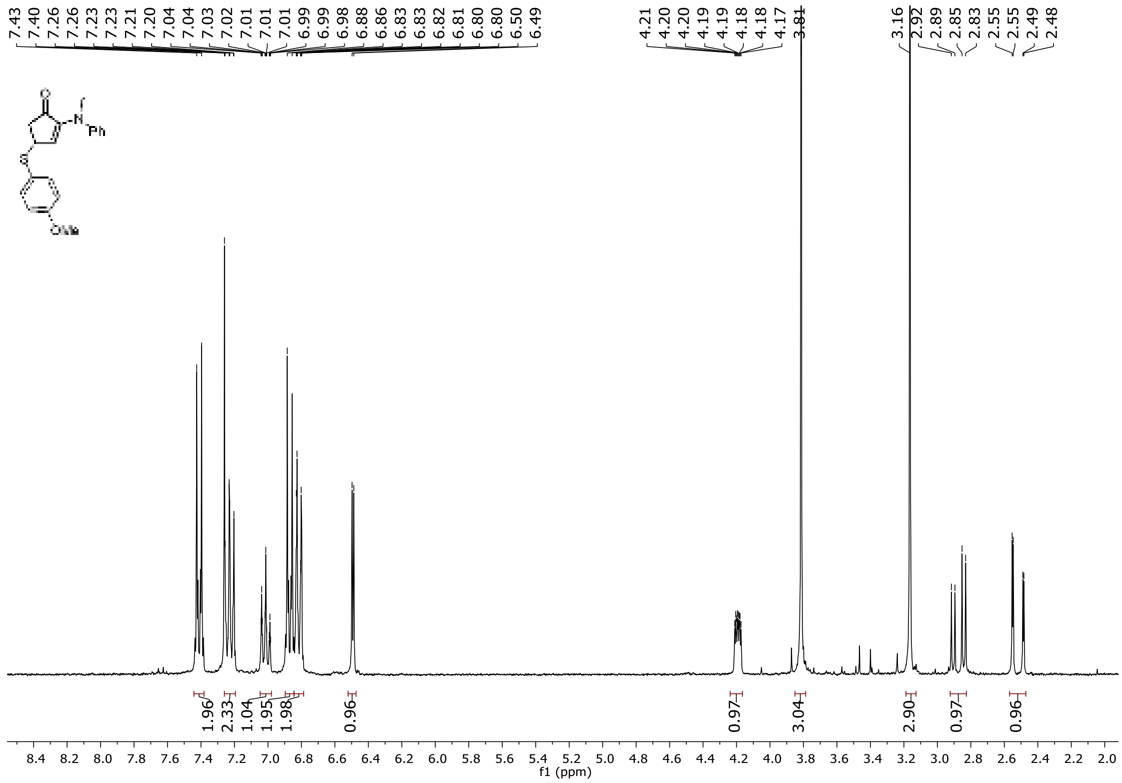

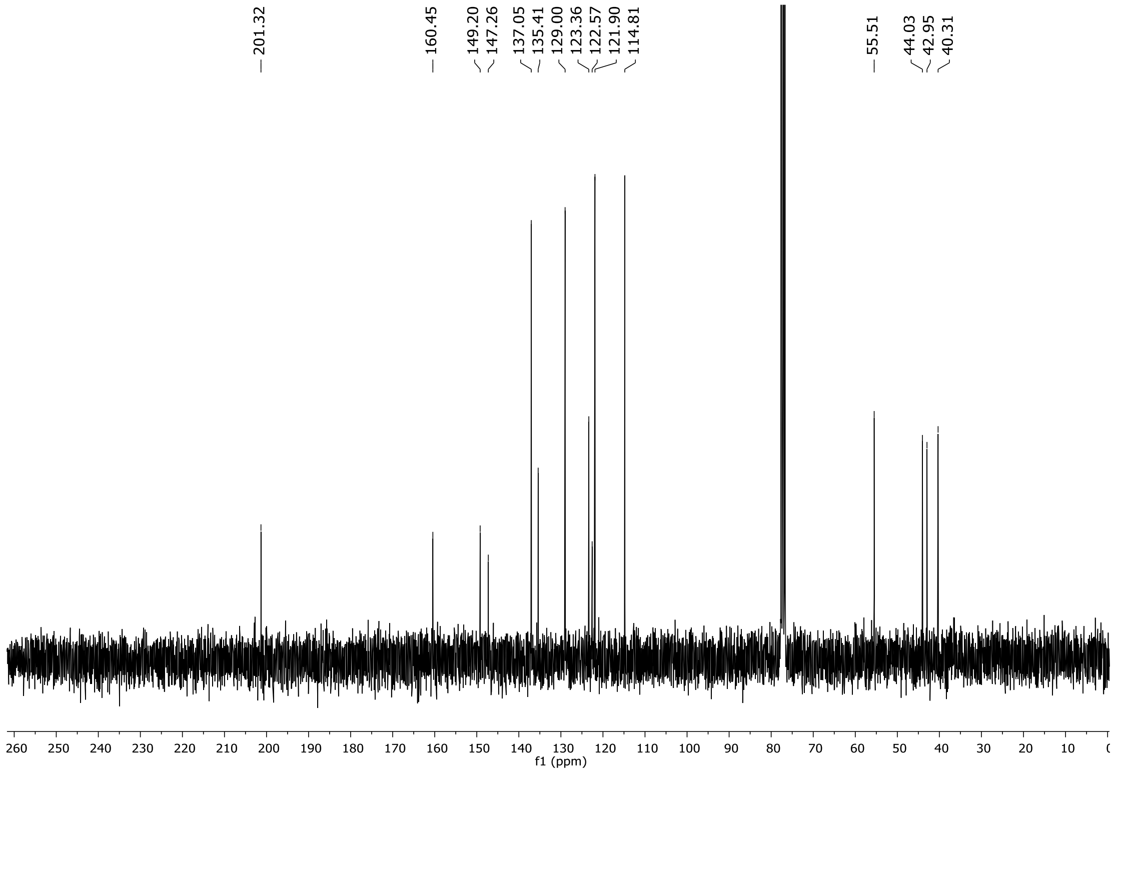

^{1}H NMR (300 MHz, $CDCl_3$) and ^{13}C NMR (75 MHz, $CDCl_3$) of compound **2d**

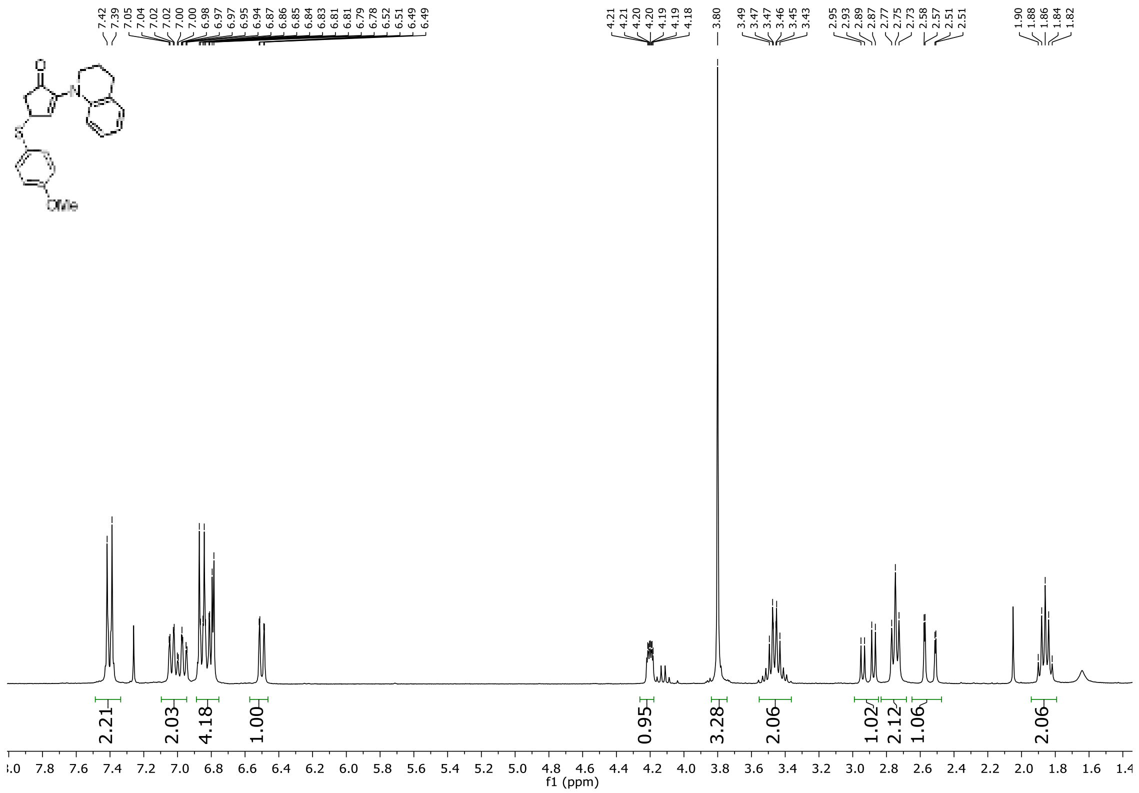

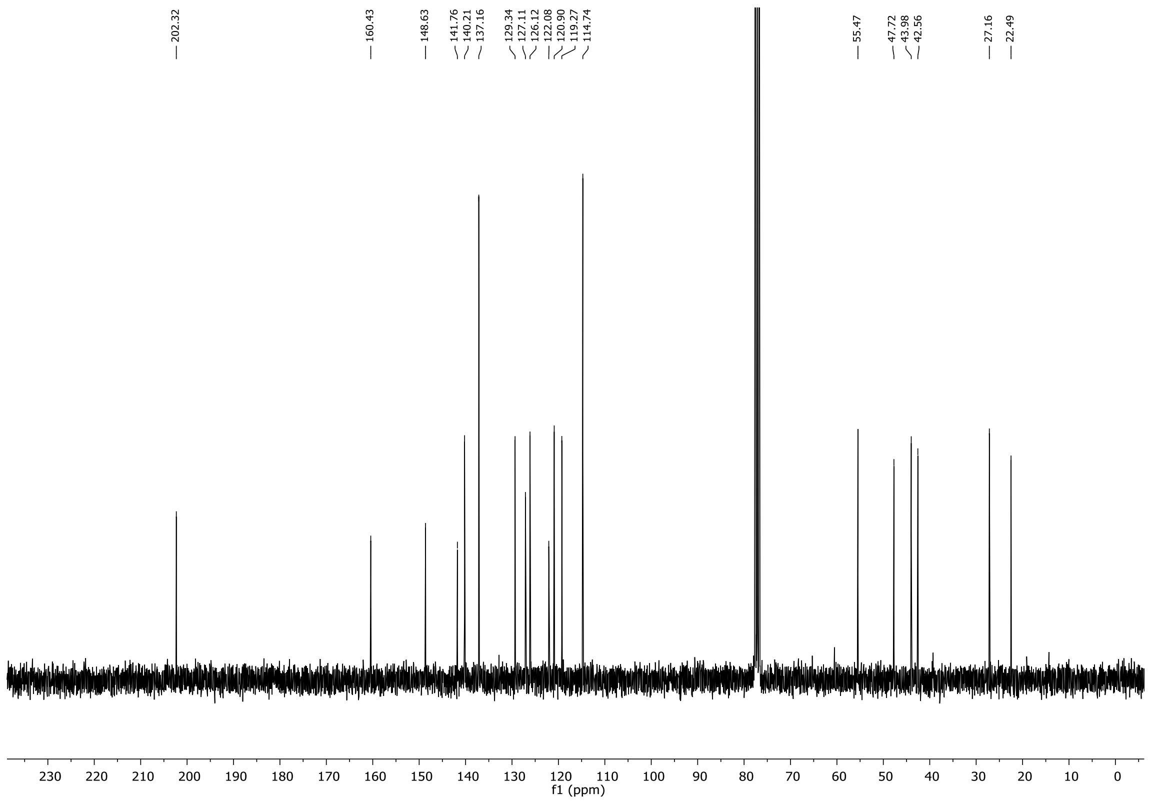

^{1}H NMR (300 MHz, $CDCl_3$) and ^{13}C NMR (75 MHz, $CDCl_3$) of compound **2e**

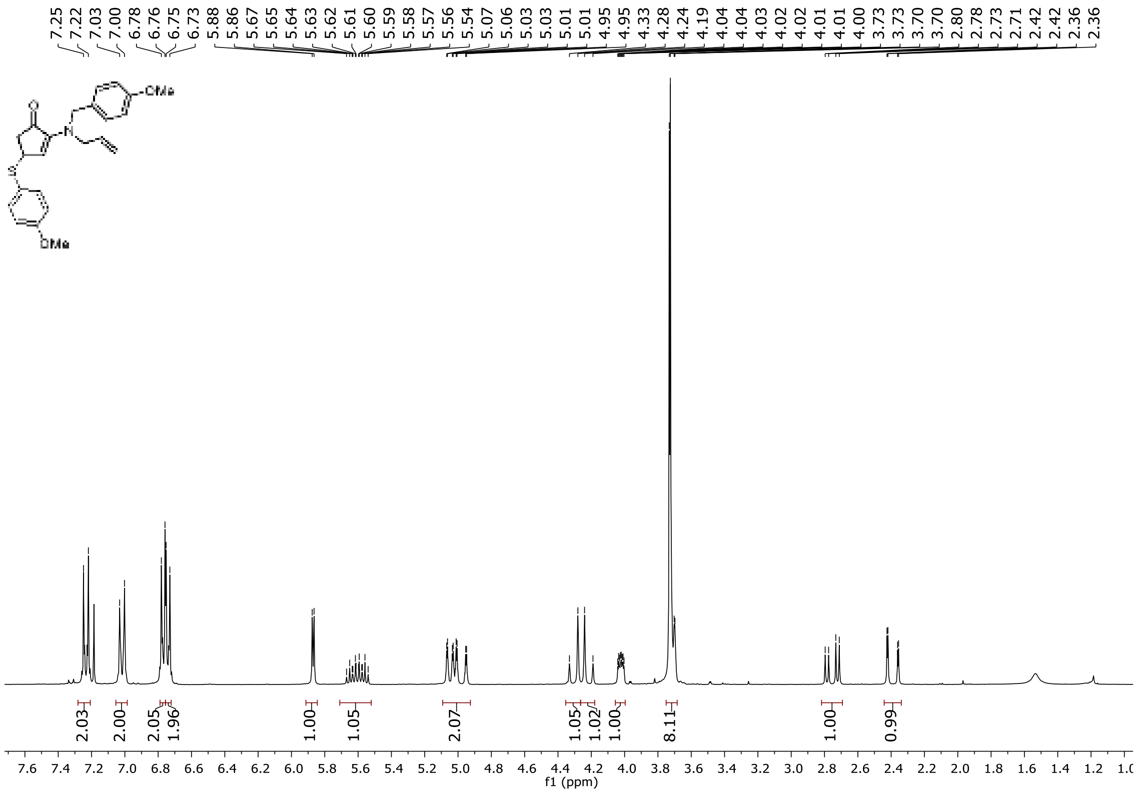

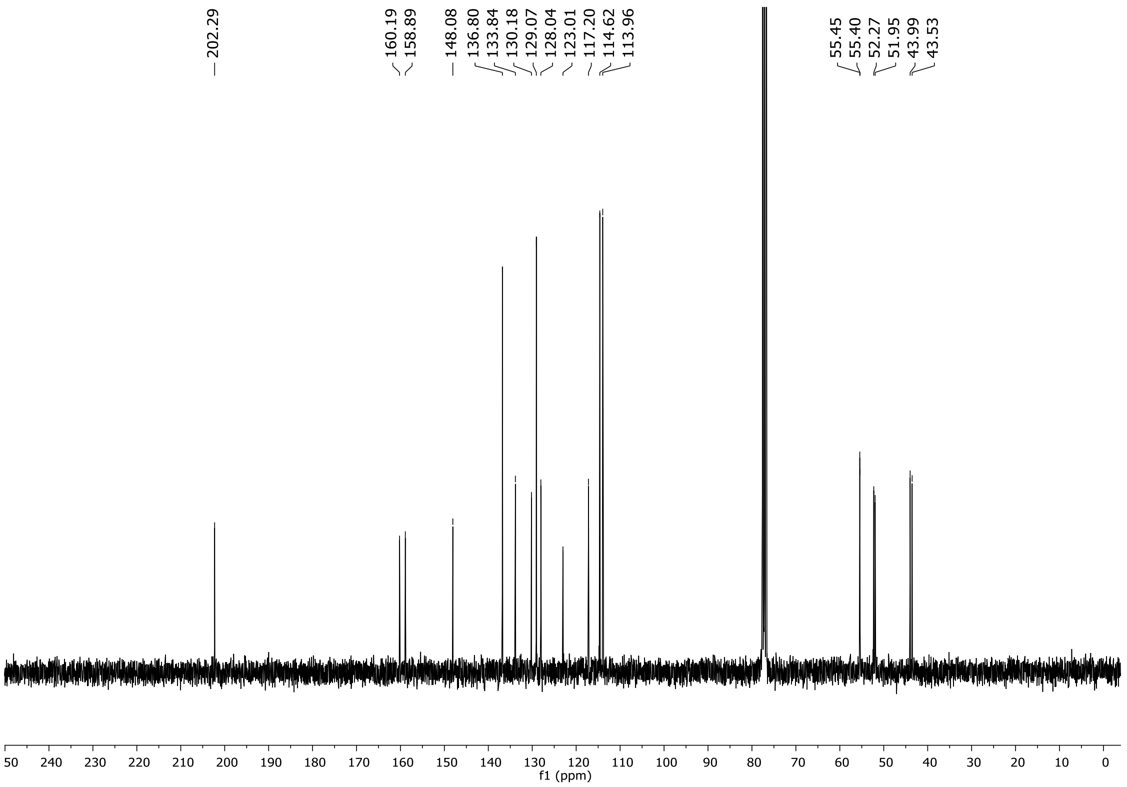

^{1}H NMR (300 MHz, $CDCl_3$) and ^{13}C NMR (75 MHz, $CDCl_3$) of compound **2f**

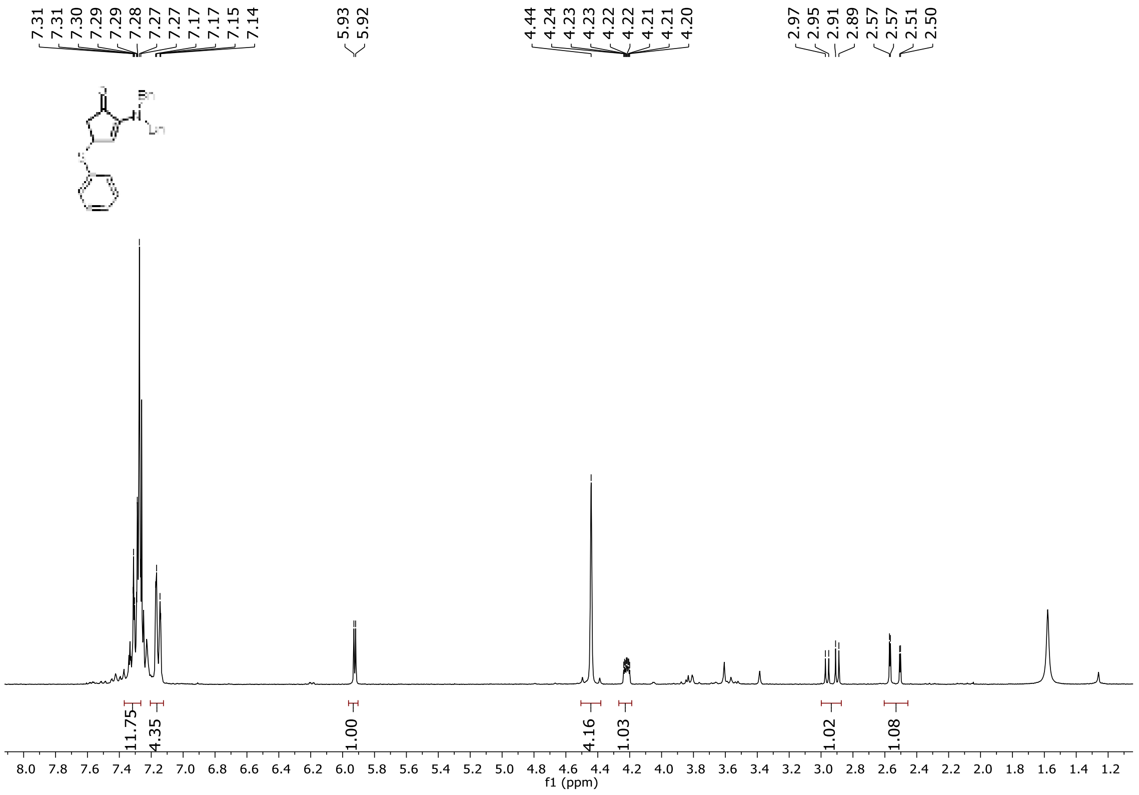

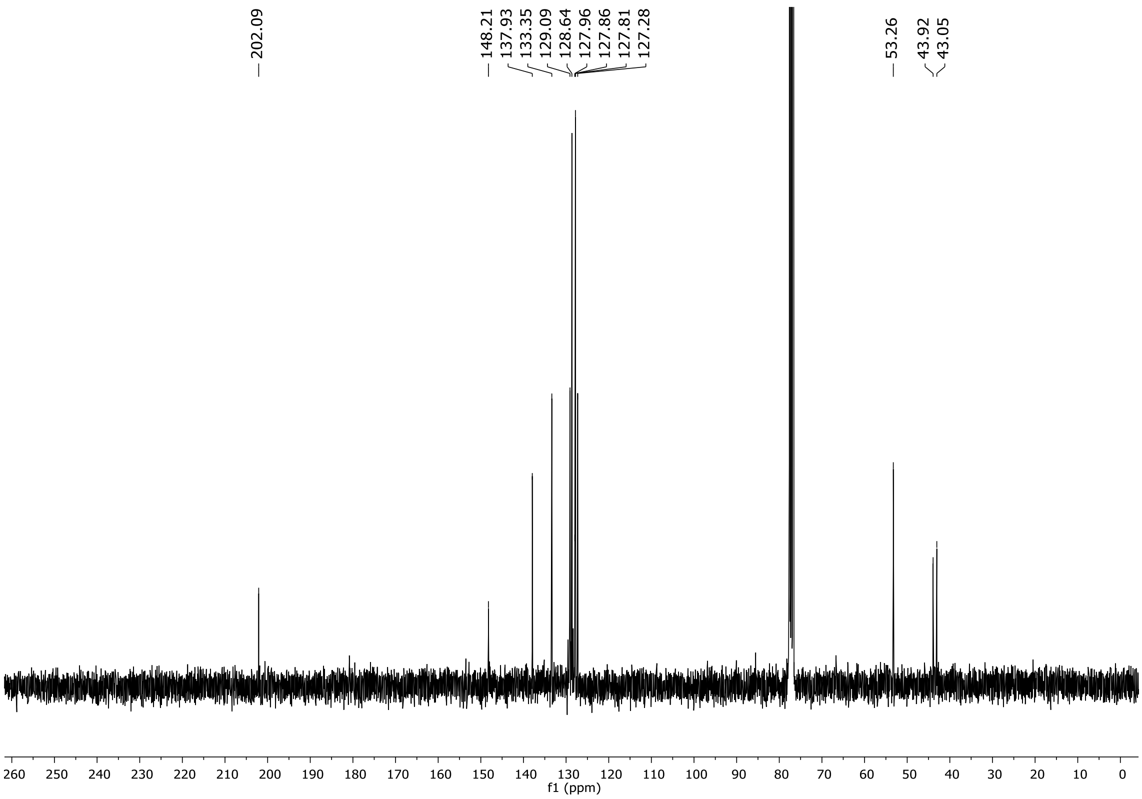

^1H NMR (300 MHz, $CDCl_3$) and ^{13}C NMR (75 MHz, $CDCl_3$) of compound **2g**

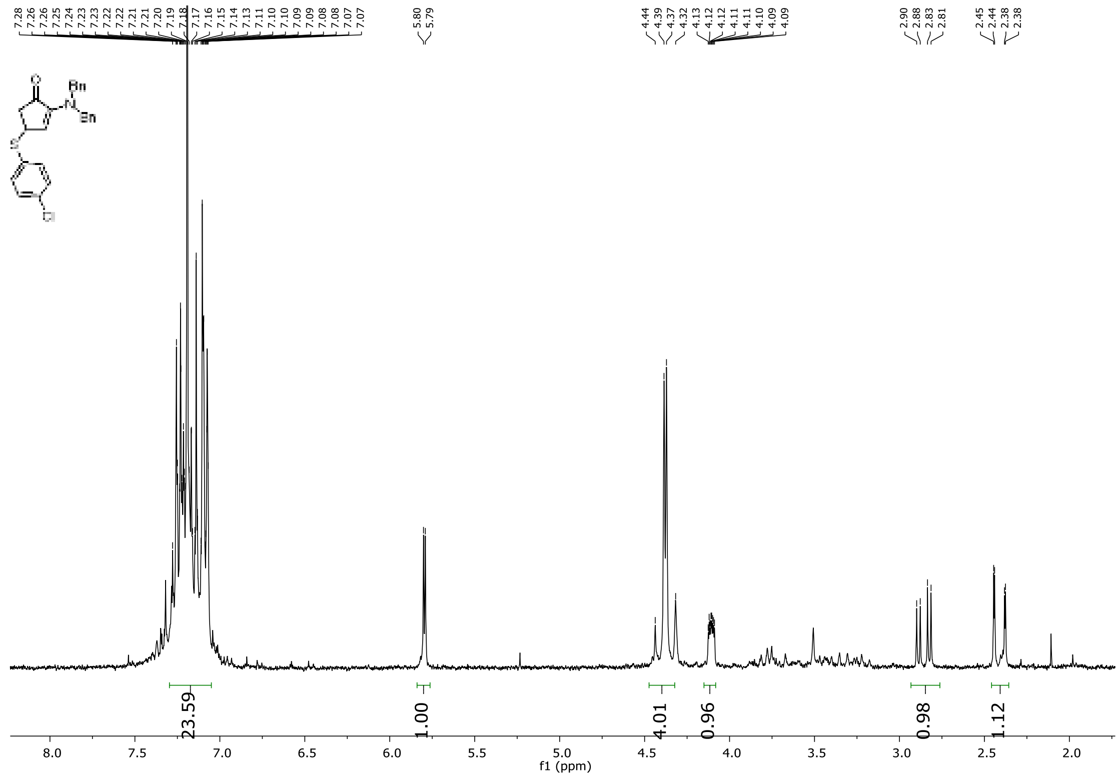

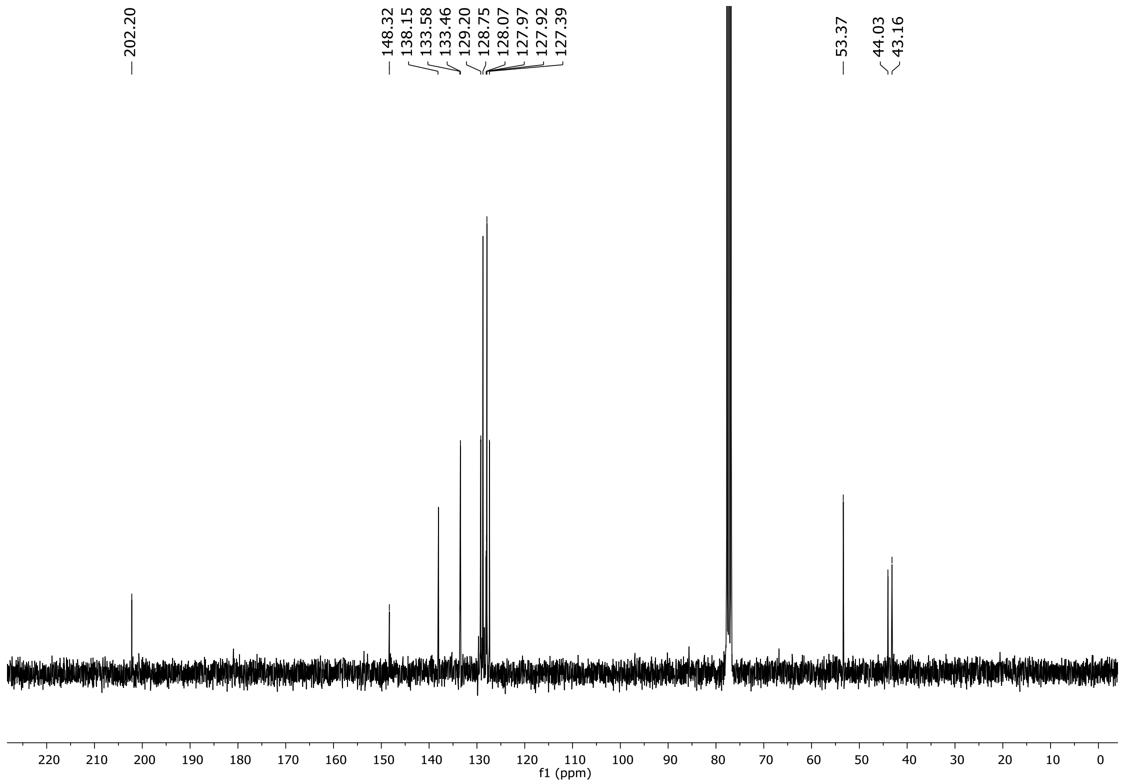

^{1}H NMR (300 MHz, $CDCl_3$) and ^{13}C NMR (75 MHz, $CDCl_3$) of compound **2h**

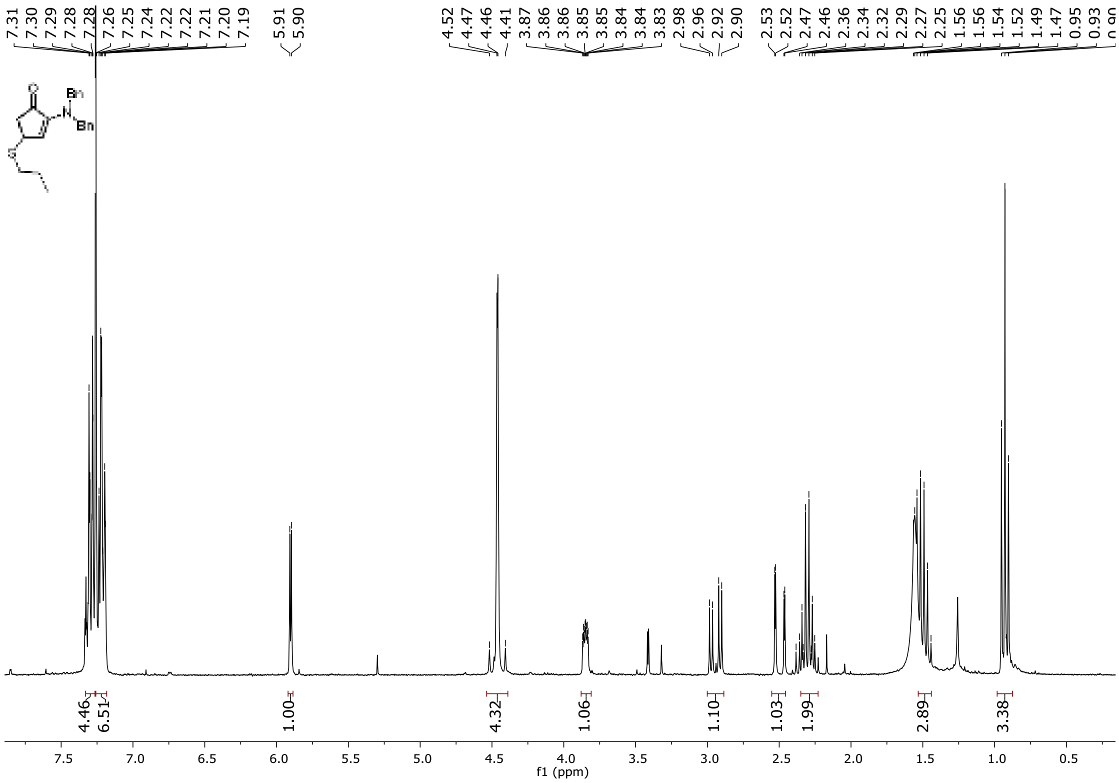

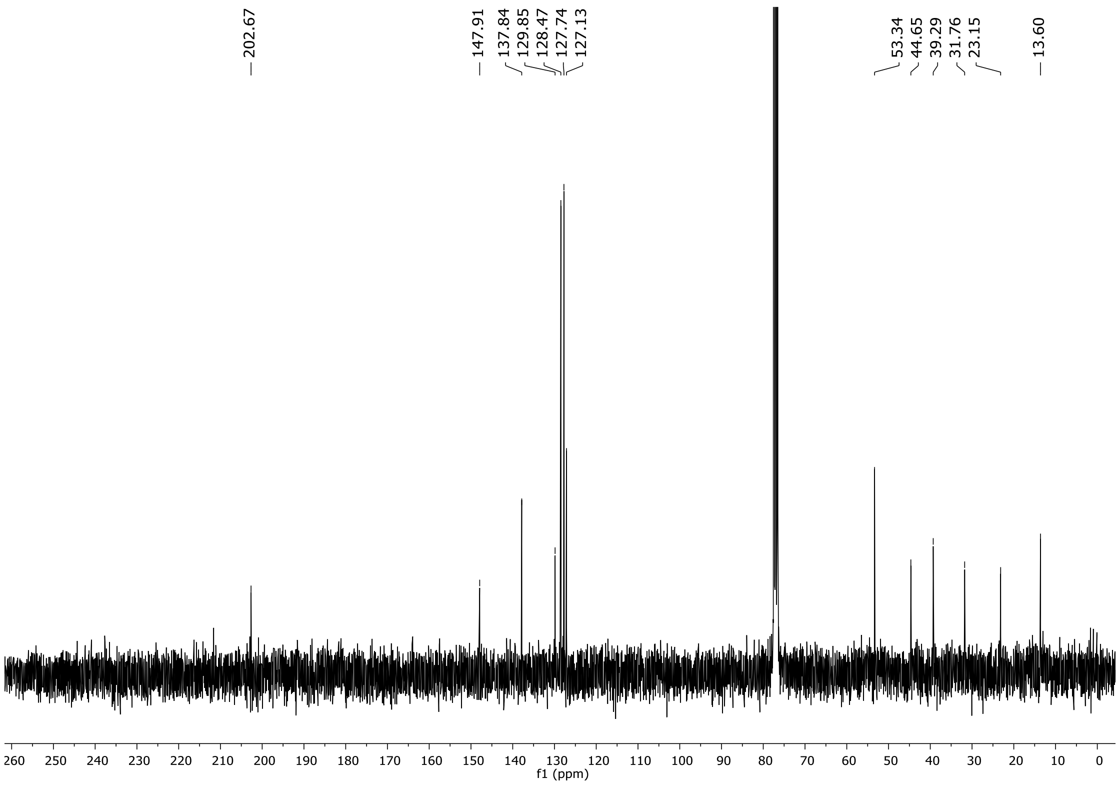

^{1}H NMR (300 MHz, $CDCl_3$) and ^{13}C NMR (75 MHz, $CDCl_3$) of compound **2i**

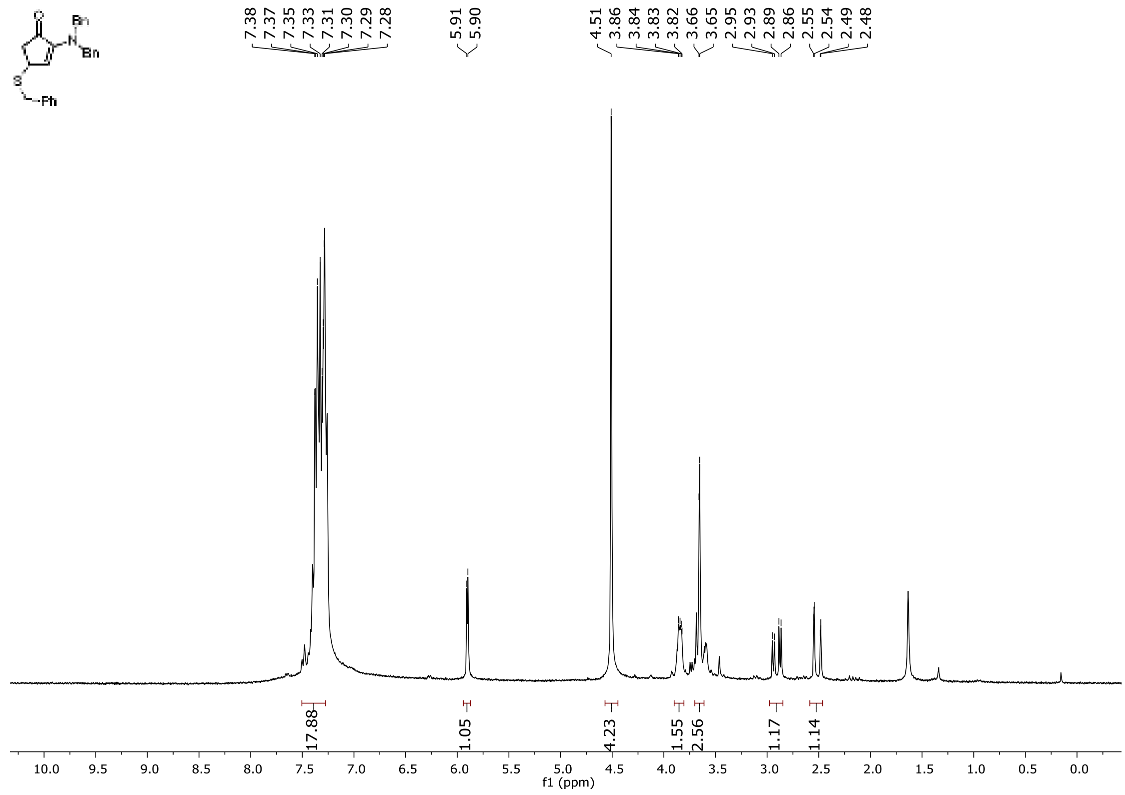

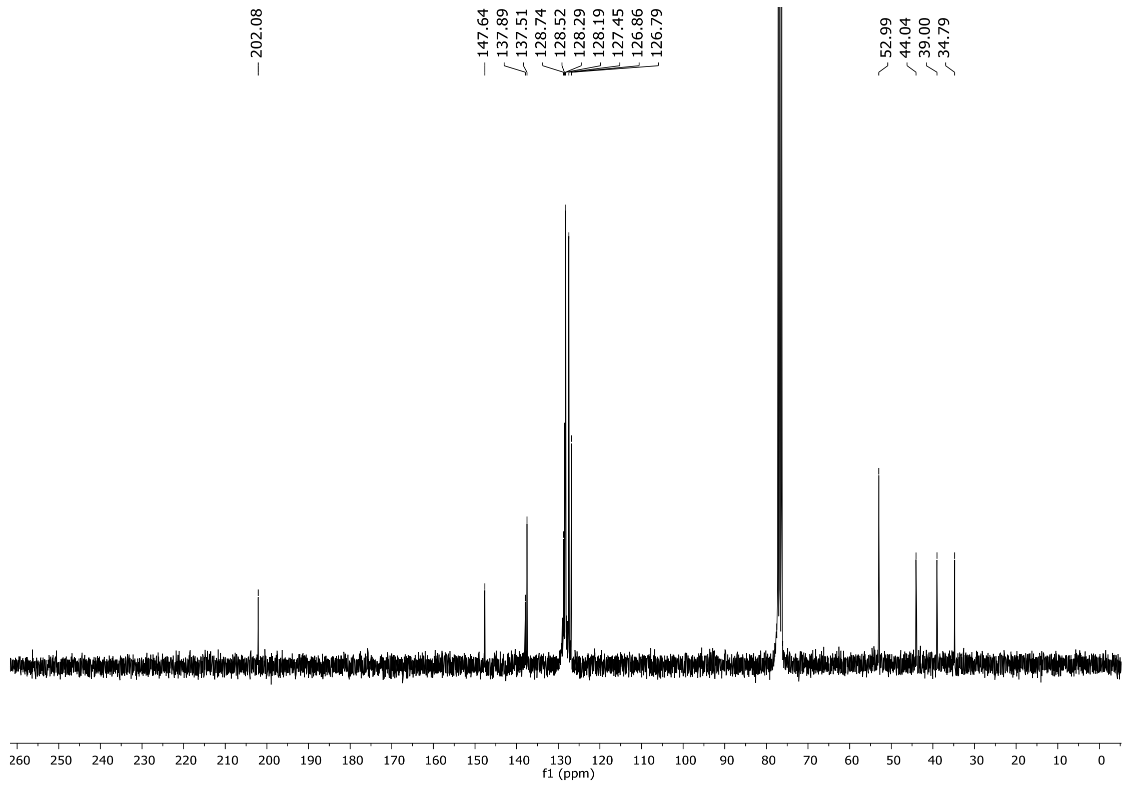

^{1}H NMR (300 MHz, $CDCl_3$) and ^{13}C NMR (75 MHz, $CDCl_3$) of compound **S1**

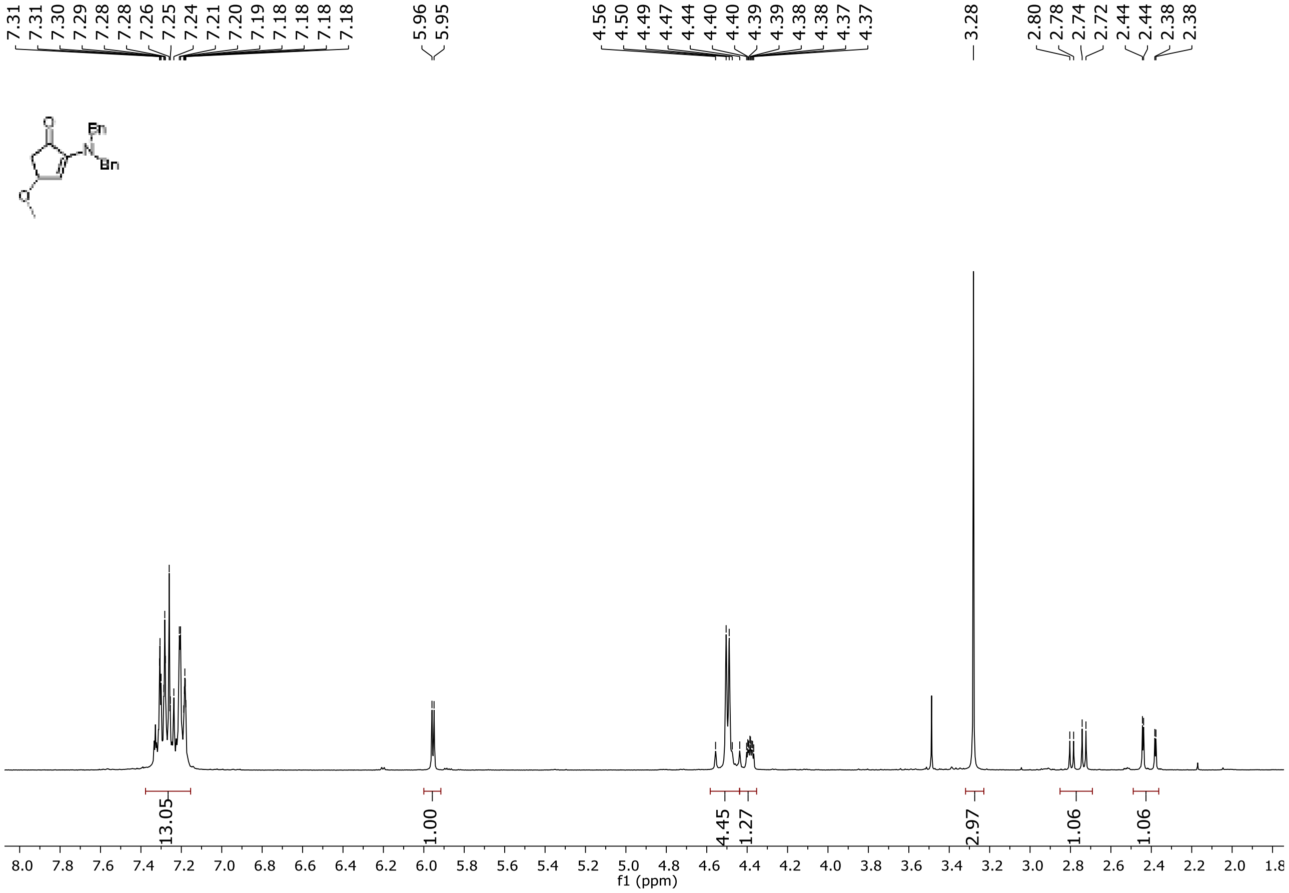

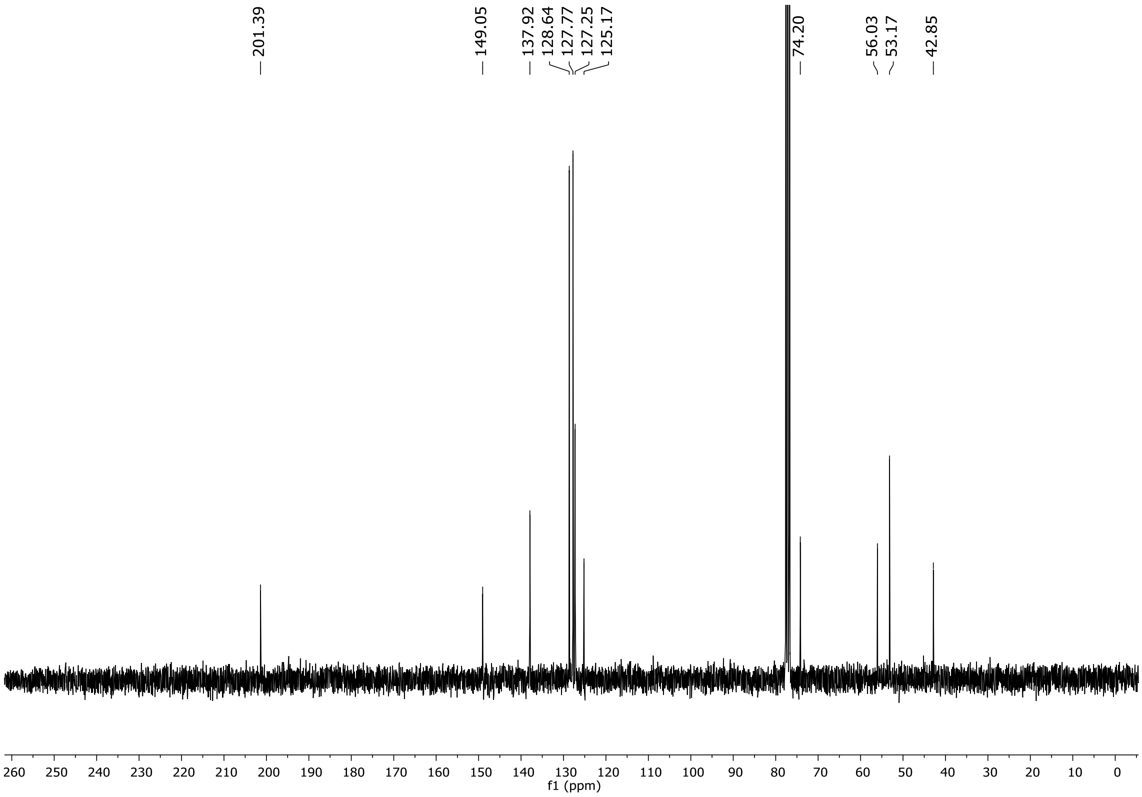

HSQC and HMBC of compound **S1**

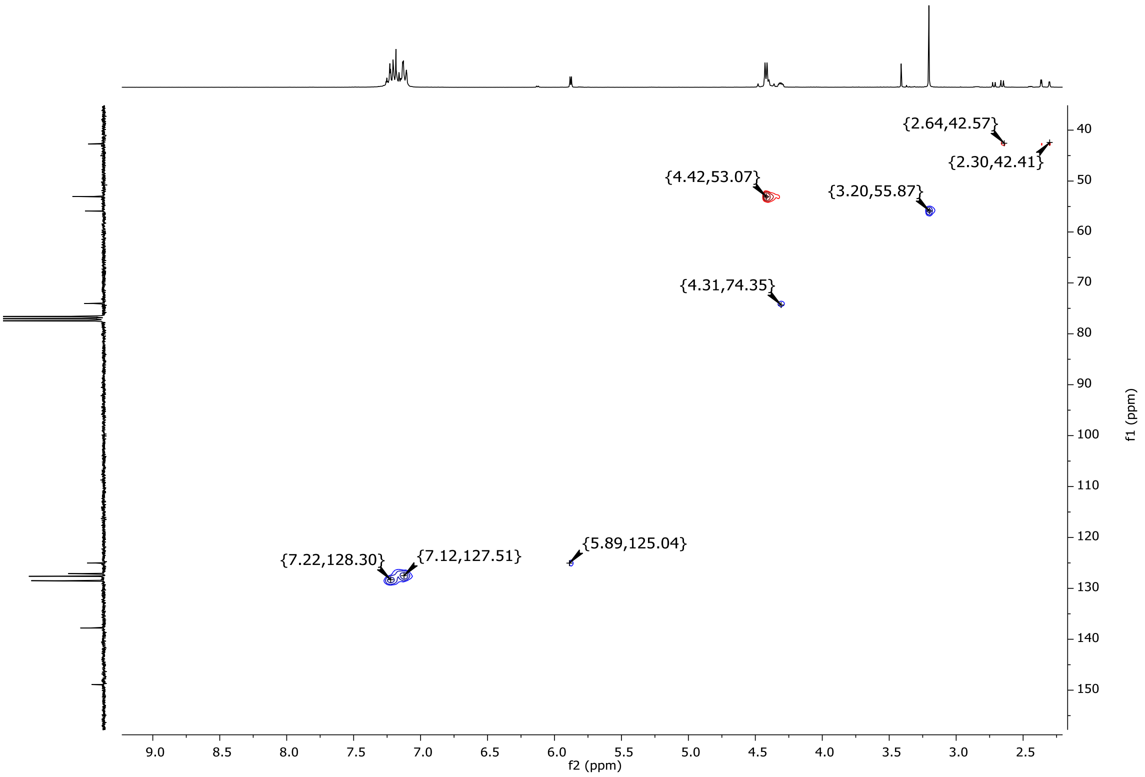

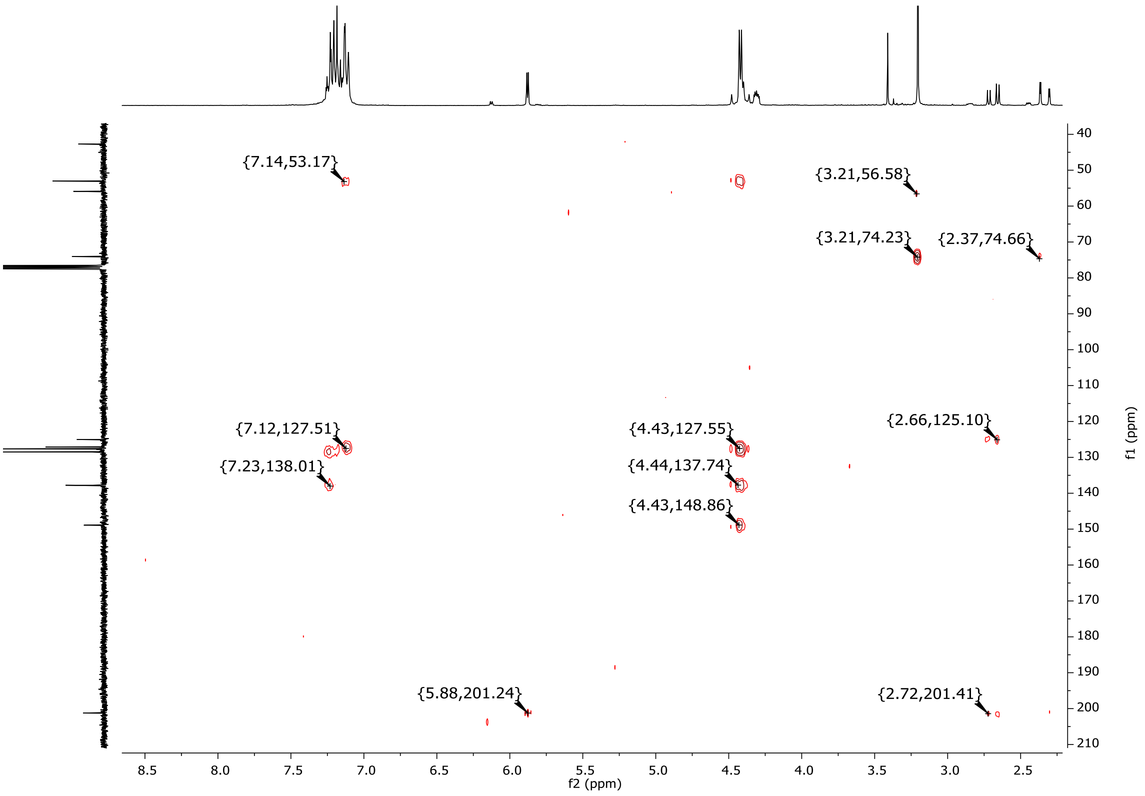

^{1}H NMR (300 MHz, $CDCl_3$) and ^{13}C NMR (75 MHz, $CDCl_3$) of compound **S2**

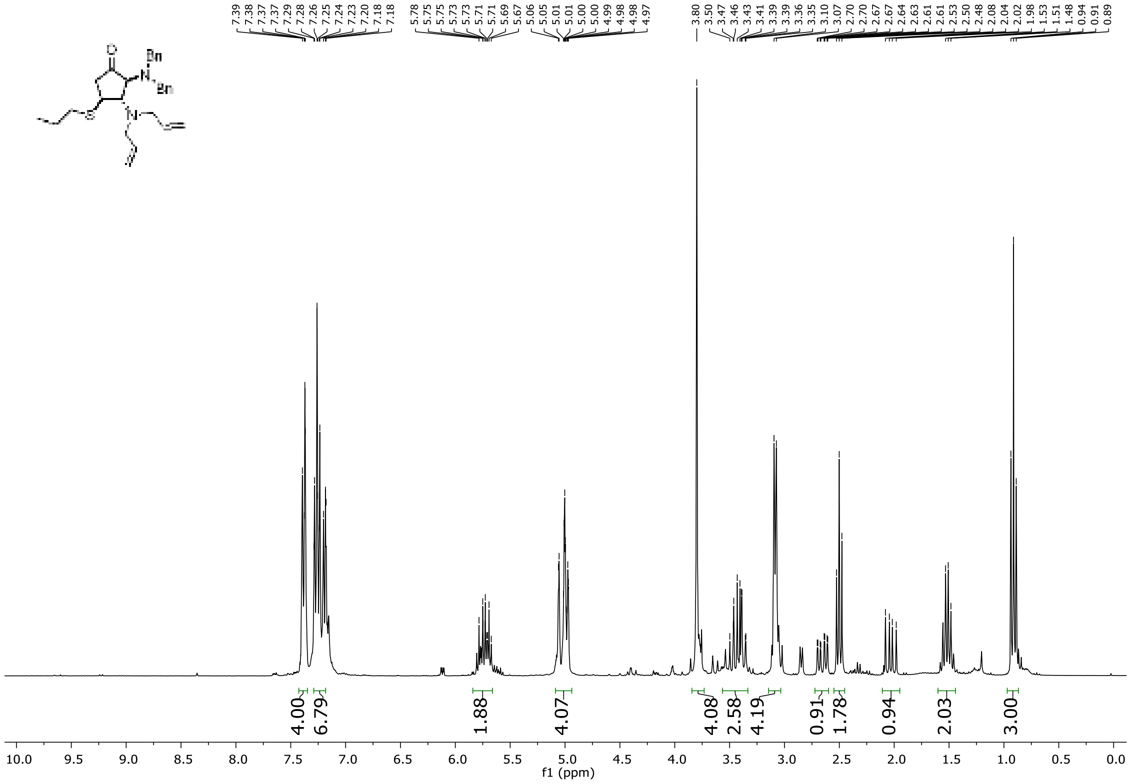

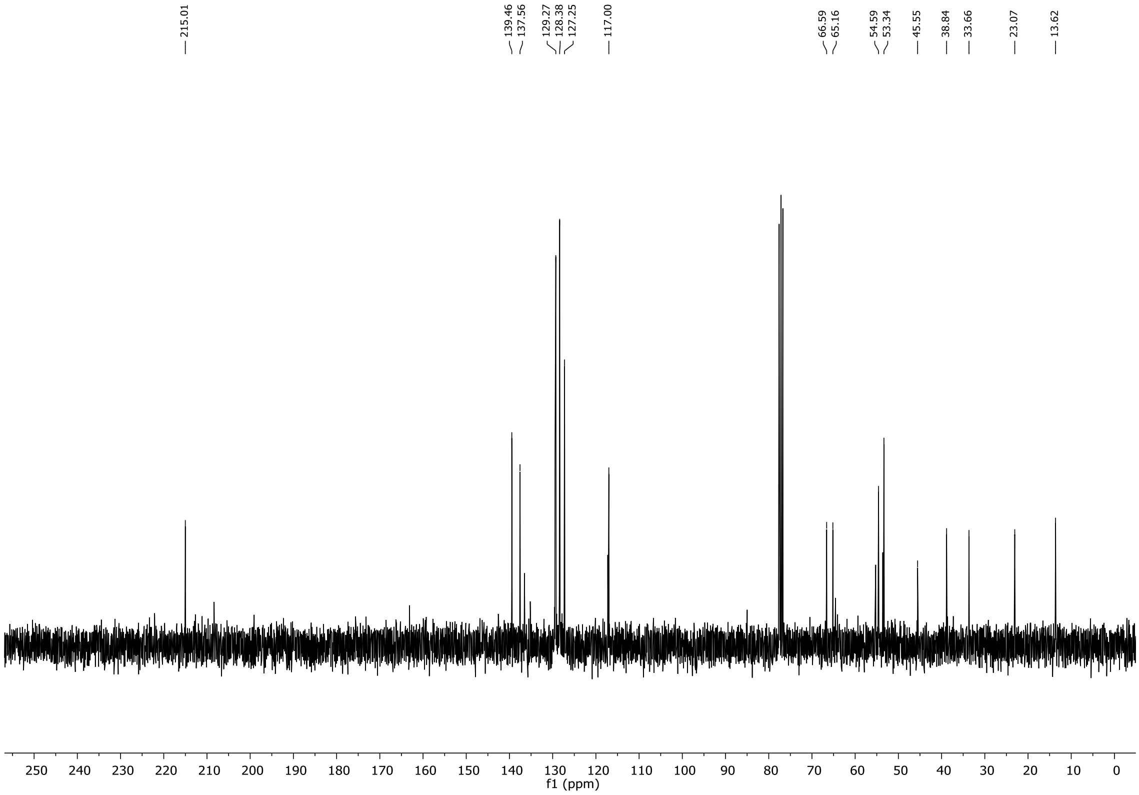

^{1}H NMR (300 MHz, $CDCl_3$) and ^{13}C NMR (75 MHz, $CDCl_3$) of compound **S5**

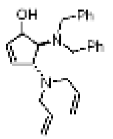

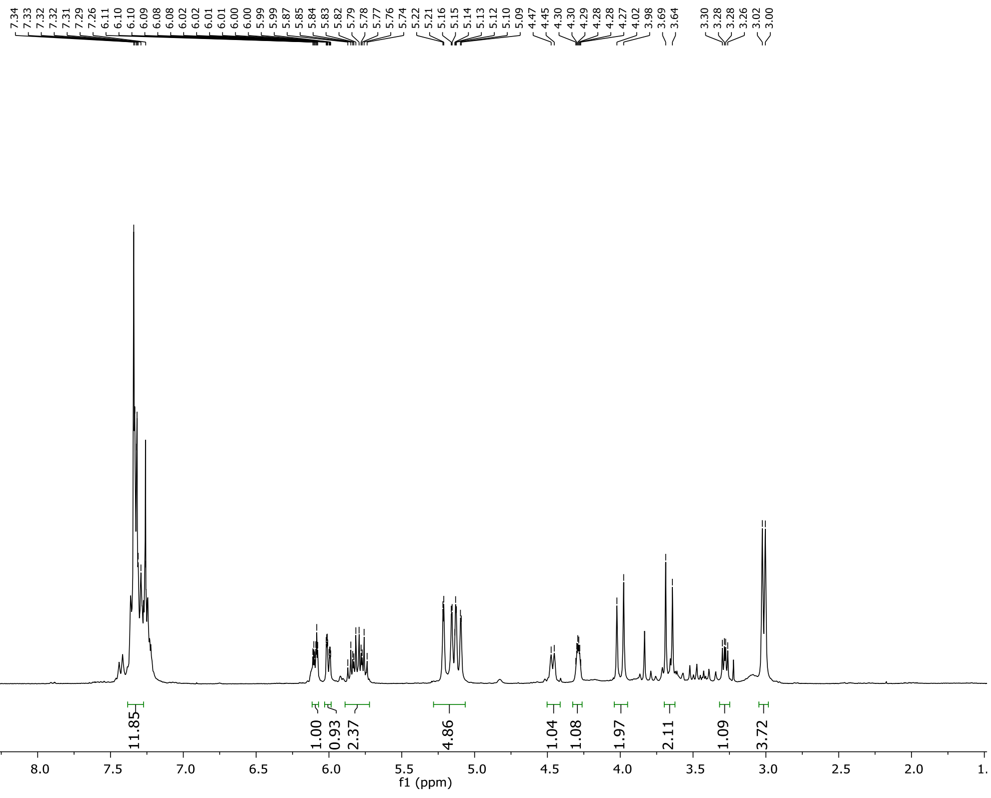

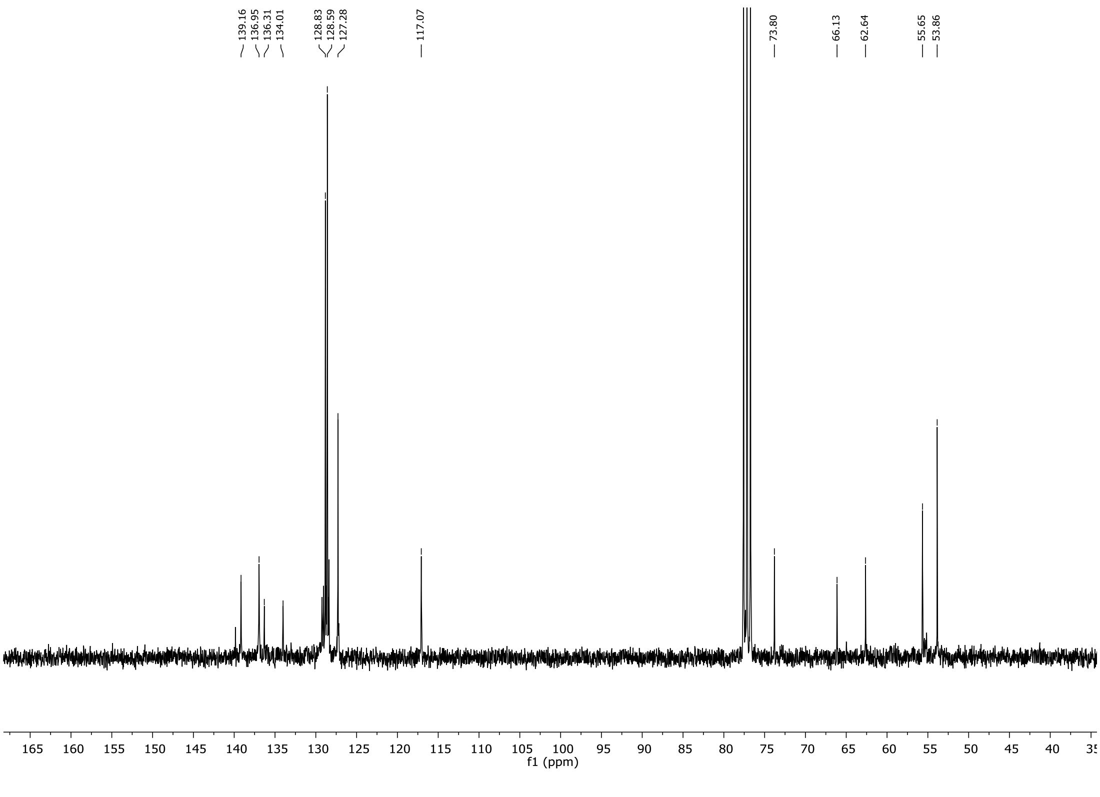

HSQC and HMBC of compound **S5**

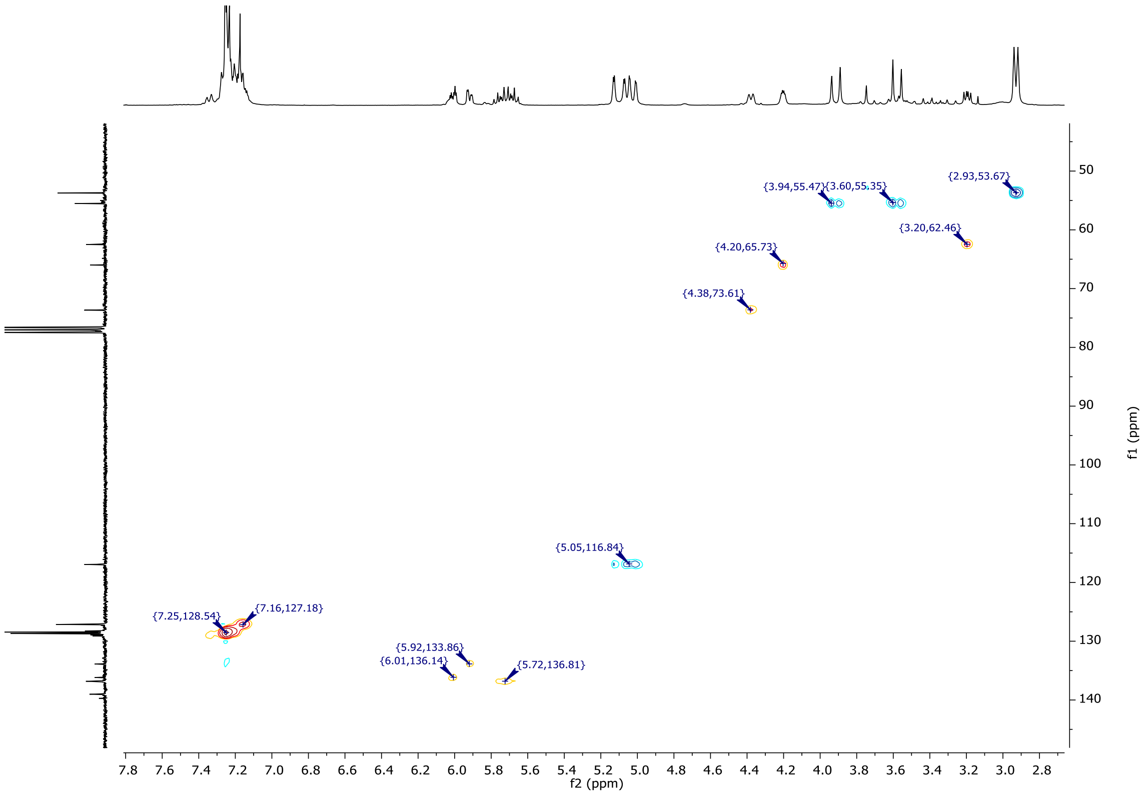

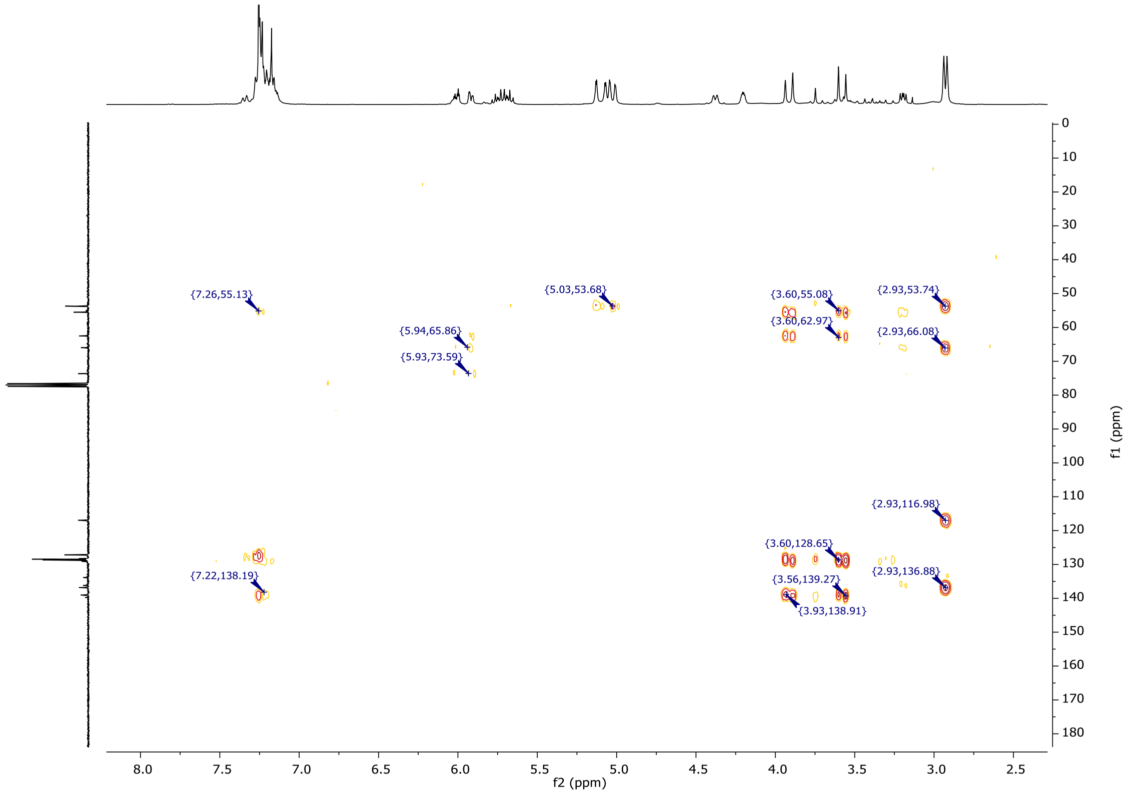

^{1}H NMR (300 MHz, $CDCl_3$) and ^{13}C NMR (75 MHz, $CDCl_3$) of compound **4**

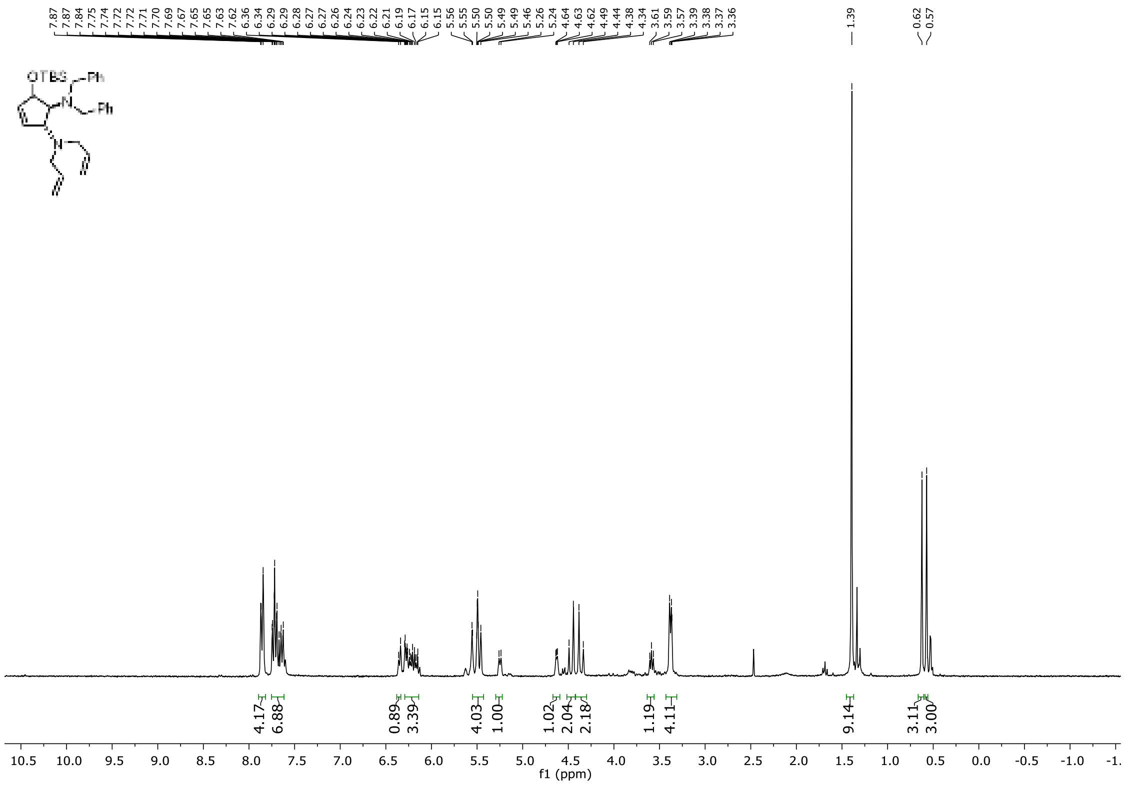

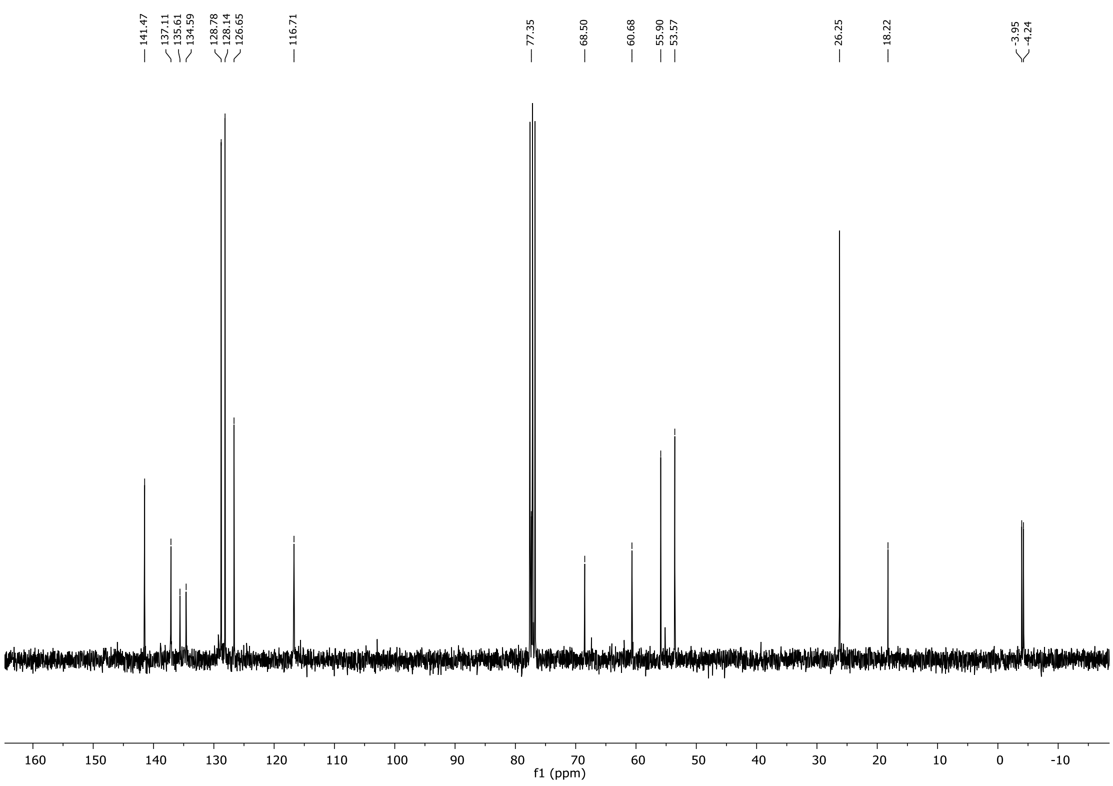

HSQC of compound **4**

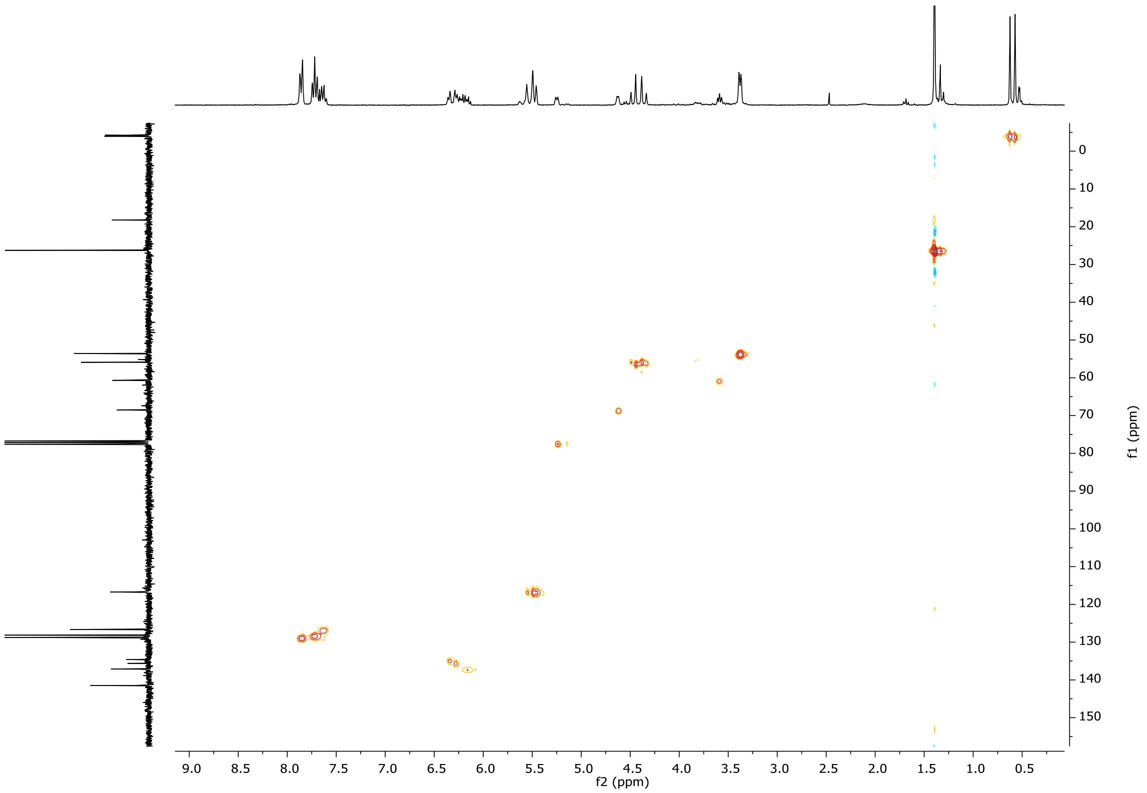

^{1}H NMR (300 MHz, $CDCl_3$) and ^{13}C NMR (75 MHz, $CDCl_3$) of compound **5**

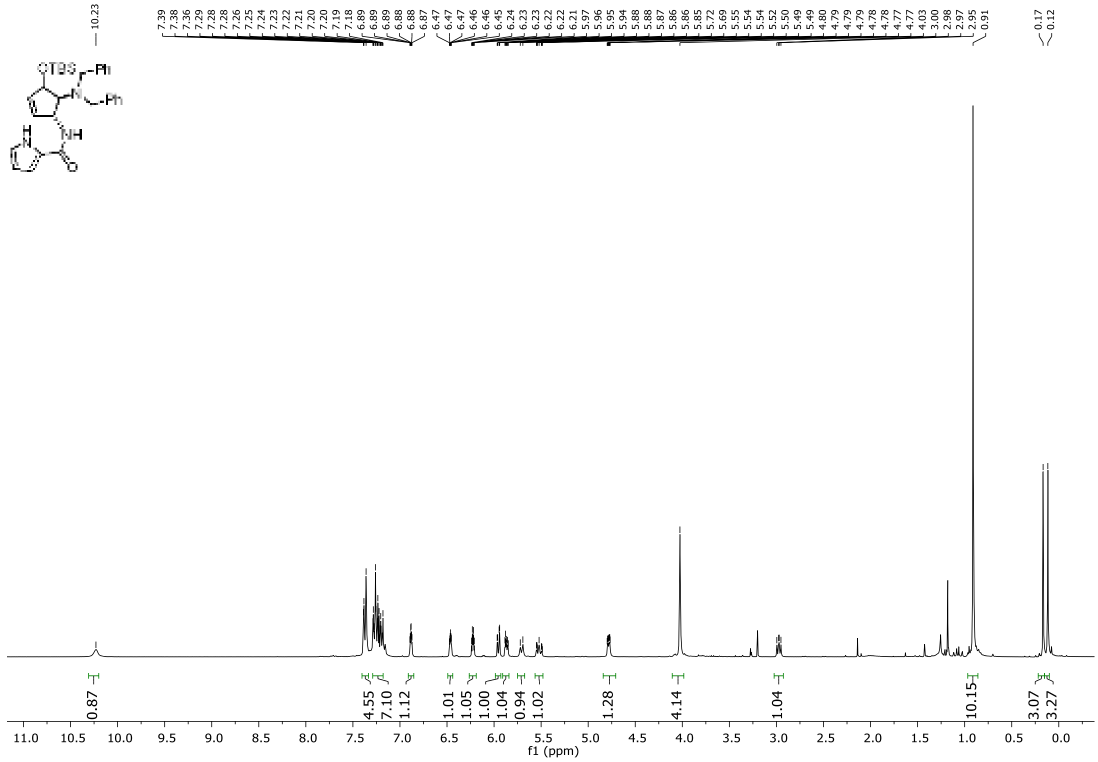

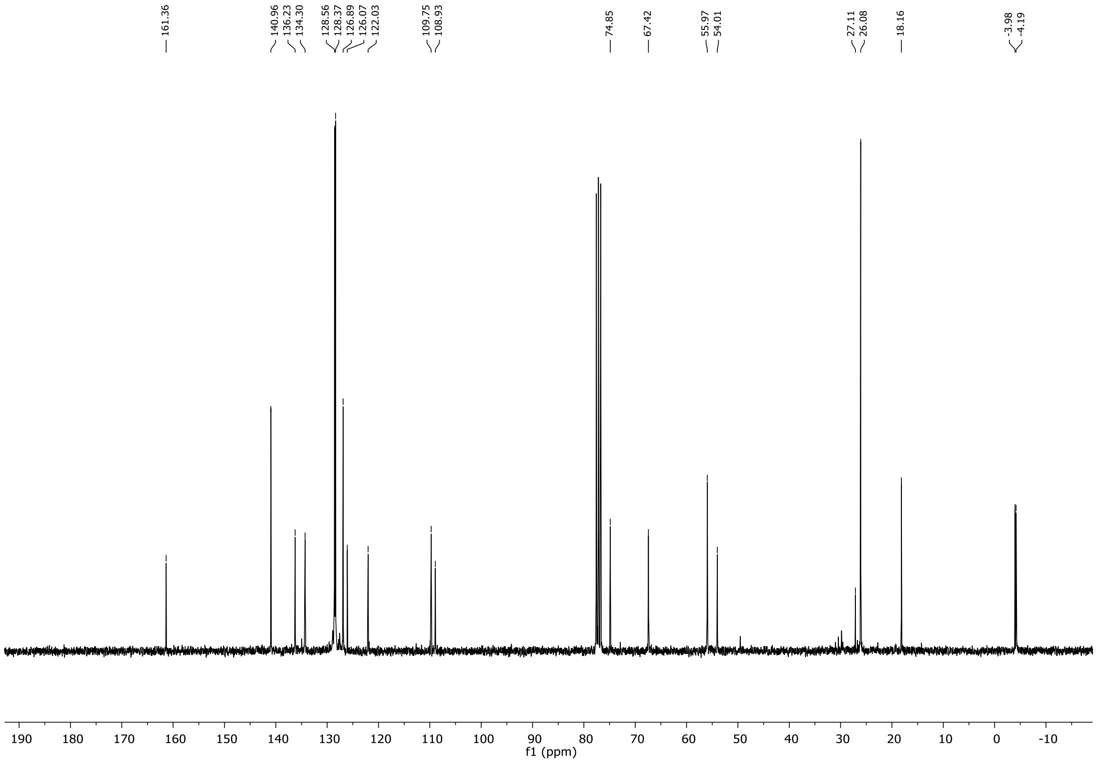

HSQC and HMBC of compound **5**

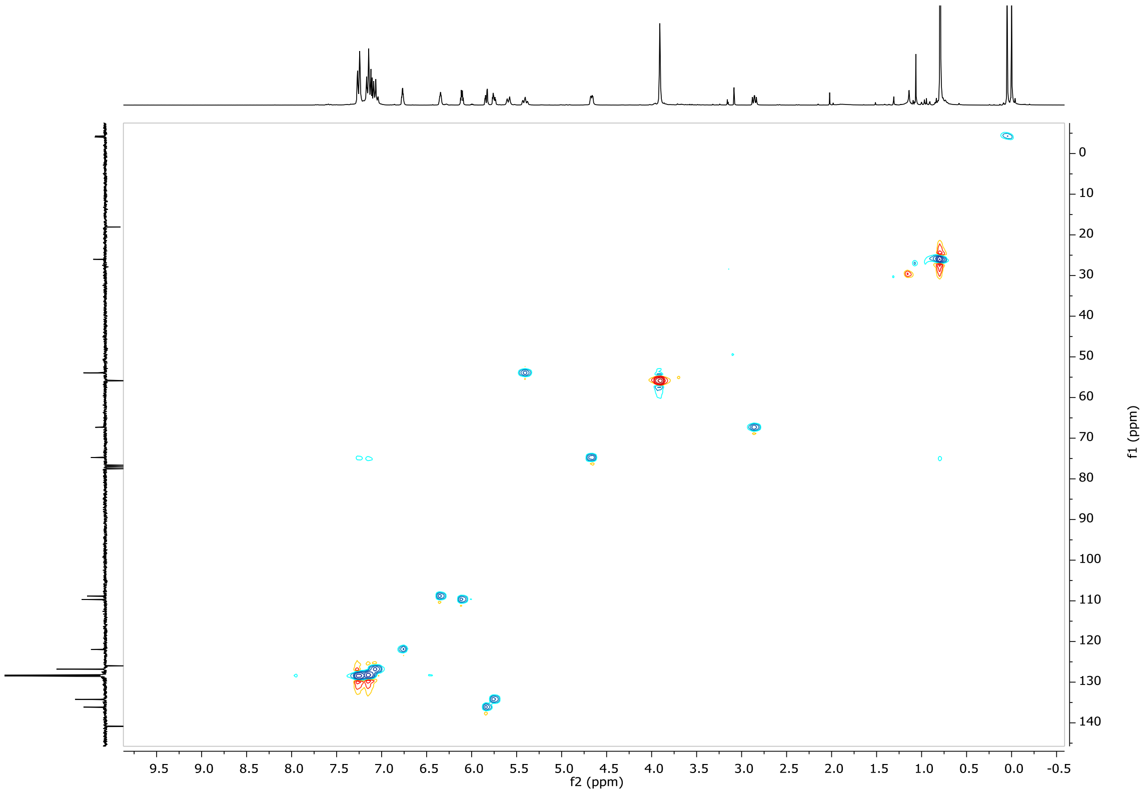

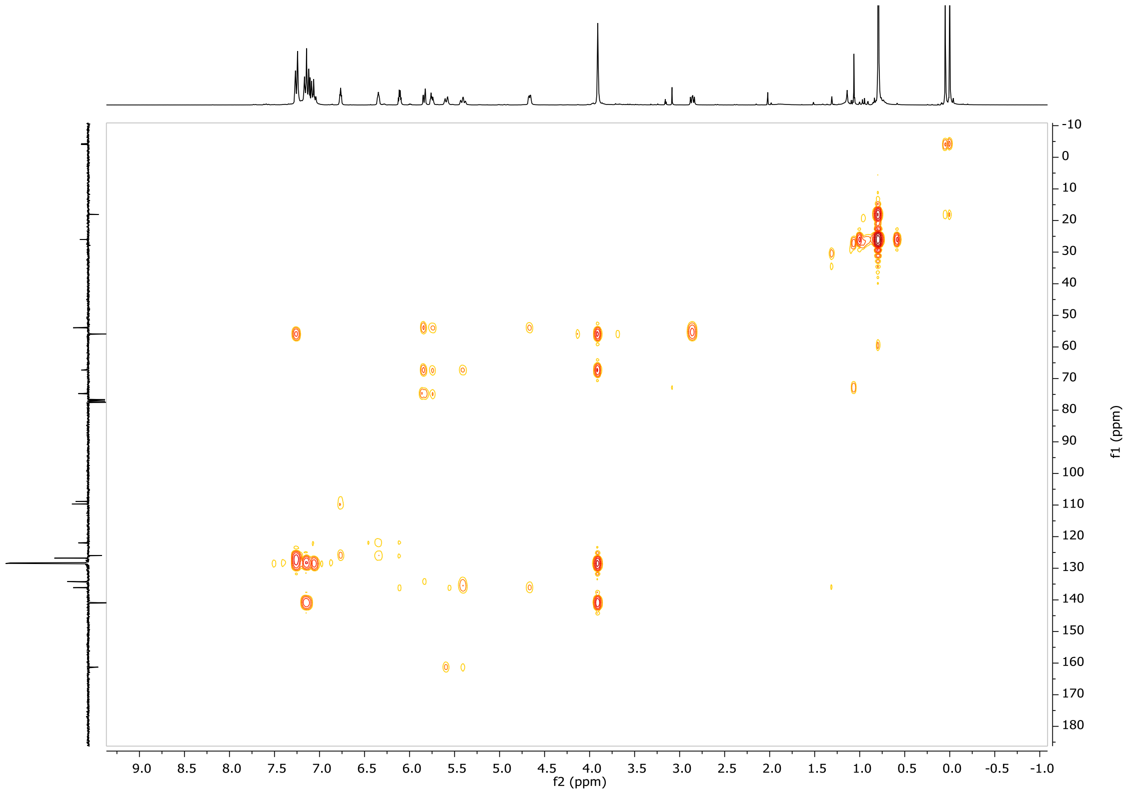

^{1}H NMR (300 MHz, $CDCl_3$) of compound **3**

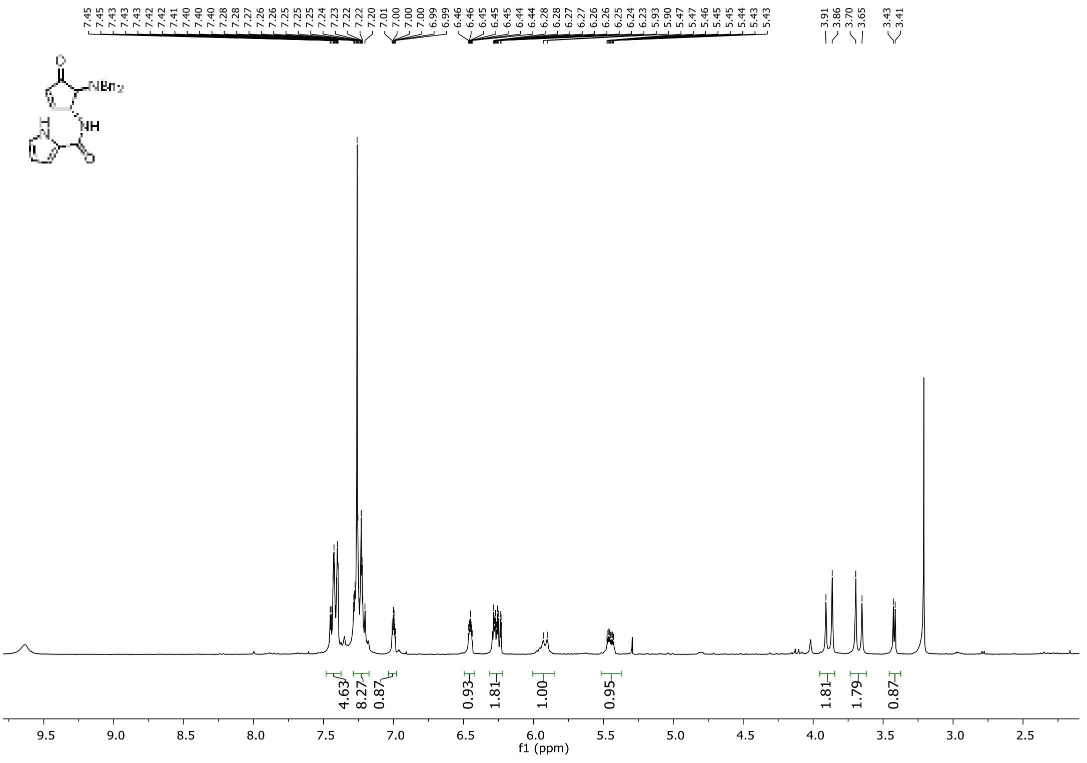

^{1}H NMR (300 MHz, DMSO-d6) of compound **9**

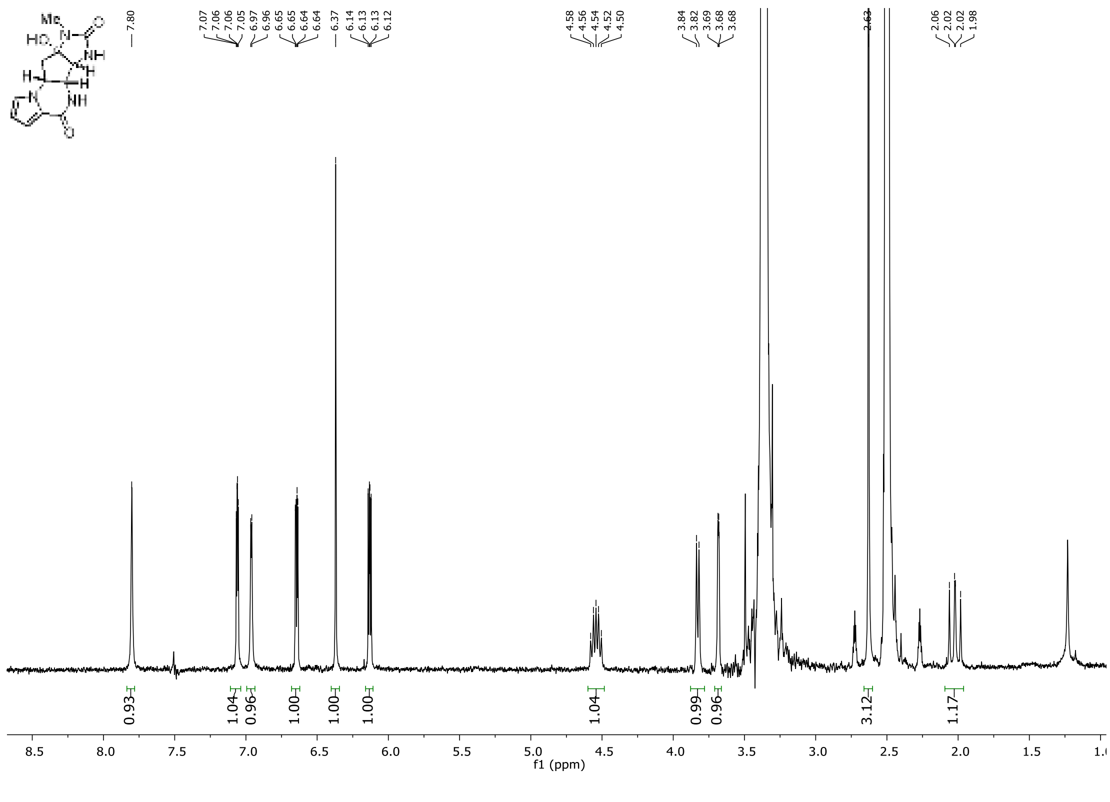

^{1}H NMR (300 MHz, DMSO-d6) and ^{13}C NMR (75 MHz, $CDCl_3$) of **(±)-Agelastatin A (AglA)**

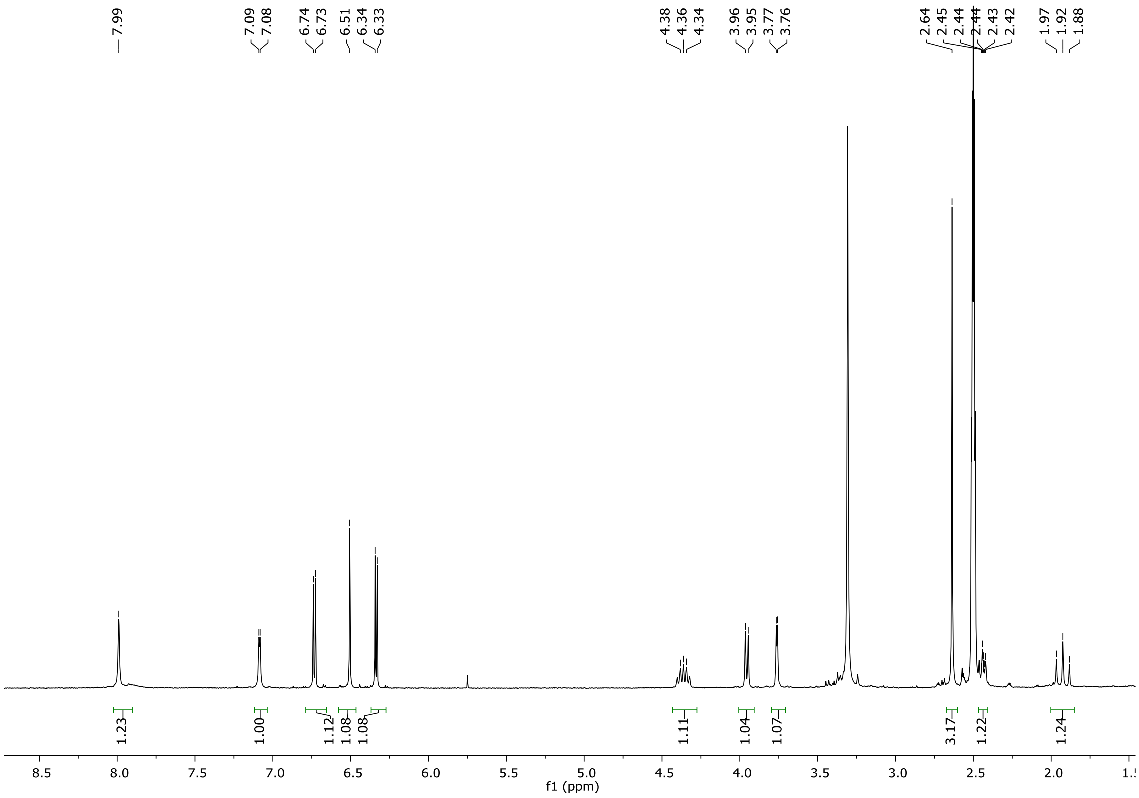

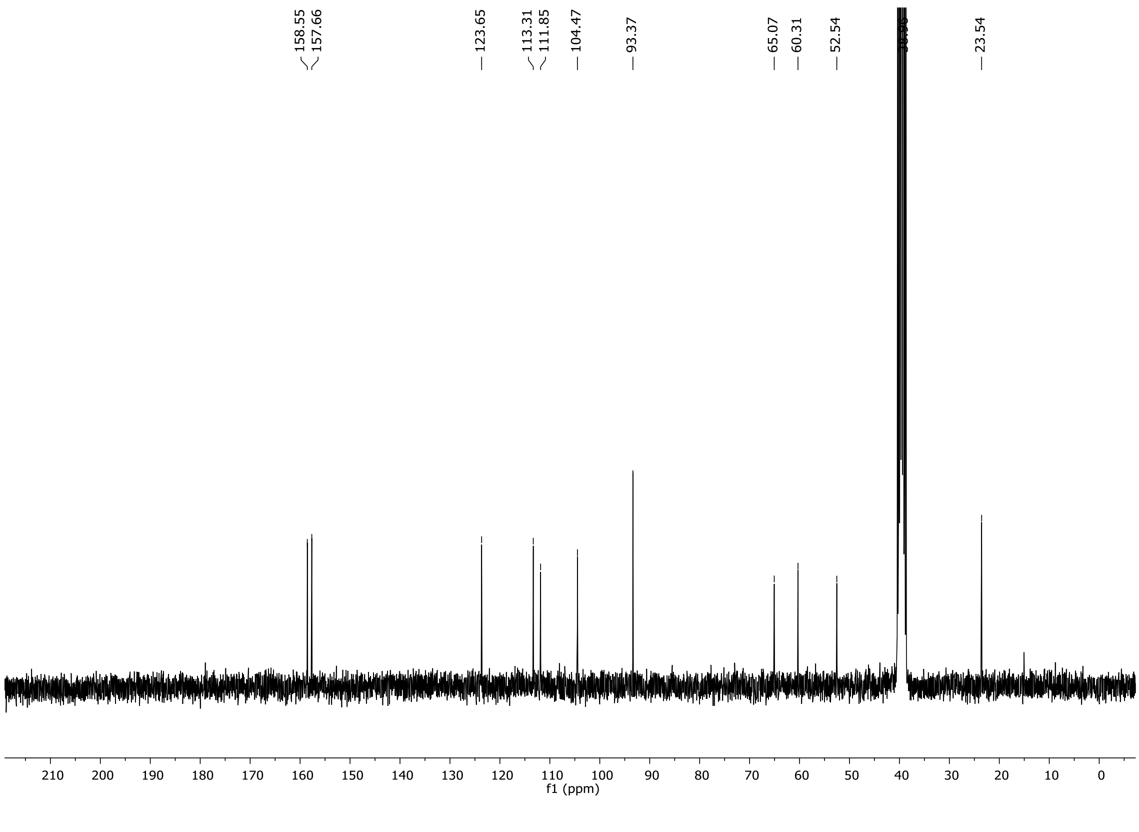

HSQC of **(±)-Agelastatin A (AglA)**

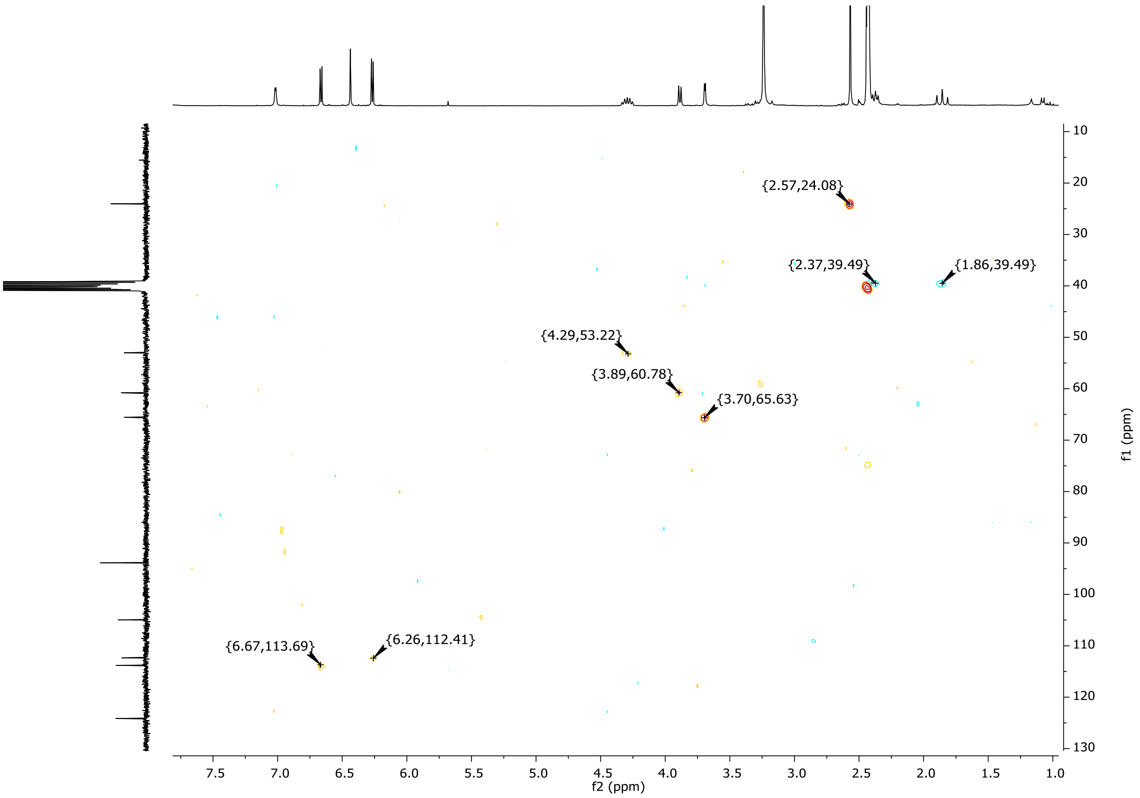

References

(1) a. Davis, F. A.; Deng, J. Asymmetric Total Synthesis of (−)-Agelastatin A Using sulfinimine (N-Sulfinyl Imine) Derived Methodologies. *Org. Lett.* **2005**, 7, 621–623; b. Davis, F. A.; Zhang, J.; Zhang, Y.; Qiu, H. Improved Synthesis of (-)-Agelastatin A. *Synth. Commun.* **2009**, *39*, 1914–1919.

(2) Stout, E. P.; Choi, M. Y.; Castro, J. E.; Molinski, T. F. Potent Fluorinated Agelastatin Analogues for Chronic Lymphocytic Leukemia: Design, Synthesis, and Pharmacokinetic Studies. *J. Med. Chem.* **2014**, *57*, 5085–5093.

(3) D'Ambrosio, M.; Guerriero, A.; Debitus, C.; Ribes, O.; Pusset, J.; Leroy, S.; Pietra, F. Agelastatin A, a New Skeleton Cytotoxic Alkaloid of the Oroidin Family. Isolation from the Axinellid Sponge Agelas Dendromorpha of the Coral Sea. *J. Chem. Soc. Chem. Commun.* **1993**, No. 16, 1305–1306.
